# Supplementary material for: Structural Characterization and Spatial Mapping of Tetrodotoxins in Australian Polyclads
Source: Mar Drugs. 2022 Dec 19;20(12):788. doi: 10.3390/md20120788 (PMC9782485; doi:10.3390/md20120788)
Supplement: Supplementary file 1 [file marinedrugs-20-00788-s001.zip › marinedrugs-2041864-supplementary.pdf]

Supplementary Materials

Structural Characterization and Spatial Mapping of Tetrodotoxins in Australian Polyclads

Justin M. McNab, Matthew T Briggs, Jane E. Williamson, Peter Hoffmann, Jorge Rodriguez and Peter Karuso*

Site	Latitude	Longitude	Months sampled	Number collected	Number of species found
Chowder Bay	-33.8389	151.2546	April-May	11	8
Bottle and Glass point	-33.8475	151.2701	April-August	3	3
Bare Island	-33.9911	151.2317	April-May	5	3
Cronulla	-34.0373	151.1481	June-July	5	1
Jervis Bay	-35.0593	150.7254	July	4	1

Table S1. Australian sites where flatworms were collected for this study

Table S2: List of PRM channels tested during HILIC-MS.

Chemical	PRM precursor	PRM product ions
11-deoxyTTX	304.1139 [1]	176.07701
11-norTTX-6(S)-ol	290.0983 [1]	162.07501
TTX	320.1088 [1]	302.0981 (–H2O)
		162.0661 (quantitative)[2]
		60.0550 (confirmative)[2]
4,9-anhydroTTX	302.0983 [1]	162.07794
6,11 dideoxyTTX	288.1190 [1]	162.07458
5,6,11-trideoxyTTX	272.1241 [1]	254.0931
1-hydroxy-4,4a-anhydro-8-epi-5,6,11-trideoxyTTX	270.1085 [1]	162.06645
4,9-anhydro-8-epi-5,6,11-trideoxy TTX	254.1135 [1]	162.07721

Table S3: GenBank NCBI accession numbers.

Species	Accession number
Stylochus cf mcgrathi	MZ813366
Stylochus cf mcgrathi	MZ813367
Stylochus cf mcgrathi	MZ813368
Stylochus cf mcgrathi	MZ813369
Stylochus cf mcgrathi	MZ813370
Pseudoceros sp 3	MZ813371
Stylochus sp 4	MZ813372
Pseudoceros sp 1	MZ813373
Cycloporus sp	MZ813374
Eurylepta sp	MZ813375
Pseudoceros sp 2	MZ813376
Pseudoceros cf velutinus	MZ813377
Pseudoceros cf velutinus	MZ813378
Thysanozoon brocchii	MZ813379
Thysanozoon brocchii	MZ813380
Notoplana cf longiducta	MZ813381
Stylochus cf mcgrathi	MZ813382
Stylochus cf mcgrathi	MZ813383
Echinoplana cf celerrima	MZ813384

Supplementary PRM data for all compounds detected

TTX (1) m/z 320.1088

Samples were determined to have analytes based on identical peaks and retention times (RT). Firstly, tetrodotoxin samples were compared directly to the analytical standard (ABCAM, Cat. No: ab120054), the samples that contained a peak at the mass range 320.1088 m/z contained TTX. Additionally, daughter ions reported in Bane, et al. [3] were also investigated and samples that retained these ions were considered to contain TTX. These samples were: *S. mcgrathi* (RT:18.01), *S. mcgrathi* (RT: 17.41), *S. mcgrathi* (RT:18.29), *S. mcgrathi* (RT:18.32), *S. mcgrathi* (RT:18.25), *S. mcgrathi* (RT:17.55). These samples were determined to contain TTX because of the retention of 162.06 and 178.06 at the indicated time of around 17 or 18 mins (one run of samples eluted at around 17 minutes however the other samples and standards all eluted around 18 minutes).

Aqueous standard

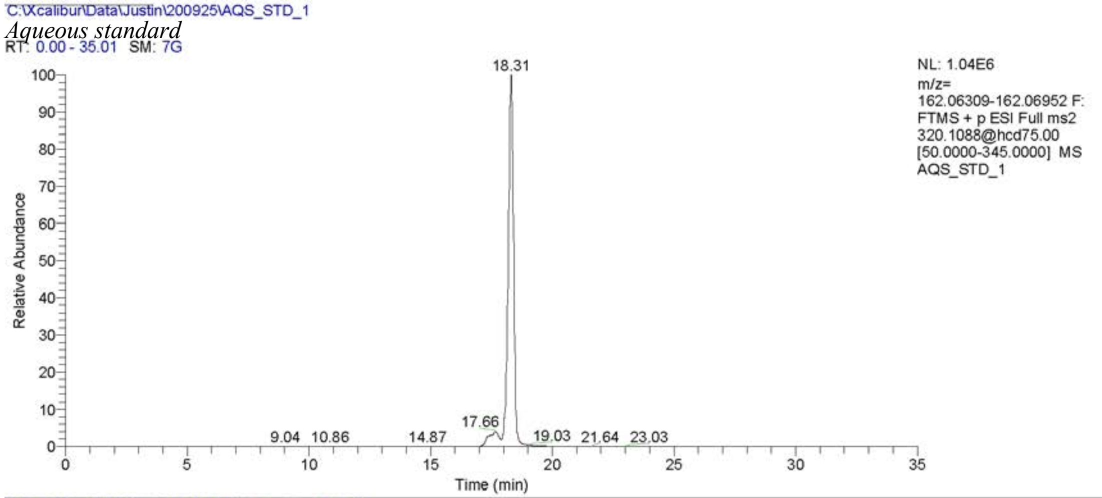

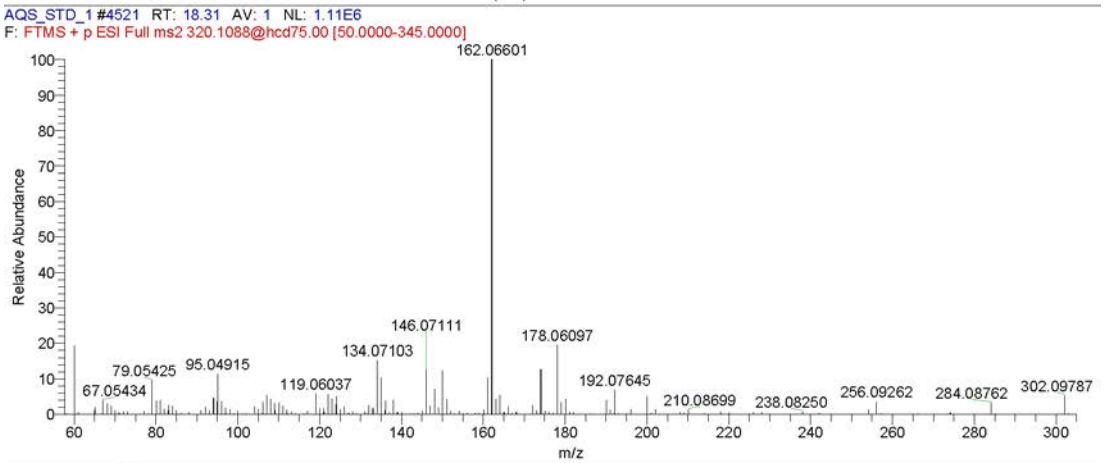

AQS_STD_1#4521 RT: 18.31

F: FTMS + p ESI Full ms2 320.1088@hcd75.00 [50.0000-345.0000]

m/z= 50.00000-750.00000

m/z	Intensity	Relative
135.05511	114891.7	10.25
146.07111	141177.1	12.59
148.05019	82008.5	7.32
150.06610	138665.0	12.37
161.05823	116700.0	10.41
162.06601	1120990.1	100.00
164.08167	61383.7	5.48
174.06602	145958.7	13.02
178.06097	221593.9	19.77
192.07645	76122.3	6.79
200.04501	59051.8	5.27
302.09787	59252.5	5.29

C:\Xcalibur\...\200923\OFF1_extract_1

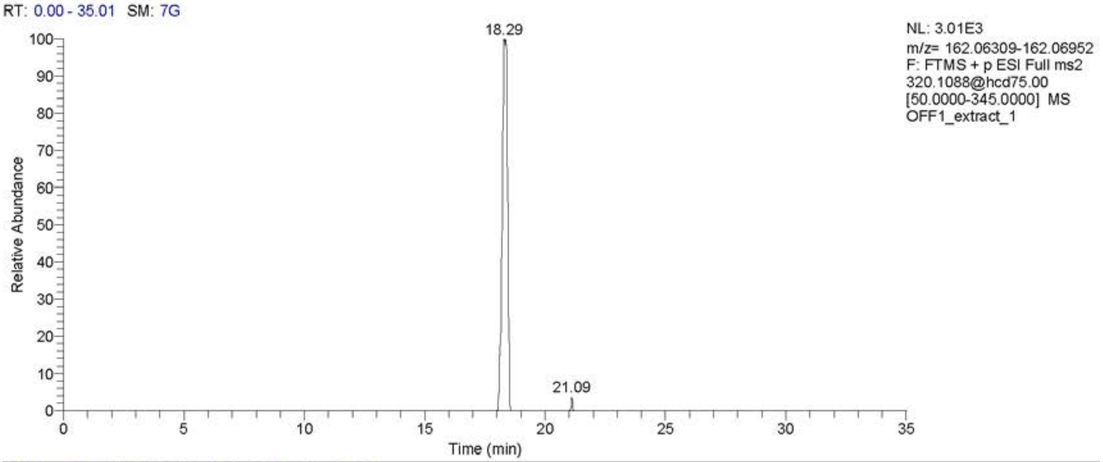

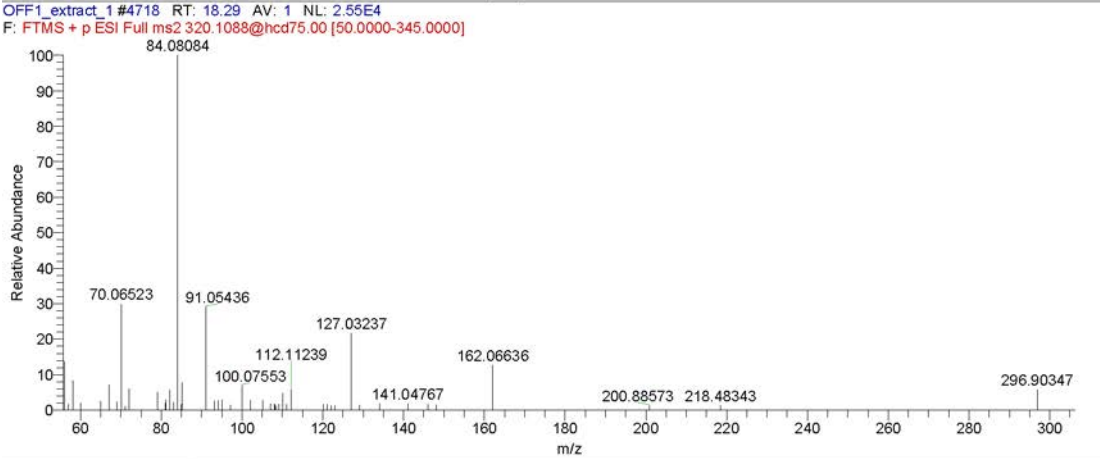

OFF1_extract_1#4718 RT: 18.29
F: FTMS + p ESI Full ms2 320.1088@hcd75.00 [50.0000-345.0000]
m/z= 55.75695-306.27144

m/z	Intensity	Relative
85.08411	2017.2	7.74
91.05436	7447.7	28.58
94.06532	707.4	2.71
95.04892	765.7	2.94
100.07553	1830.0	7.02
105.07022	715.0	2.74
110.07139	1288.9	4.95
112.11239	1549.7	5.95
127.03237	5818.6	22.33
162.06636	3318.5	12.73
296.90347	1467.3	5.63

S. mcgrathi

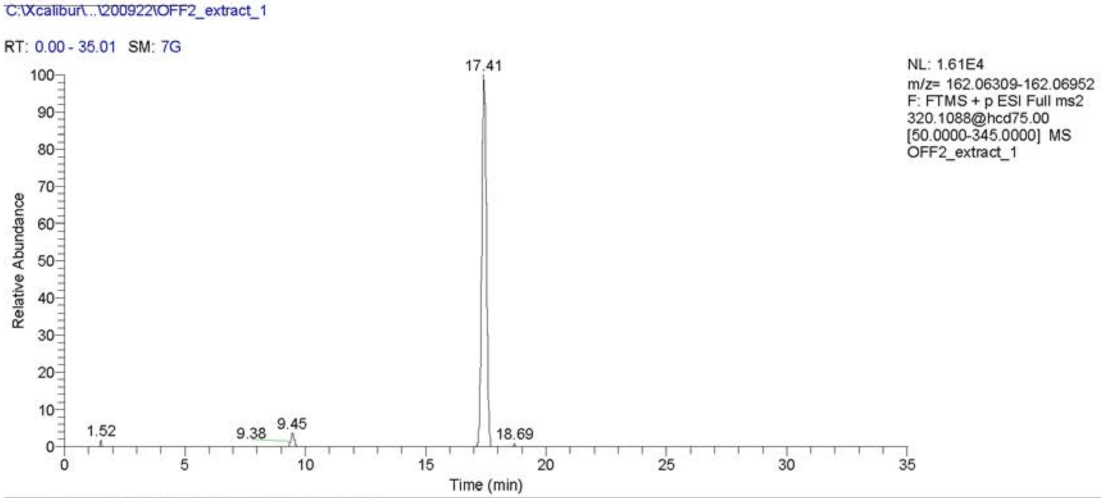

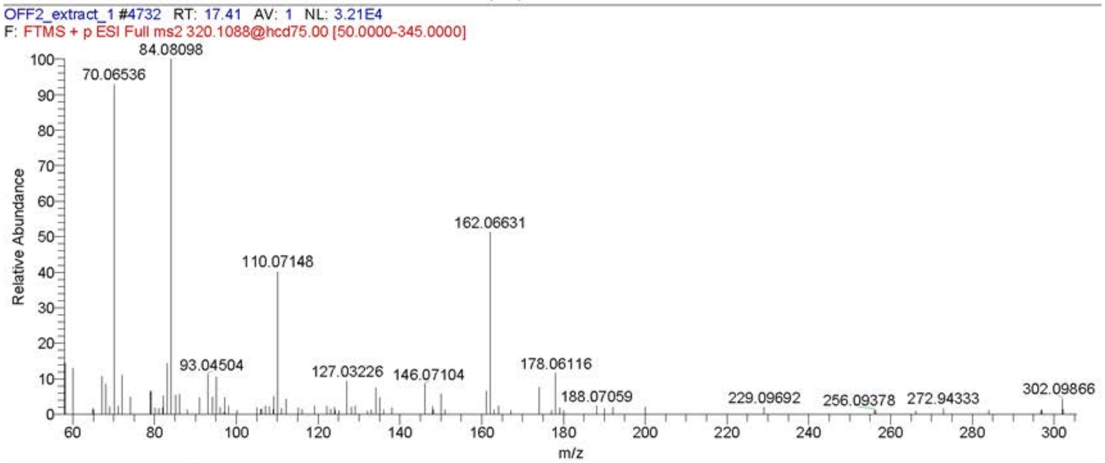

OFF2_extract_1#4733 RT: 17.41 SM: 7G
F: FTMS + p ESI Full ms2 320.1088@hcd75.00 [50.0000-345.0000]
m/z= 50.00000-750.00000

m/z	Intensity	Relative
55.05452	2295.1	6.78
56.04985	6208.4	18.34
58.06542	3984.6	11.77
60.05595	4660.9	13.77
67.05432	2283.7	6.75
68.04961	2236.0	6.60
70.06535	33855.9	100.00
72.08097	2911.4	8.60
82.06520	2282.4	6.74
83.06055	4161.6	12.29
84.04475	2559.7	7.56
84.08097	28244.6	83.43
93.04488	3213.3	9.49
95.06044	2832.7	8.37
109.07631	2125.7	6.28

S. mcgrathi

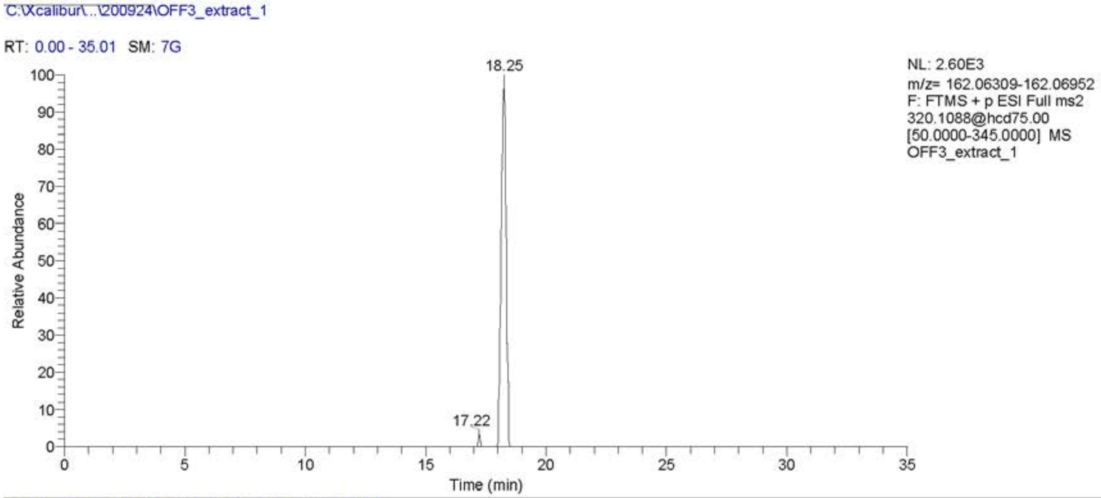

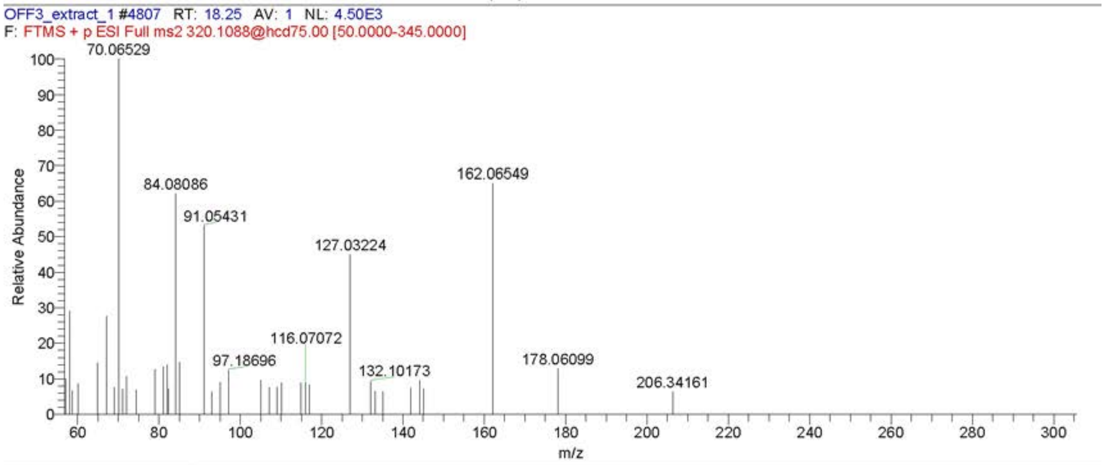

OFF3_extract_1#4807 RT: 18.25
F: FTMS + p ESI Full ms2 320.1088@hcd75.00 [50.0000-345.0000]
m/z= 50.00000-750.00000

m/z	Intensity	Relative
85.02833	684.6	15.15
91.05431	2412.2	53.39
97.18696	596.4	13.20
105.06971	448.5	9.93
127.03224	2034.1	45.02
162.05376	473.2	10.47
162.06549	2972.8	65.79
178.06099	604.8	13.39

S. mcgrathi

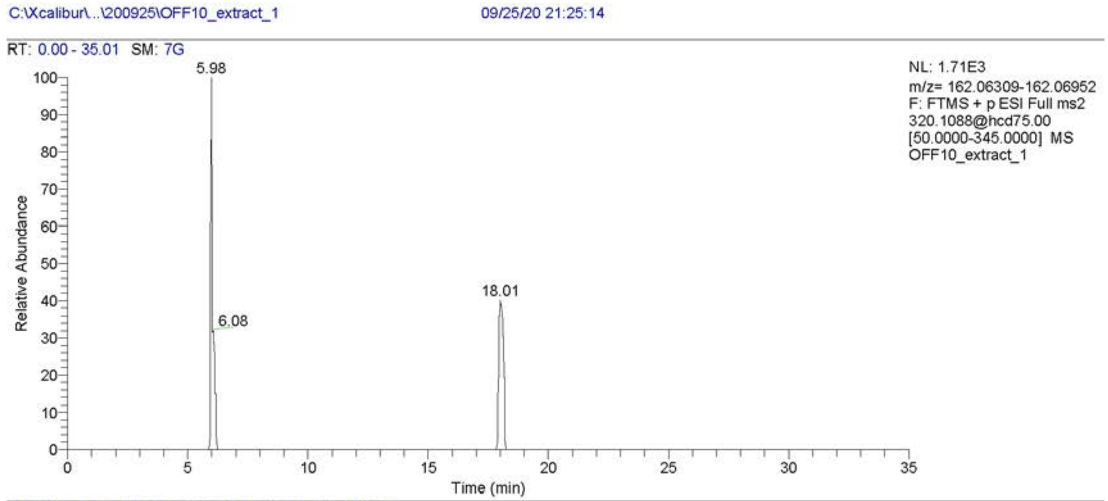

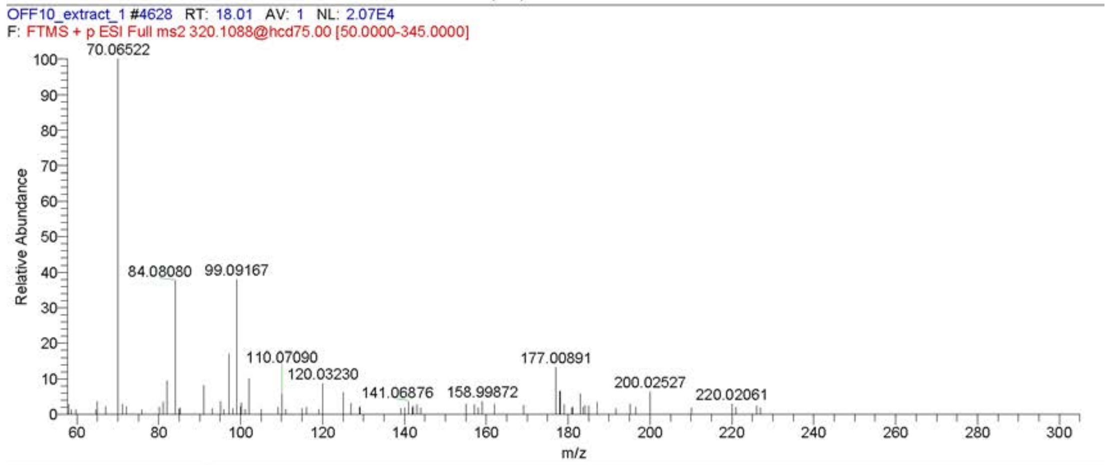

OFF10_extract_1#4626 RT: 18.00
F: FTMS + p ESI Full ms2 320.1088@hcd75.00 [50.0000-345.0000]
m/z= 50.00000-750.00000

m/z	Intensity	Relative
99.09170	7864.5	38.20
100.09492	759.1	3.69
102.02180	1708.0	8.30
120.03244	2167.5	10.53
125.03793	733.2	3.56
162.06519	670.9	3.26
175.07358	676.7	3.29
177.00934	3127.0	15.19
178.01106	679.9	3.30
179.00737	1240.6	6.03
187.07507	716.8	3.48
200.02545	1589.5	7.72
219.01979	756.8	3.68

S. mcgrathi

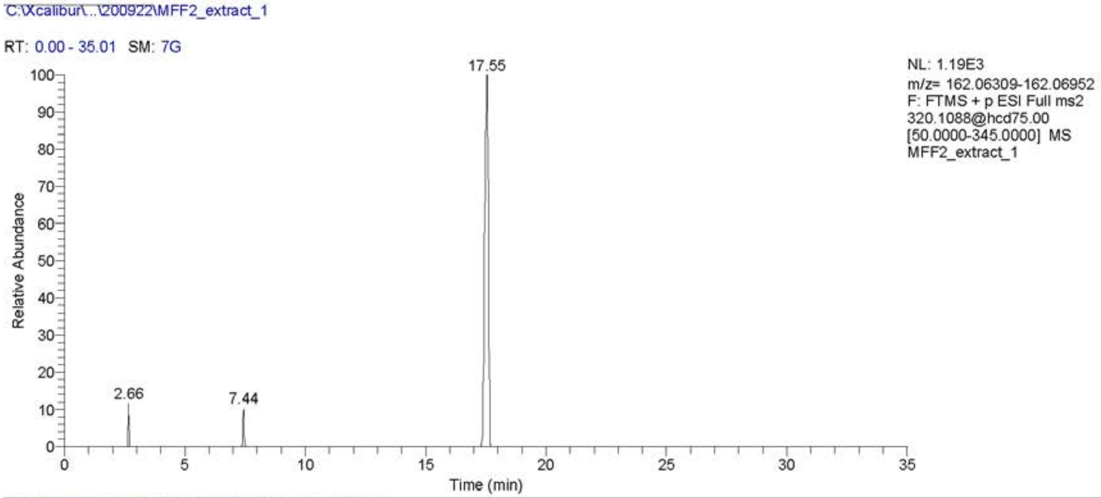

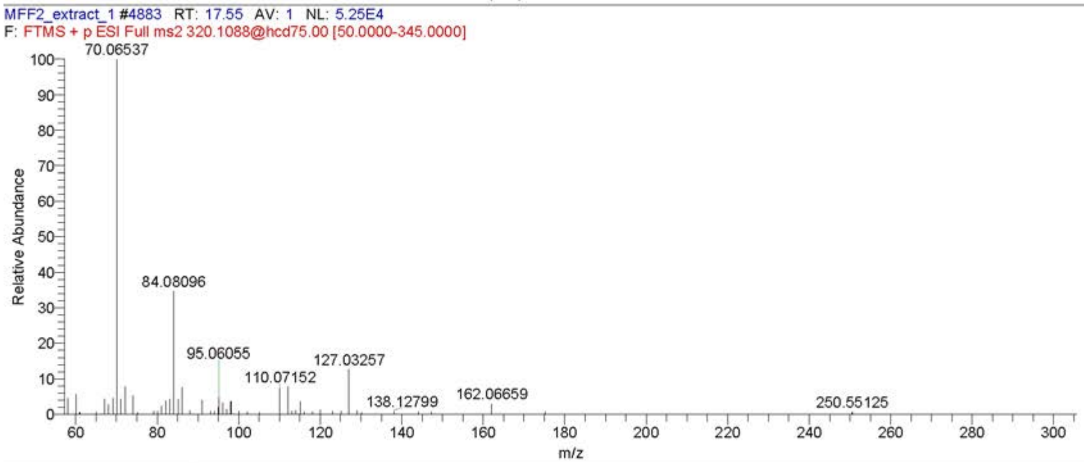

MFF2_extract_1#4881 RT: 17.54
F: FTMS + p ESI Full ms2 320.1088@hcd75.00 [50.0000-345.0000]
m/z= 57.95455-305.90909

m/z	Intensity	Relative
60.05600	4500.8	7.03
68.04964	1621.1	2.53
70.06535	64007.4	100.00
71.04932	2917.1	4.56
72.08091	3858.6	6.03
82.06514	1850.7	2.89
83.06040	1905.2	2.98
84.04457	5195.8	8.12
84.08096	21896.0	34.21
85.08427	2200.6	3.44
86.09666	4293.7	6.71
91.05445	1834.0	2.87
95.06046	3369.4	5.26
96.04449	1651.7	2.58
98.06011	2509.3	3.92

S. mcgrathi

11-deoxyTTX (2) m/z 304.1139

Samples were determined to contain this analyte when either the daughter ion 176.07 or 286.07 was observed at a similar time in each sample. These daughter ions were identified in Bane, Lehane, Dikshit, O'Riordan and Furey [1]. The samples that were determined to contain this analyte were, *P. velutinus* (RT: 6.17), *Pseudoceros sp. 2* (RT: 6.20), *Stylochus sp. 1* (RT: 6.15), *S. mcgrathi* (RT: 6.19), *S. mcgrathi* (RT: 6.02), *Stylochus sp. 1* (RT: 6.11), *S. mcgrathi* (RT: 6.15), *E. celerrima* (RT: 6.14), *and S. mcgrathi* (RT: 6.06).

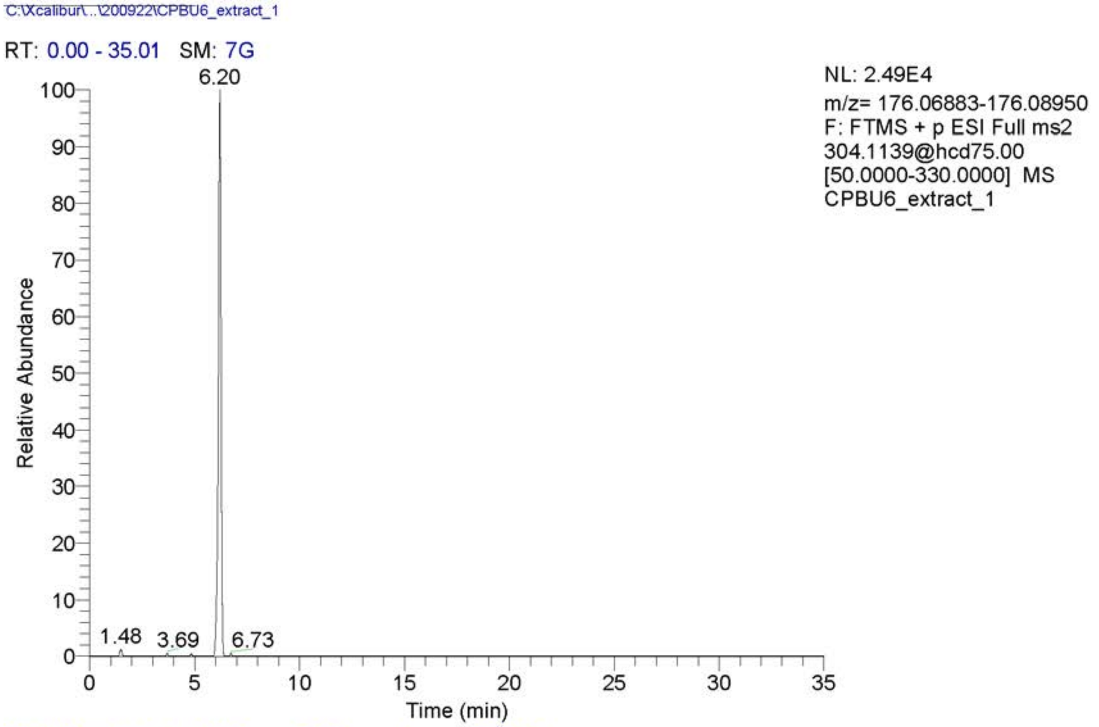

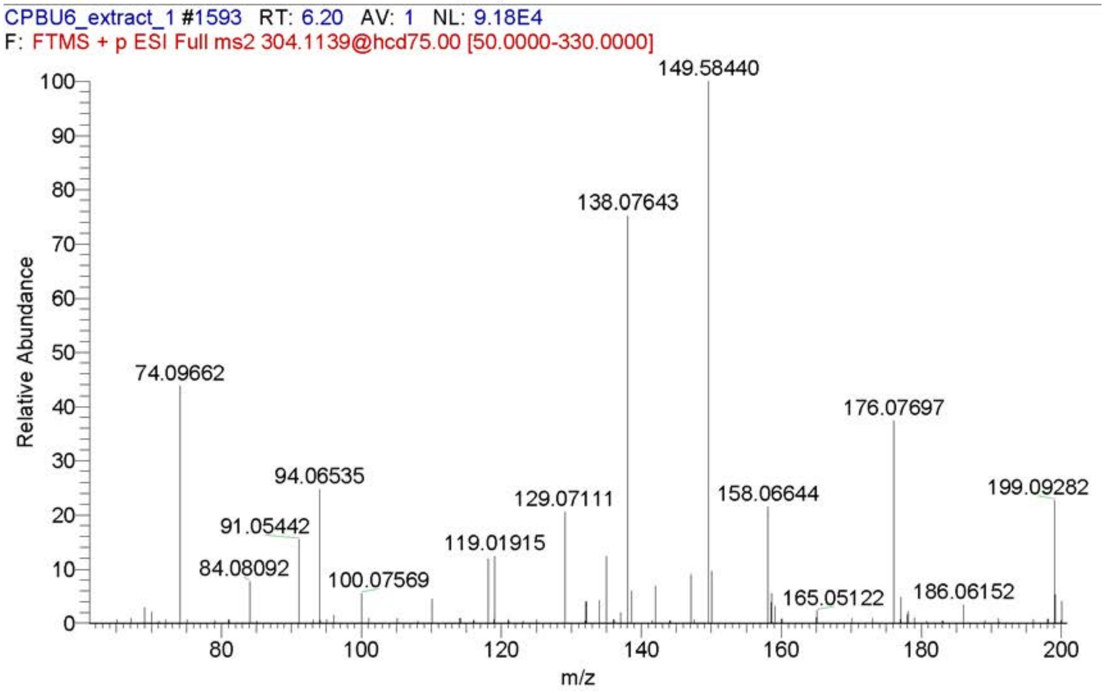

Pseudoceros sp 2

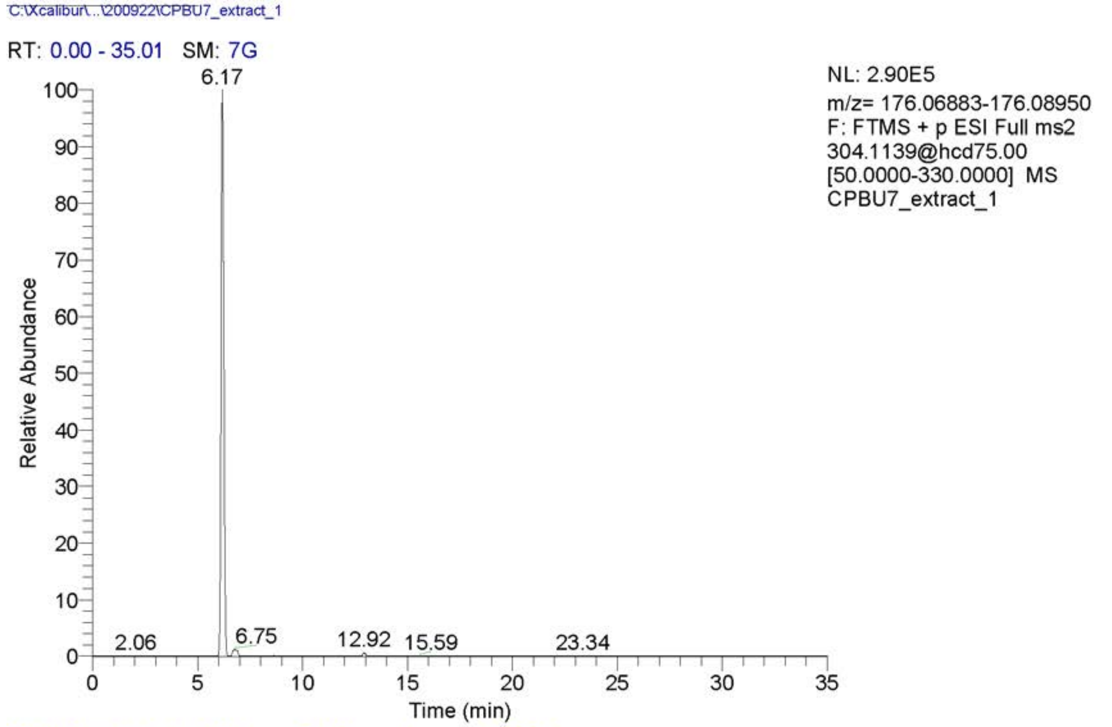
C:\Xcalibur\...\200922\CPBU7_extract_1
RT: 0.00 - 35.01 SM: 7G
NL: 2.90E5
m/z= 176.06883-176.08950
F: FTMS + p ESI Full ms2
304.1139@hcd75.00
[50.0000-330.0000] MS
CPBU7_extract_1
6.17
2.06
6.75
12.92
15.59
23.34
Relative Abundance
Time (min)

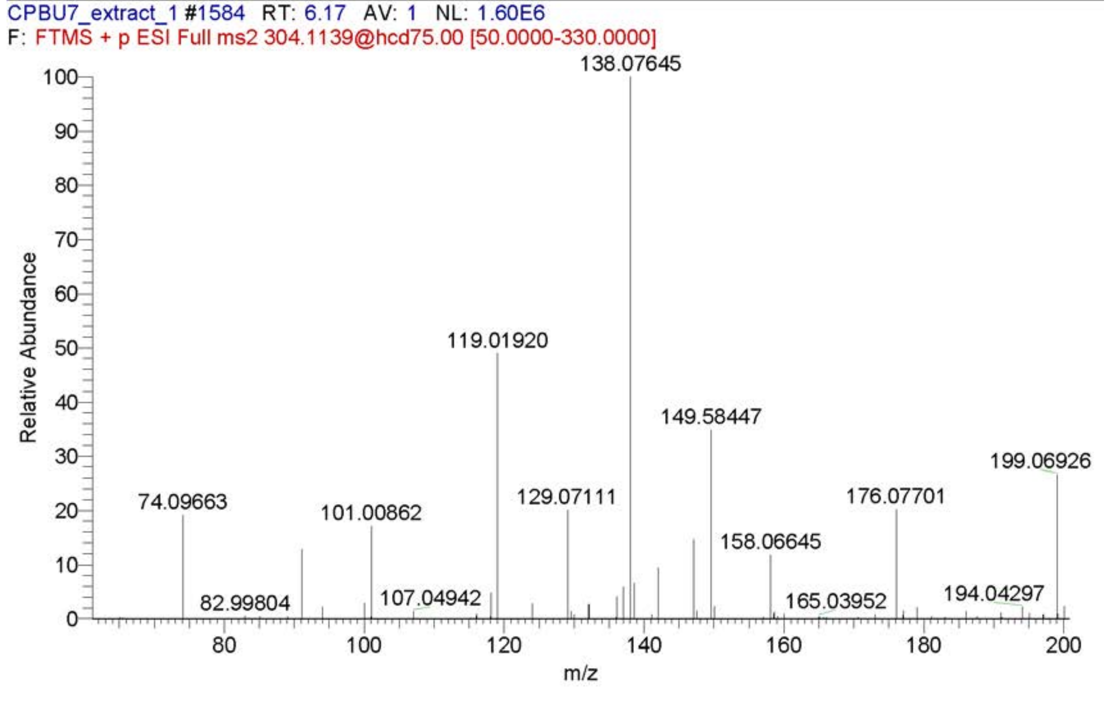
CPBU7_extract_1 #1584 RT: 6.17 AV: 1 NL: 1.60E6
F: FTMS + p ESI Full ms2 304.1139@hcd75.00 [50.0000-330.0000]
138.07645
119.01920
149.58447
199.06926
74.09663
129.07111
176.07701
101.00862
158.06645
82.99804
107.04942
165.03952
194.04297
Relative Abundance
m/z

P. velutinus

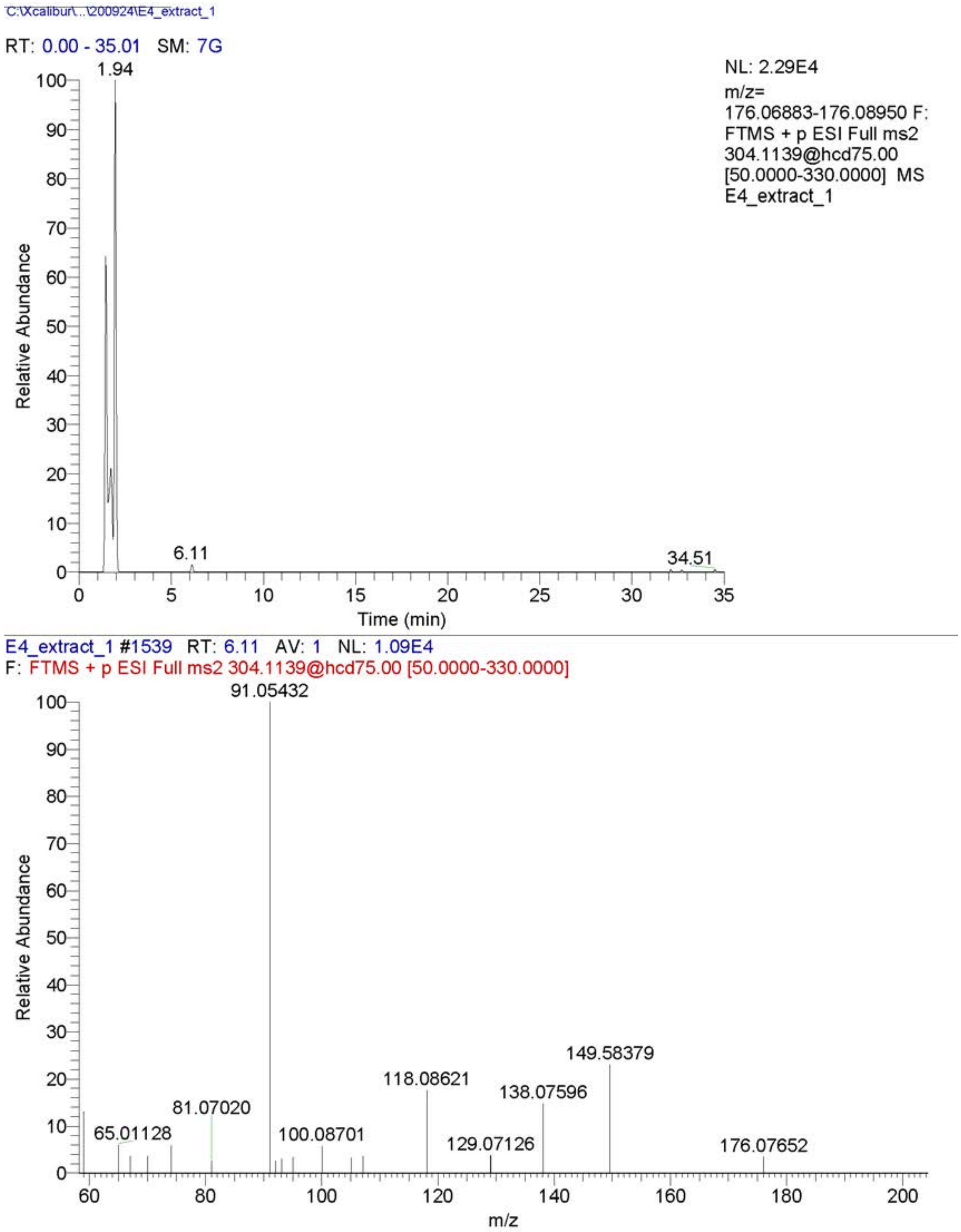
C:\Xcalibur\...\200924\E4_extract_1
RT: 0.00 - 35.01 SM: 7G
NL: 2.29E4
m/z=
176.06883-176.08950 F:
FTMS + p ESI Full ms2
304.1139@hcd75.00
[50.0000-330.0000] MS
E4_extract_1
1.94
6.11
34.51
Relative Abundance
Time (min)
E4_extract_1 #1539 RT: 6.11 AV: 1 NL: 1.09E4
F: FTMS + p ESI Full ms2 304.1139@hcd75.00 [50.0000-330.0000]
91.05432
149.58379
118.08621
138.07596
81.07020
65.01128
100.08701
129.07126
176.07652
Relative Abundance
m/z

E. celerrima

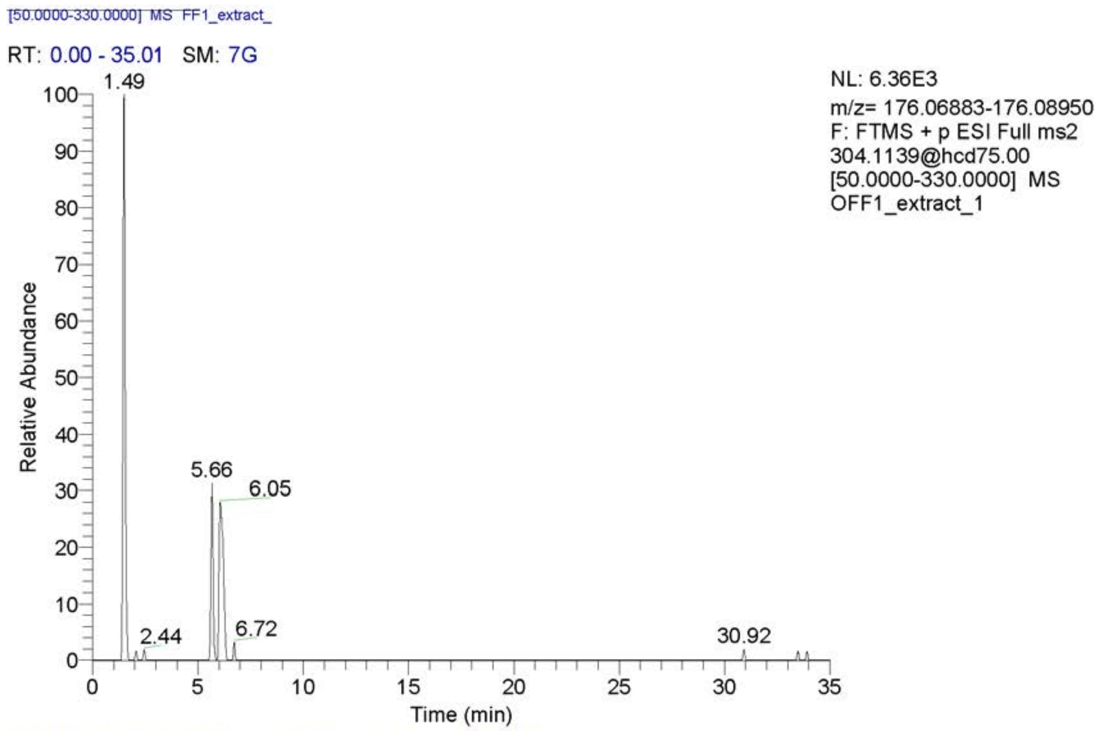
[50.0000-330.0000] MS FF1_extract_
RT: 0.00 - 35.01 SM: 7G
NL: 6.36E3
m/z= 176.06883-176.08950
F: FTMS + p ESI Full ms2 304.1139@hcd75.00 [50.0000-330.0000] MS OFF1_extract_1
1.49
5.66
6.05
2.44
6.72
30.92
Relative Abundance
Time (min)

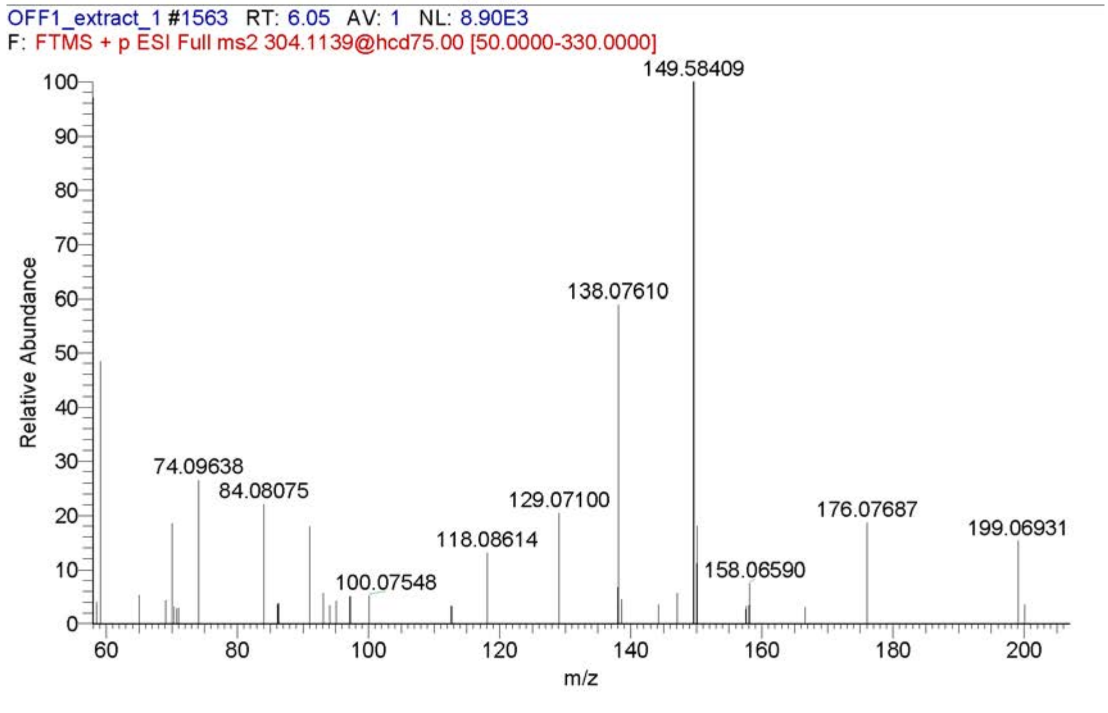
OFF1_extract_1 #1563 RT: 6.05 AV: 1 NL: 8.90E3
F: FTMS + p ESI Full ms2 304.1139@hcd75.00 [50.0000-330.0000]
149.58409
138.07610
74.09638
84.08075
129.07100
176.07687
199.06931
118.08614
158.06590
100.07548
Relative Abundance
m/z

S. mcgrathi

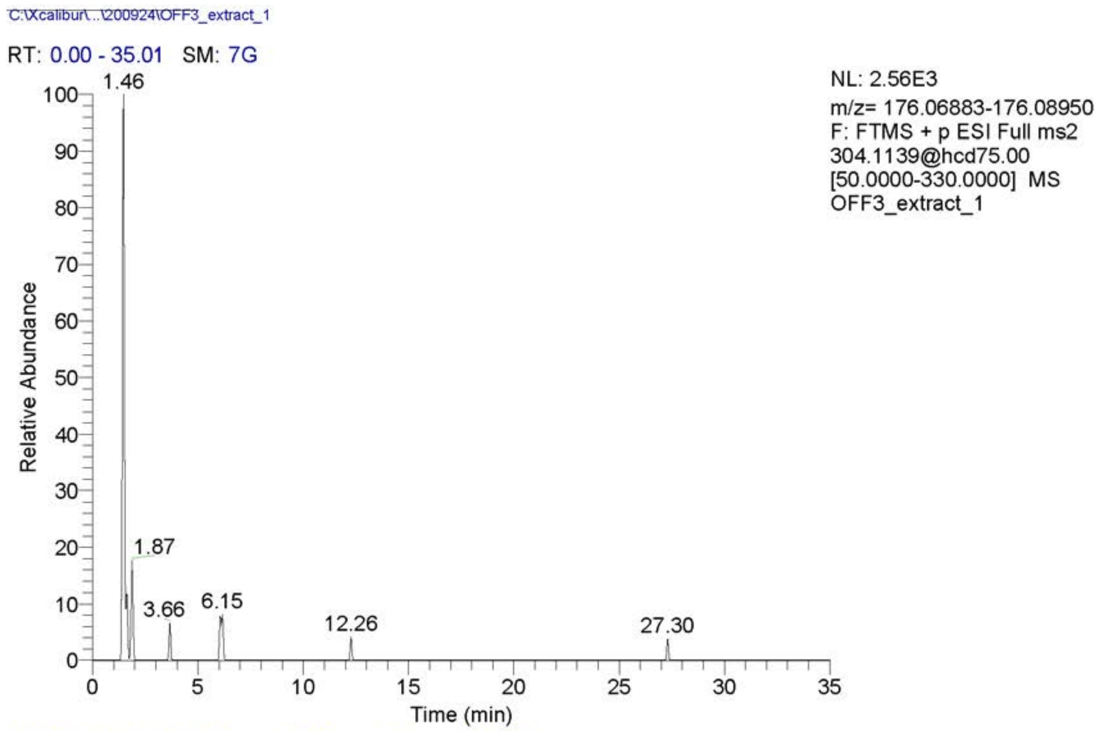
C:\Xcalibur\...\200924\OFF3_extract_1
RT: 0.00 - 35.01 SM: 7G
NL: 2.56E3
m/z= 176.06883-176.08950
F: FTMS + p ESI Full ms2
304.1139@hcd75.00
[50.0000-330.0000] MS
OFF3_extract_1
1.46
1.87
3.66
6.15
12.26
27.30
Relative Abundance
Time (min)

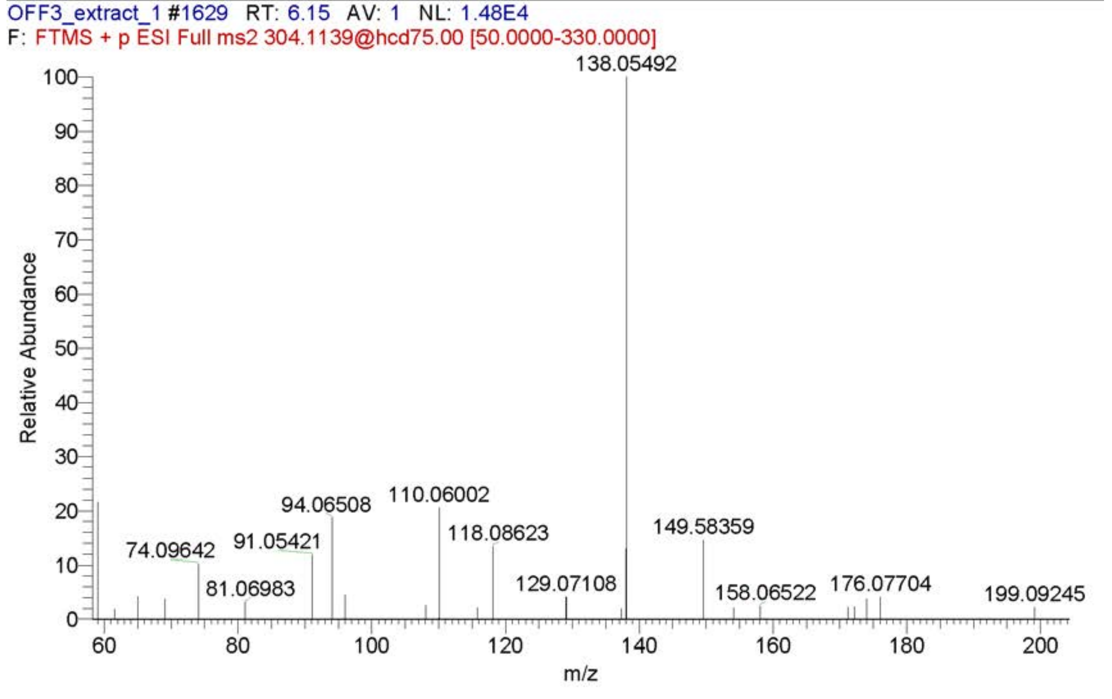
OFF3_extract_1 #1629 RT: 6.15 AV: 1 NL: 1.48E4
F: FTMS + p ESI Full ms2 304.1139@hcd75.00 [50.0000-330.0000]
138.05492
94.06508
110.06002
74.09642
91.05421
118.08623
149.58359
81.06983
129.07108
158.06522
176.07704
199.09245
Relative Abundance
m/z

S. mcgrathi

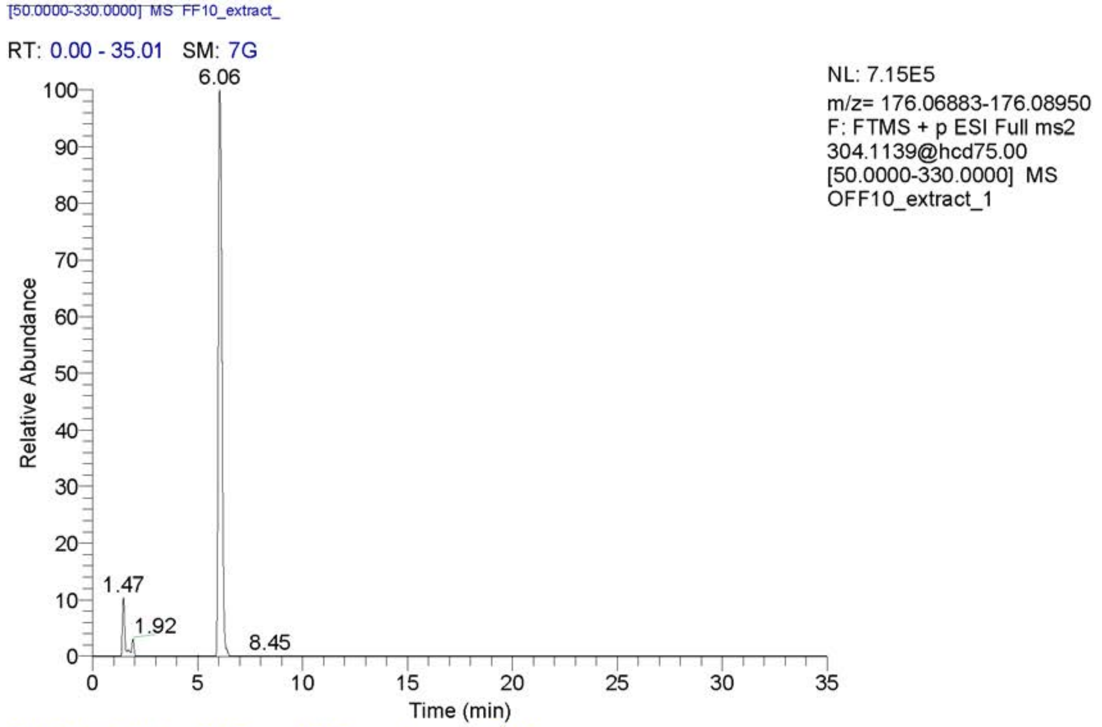
[50.0000-330.0000] MS FF10_extract_
RT: 0.00 - 35.01 SM: 7G
NL: 7.15E5
m/z= 176.06883-176.08950
F: FTMS + p ESI Full ms2
304.1139@hcd75.00
[50.0000-330.0000] MS
OFF10_extract_1
6.06
1.47
1.92
8.45
Relative Abundance
Time (min)

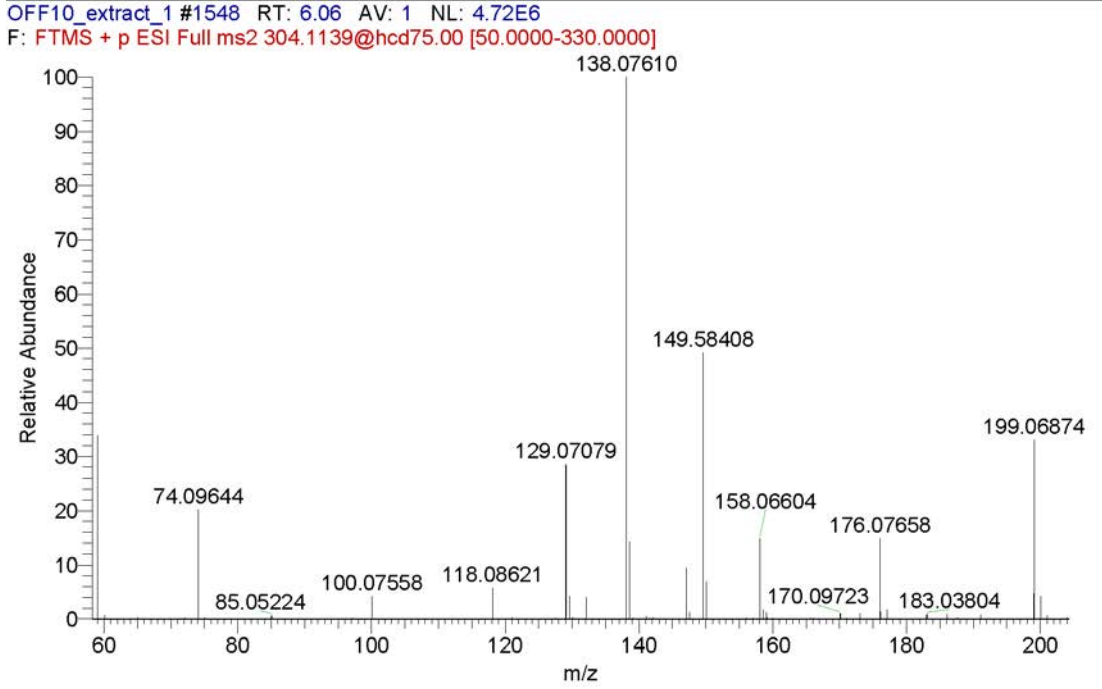
OFF10_extract_1 #1548 RT: 6.06 AV: 1 NL: 4.72E6
F: FTMS + p ESI Full ms2 304.1139@hcd75.00 [50.0000-330.0000]
138.07610
149.58408
199.06874
129.07079
74.09644
158.06604
176.07658
118.08621
100.07558
85.05224
170.09723
183.03804
Relative Abundance
m/z

S. mcgrathi

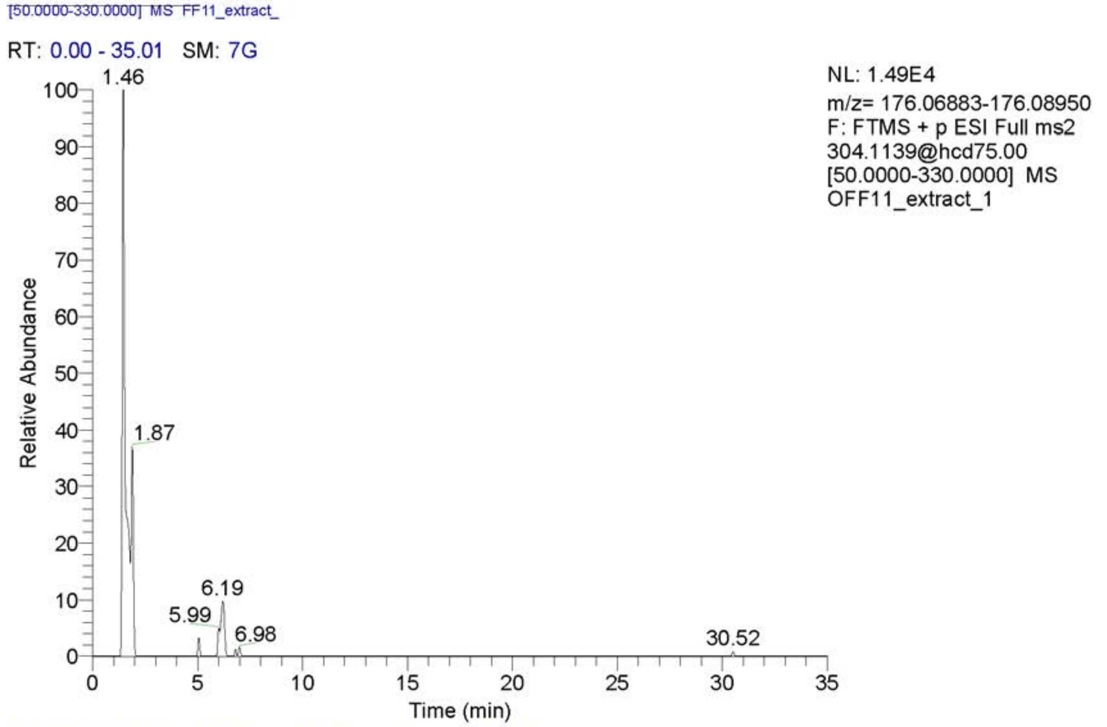
[50.0000-330.0000] MS FF11_extract_
RT: 0.00 - 35.01 SM: 7G
NL: 1.49E4
m/z= 176.06883-176.08950
F: FTMS + p ESI Full ms2 304.1139@hcd75.00 [50.0000-330.0000] MS OFF11_extract_1
1.46
1.87
6.19
5.99
6.98
30.52
Relative Abundance
Time (min)

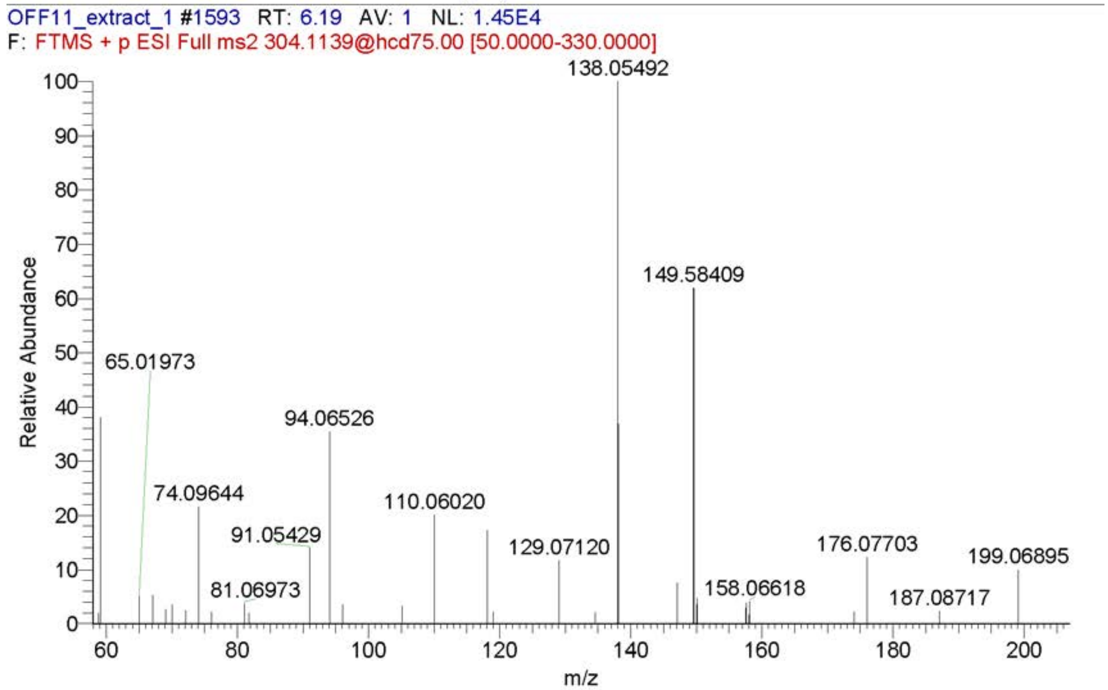
OFF11_extract_1 #1593 RT: 6.19 AV: 1 NL: 1.45E4
F: FTMS + p ESI Full ms2 304.1139@hcd75.00 [50.0000-330.0000]
138.05492
149.58409
65.01973
94.06526
74.09644
110.06020
91.05429
129.07120
176.07703
199.06895
81.06973
158.06618
187.08717
Relative Abundance
m/z

S. mcgrathi

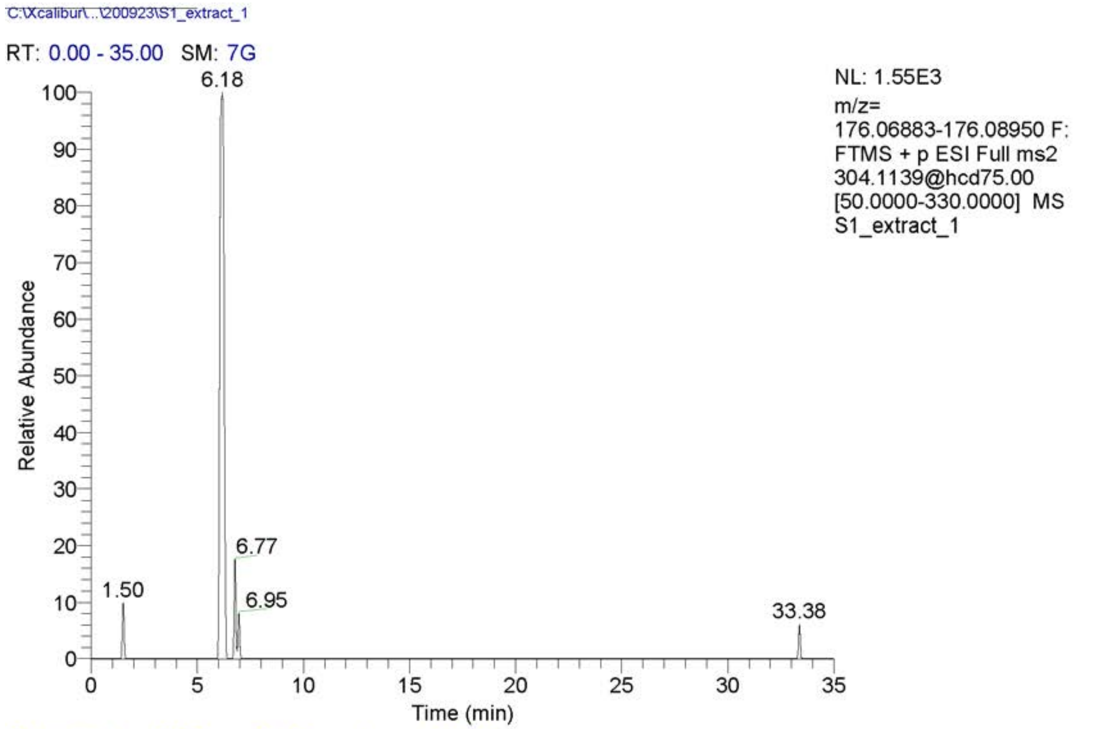

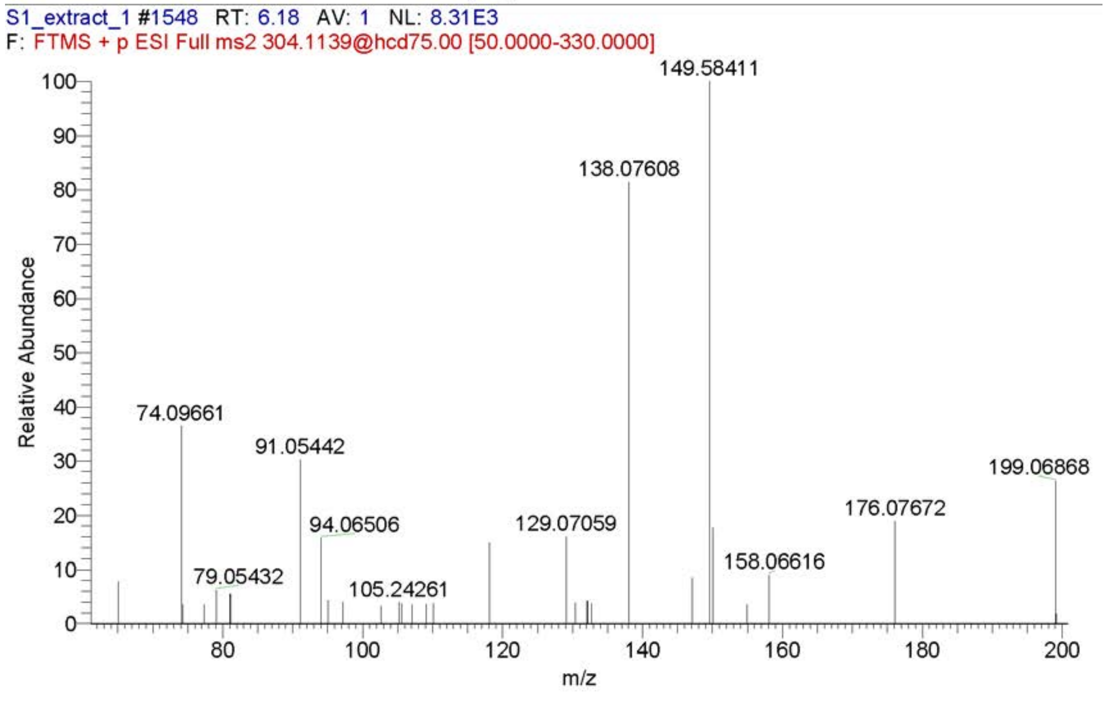

Stylochus sp 1.

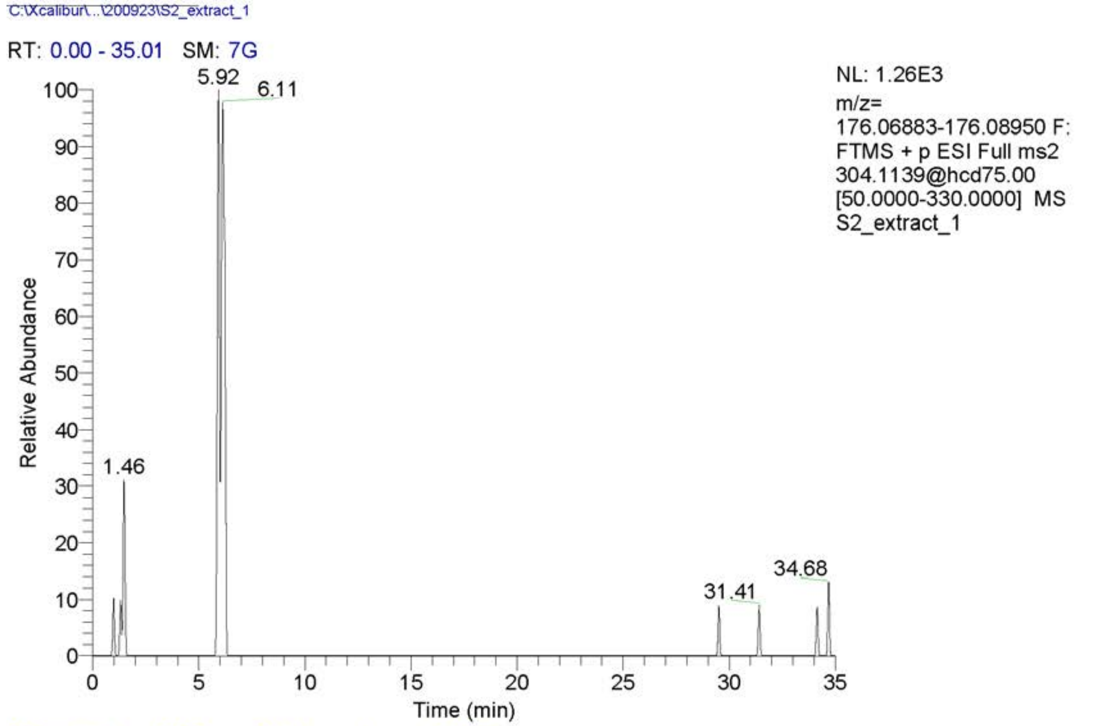

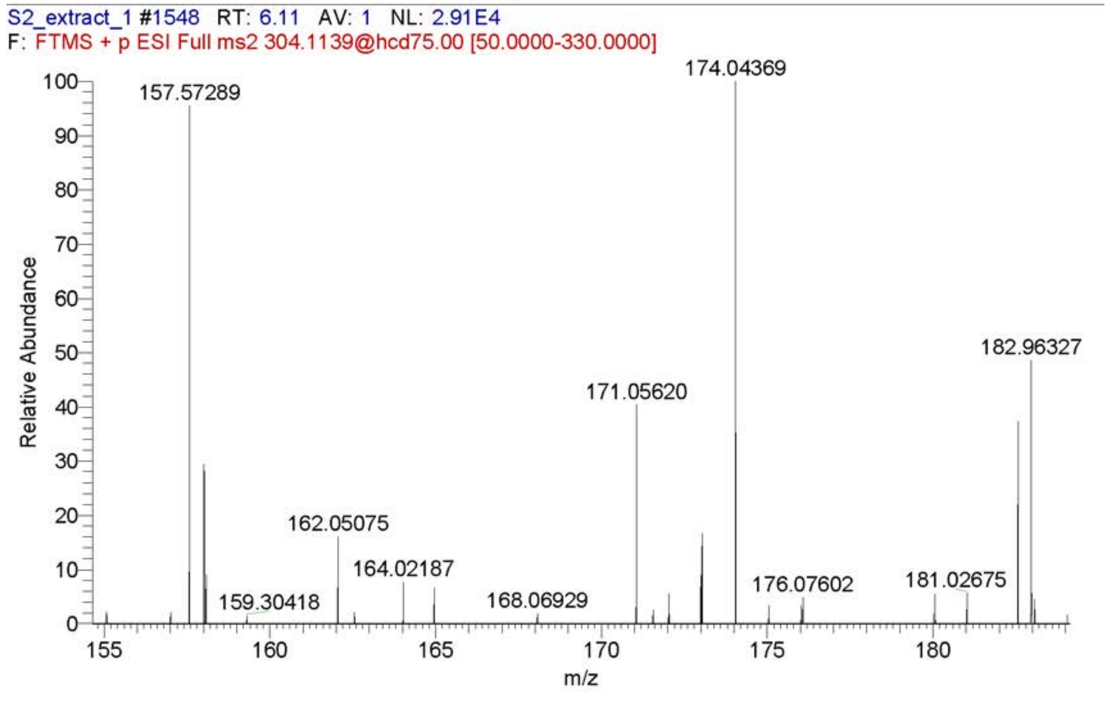

Stylochus sp 1.

11-norTTX-6-(*S*)-ol ***(3) m/z 290.0983***

This analyte was considered present when the mass elective scan showed the daughter ions of either 272.07 or 162.07 at similar retention times. These daughter ions were taken from Bane, Lehane, Dikshit, O'Riordan and Furey [1]. The samples that were reported to contain this analyte were *Stylochus sp. 1* (RT: 7.58), *Stylochus sp. 1* (RT: 7.58), *S. mcgrathi* (RT: 7.61), *S. mcgrathi* (RT: 7.55), *S. mcgrathi* (RT: 7.61), *S. mcgrathi* (RT: 7.58), *S. mcgrathi* (RT: 7.61), *S. mcgrathi* (RT: 7.55), *S. mcgrathi* (RT: 7.62), *S. mcgrathi* (RT: 7.61), *S. mcgrathi* (RT: 7.59), *E. celerrima* (RT: 7.56), *E. celerrima* (RT: 7.56), *E. celerrima* (RT: 7.58), *E. celerrima* (RT: 7.63), *N. longiducta* (RT: 7.56), *Pseudoceros sp. 1* (RT: 7.58), *Pseudoceros sp. 3* (RT: 7.55), *P. velutinus* (RT: 7.62), *P. velutinus* (RT: 7.53), *T. brocchii* (RT: 7.58), *T. brocchii* (RT: 7.61), *Cycloporus sp* (RT: 7.60), *and C. rubocincta* (RT: 7.49).

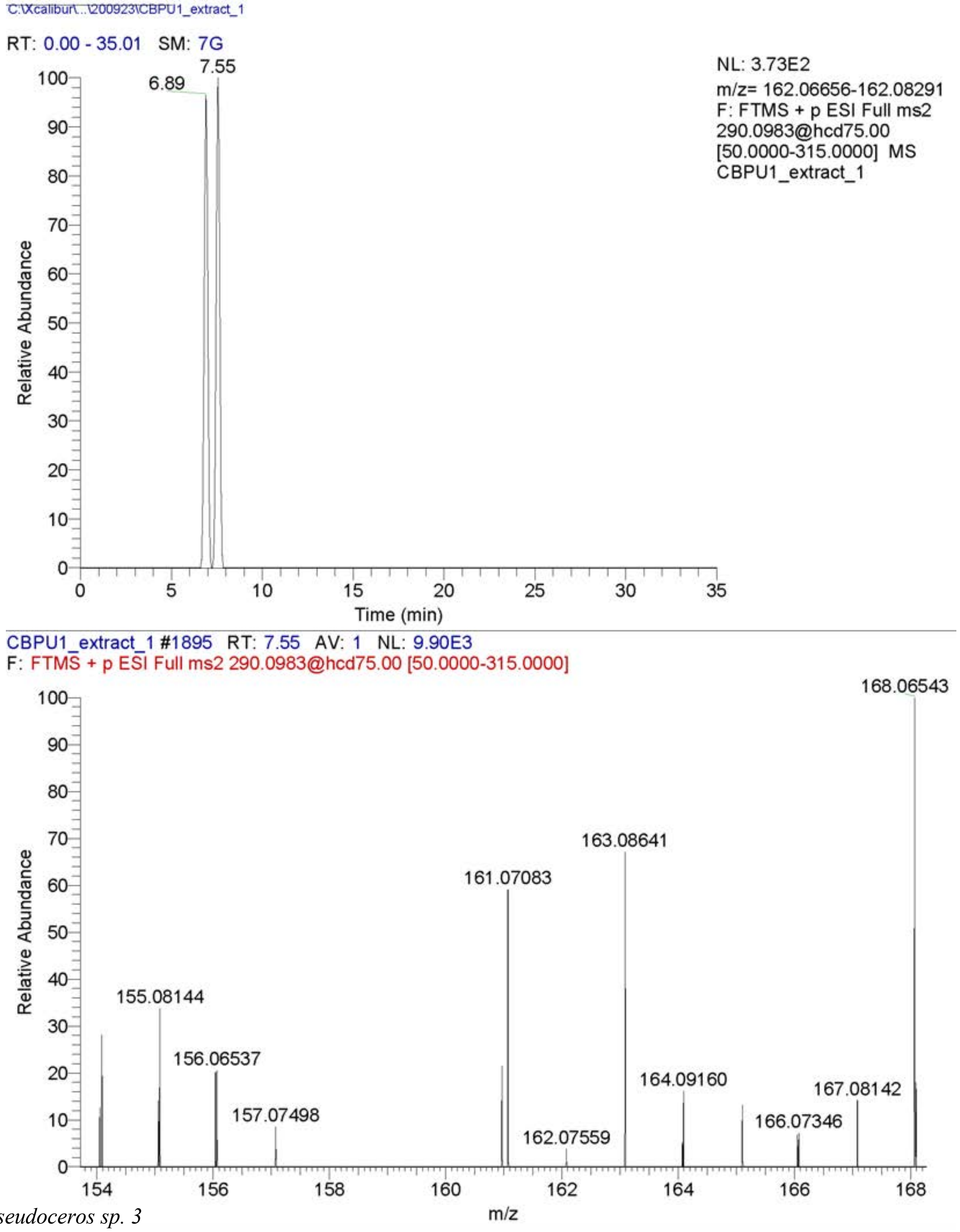

Pseudoceros sp. 3

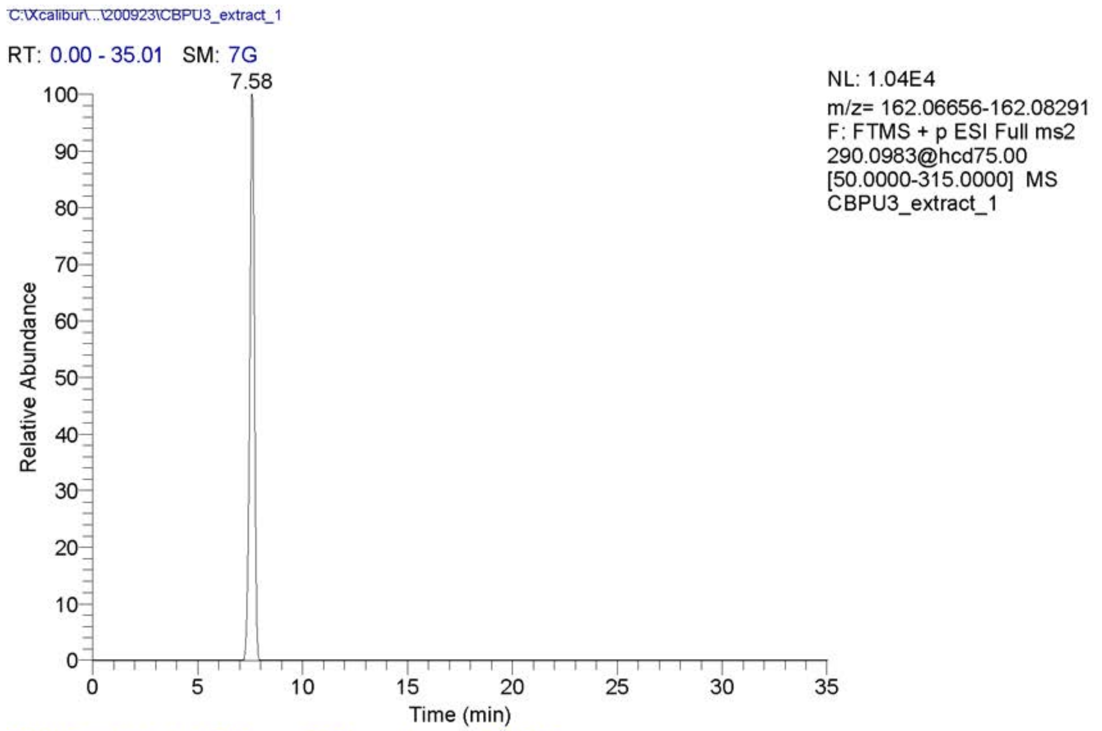

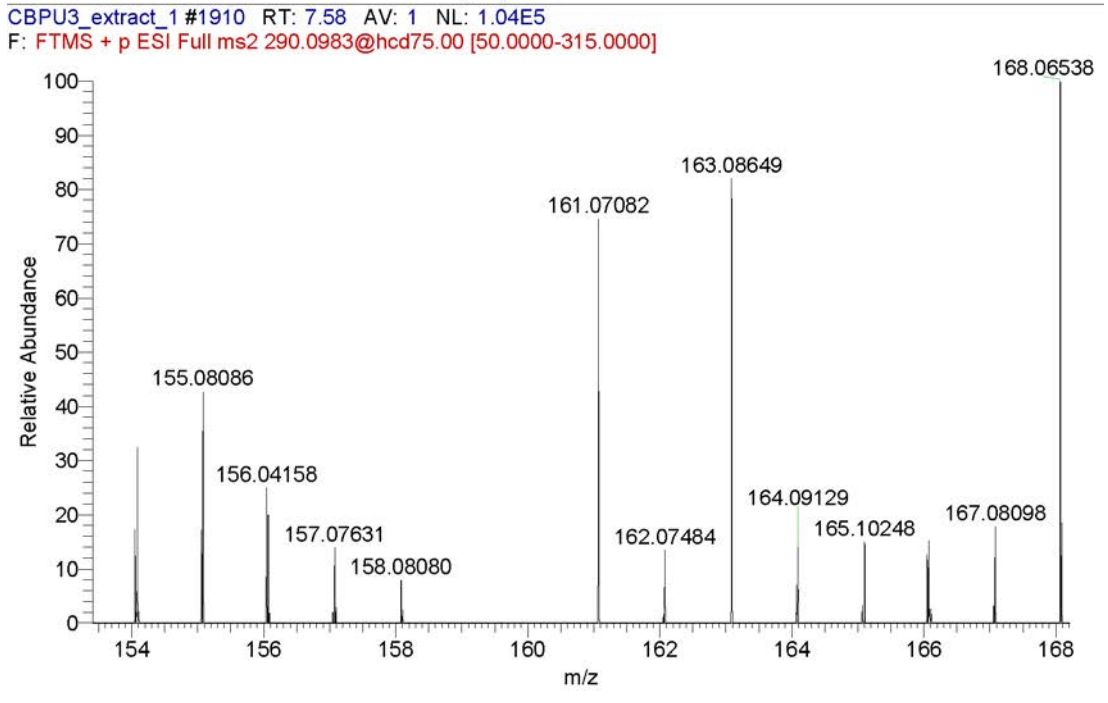

Pseudoceros sp. 1

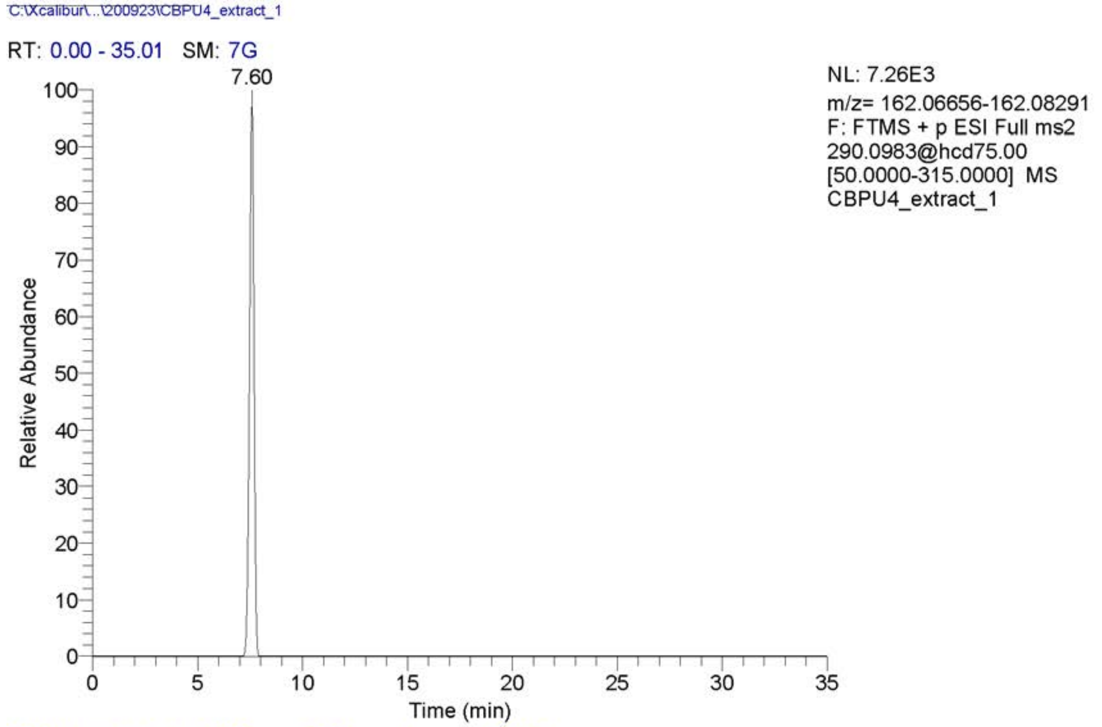

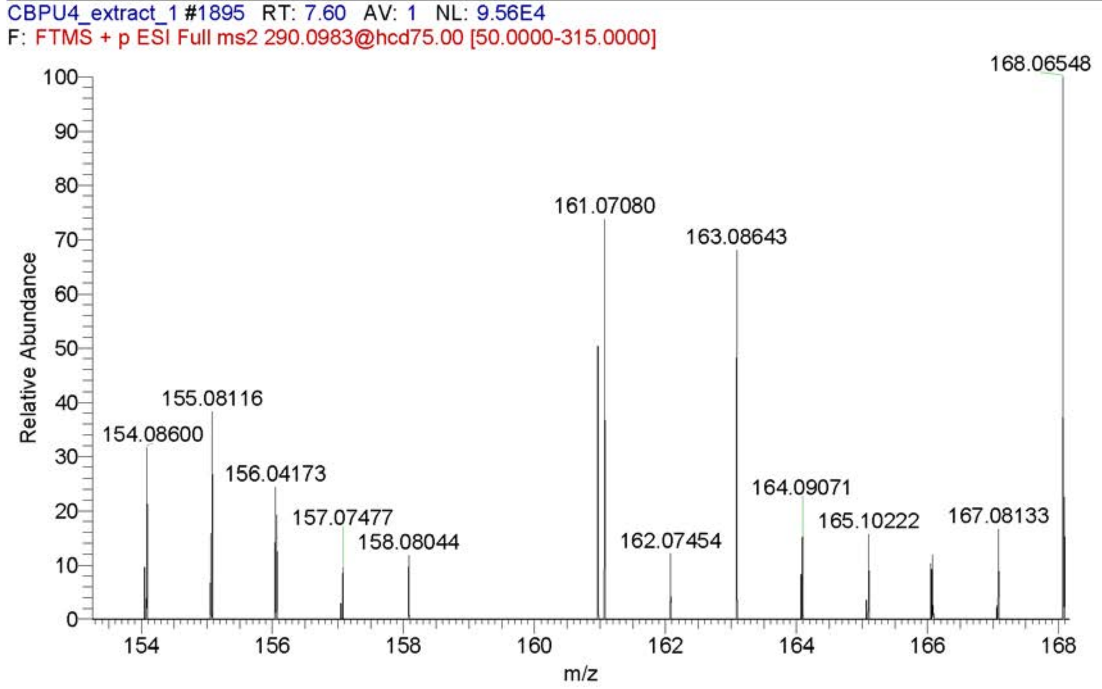

Cycloporus sp.

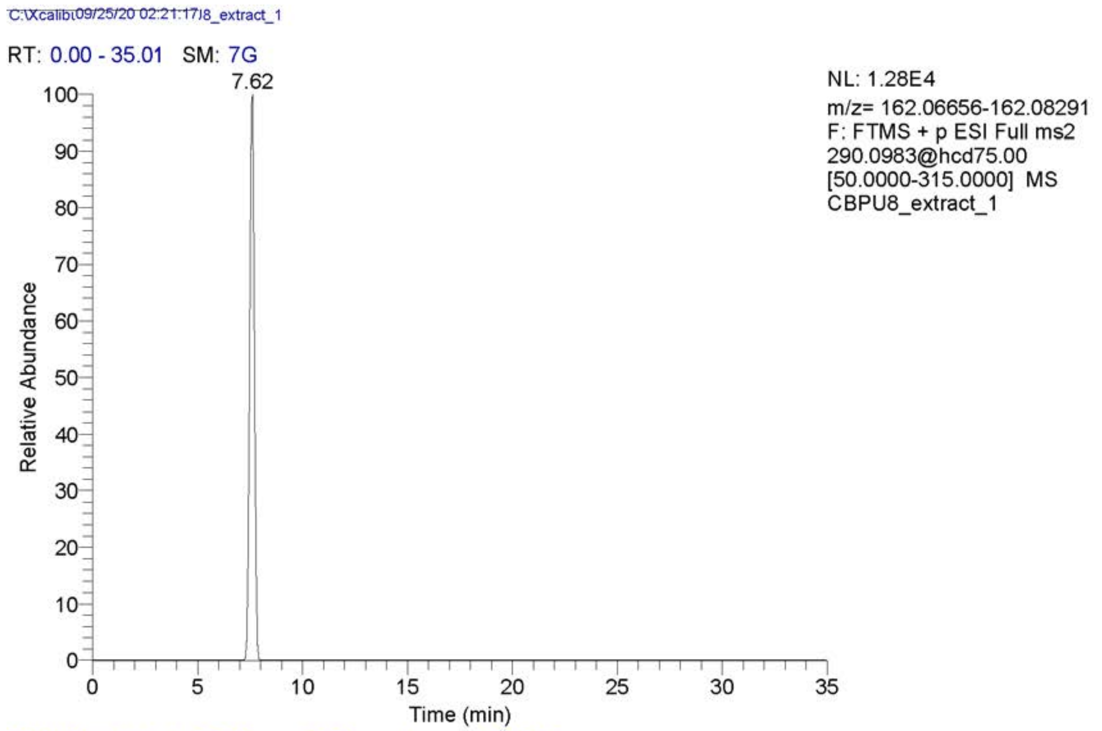
C:\Xcalib\09/25/20 02:21:17\8_extract_1
RT: 0.00 - 35.01 SM: 7G
7.62
NL: 1.28E4
m/z= 162.06656-162.08291
F: FTMS + p ESI Full ms2 290.0983@hcd75.00 [50.0000-315.0000] MS
CBPU8_extract_1
Relative Abundance
Time (min)

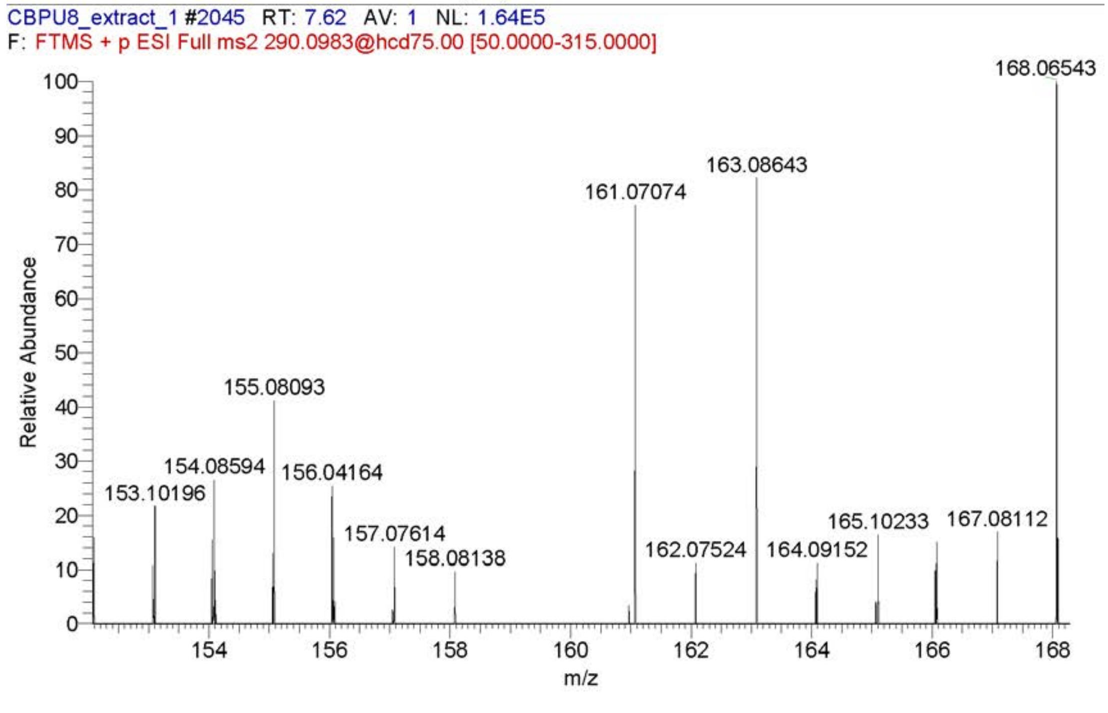
CBPU8_extract_1 #2045 RT: 7.62 AV: 1 NL: 1.64E5
F: FTMS + p ESI Full ms2 290.0983@hcd75.00 [50.0000-315.0000]
168.06543
163.08643
161.07074
155.08093
154.08594
153.10196
156.04164
157.07614
158.08138
162.07524
164.09152
165.10233
167.08112
Relative Abundance
m/z

P. velutinus

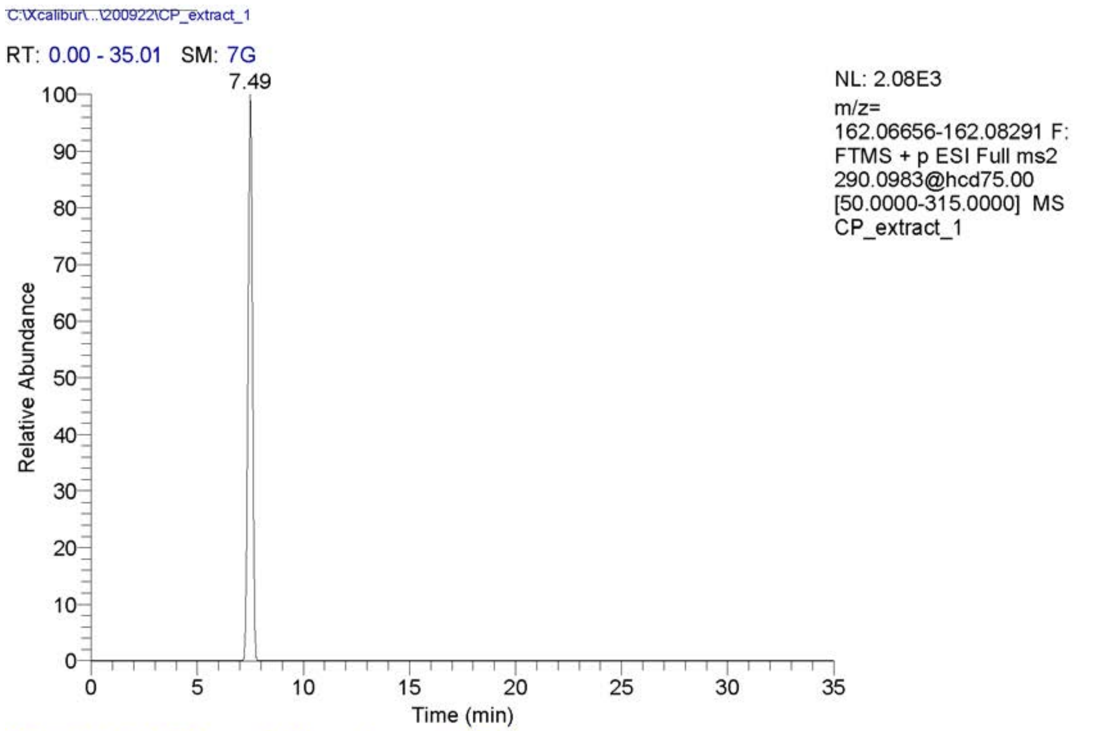

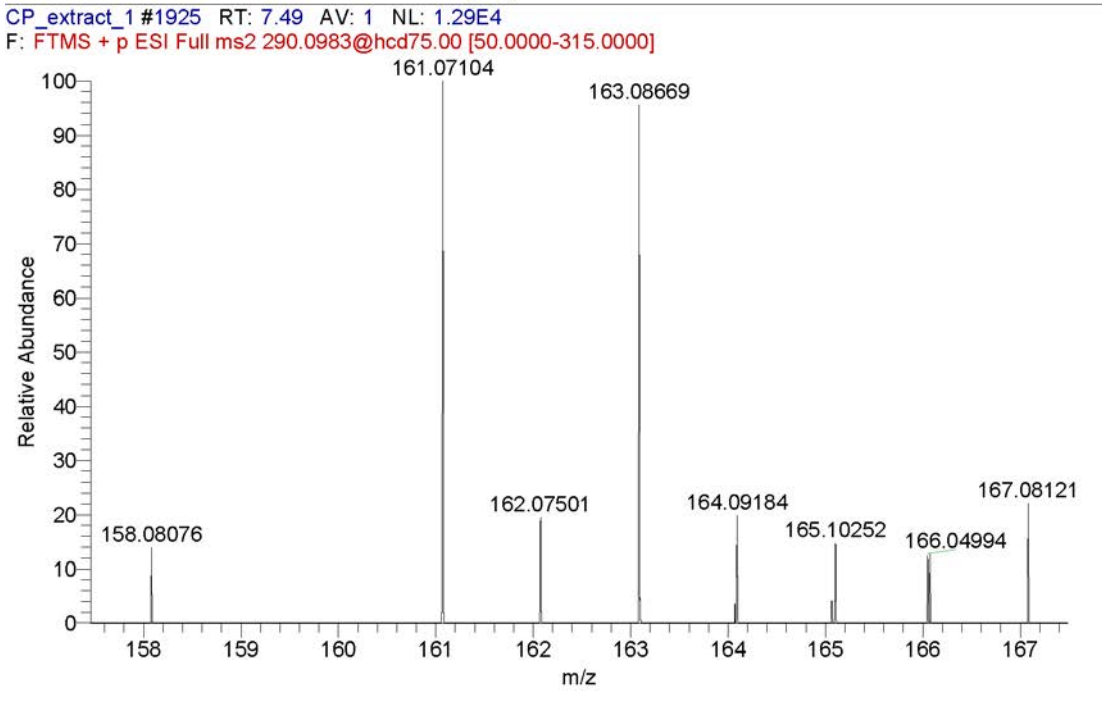

C. rubocincta

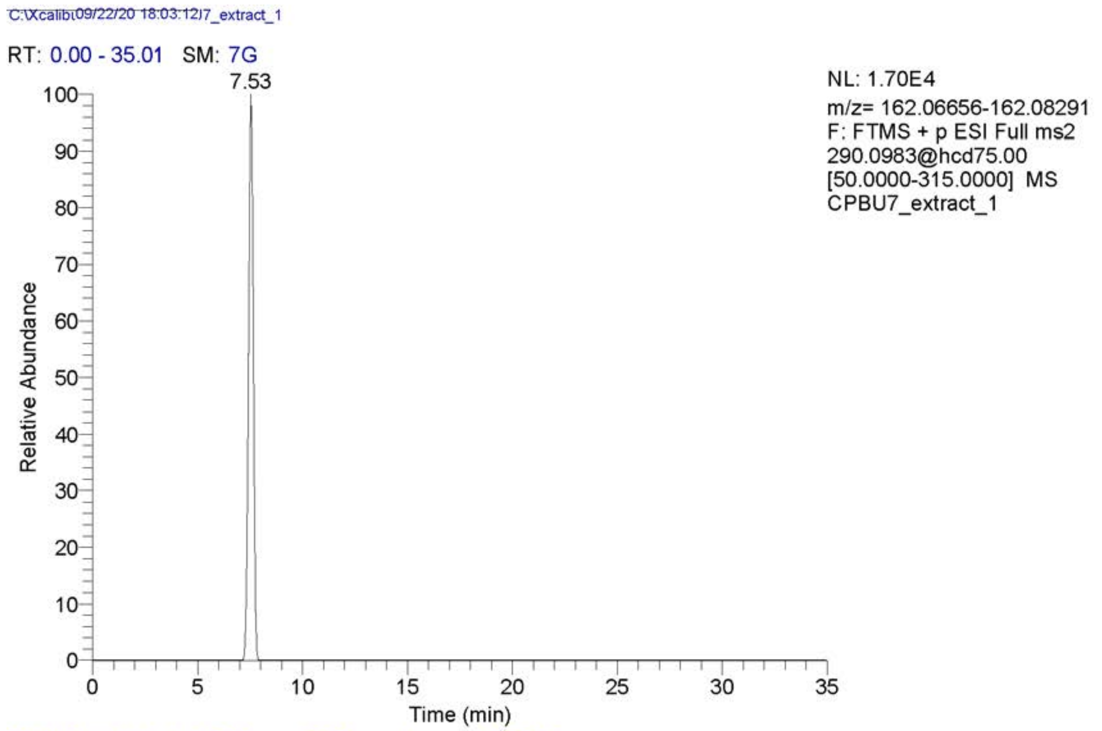
C:\Xcalibu09/22/20 18:03:12\7_extract_1
RT: 0.00 - 35.01 SM: 7G
7.53
NL: 1.70E4
m/z= 162.06656-162.08291
F: FTMS + p ESI Full ms2
290.0983@hcd75.00
[50.0000-315.0000] MS
CPBU7_extract_1
Relative Abundance
Time (min)

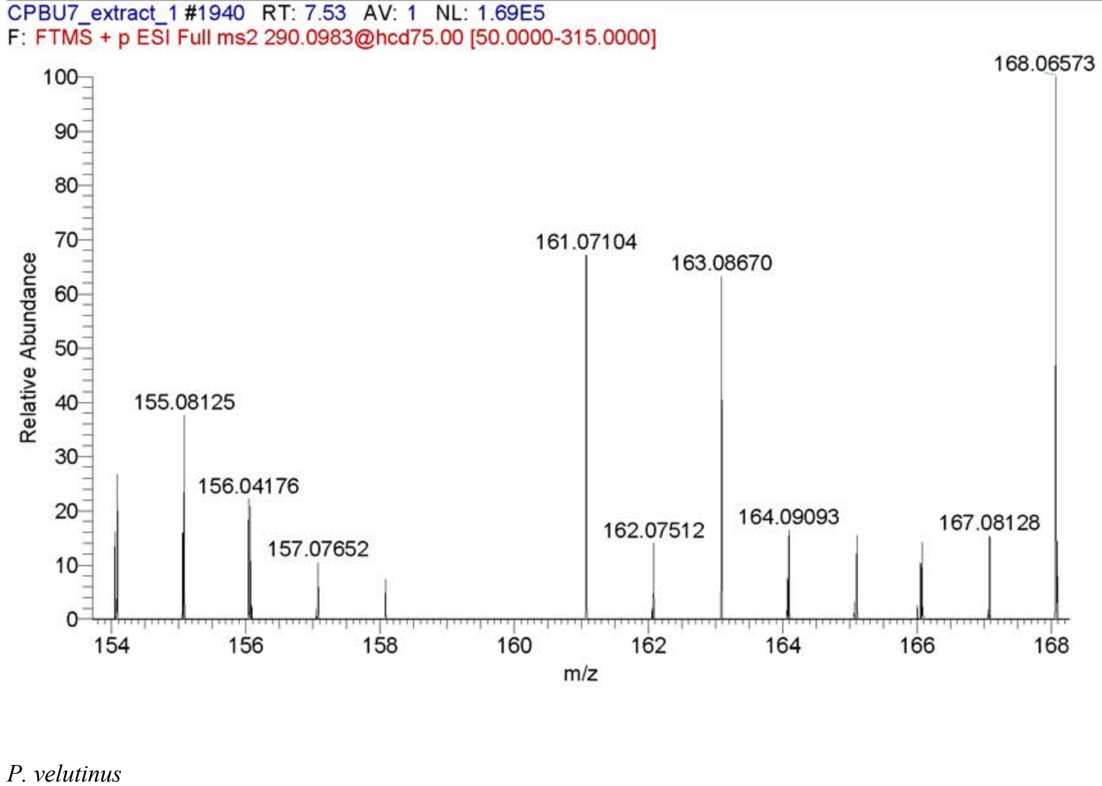
CPBU7_extract_1 #1940 RT: 7.53 AV: 1 NL: 1.69E5
F: FTMS + p ESI Full ms2 290.0983@hcd75.00 [50.0000-315.0000]
168.06573
161.07104
163.08670
155.08125
156.04176
157.07652
162.07512
164.09093
167.08128
Relative Abundance
m/z

P. velutinus

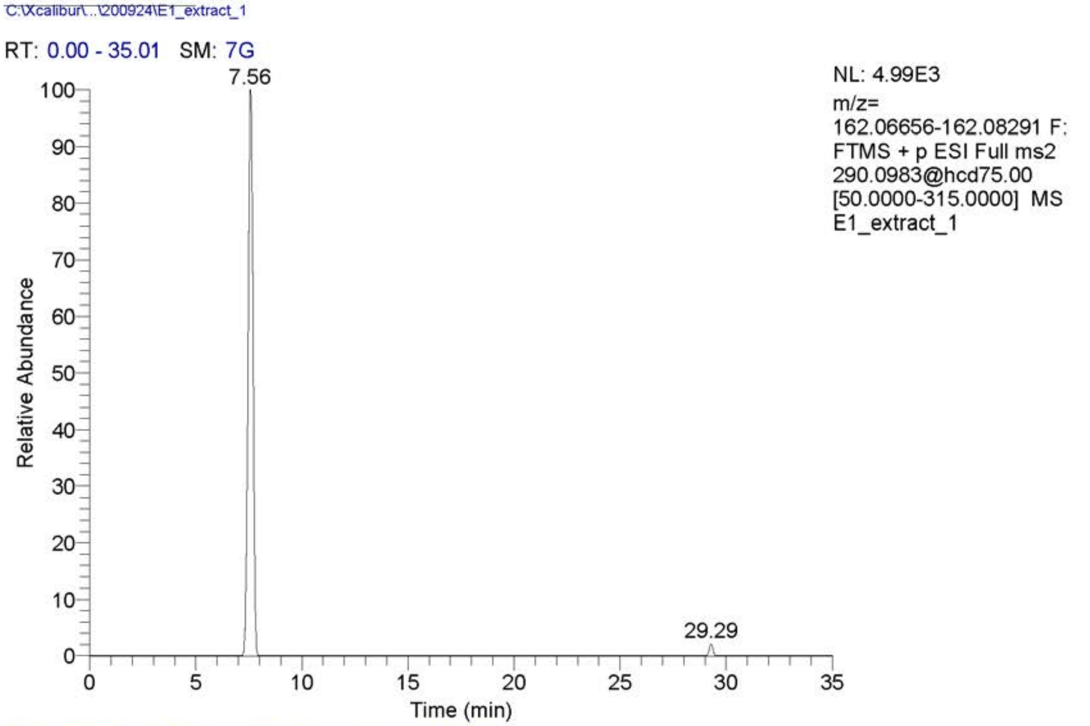

C:\Xcalibur\...\200924\E1_extract_1
RT: 0.00 - 35.01 SM: 7G
NL: 4.99E3
m/z=
162.06656-162.08291 F:
FTMS + p ESI Full ms2
290.0983@hcd75.00
[50.0000-315.0000] MS
E1_extract_1
7.56
29.29
Relative Abundance
Time (min)

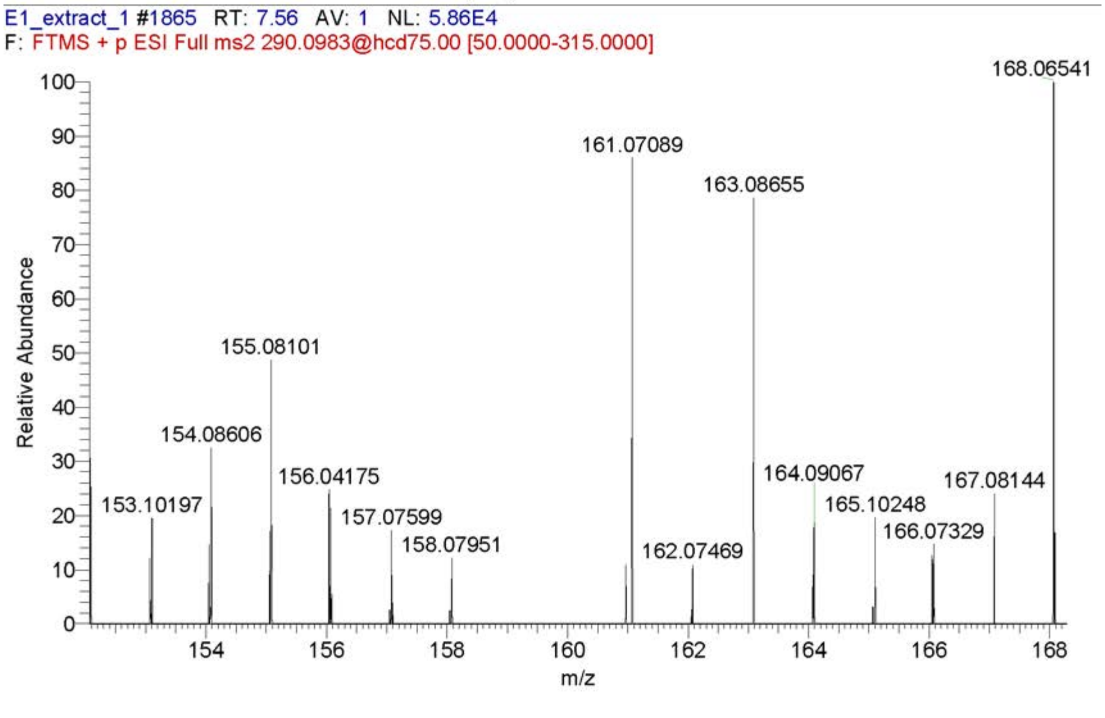

E1_extract_1 #1865 RT: 7.56 AV: 1 NL: 5.86E4
F: FTMS + p ESI Full ms2 290.0983@hcd75.00 [50.0000-315.0000]
168.06541
161.07089
163.08655
155.08101
154.08606
156.04175
153.10197
157.07599
158.07951
162.07469
164.09067
165.10248
166.07329
167.08144
Relative Abundance
m/z

E. celerrima

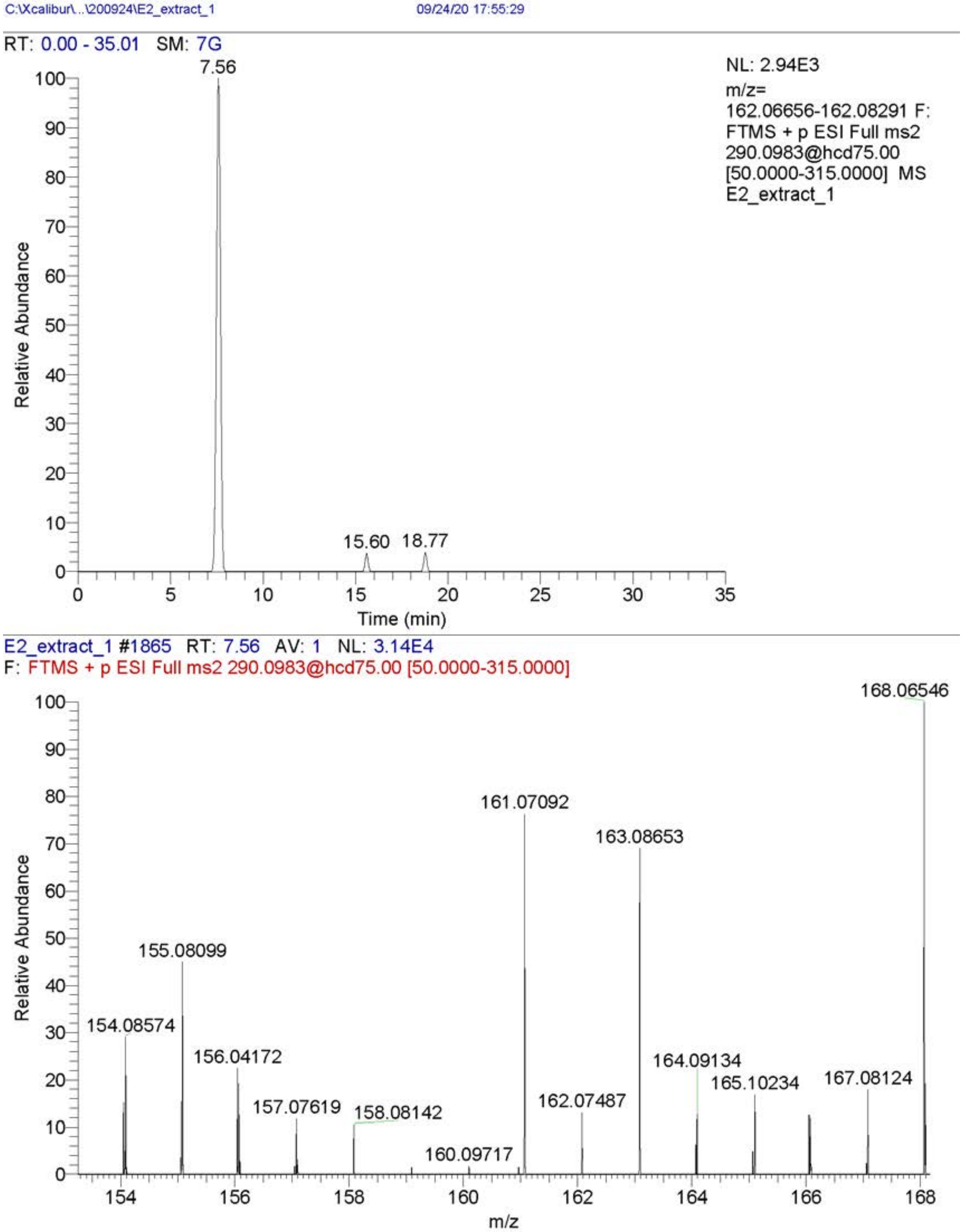
C:\Xcalibur\...\200924\E2_extract_1
09/24/20 17:55:29
RT: 0.00 - 35.01 SM: 7G
NL: 2.94E3
m/z=
162.06656-162.08291 F:
FTMS + p ESI Full ms2
290.0983@hcd75.00
[50.0000-315.0000] MS
E2_extract_1
7.56
15.60
18.77
Relative Abundance
Time (min)
E2_extract_1 #1865 RT: 7.56 AV: 1 NL: 3.14E4
F: FTMS + p ESI Full ms2 290.0983@hcd75.00 [50.0000-315.0000]
168.06546
161.07092
163.08653
155.08099
154.08574
156.04172
157.07619
158.08142
160.09717
162.07487
164.09134
165.10234
167.08124
m/z

E. celerrima

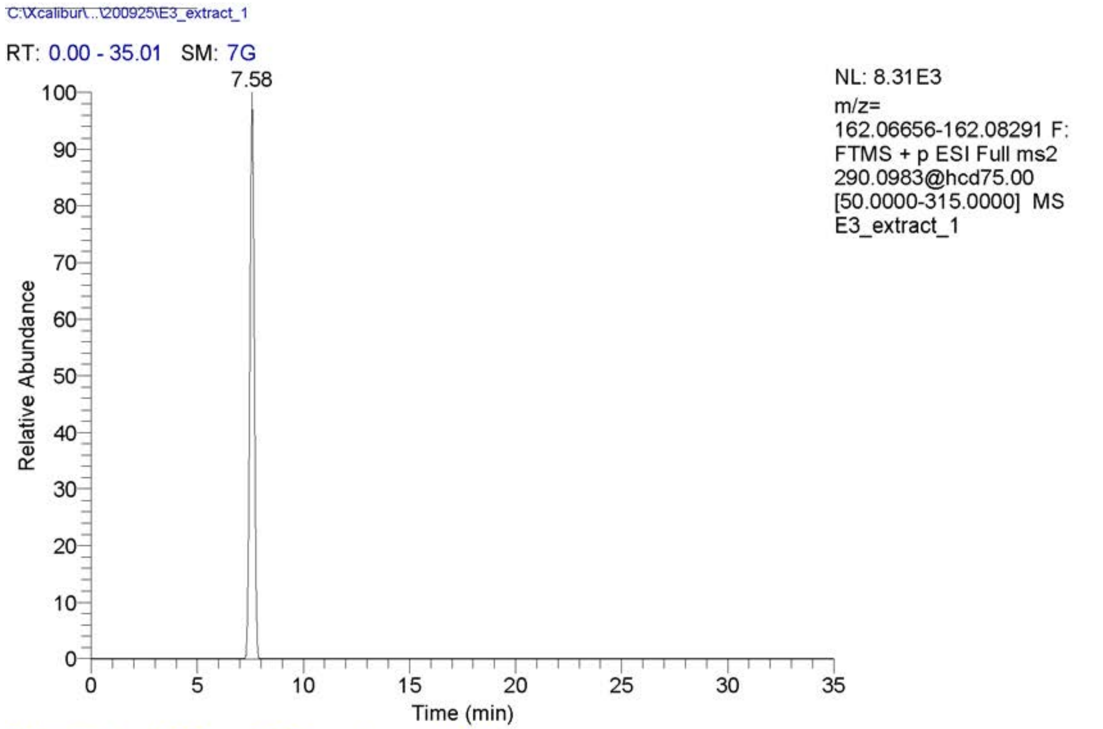
C:\Xcalibur\...\200925\E3_extract_1
RT: 0.00 - 35.01 SM: 7G
7.58
NL: 8.31E3
m/z=
162.06656-162.08291 F:
FTMS + p ESI Full ms2
290.0983@hcd75.00
[50.0000-315.0000] MS
E3_extract_1
Relative Abundance
Time (min)

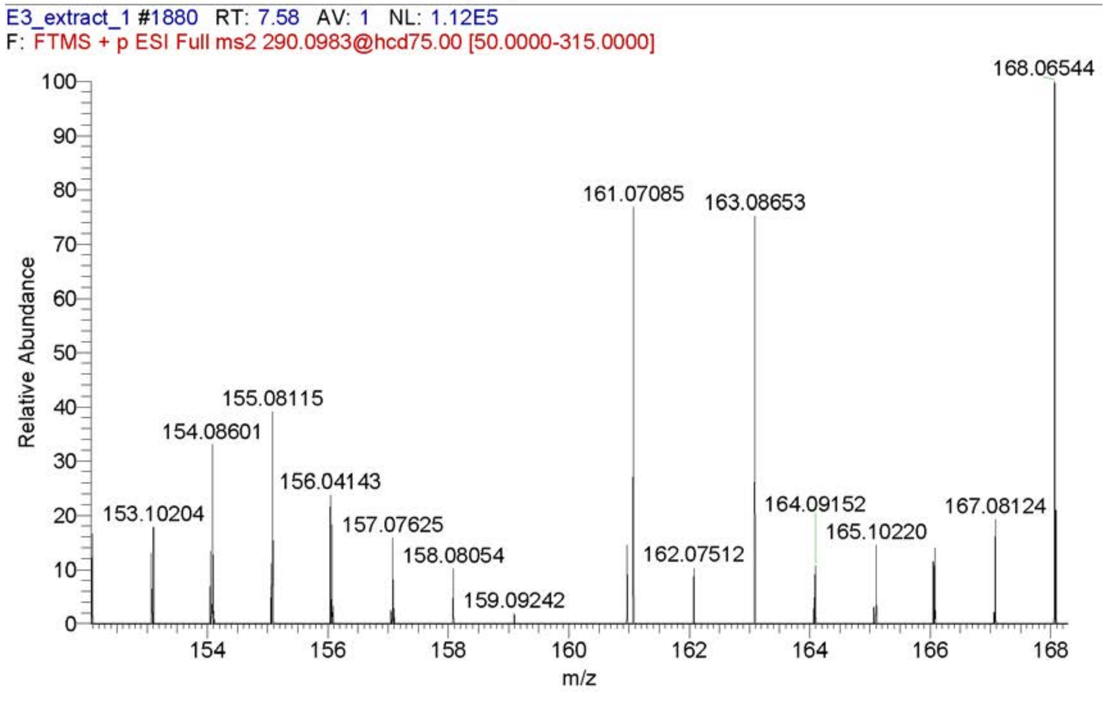
E3_extract_1 #1880 RT: 7.58 AV: 1 NL: 1.12E5
F: FTMS + p ESI Full ms2 290.0983@hcd75.00 [50.0000-315.0000]
168.06544
161.07085
163.08653
155.08115
154.08601
156.04143
153.10204
157.07625
158.08054
159.09242
162.07512
164.09152
165.10220
167.08124
Relative Abundance
m/z

E. celerrima

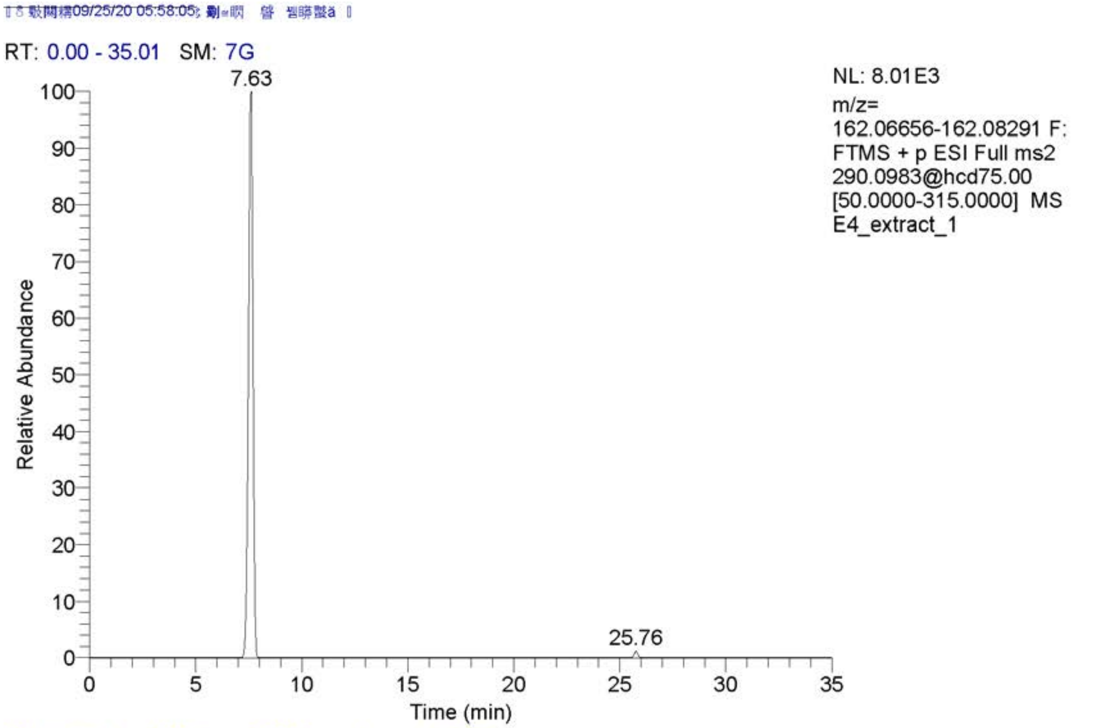
RT: 0.00 - 35.01 SM: 7G
NL: 8.01E3
m/z=
162.06656-162.08291 F:
FTMS + p ESI Full ms2
290.0983@hcd75.00
[50.0000-315.0000] MS
E4_extract_1
7.63
25.76
Relative Abundance
Time (min)

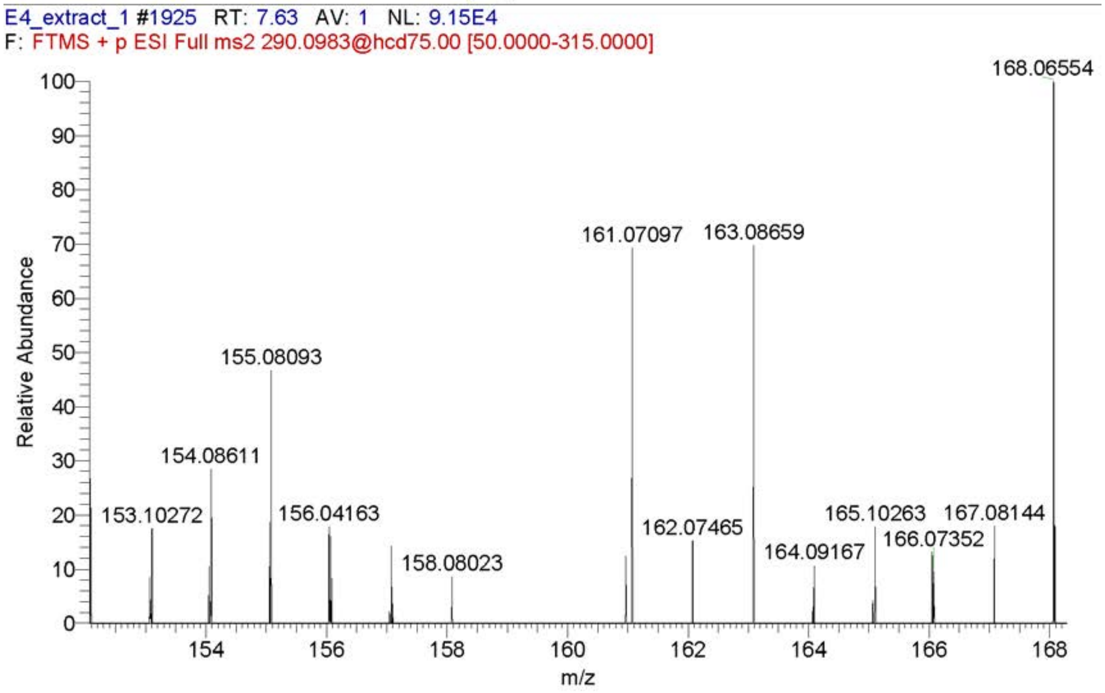
E4_extract_1 #1925 RT: 7.63 AV: 1 NL: 9.15E4
F: FTMS + p ESI Full ms2 290.0983@hcd75.00 [50.0000-315.0000]
168.06554
161.07097
163.08659
155.08093
154.08611
153.10272
156.04163
158.08023
162.07465
164.09167
165.10263
166.07352
167.08144
Relative Abundance
m/z

E. celerrima

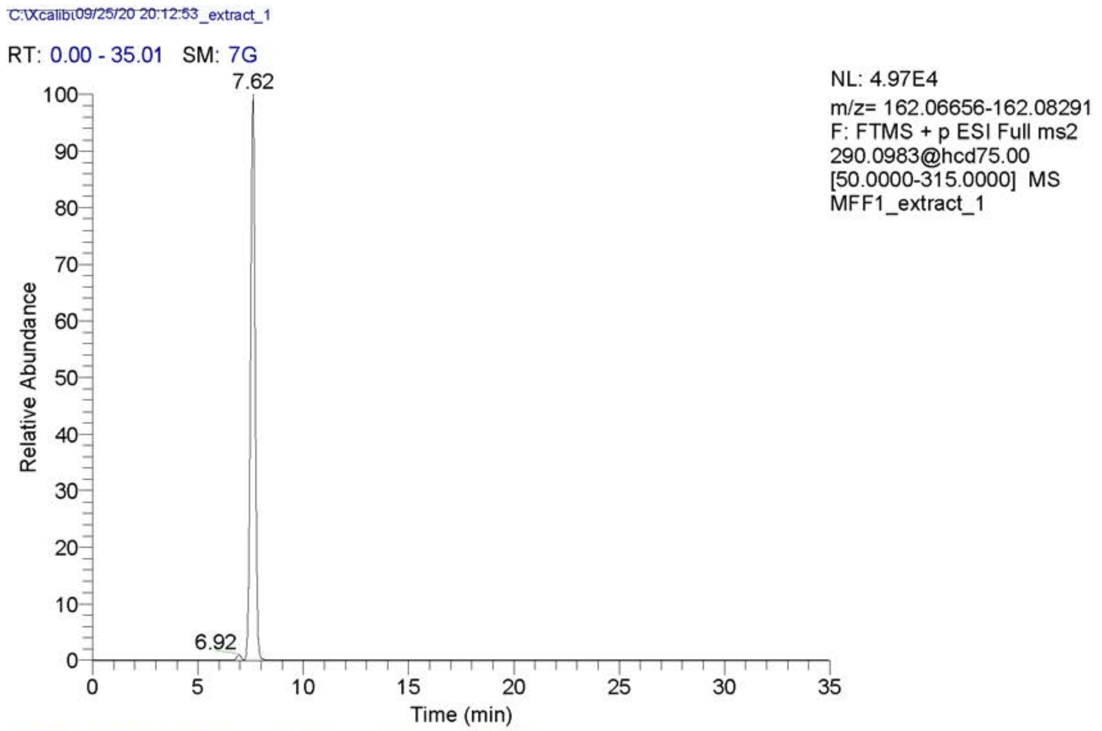
C:\Xcalibur\09/25/20 20:12:53 _extract_1
RT: 0.00 - 35.01 SM: 7G
NL: 4.97E4
m/z= 162.06656-162.08291
F: FTMS + p ESI Full ms2 290.0983@hcd75.00 [50.0000-315.0000] MS
MFF1_extract_1
7.62
6.92
Relative Abundance
Time (min)

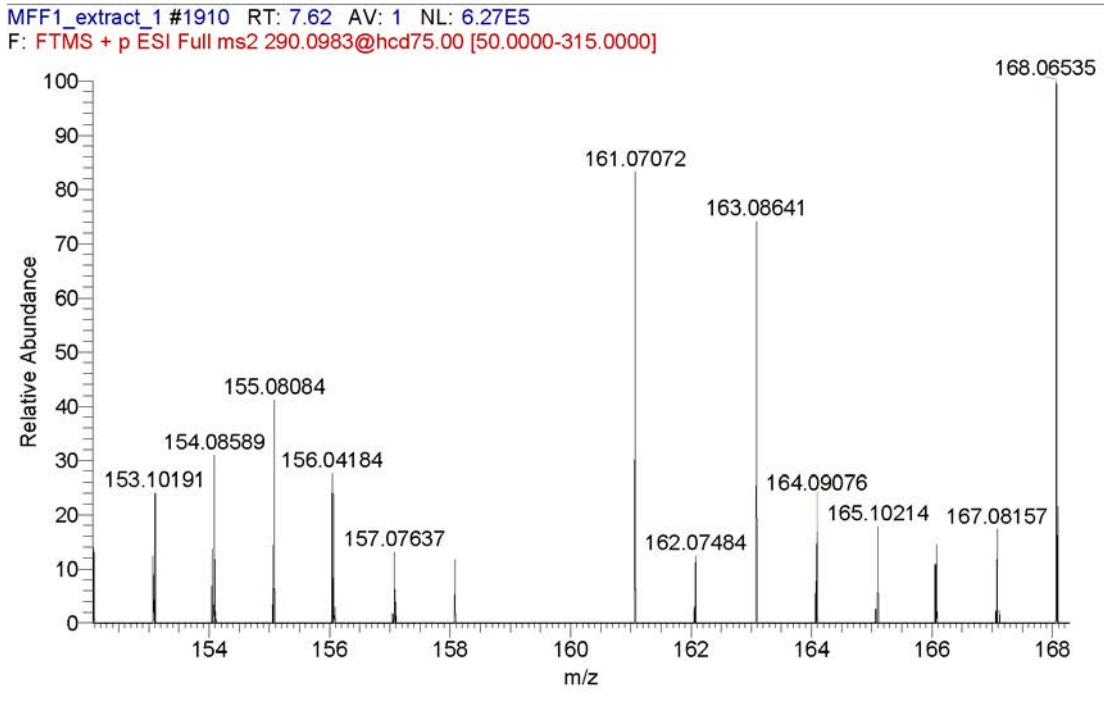
MFF1_extract_1 #1910 RT: 7.62 AV: 1 NL: 6.27E5
F: FTMS + p ESI Full ms2 290.0983@hcd75.00 [50.0000-315.0000]
168.06535
161.07072
163.08641
155.08084
154.08589
156.04184
153.10191
164.09076
165.10214
167.08157
157.07637
162.07484
Relative Abundance
m/z

S. mcgrathi

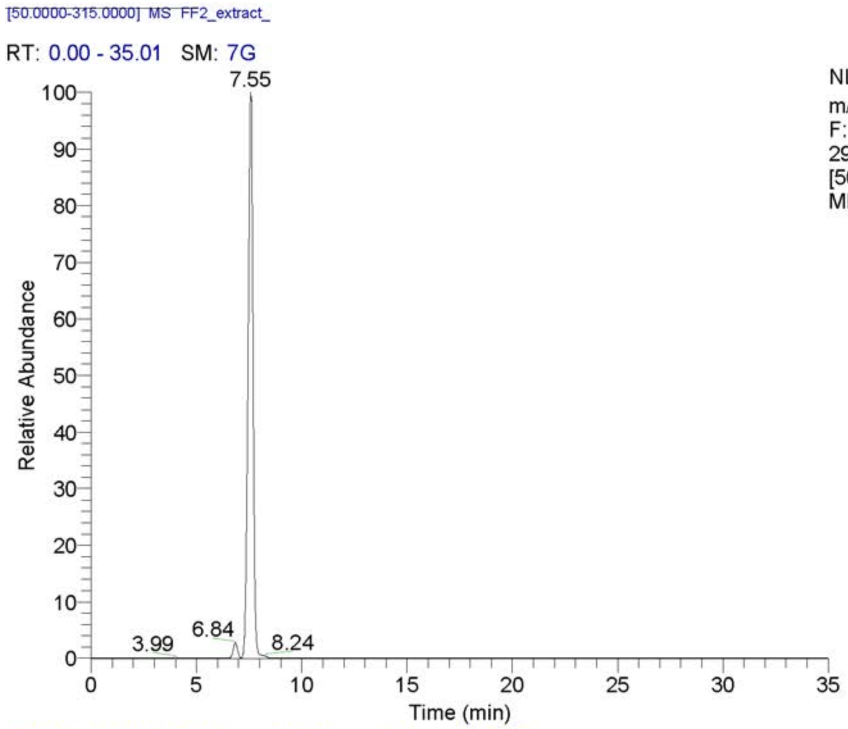
[50.0000-315.0000] MS FF2_extract_
RT: 0.00 - 35.01 SM: 7G
NL: 1.68E5
m/z= 162.06656-162.08291
F: FTMS + p ESI Full ms2 290.0983@hcd75.00 [50.0000-315.0000] MS MFF2_extract_1
7.55
3.99
6.84
8.24
Relative Abundance
Time (min)

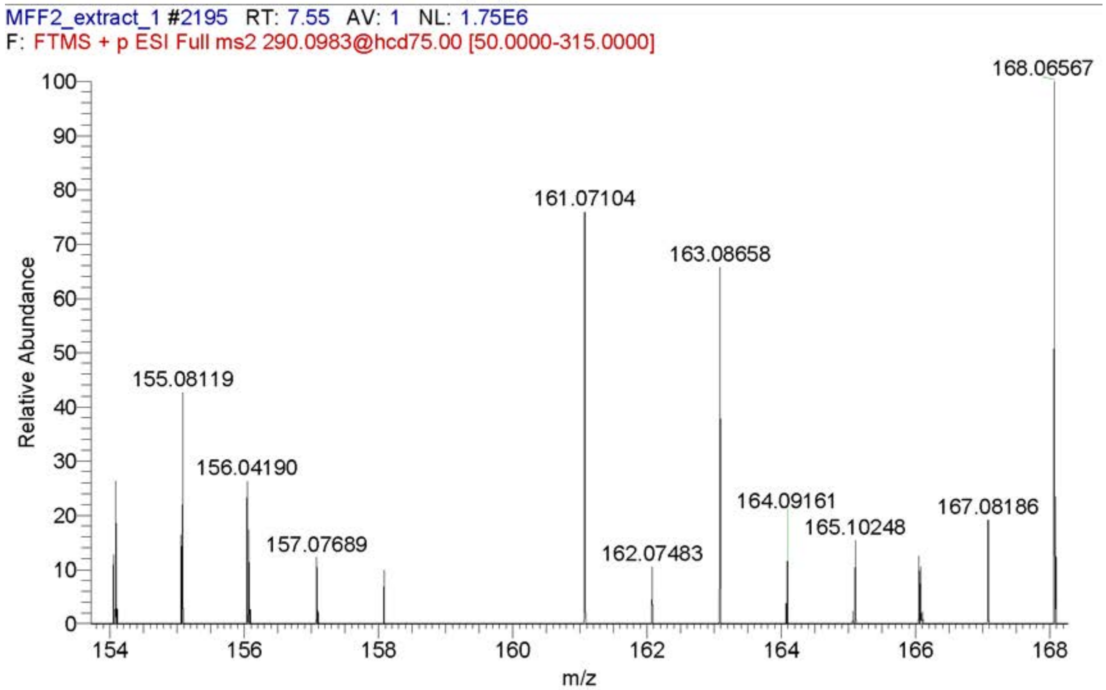
MFF2_extract_1 #2195 RT: 7.55 AV: 1 NL: 1.75E6
F: FTMS + p ESI Full ms2 290.0983@hcd75.00 [50.0000-315.0000]
168.06567
161.07104
163.08658
155.08119
156.04190
164.09161
165.10248
167.08186
157.07689
162.07483
Relative Abundance
m/z

S. mcgrathi

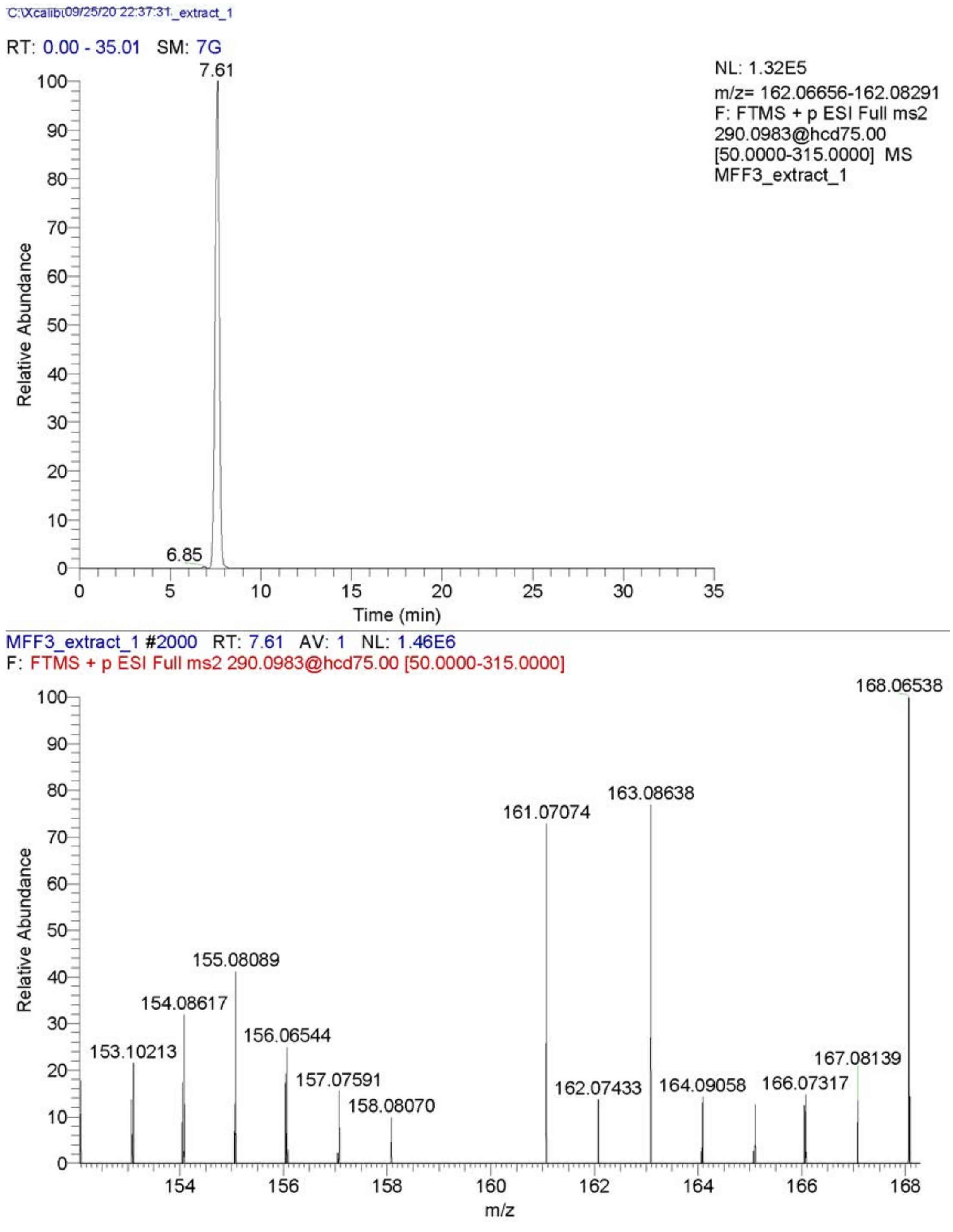
C:\Xcalibu09/25/20 22:37:31._extract_1
RT: 0.00 - 35.01 SM: 7G
NL: 1.32E5
m/z= 162.06656-162.08291
F: FTMS + p ESI Full ms2 290.0983@hcd75.00 [50.0000-315.0000] MS
MFF3_extract_1
7.61
6.85
Relative Abundance
Time (min)
MFF3_extract_1 #2000 RT: 7.61 AV: 1 NL: 1.46E6
F: FTMS + p ESI Full ms2 290.0983@hcd75.00 [50.0000-315.0000]
168.06538
163.08638
161.07074
155.08089
154.08617
156.06544
153.10213
167.08139
157.07591
162.07433
164.09058
166.07317
158.08070
m/z

S. mcgrathi

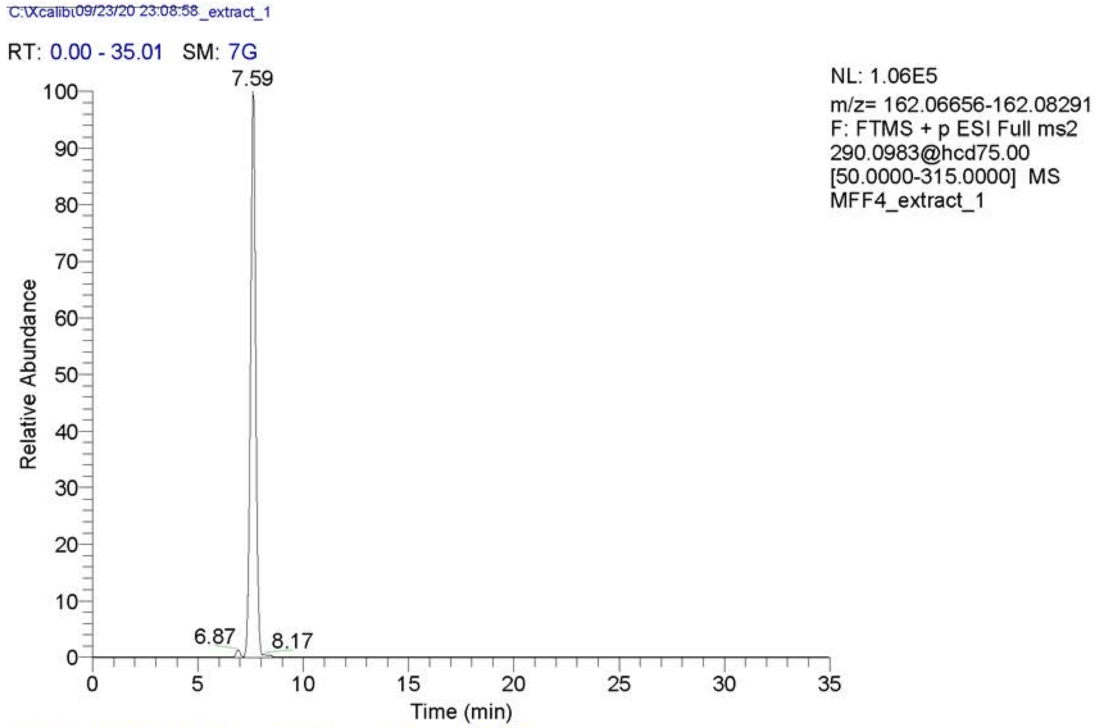
C:\Xcalibı09/23/20 23:08:58_extract_1
RT: 0.00 - 35.01 SM: 7G
NL: 1.06E5
m/z= 162.06656-162.08291
F: FTMS + p ESI Full ms2 290.0983@hcd75.00 [50.0000-315.0000] MS
MFF4_extract_1
7.59
6.87
8.17
Relative Abundance
Time (min)

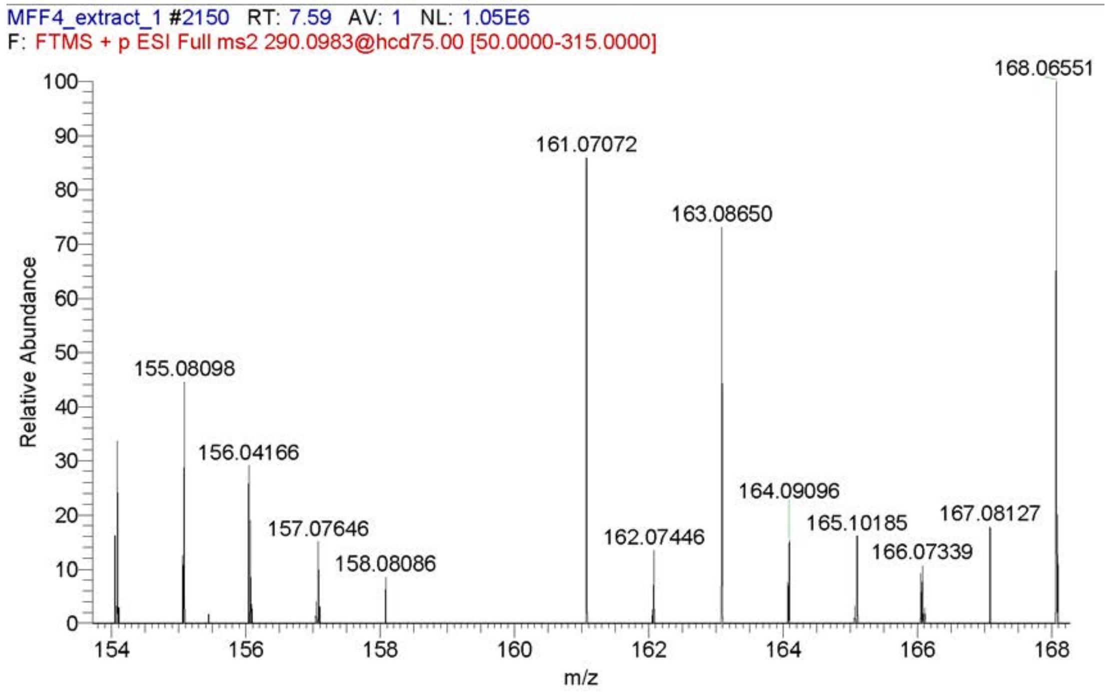
MFF4_extract_1 #2150 RT: 7.59 AV: 1 NL: 1.05E6
F: FTMS + p ESI Full ms2 290.0983@hcd75.00 [50.0000-315.0000]
168.06551
161.07072
163.08650
155.08098
156.04166
164.09096
165.10185
167.08127
157.07646
162.07446
166.07339
158.08086
Relative Abundance
m/z

S. mcgrathi

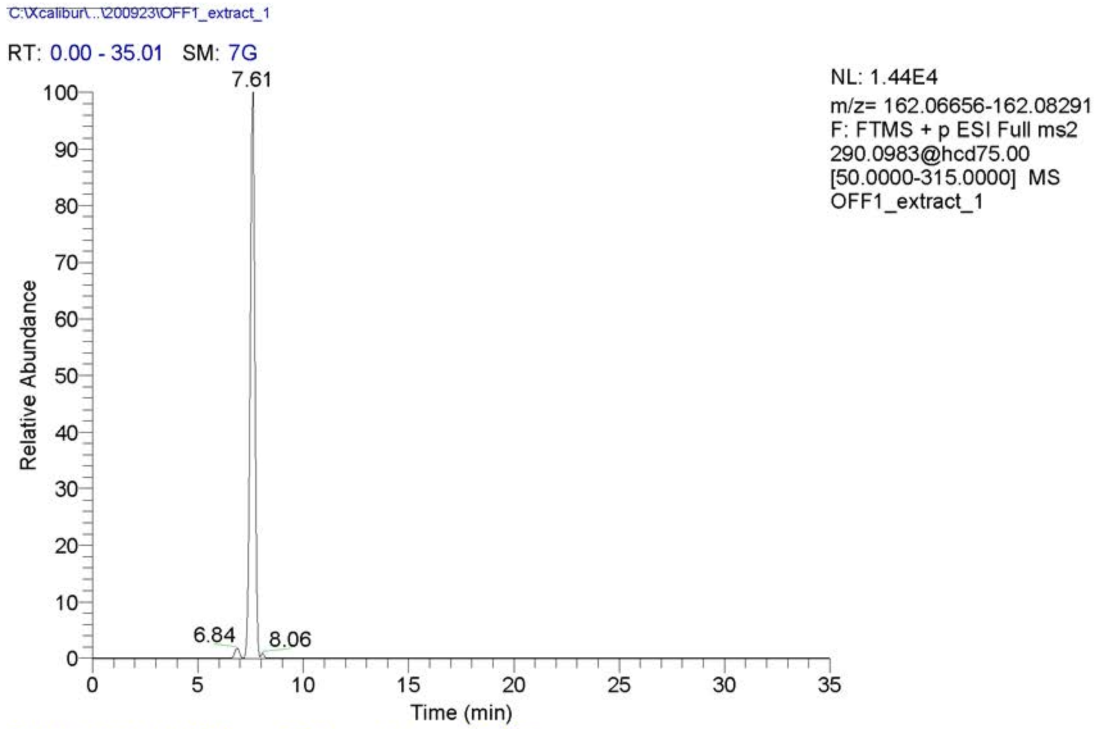
C:\Xcalibur\...\200923\OFF1_extract_1
RT: 0.00 - 35.01 SM: 7G
NL: 1.44E4
m/z= 162.06656-162.08291
F: FTMS + p ESI Full ms2 290.0983@hcd75.00 [50.0000-315.0000] MS OFF1_extract_1
7.61
6.84
8.06
Relative Abundance
Time (min)

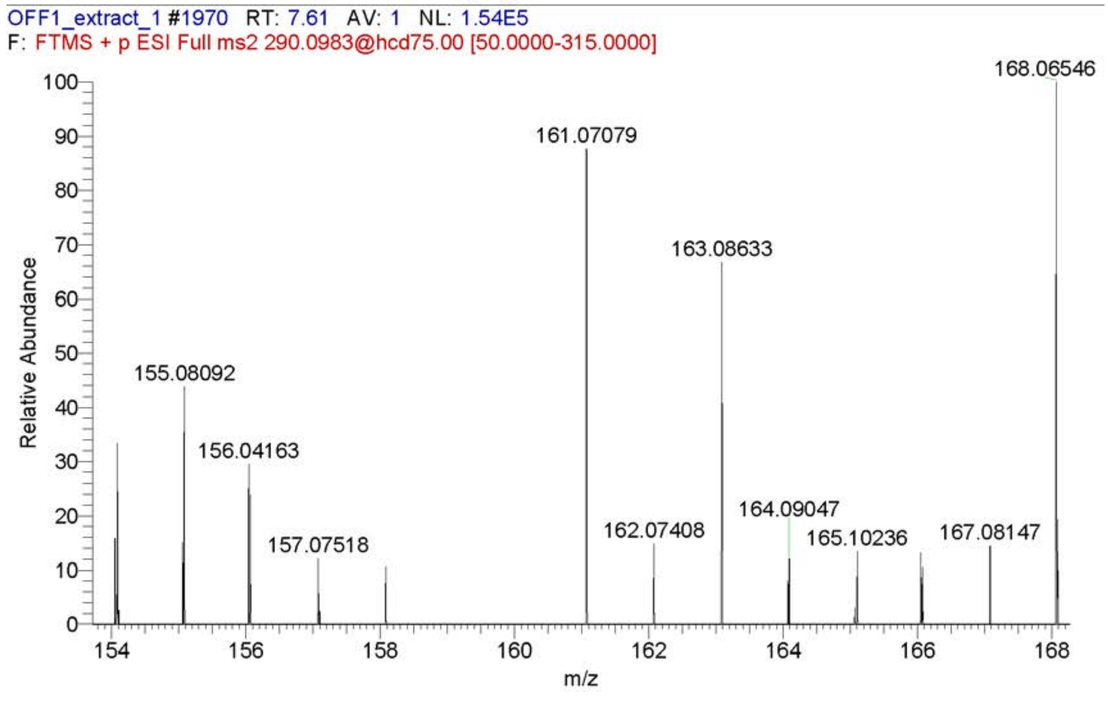
OFF1_extract_1 #1970 RT: 7.61 AV: 1 NL: 1.54E5
F: FTMS + p ESI Full ms2 290.0983@hcd75.00 [50.0000-315.0000]
168.06546
161.07079
163.08633
155.08092
156.04163
157.07518
162.07408
164.09047
165.10236
167.08147
Relative Abundance
m/z

S. mcgrathi

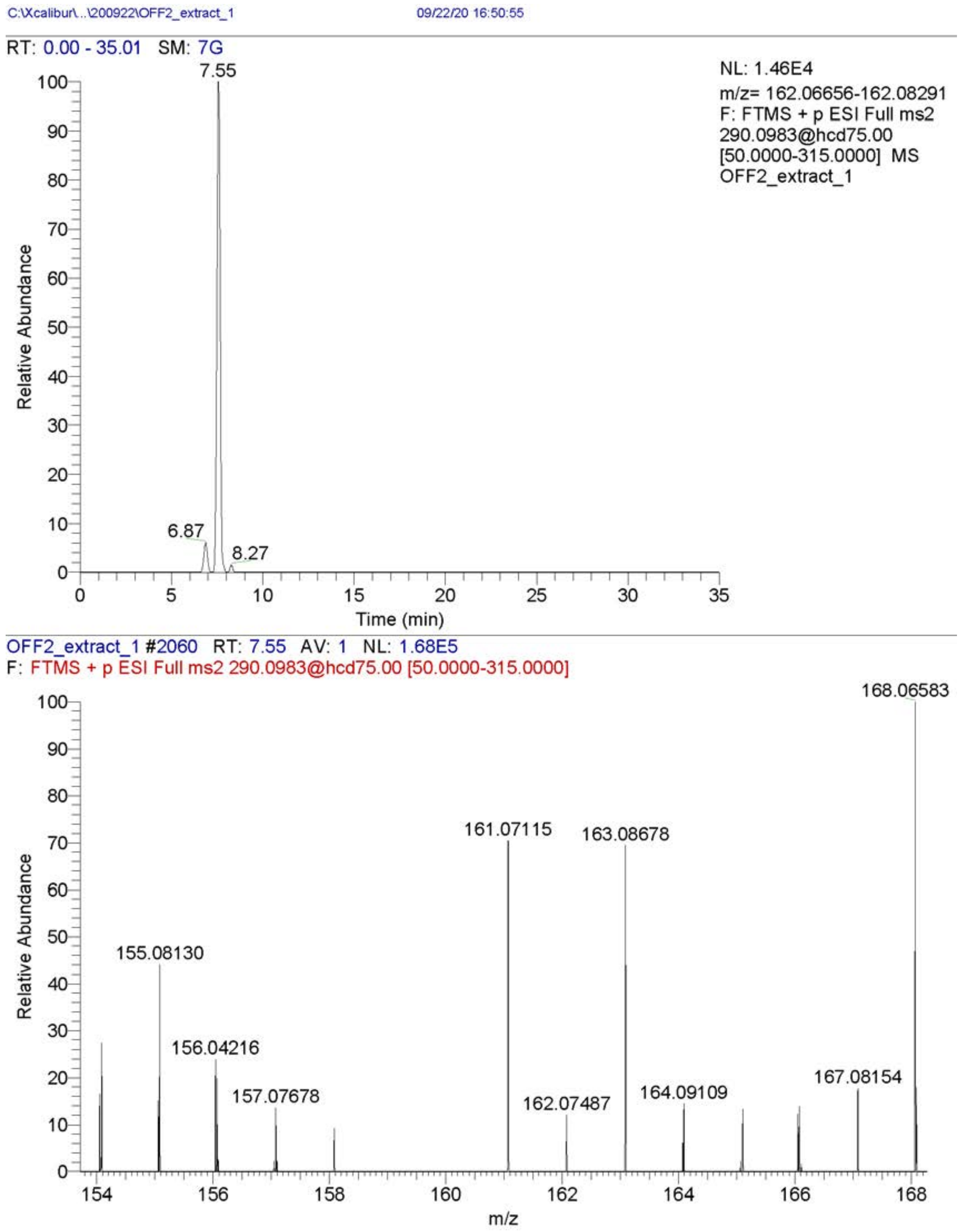
C:\Xcalibur\...\200922\OFF2_extract_1
09/22/20 16:50:55
RT: 0.00 - 35.01 SM: 7G
NL: 1.46E4
m/z= 162.06656-162.08291
F: FTMS + p ESI Full ms2
290.0983@hcd75.00
[50.0000-315.0000] MS
OFF2_extract_1
7.55
6.87
8.27
Relative Abundance
Time (min)
OFF2_extract_1 #2060 RT: 7.55 AV: 1 NL: 1.68E5
F: FTMS + p ESI Full ms2 290.0983@hcd75.00 [50.0000-315.0000]
168.06583
161.07115
163.08678
155.08130
156.04216
157.07678
162.07487
164.09109
167.08154
Relative Abundance
m/z

S. mcgrathi

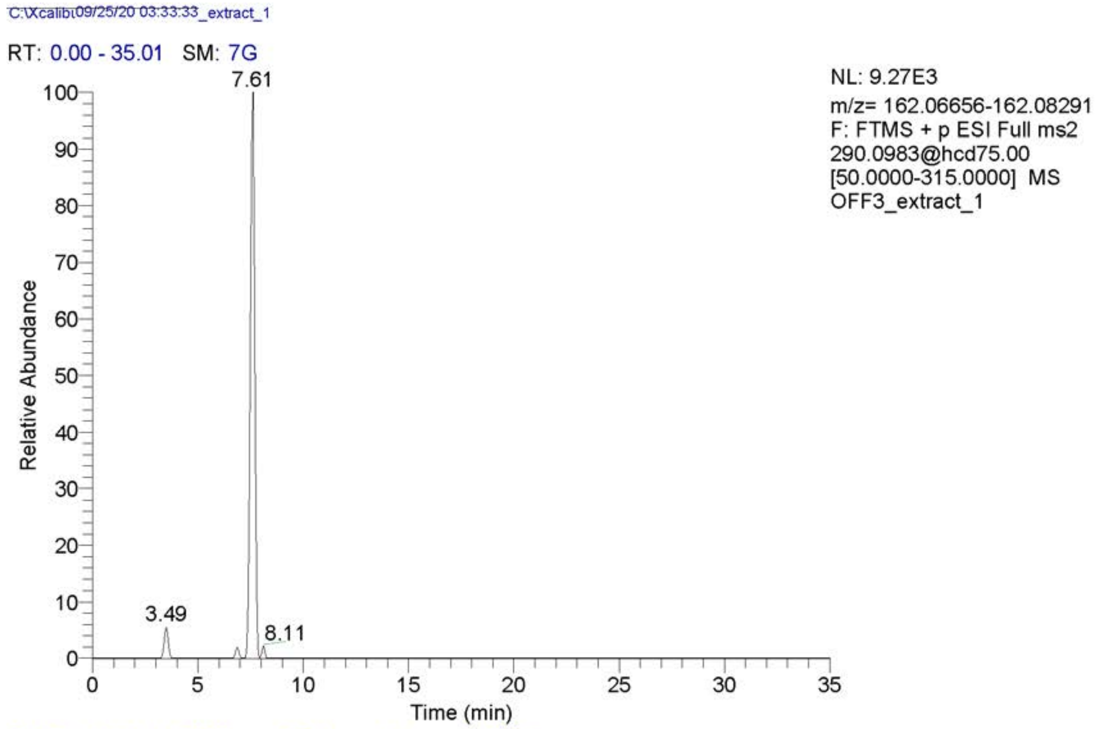
C:\Xcalibι09/25/20 03:33:33_extract_1
RT: 0.00 - 35.01 SM: 7G
NL: 9.27E3
m/z= 162.06656-162.08291
F: FTMS + p ESI Full ms2
290.0983@hcd75.00
[50.0000-315.0000] MS
OFF3_extract_1
7.61
3.49
8.11
Relative Abundance
Time (min)

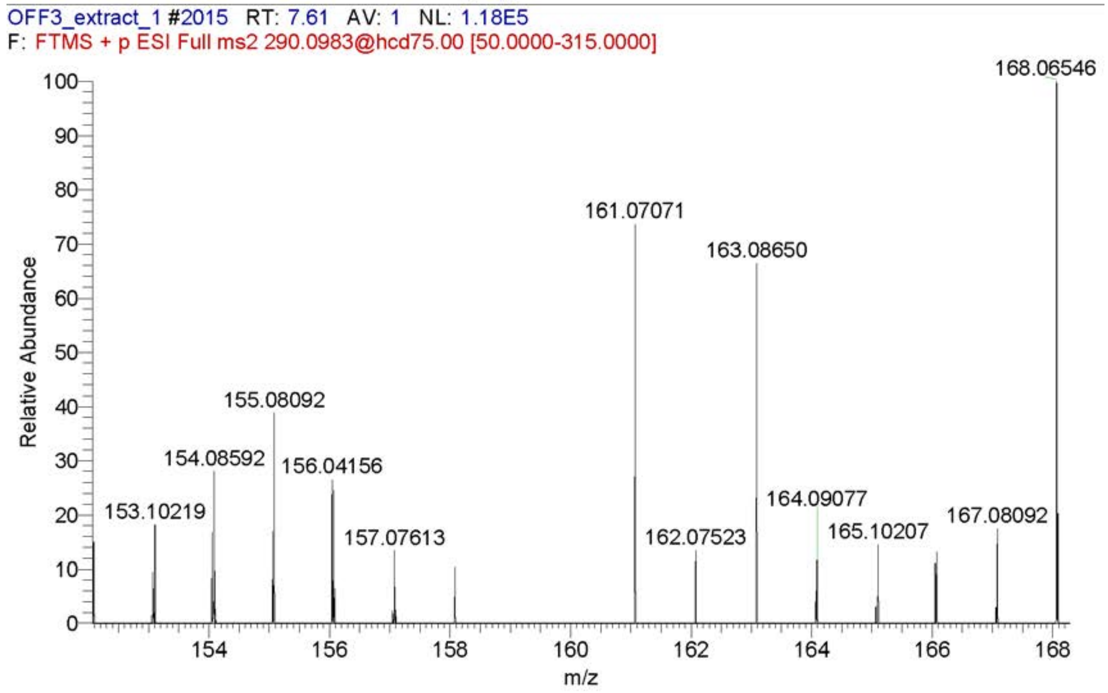
OFF3_extract_1 #2015 RT: 7.61 AV: 1 NL: 1.18E5
F: FTMS + p ESI Full ms2 290.0983@hcd75.00 [50.0000-315.0000]
168.06546
161.07071
163.08650
155.08092
154.08592
156.04156
153.10219
157.07613
162.07523
164.09077
165.10207
167.08092
Relative Abundance
m/z

S. mcgrathi

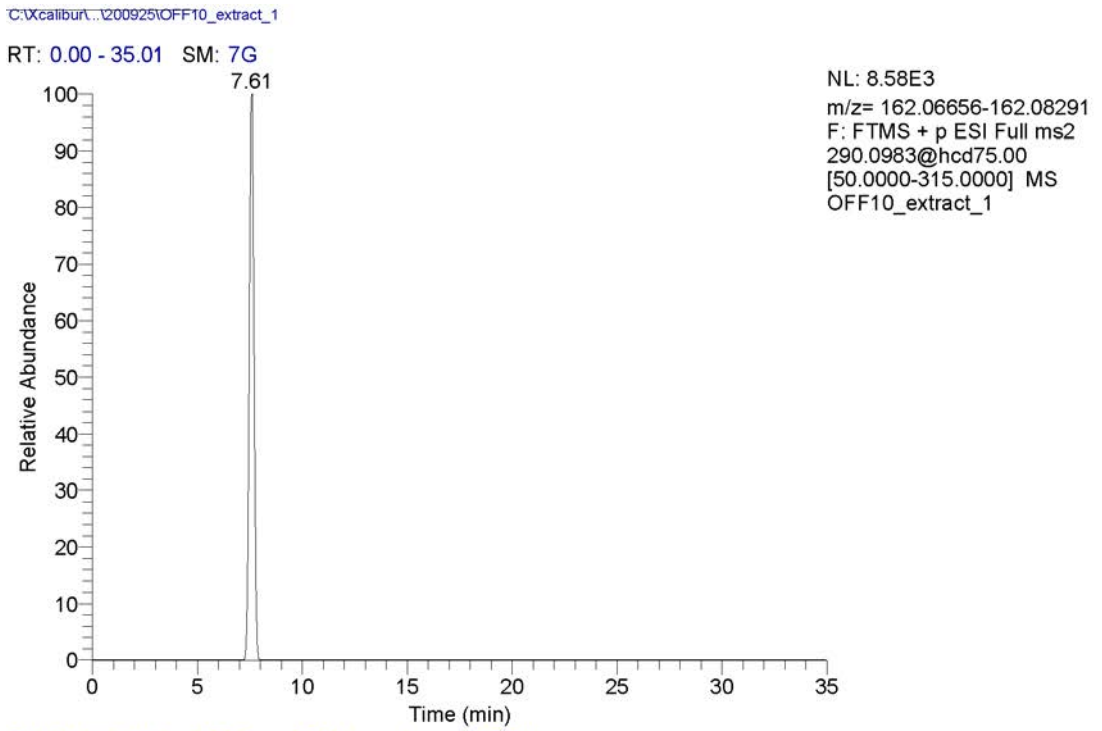
C:\Xcalibur\...\200925\OFF10_extract_1
RT: 0.00 - 35.01 SM: 7G
7.61
NL: 8.58E3
m/z= 162.06656-162.08291
F: FTMS + p ESI Full ms2
290.0983@hcd75.00
[50.0000-315.0000] MS
OFF10_extract_1
Relative Abundance
Time (min)

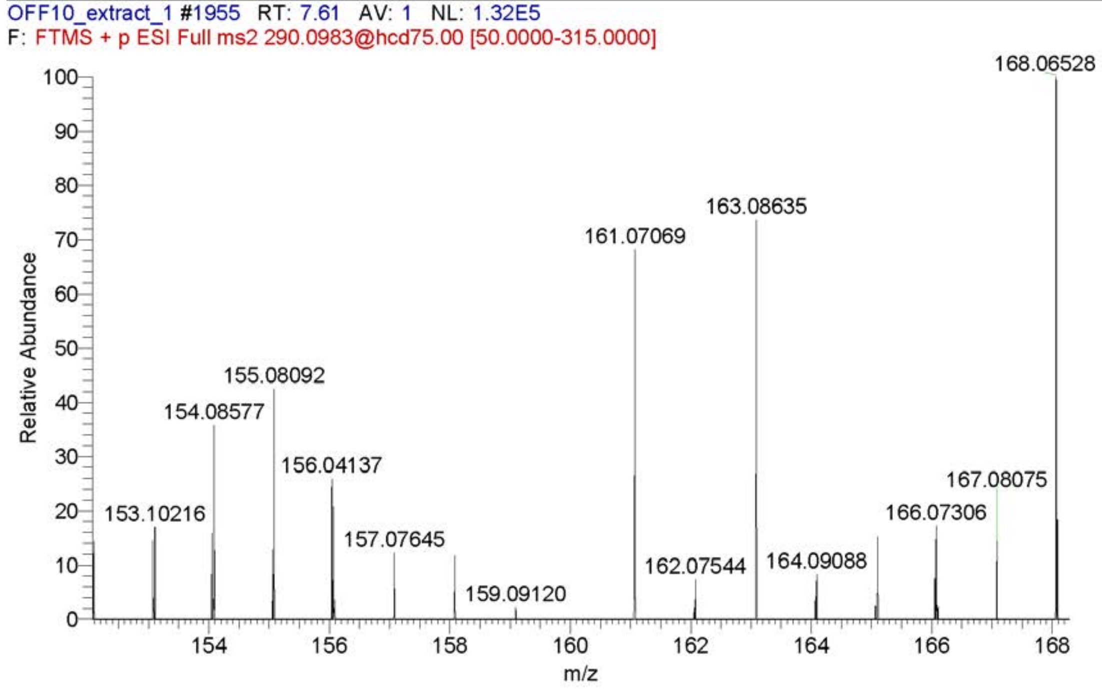
OFF10_extract_1 #1955 RT: 7.61 AV: 1 NL: 1.32E5
F: FTMS + p ESI Full ms2 290.0983@hcd75.00 [50.0000-315.0000]
168.06528
163.08635
161.07069
155.08092
154.08577
156.04137
167.08075
166.07306
153.10216
157.07645
162.07544
164.09088
159.09120
Relative Abundance
m/z

S. mcgrathi

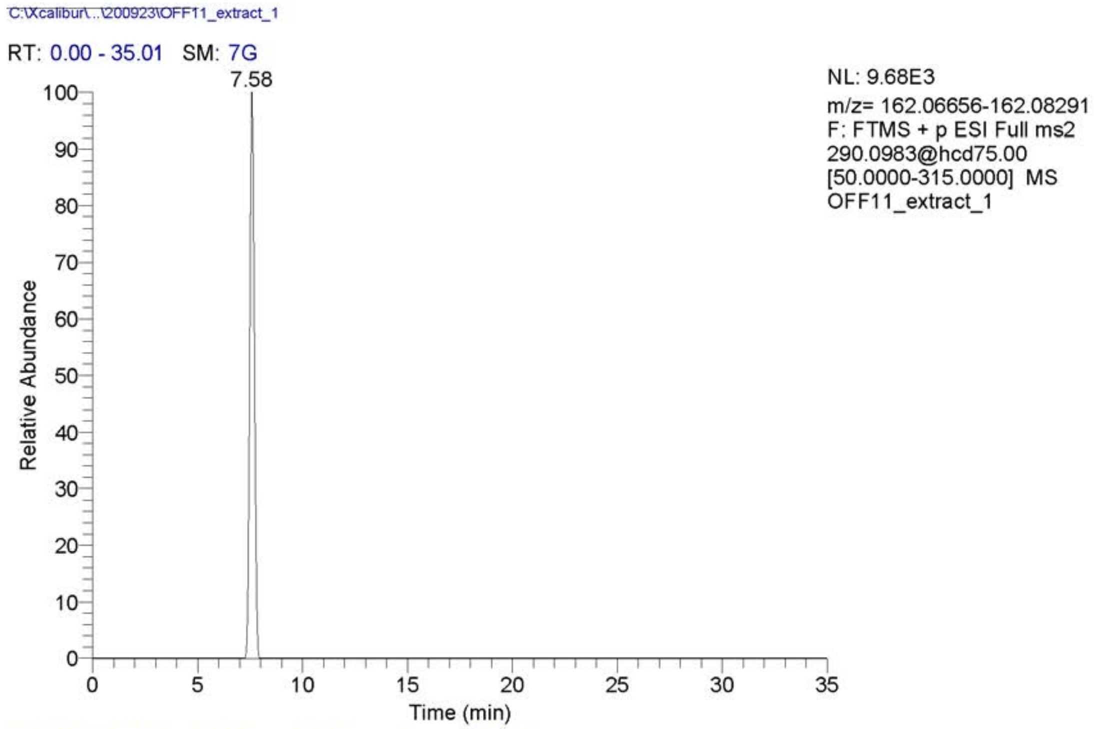
C:\Xcalibur\...\200923\OFF11_extract_1
RT: 0.00 - 35.01 SM: 7G
7.58
NL: 9.68E3
m/z= 162.06656-162.08291
F: FTMS + p ESI Full ms2
290.0983@hcd75.00
[50.0000-315.0000] MS
OFF11_extract_1
Relative Abundance
Time (min)

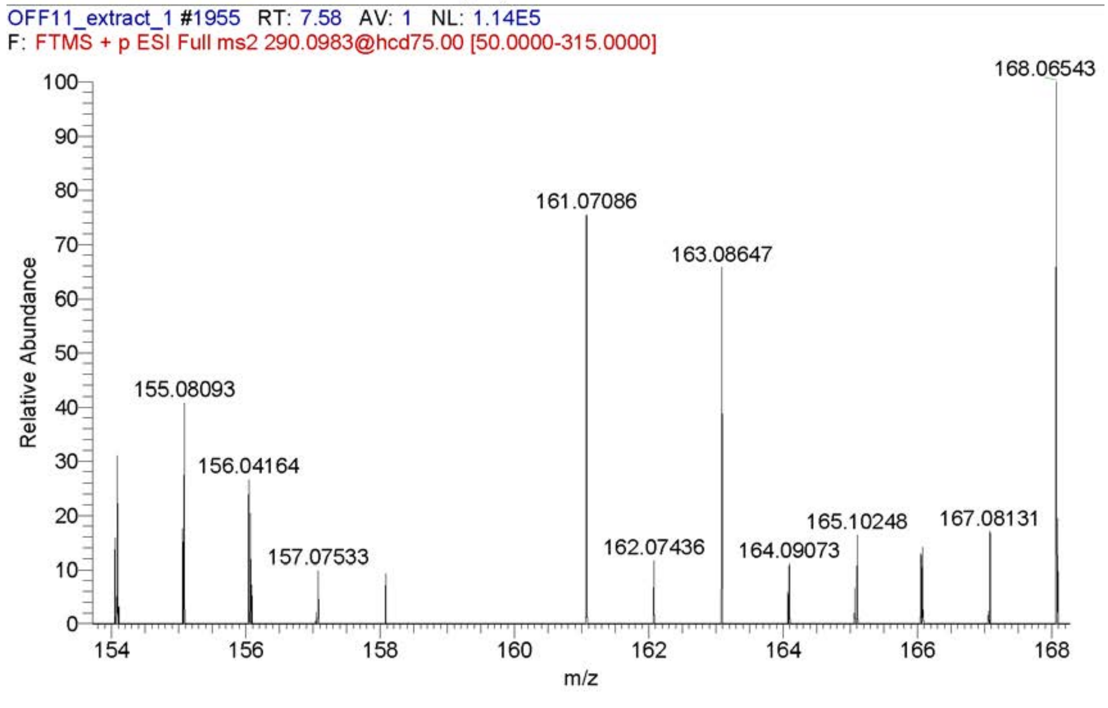
OFF11_extract_1 #1955 RT: 7.58 AV: 1 NL: 1.14E5
F: FTMS + p ESI Full ms2 290.0983@hcd75.00 [50.0000-315.0000]
168.06543
161.07086
163.08647
155.08093
156.04164
157.07533
162.07436
164.09073
165.10248
167.08131
Relative Abundance
m/z

S. mcgrathi

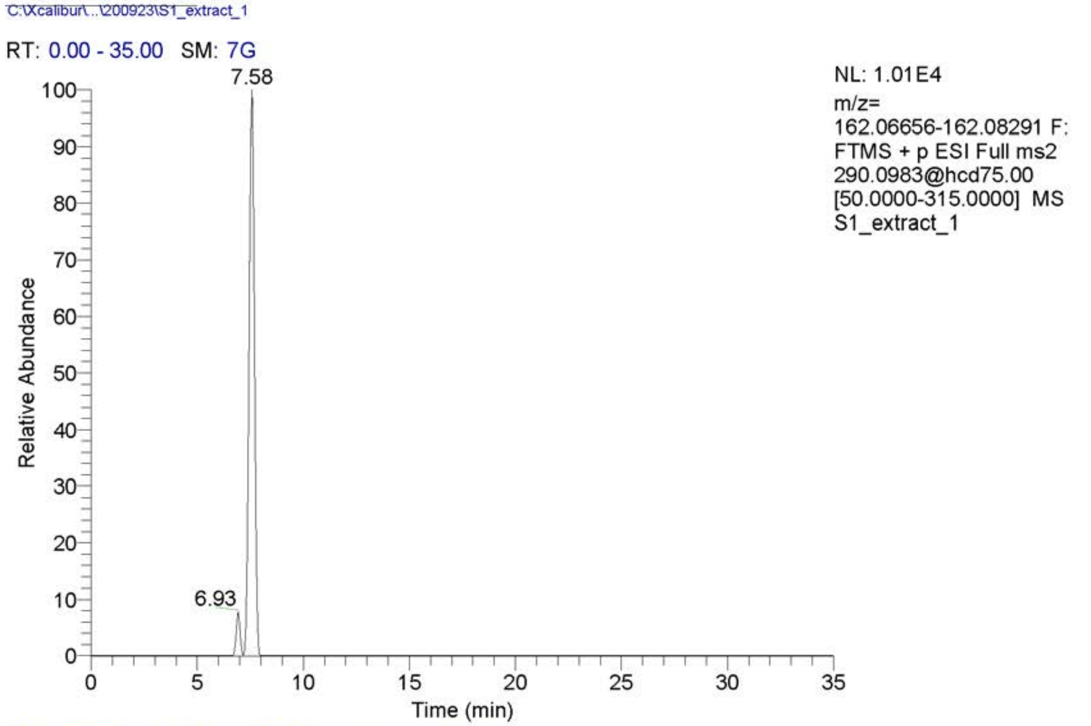

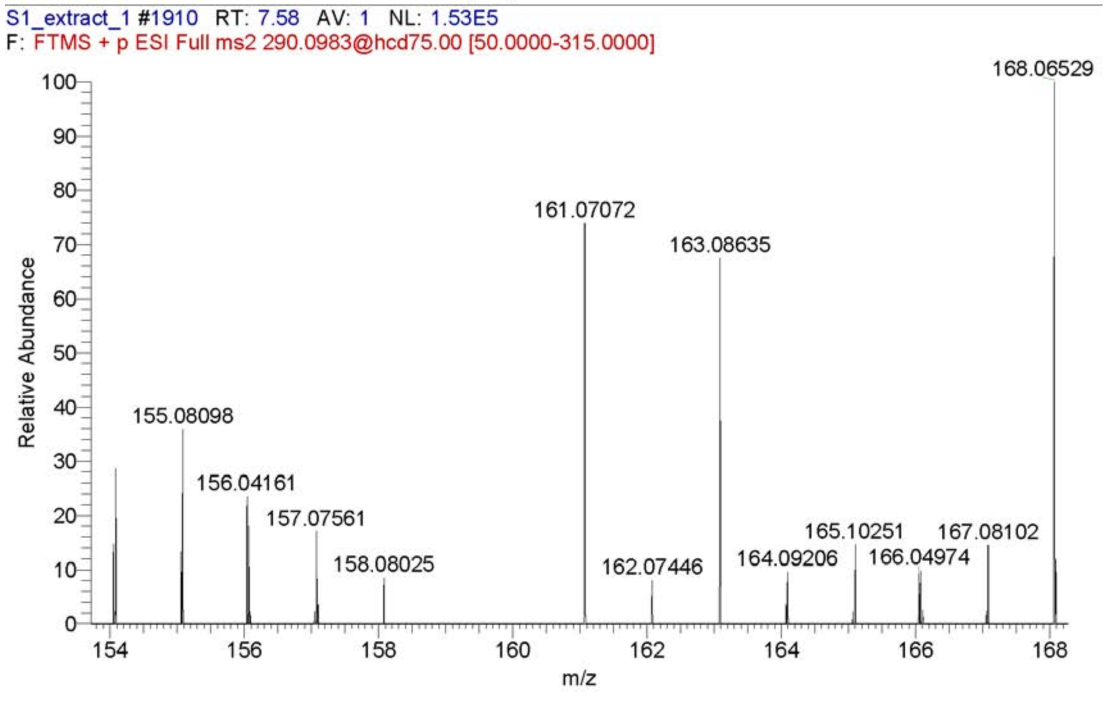

Stylochus sp. 1

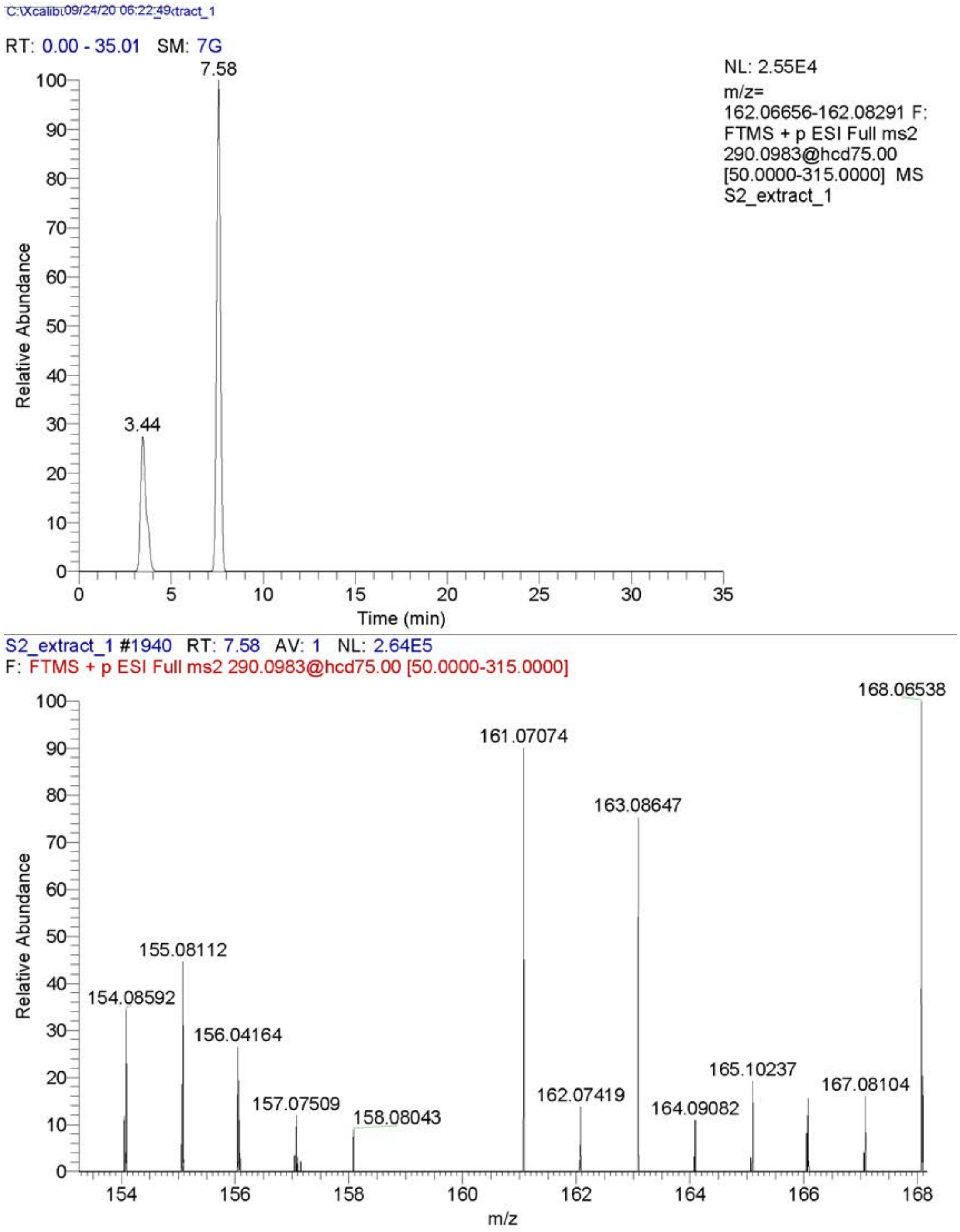

Stylochus sp.1

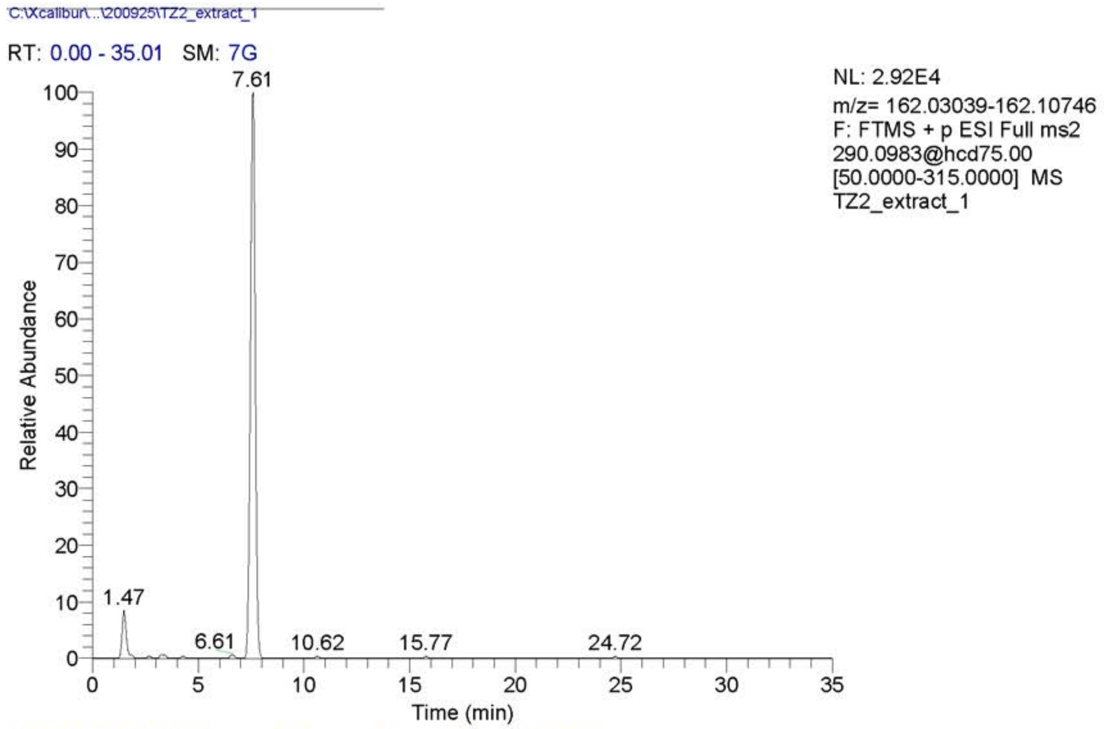

C:\Xcalibur\...\200925\TZ2_extract_1
RT: 0.00 - 35.01 SM: 7G
NL: 2.92E4
m/z= 162.03039-162.10746
F: FTMS + p ESI Full ms2
290.0983@hcd75.00
[50.0000-315.0000] MS
TZ2_extract_1
7.61
1.47
6.61
10.62
15.77
24.72
Relative Abundance
Time (min)

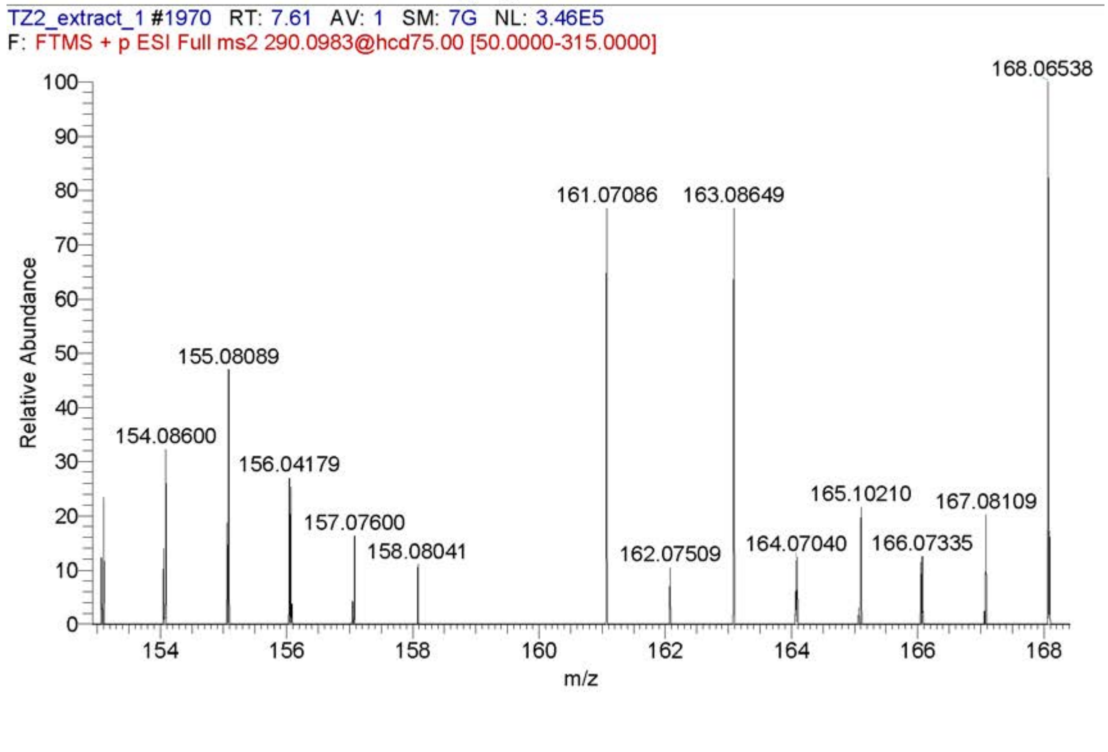

TZ2_extract_1 #1970 RT: 7.61 AV: 1 SM: 7G NL: 3.46E5
F: FTMS + p ESI Full ms2 290.0983@hcd75.00 [50.0000-315.0000]
168.06538
161.07086
163.08649
155.08089
154.08600
156.04179
157.07600
158.08041
162.07509
164.07040
165.10210
166.07335
167.08109
Relative Abundance
m/z

T. brocchii

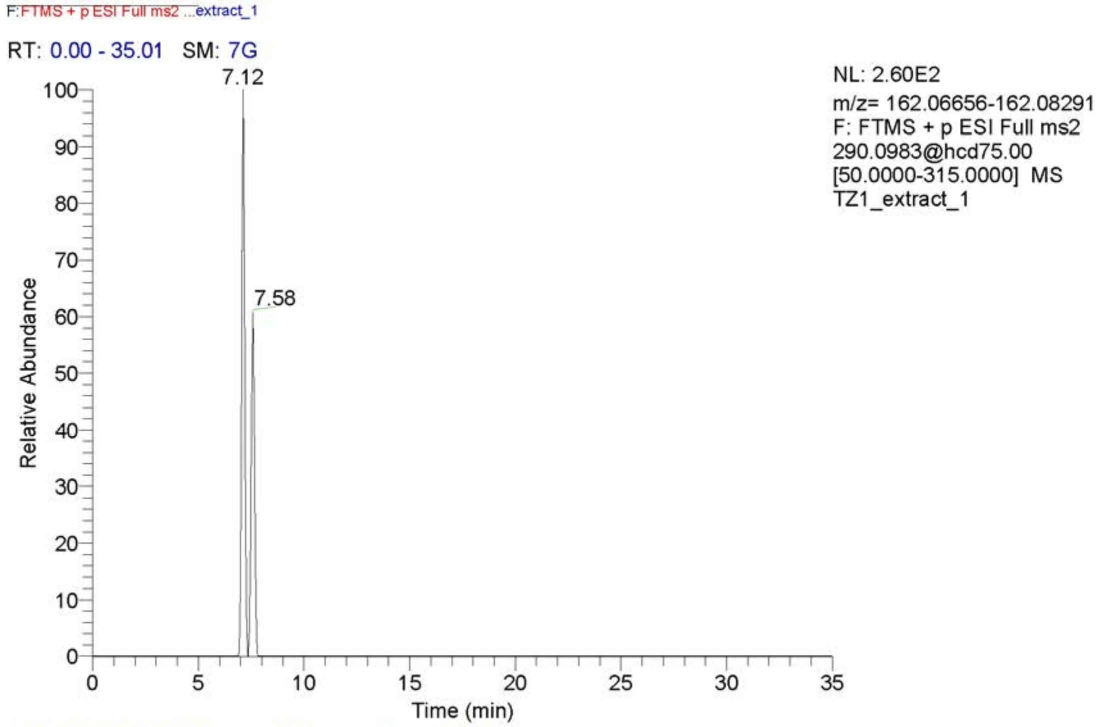
F: FTMS + p ESI Full ms2 ...extract_1
RT: 0.00 - 35.01 SM: 7G
NL: 2.60E2
m/z= 162.06656-162.08291
F: FTMS + p ESI Full ms2
290.0983@hcd75.00
[50.0000-315.0000] MS
TZ1_extract_1
7.12
7.58
Relative Abundance
Time (min)

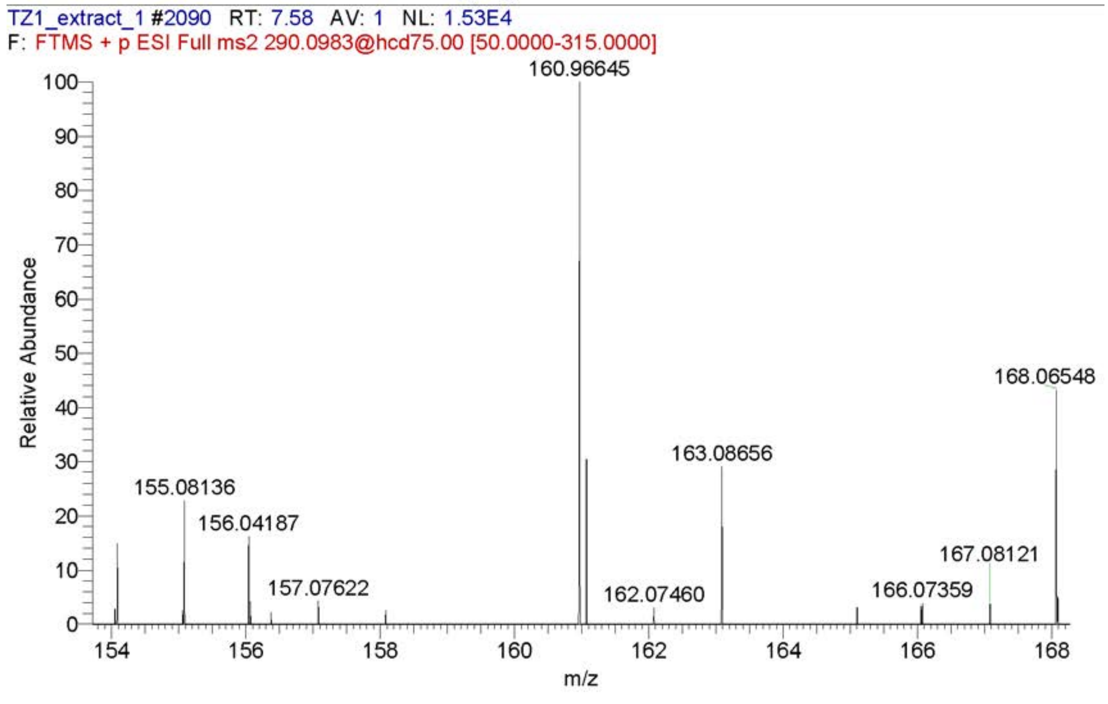
TZ1_extract_1 #2090 RT: 7.58 AV: 1 NL: 1.53E4
F: FTMS + p ESI Full ms2 290.0983@hcd75.00 [50.0000-315.0000]
160.96645
168.06548
163.08656
155.08136
156.04187
167.08121
157.07622
162.07460
166.07359
Relative Abundance
m/z

T. brocchii

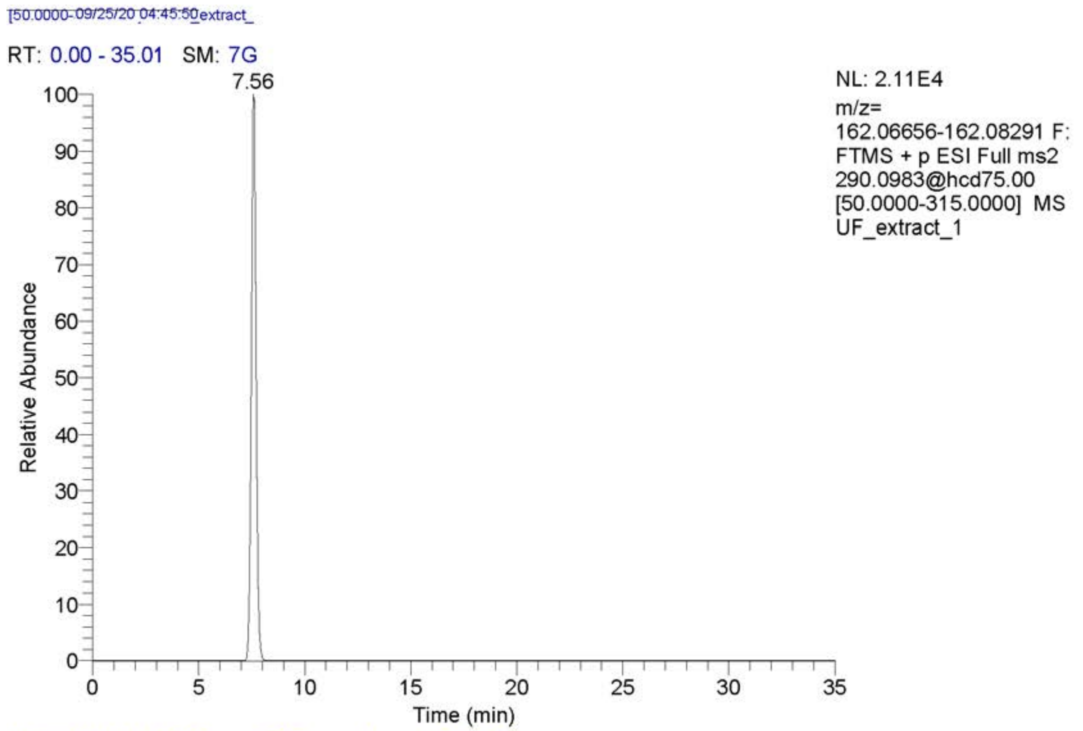

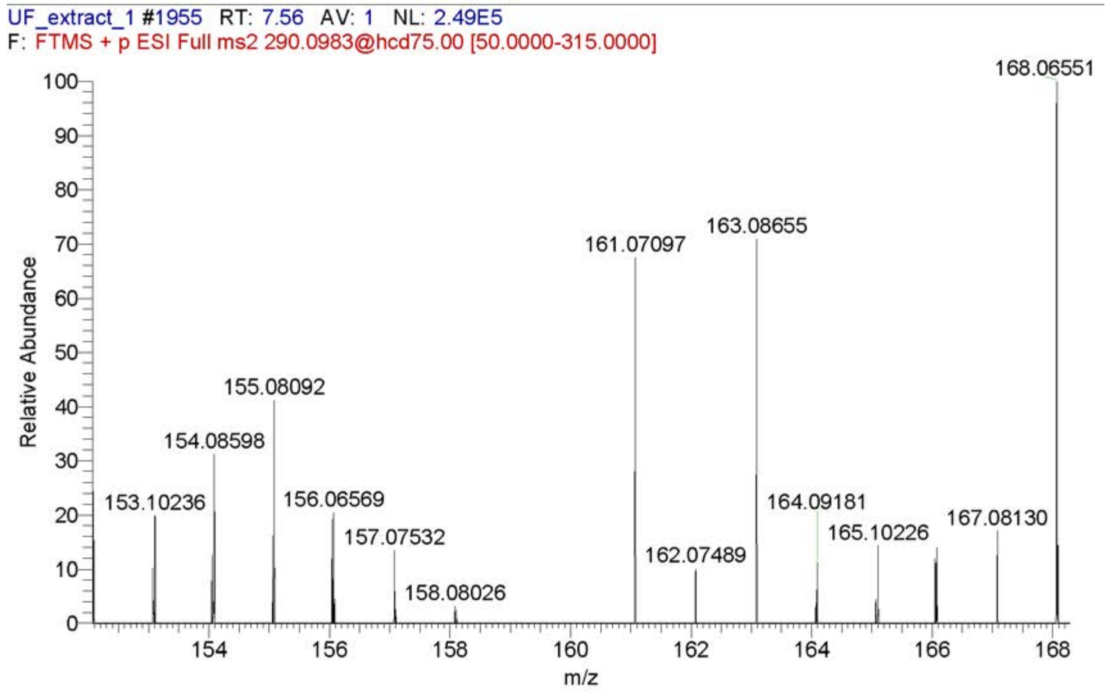

N. longiducta

6,11-dideoxyTTX (4) m/z 288.1190

This analyte was considered present when the mass elective scan showed the daughter ion 162.07 at similar retention times. This daughter ion was taken from Bane, Lehane, Dikshit, O'Riordan and Furey [1]. The samples that were reported to contain this analyte was *S. mcgrathi* (RT: 4.91), *S. mcgrathi* (RT: 4.86), *S. mcgrathi* (RT: 4.75), *S. mcgrathi* (RT: 4.82), *S. mcgrathi* (RT: 4.78), *S. mcgrathi* (RT: 4.89), *S. mcgrathi* (RT: 4.89), *S. mcgrathi* (RT: 4.89), *E. celerrima* (RT: 4.80), *E. celerrima* (RT: 4.84), *E. celerrima* (RT: 4.84), *E. celerrima* (RT: 4.78), *N. longiducta* (RT: 4.78), *Pseudoceros sp. 1* (RT: 4.91), *P. velutinus* (RT: 4.84), and *Cycloporus sp*. (RT: 4.85).

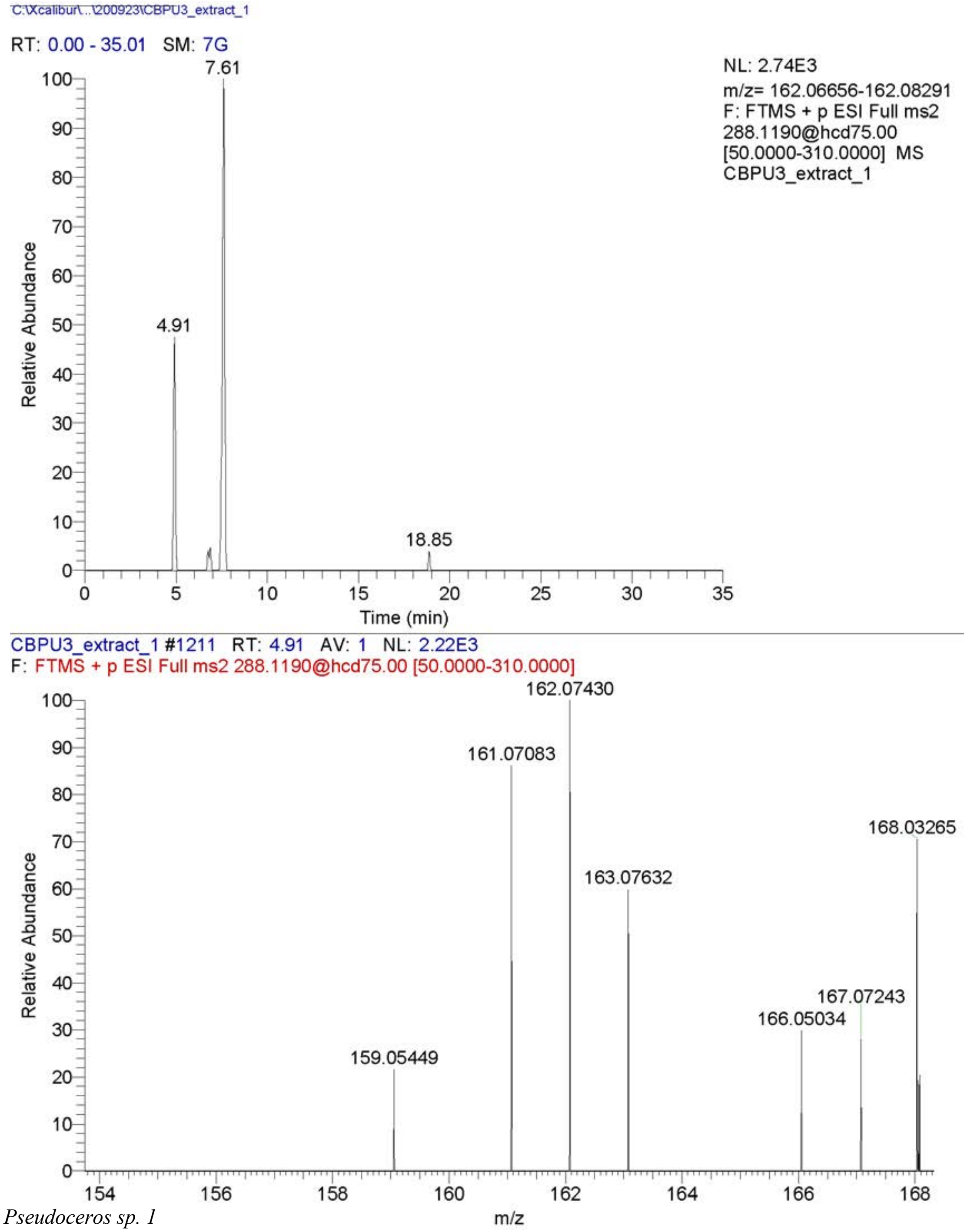

Pseudoceros sp. 1

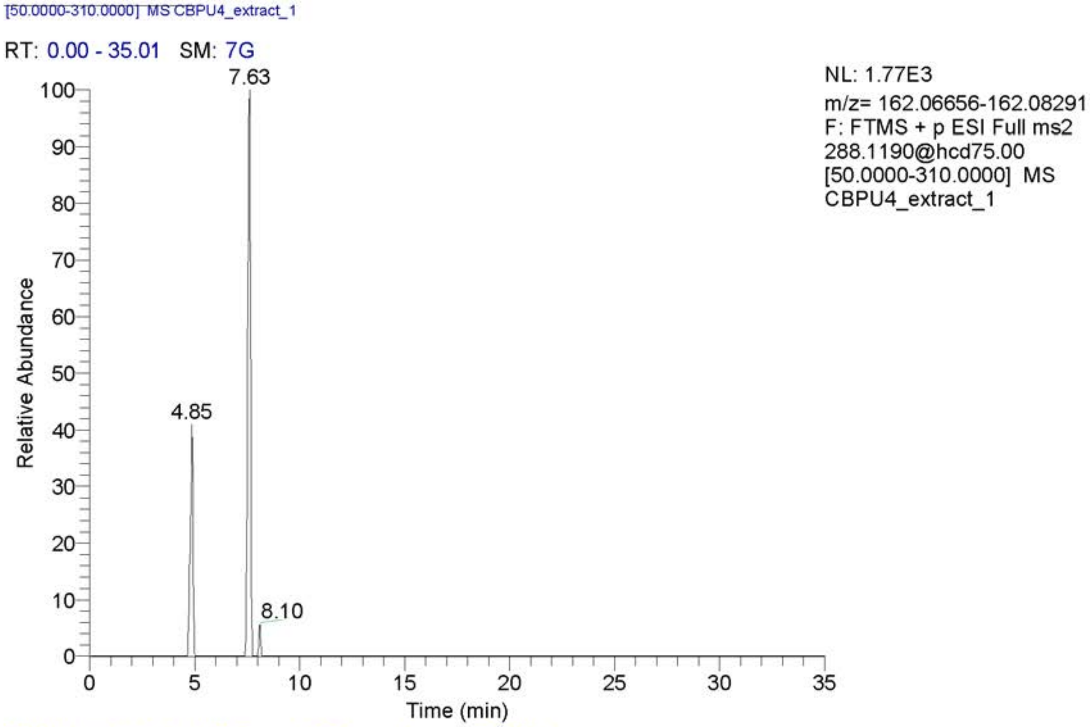

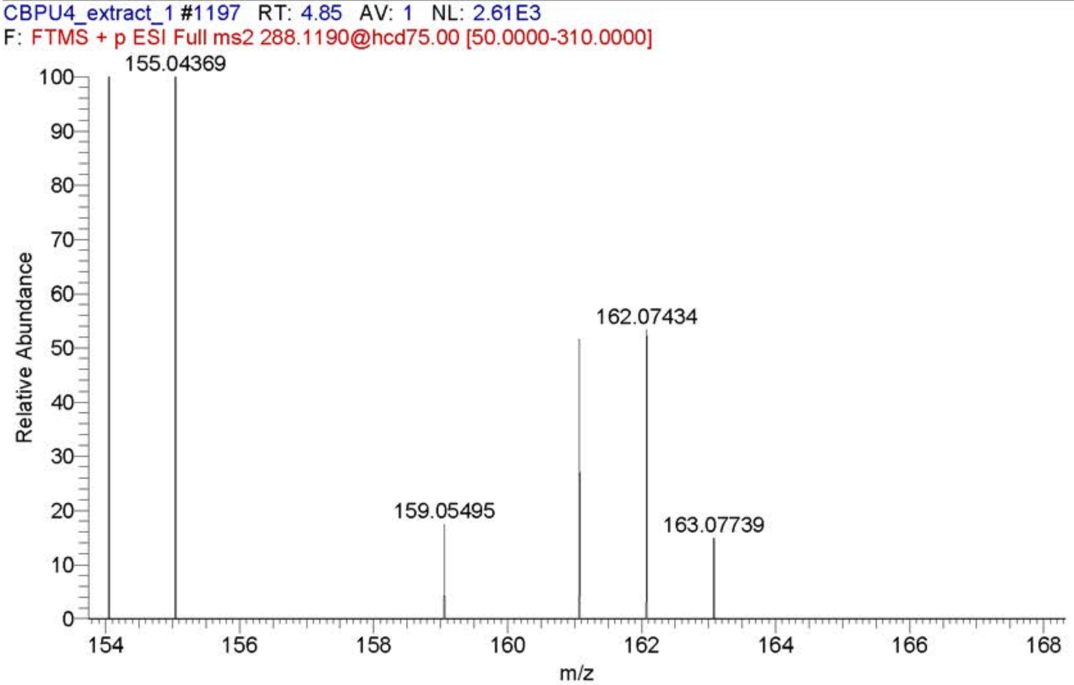

Cycloporus sp.

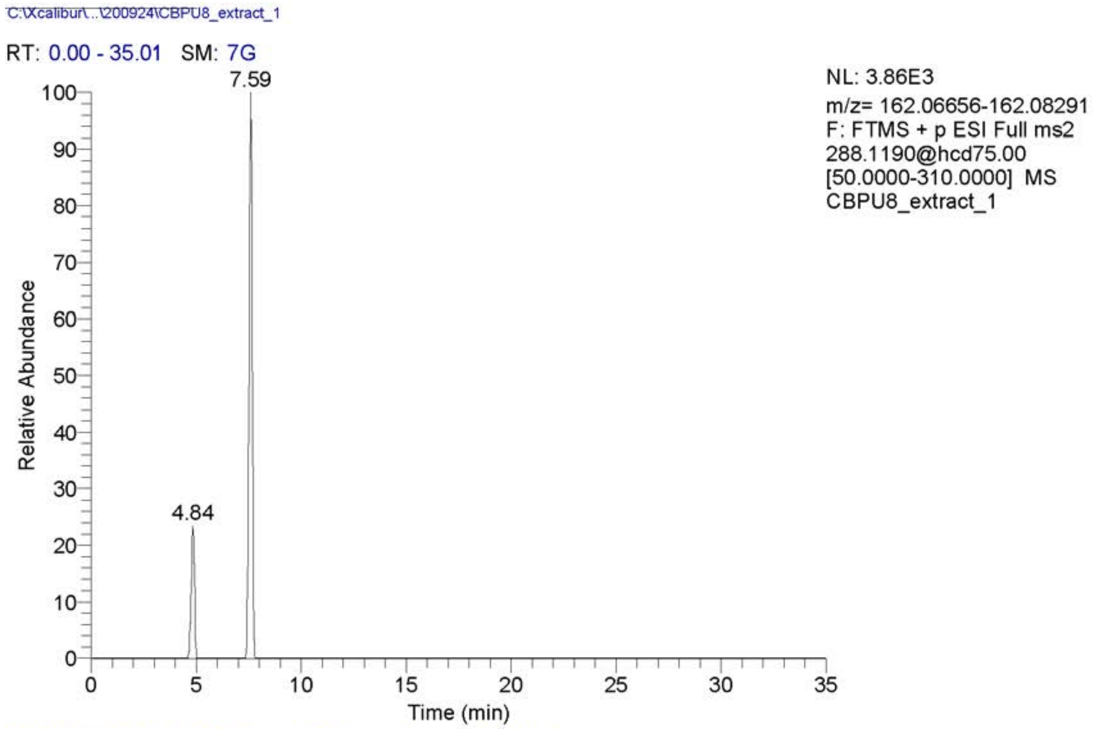
C:\Xcalibur\...\200924\CBPU8_extract_1
RT: 0.00 - 35.01 SM: 7G
NL: 3.86E3
m/z= 162.06656-162.08291
F: FTMS + p ESI Full ms2 288.1190@hcd75.00 [50.0000-310.0000] MS CBPU8_extract_1
7.59
4.84
Relative Abundance
Time (min)

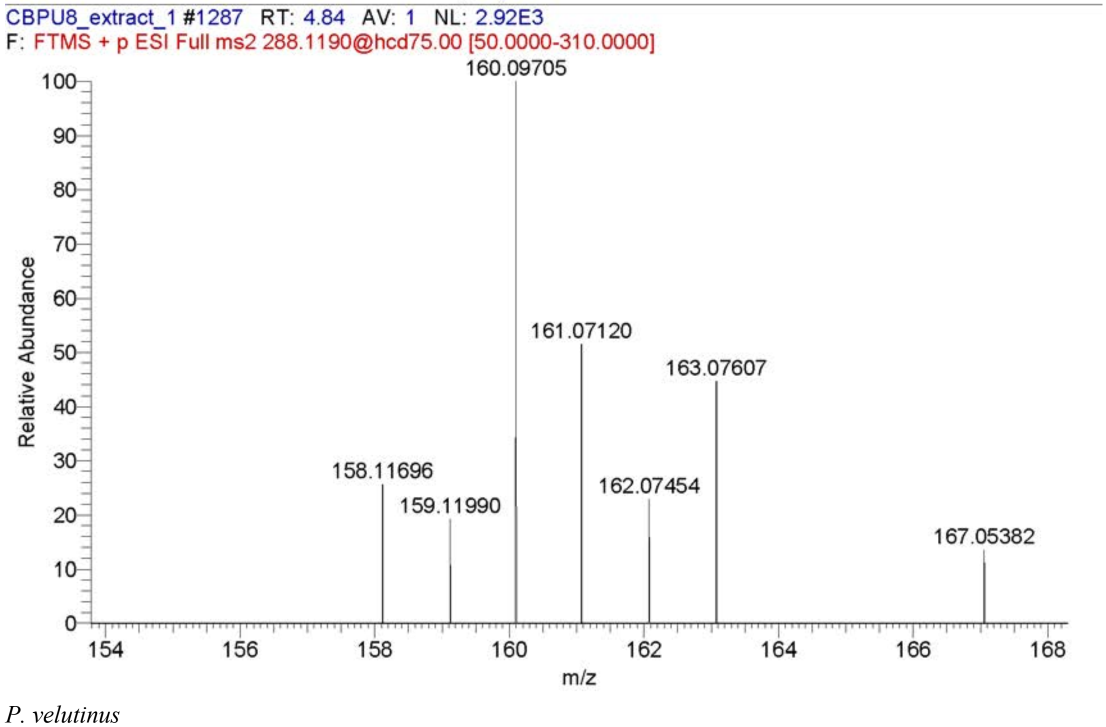
CBPU8_extract_1 #1287 RT: 4.84 AV: 1 NL: 2.92E3
F: FTMS + p ESI Full ms2 288.1190@hcd75.00 [50.0000-310.0000]
160.09705
161.07120
163.07607
158.11696
159.11990
162.07454
167.05382
Relative Abundance
m/z

P. velutinus

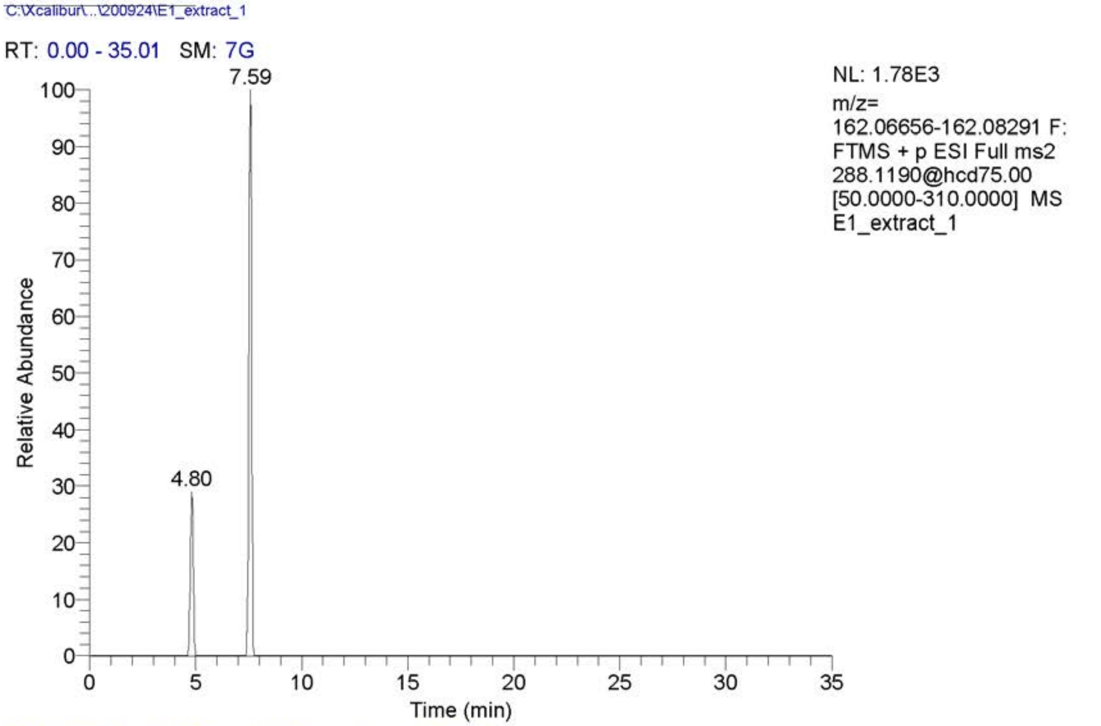
C:\Xcalibur\...\200924\E1_extract_1
RT: 0.00 - 35.01 SM: 7G
NL: 1.78E3
m/z=
162.06656-162.08291 F:
FTMS + p ESI Full ms2
288.1190@hcd75.00
[50.0000-310.0000] MS
E1_extract_1
7.59
4.80
Relative Abundance
Time (min)

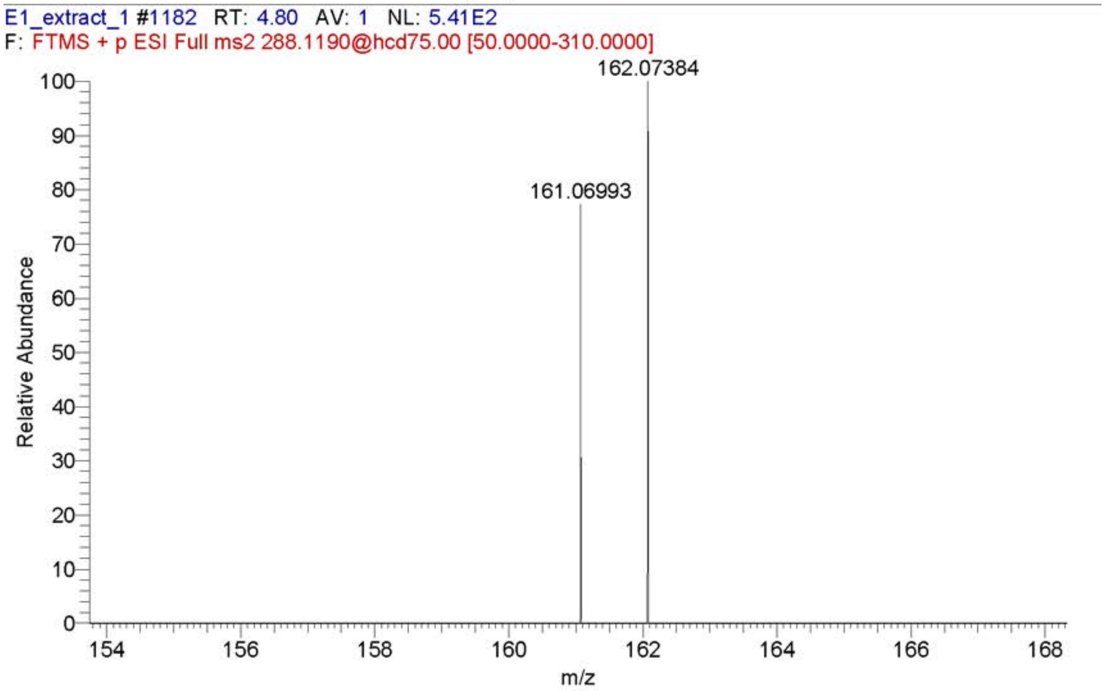
E1_extract_1 #1182 RT: 4.80 AV: 1 NL: 5.41E2
F: FTMS + p ESI Full ms2 288.1190@hcd75.00 [50.0000-310.0000]
162.07384
161.06993
Relative Abundance
m/z

E. celerrima

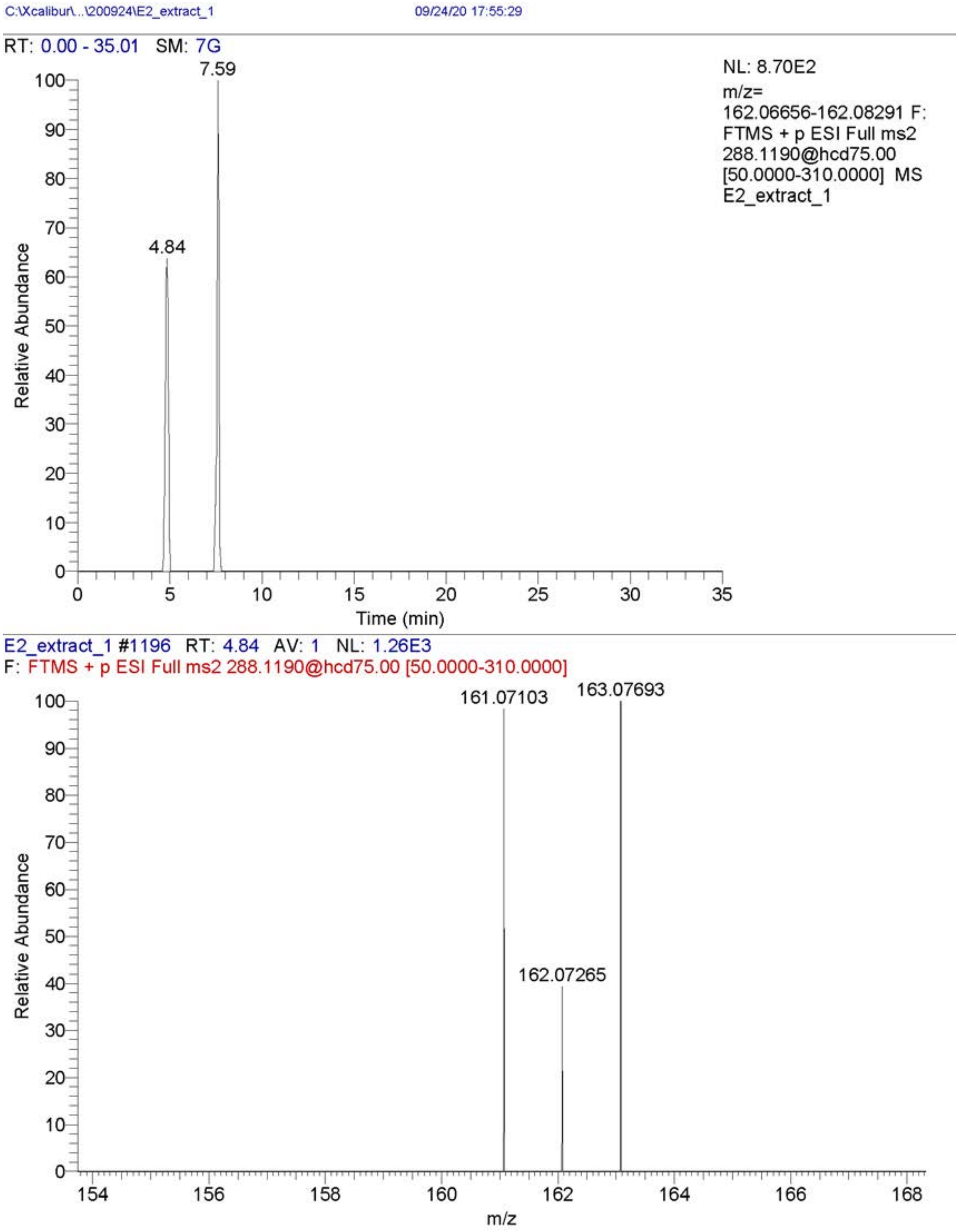
C:\Xcalibur\...\200924\E2_extract_1
09/24/20 17:55:29
RT: 0.00 - 35.01 SM: 7G
7.59
4.84
NL: 8.70E2
m/z=
162.06656-162.08291 F:
FTMS + p ESI Full ms2
288.1190@hcd75.00
[50.0000-310.0000] MS
E2_extract_1
Relative Abundance
Time (min)
E2_extract_1 #1196 RT: 4.84 AV: 1 NL: 1.26E3
F: FTMS + p ESI Full ms2 288.1190@hcd75.00 [50.0000-310.0000]
161.07103
163.07693
162.07265
Relative Abundance
m/z

E. celerrima

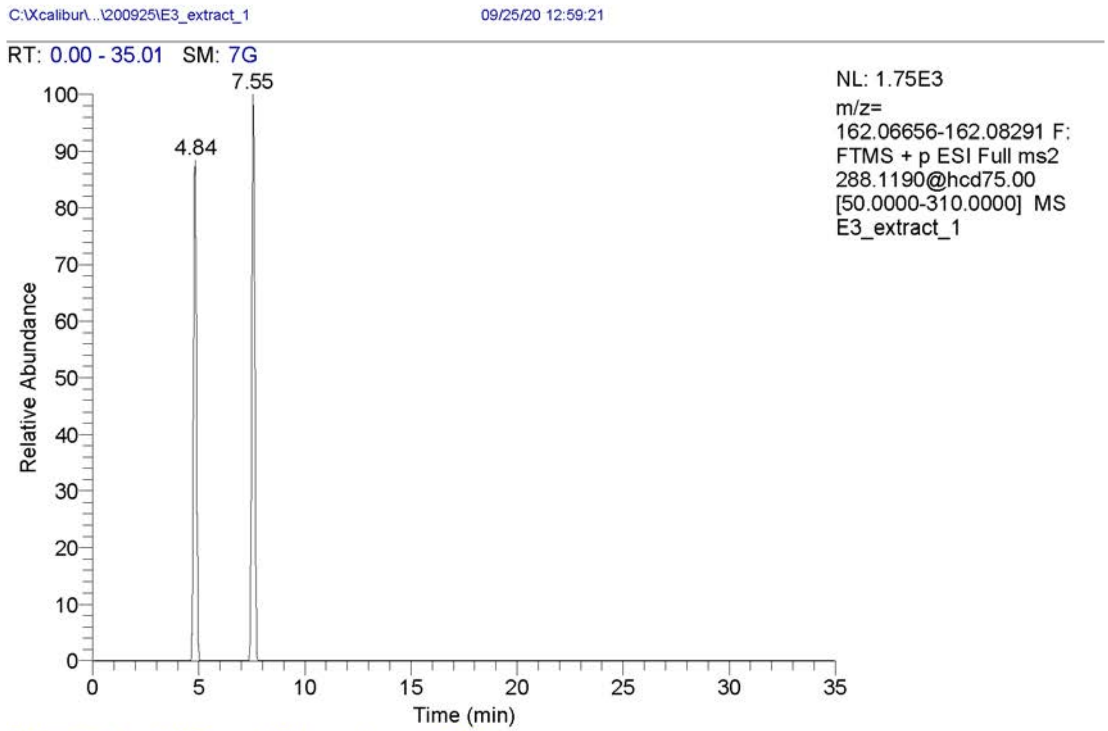
C:\Xcalibur\...\200925\E3_extract_1
09/25/20 12:59:21
RT: 0.00 - 35.01 SM: 7G
NL: 1.75E3
m/z=
162.06656-162.08291 F:
FTMS + p ESI Full ms2
288.1190@hcd75.00
[50.0000-310.0000] MS
E3_extract_1
7.55
4.84
Relative Abundance
Time (min)

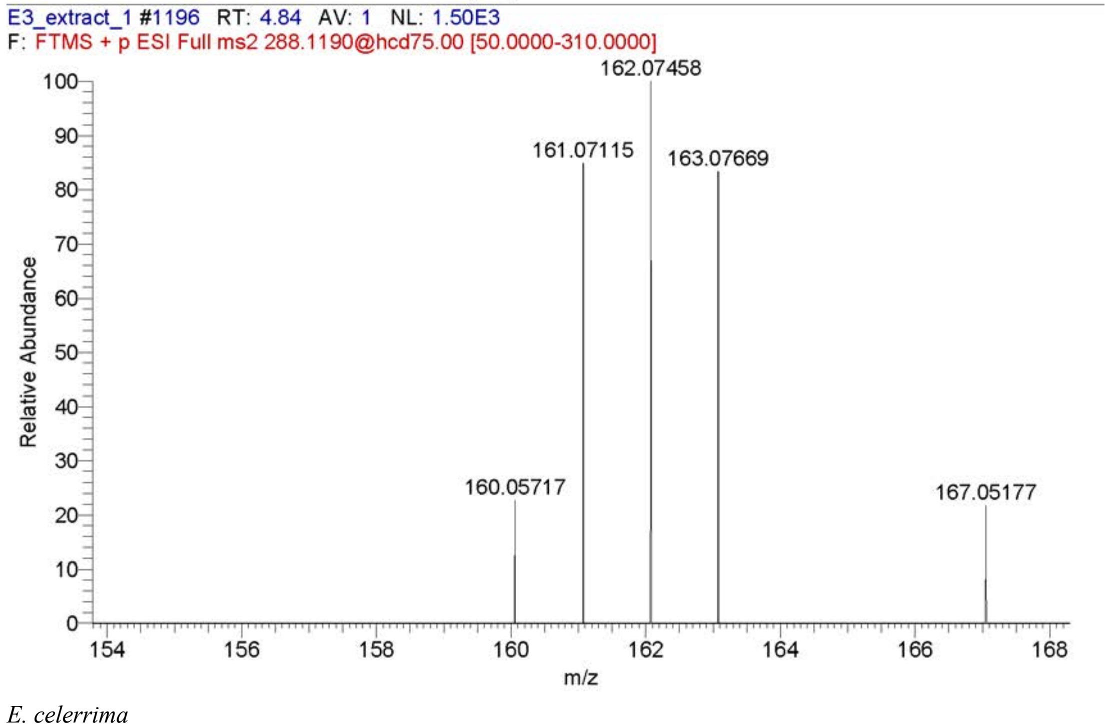
E3_extract_1 #1196 RT: 4.84 AV: 1 NL: 1.50E3
F: FTMS + p ESI Full ms2 288.1190@hcd75.00 [50.0000-310.0000]
162.07458
161.07115
163.07669
160.05717
167.05177
Relative Abundance
m/z

E. celerrima

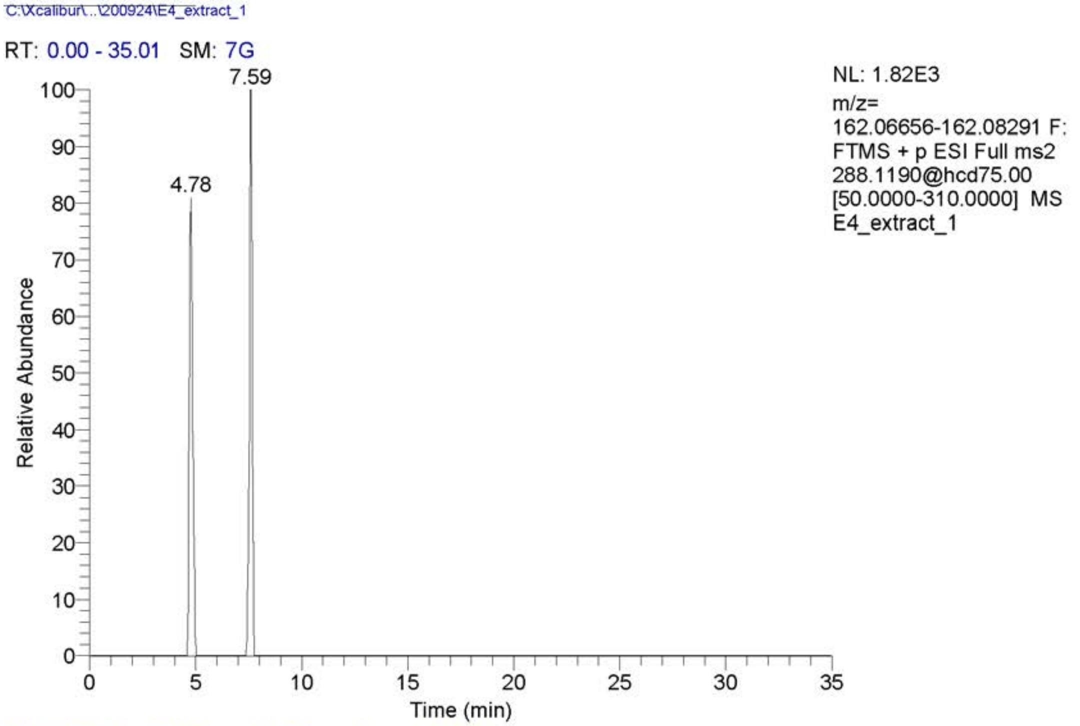
C:\Xcalibur\...\200924\E4_extract_1
RT: 0.00 - 35.01 SM: 7G
NL: 1.82E3
m/z=
162.06656-162.08291 F:
FTMS + p ESI Full ms2
288.1190@hcd75.00
[50.0000-310.0000] MS
E4_extract_1
7.59
4.78
Relative Abundance
Time (min)

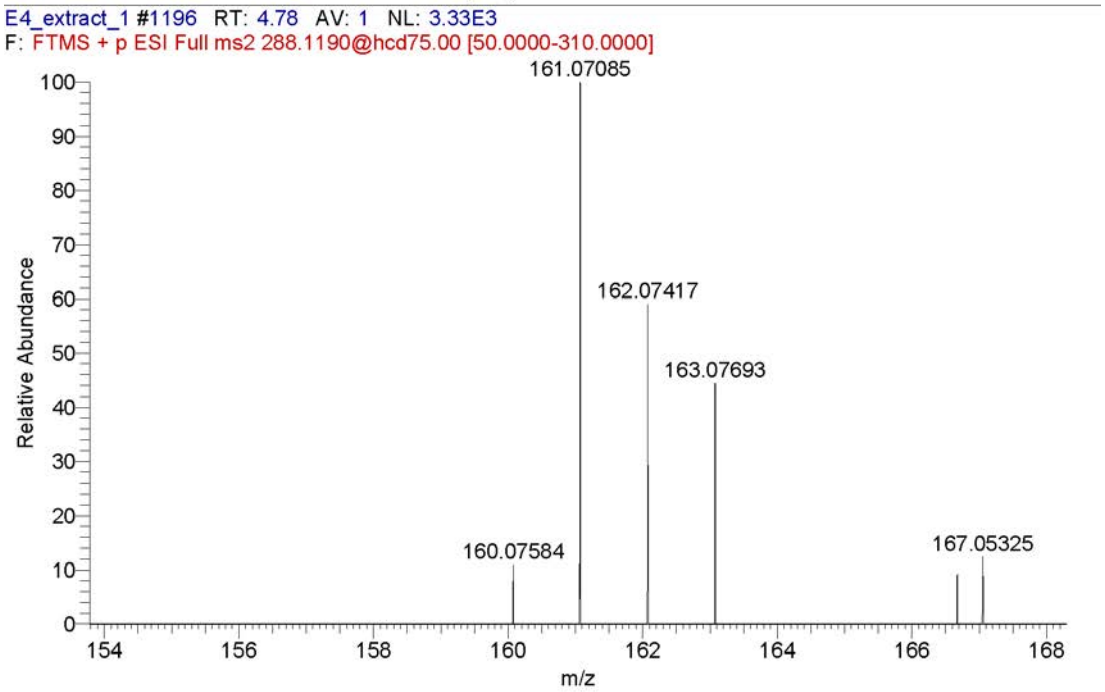
E4_extract_1 #1196 RT: 4.78 AV: 1 NL: 3.33E3
F: FTMS + p ESI Full ms2 288.1190@hcd75.00 [50.0000-310.0000]
161.07085
162.07417
163.07693
160.07584
167.05325
Relative Abundance
m/z

E. celerrima

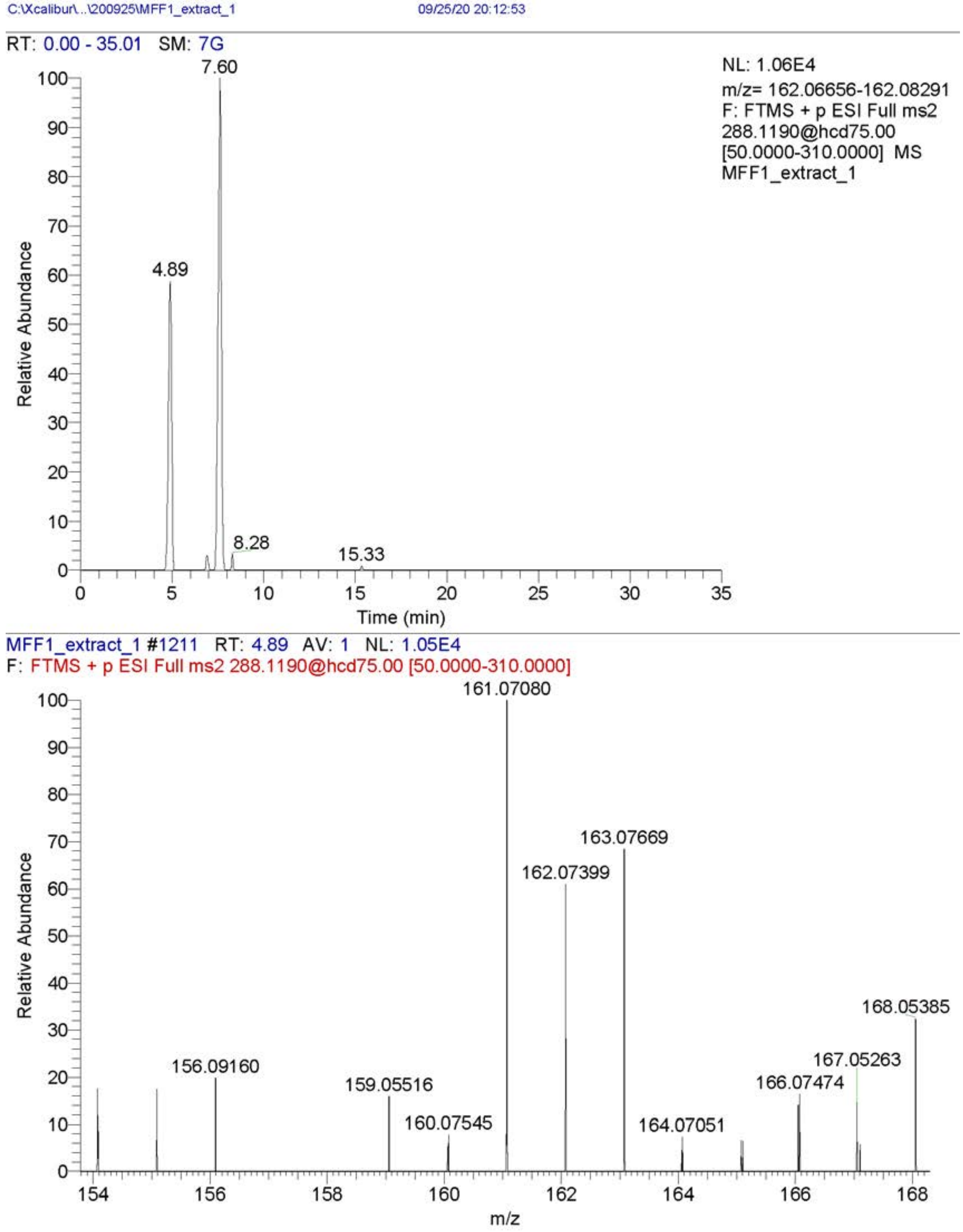
C:\Xcalibur\...\200925\MFF1_extract_1
09/25/20 20:12:53
RT: 0.00 - 35.01 SM: 7G
NL: 1.06E4
m/z= 162.06656-162.08291
F: FTMS + p ESI Full ms2
288.1190@hcd75.00
[50.0000-310.0000] MS
MFF1_extract_1
Relative Abundance
Time (min)
7.60
4.89
8.28
15.33
MFF1_extract_1 #1211 RT: 4.89 AV: 1 NL: 1.05E4
F: FTMS + p ESI Full ms2 288.1190@hcd75.00 [50.0000-310.0000]
161.07080
163.07669
162.07399
168.05385
167.05263
166.07474
156.09160
159.05516
160.07545
164.07051
m/z

S. mcgrathi

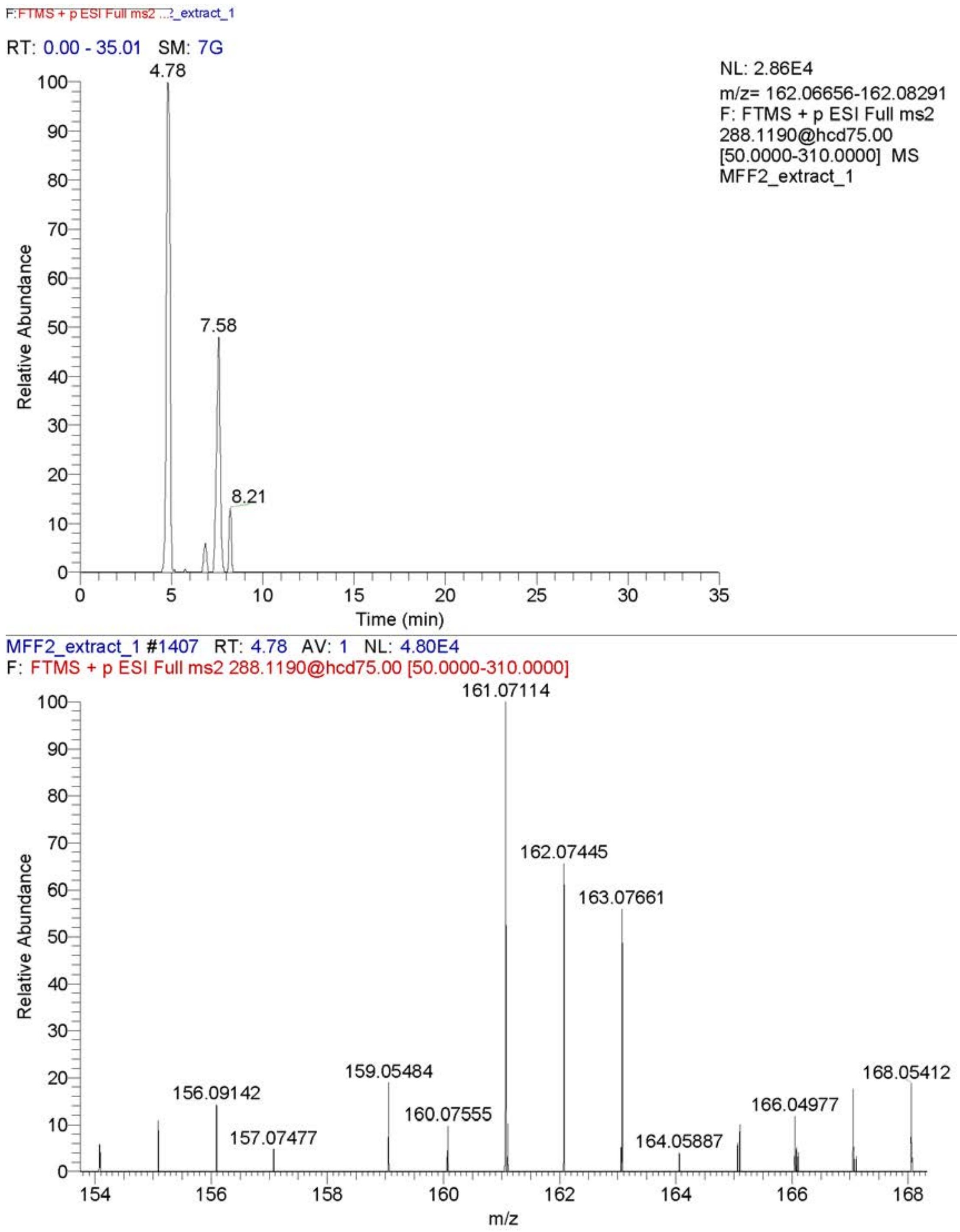
F: FTMS + p ESI Full ms2 ..._extract_1
RT: 0.00 - 35.01 SM: 7G
NL: 2.86E4
m/z= 162.06656-162.08291
F: FTMS + p ESI Full ms2
288.1190@hcd75.00
[50.0000-310.0000] MS
MFF2_extract_1
4.78
7.58
8.21
Relative Abundance
Time (min)
MFF2_extract_1 #1407 RT: 4.78 AV: 1 NL: 4.80E4
F: FTMS + p ESI Full ms2 288.1190@hcd75.00 [50.0000-310.0000]
161.07114
162.07445
163.07661
156.09142
157.07477
159.05484
160.07555
164.05887
166.04977
168.05412
m/z

S. mcgrathi

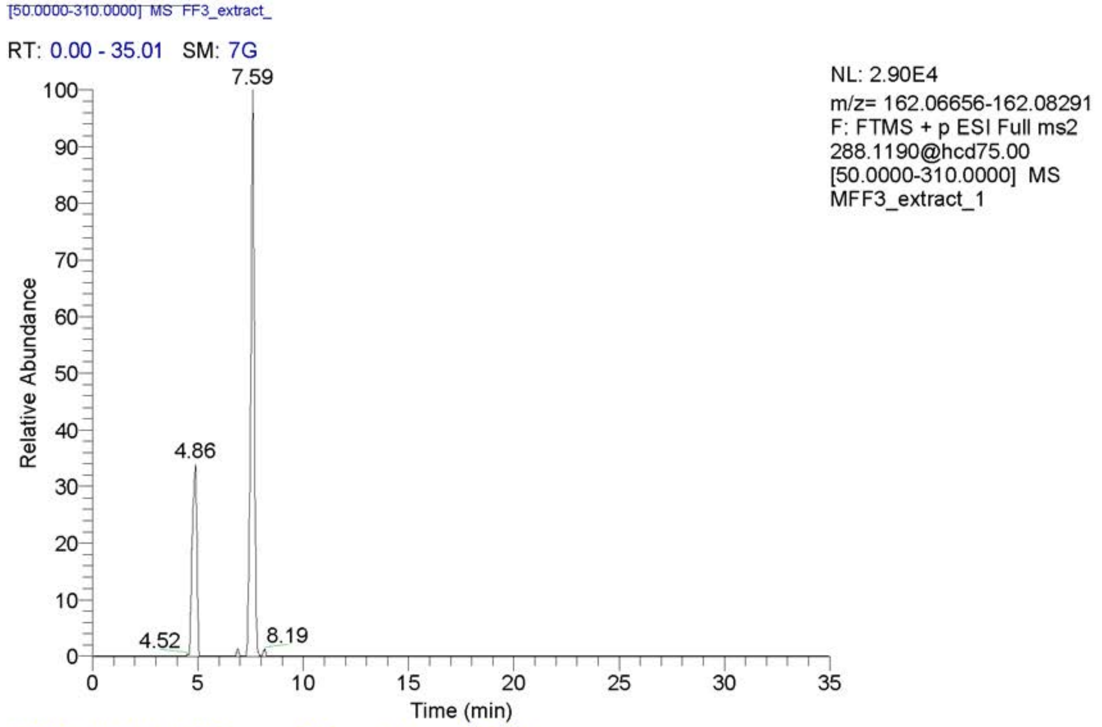

[50.0000-310.0000] MS FF3_extract_
RT: 0.00 - 35.01 SM: 7G
NL: 2.90E4
m/z= 162.06656-162.08291
F: FTMS + p ESI Full ms2
288.1190@hcd75.00
[50.0000-310.0000] MS
MFF3_extract_1
7.59
4.86
4.52
8.19
Relative Abundance
Time (min)

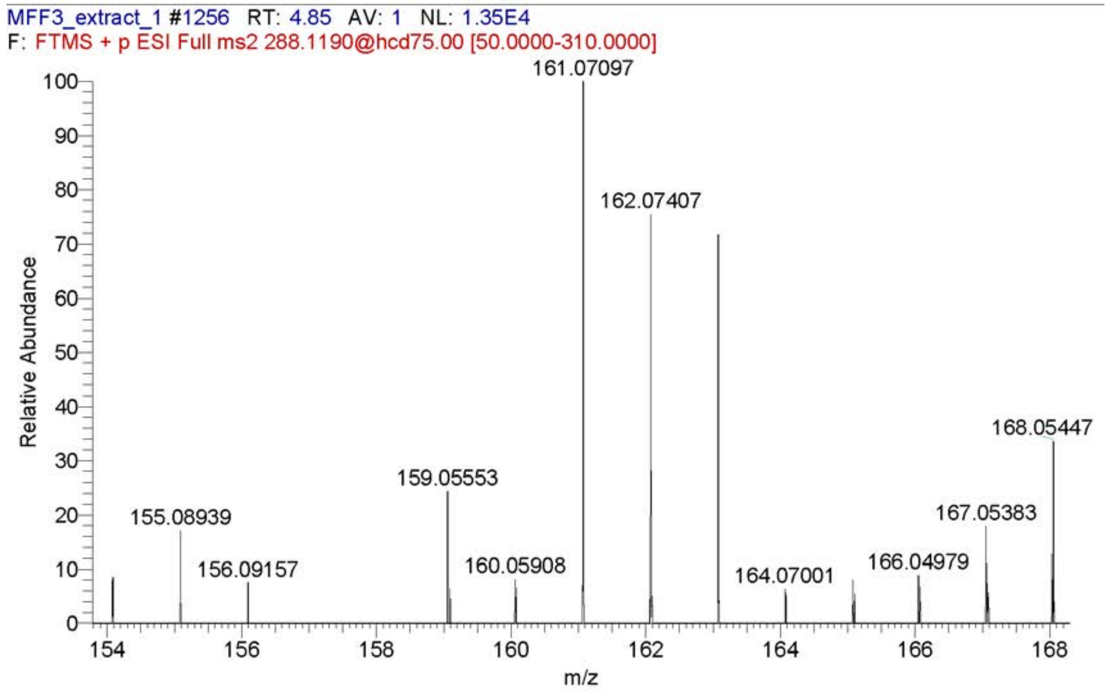

MFF3_extract_1 #1256 RT: 4.85 AV: 1 NL: 1.35E4
F: FTMS + p ESI Full ms2 288.1190@hcd75.00 [50.0000-310.0000]
161.07097
162.07407
168.05447
159.05553
167.05383
155.08939
156.09157
160.05908
164.07001
166.04979
Relative Abundance
m/z

S. mcgrathi

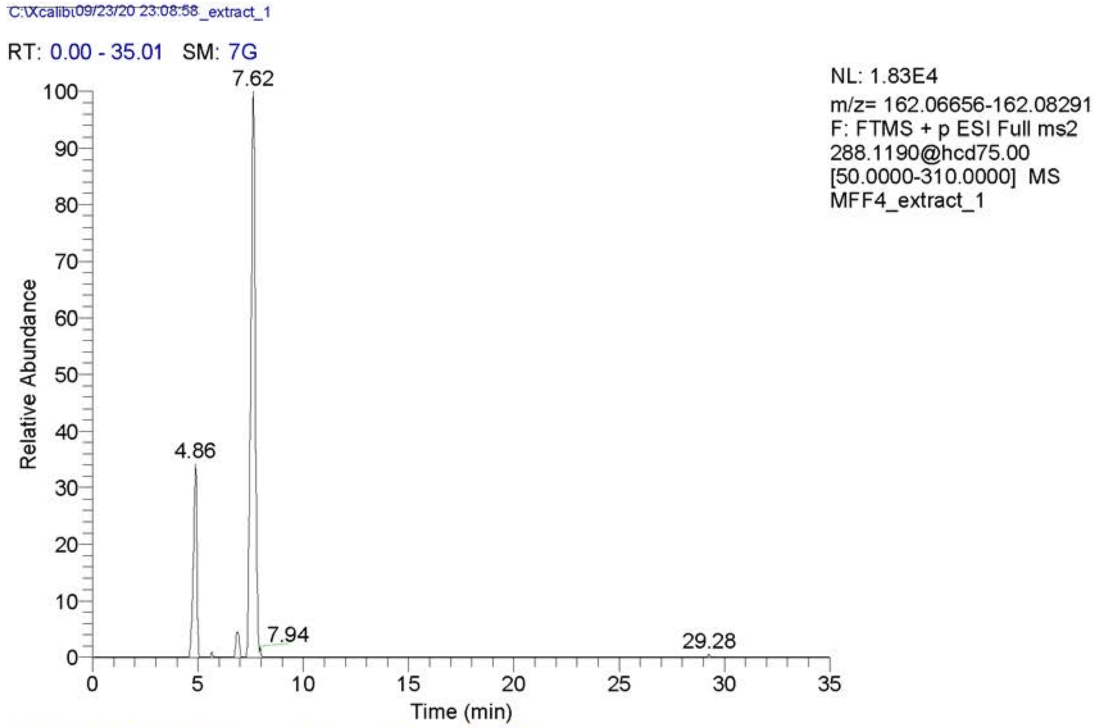
C:\Xcalibu\09/23/20 23:08:58_extract_1
RT: 0.00 - 35.01 SM: 7G
NL: 1.83E4
m/z= 162.06656-162.08291
F: FTMS + p ESI Full ms2
288.1190@hcd75.00
[50.0000-310.0000] MS
MFF4_extract_1
7.62
4.86
7.94
29.28
Relative Abundance
Time (min)

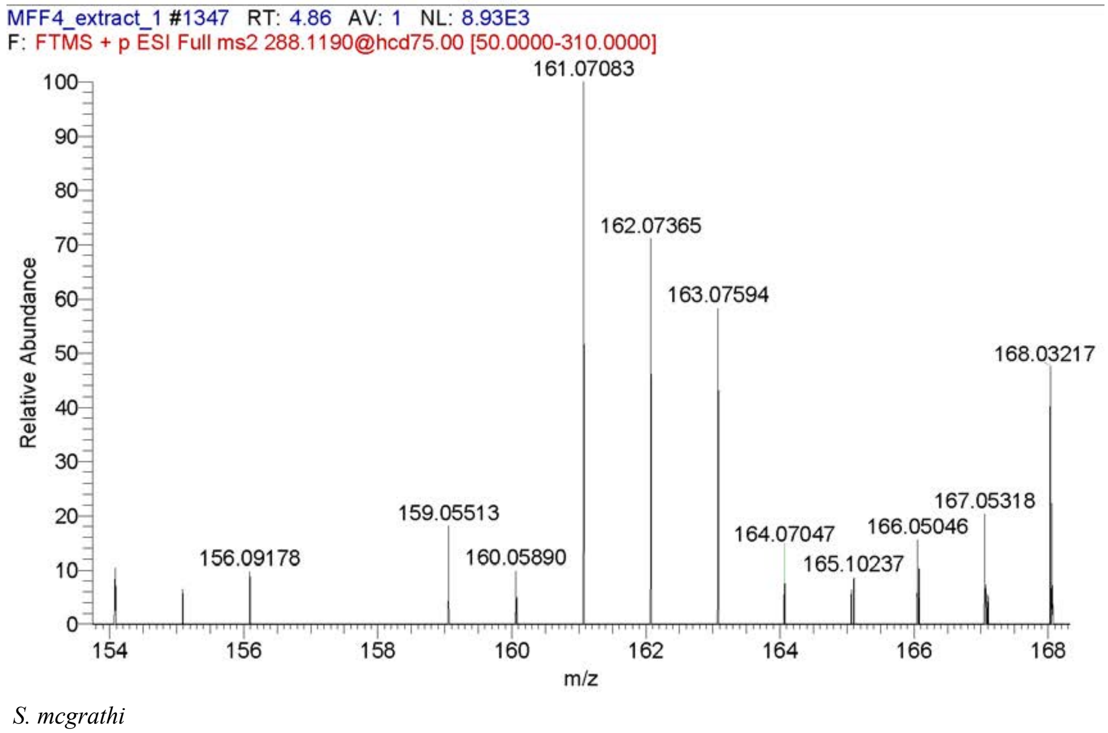
MFF4_extract_1 #1347 RT: 4.86 AV: 1 NL: 8.93E3
F: FTMS + p ESI Full ms2 288.1190@hcd75.00 [50.0000-310.0000]
161.07083
162.07365
163.07594
168.03217
167.05318
159.05513
164.07047
166.05046
165.10237
156.09178
160.05890
Relative Abundance
m/z

S. mcgrathi

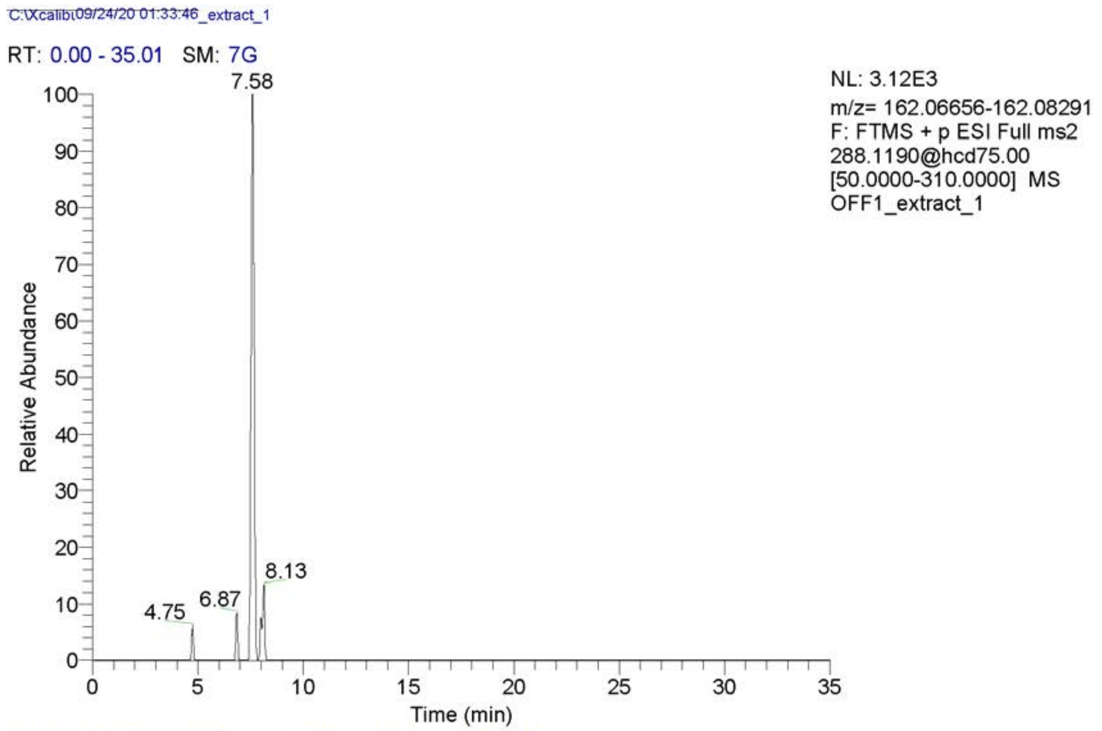
C:\Xcalibu09/24/20 01:33:46_extract_1
RT: 0.00 - 35.01 SM: 7G
NL: 3.12E3
m/z= 162.06656-162.08291
F: FTMS + p ESI Full ms2
288.1190@hcd75.00
[50.0000-310.0000] MS
OFF1_extract_1
7.58
8.13
6.87
4.75
Relative Abundance
Time (min)

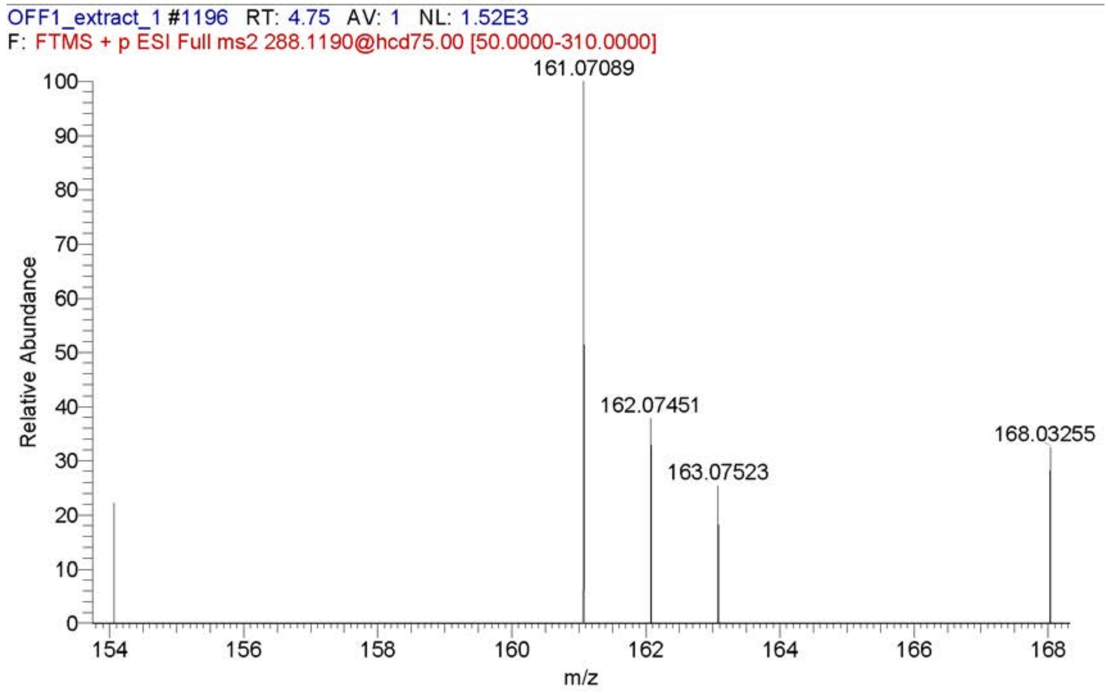
OFF1_extract_1 #1196 RT: 4.75 AV: 1 NL: 1.52E3
F: FTMS + p ESI Full ms2 288.1190@hcd75.00 [50.0000-310.0000]
161.07089
162.07451
163.07523
168.03255
Relative Abundance
m/z

S. mcgrathi

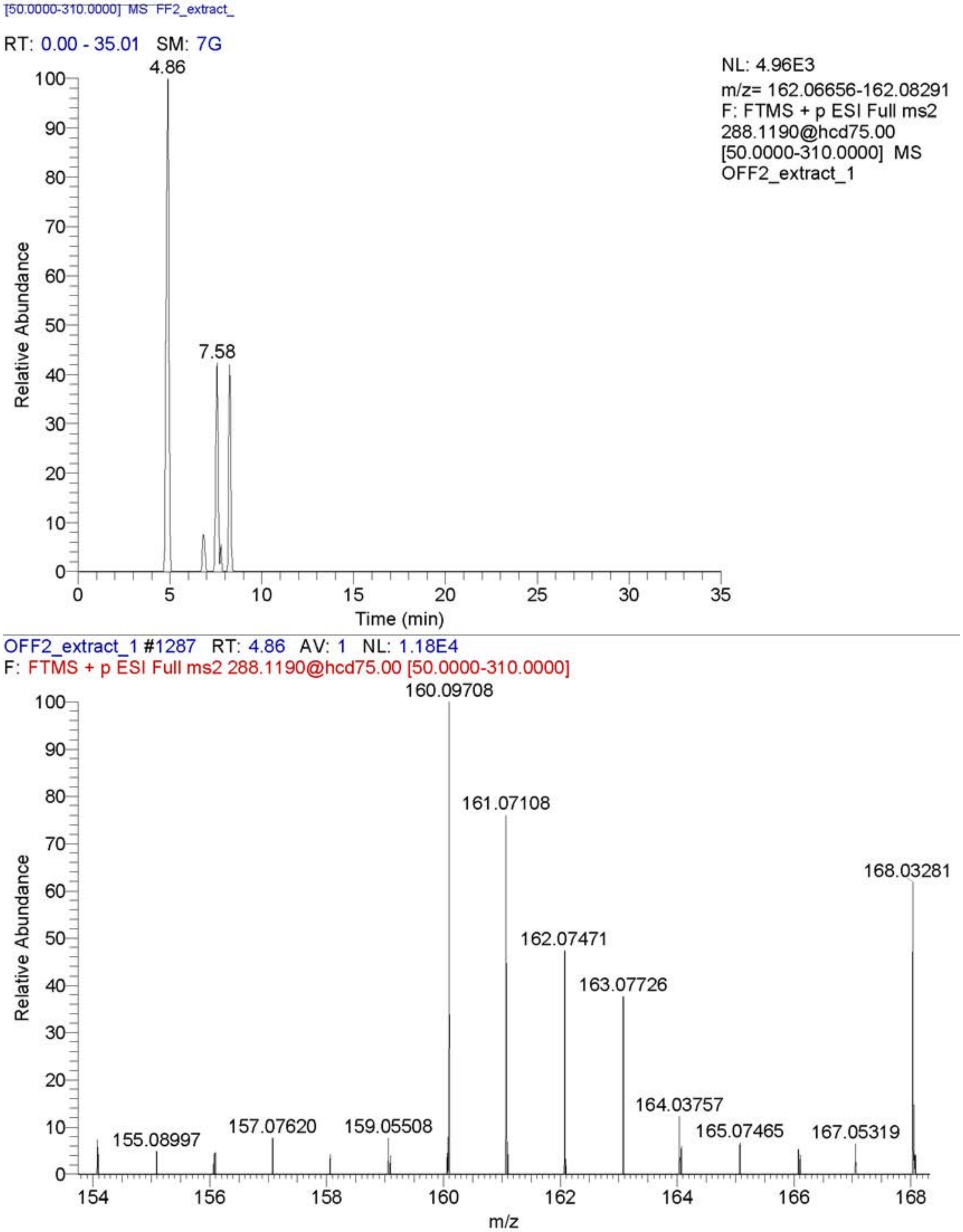
[50.0000-310.0000] MS FF2_extract_
RT: 0.00 - 35.01 SM: 7G
NL: 4.96E3
m/z= 162.06656-162.08291
F: FTMS + p ESI Full ms2
288.1190@hcd75.00
[50.0000-310.0000] MS
OFF2_extract_1
4.86
7.58
Relative Abundance
Time (min)
OFF2_extract_1 #1287 RT: 4.86 AV: 1 NL: 1.18E4
F: FTMS + p ESI Full ms2 288.1190@hcd75.00 [50.0000-310.0000]
160.09708
161.07108
168.03281
162.07471
163.07726
164.03757
155.08997
157.07620
159.05508
165.07465
167.05319
Relative Abundance
m/z

S. mcgrathi

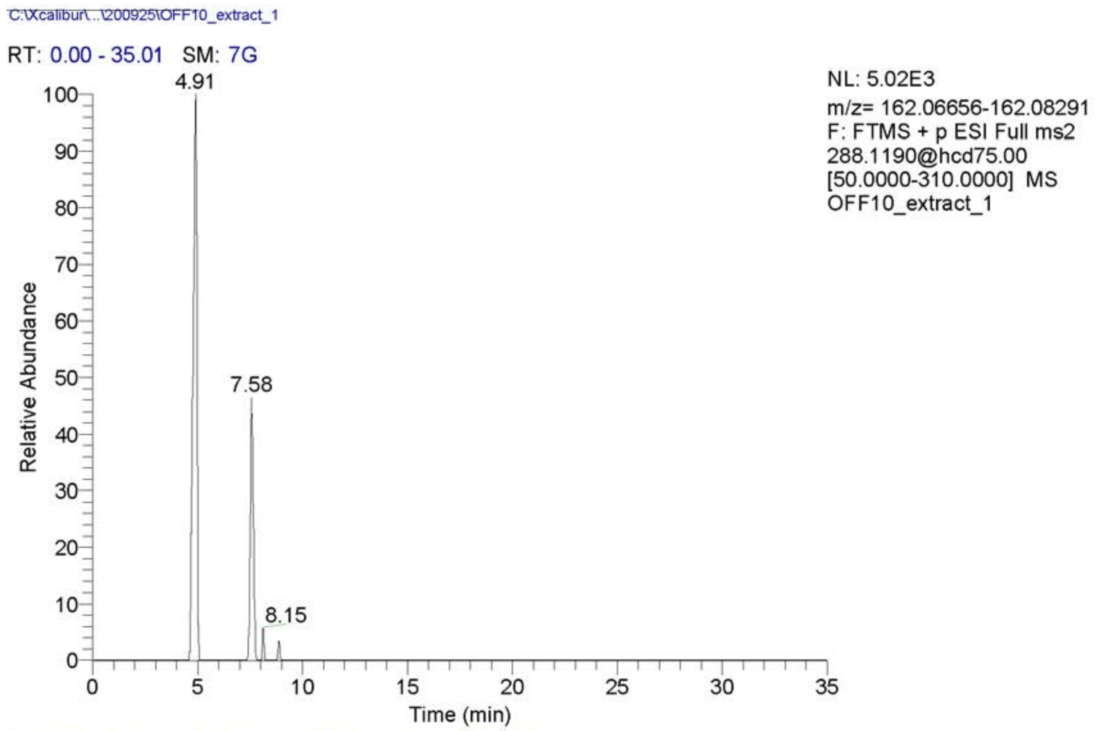
C:\Xcalibur\...\200925\OFF10_extract_1
RT: 0.00 - 35.01 SM: 7G
NL: 5.02E3
m/z= 162.06656-162.08291
F: FTMS + p ESI Full ms2 288.1190@hcd75.00 [50.0000-310.0000] MS
OFF10_extract_1
4.91
7.58
8.15
Relative Abundance
Time (min)

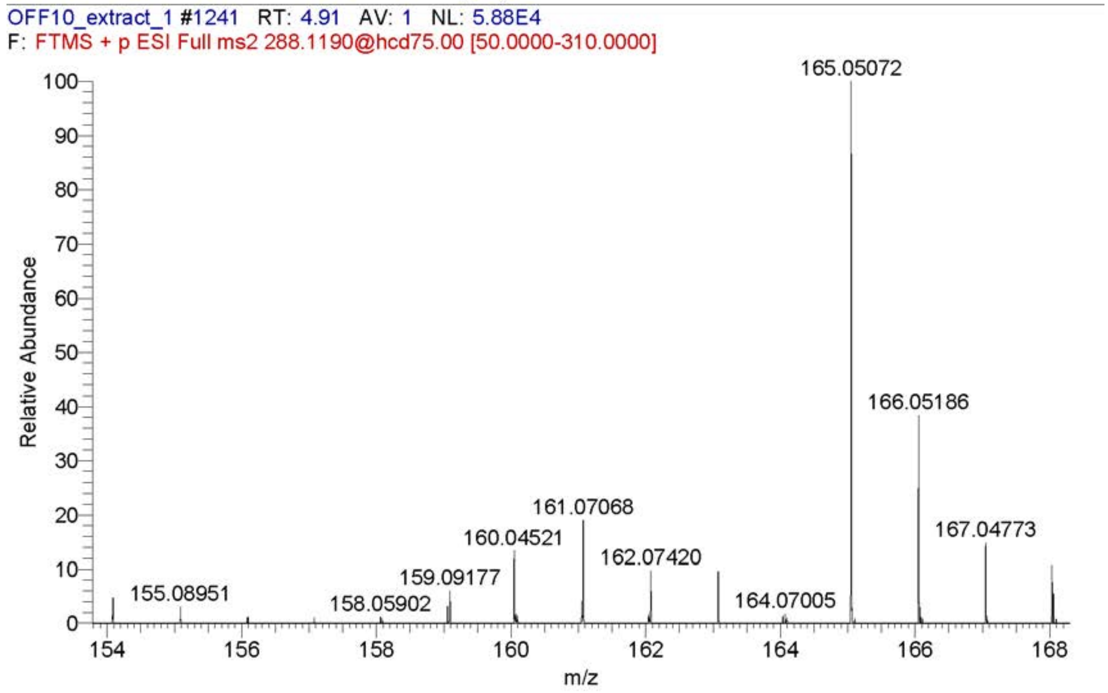
OFF10_extract_1 #1241 RT: 4.91 AV: 1 NL: 5.88E4
F: FTMS + p ESI Full ms2 288.1190@hcd75.00 [50.0000-310.0000]
165.05072
166.05186
167.04773
161.07068
160.04521
162.07420
159.09177
155.08951
158.05902
164.07005
Relative Abundance
m/z

S. mcgrathi

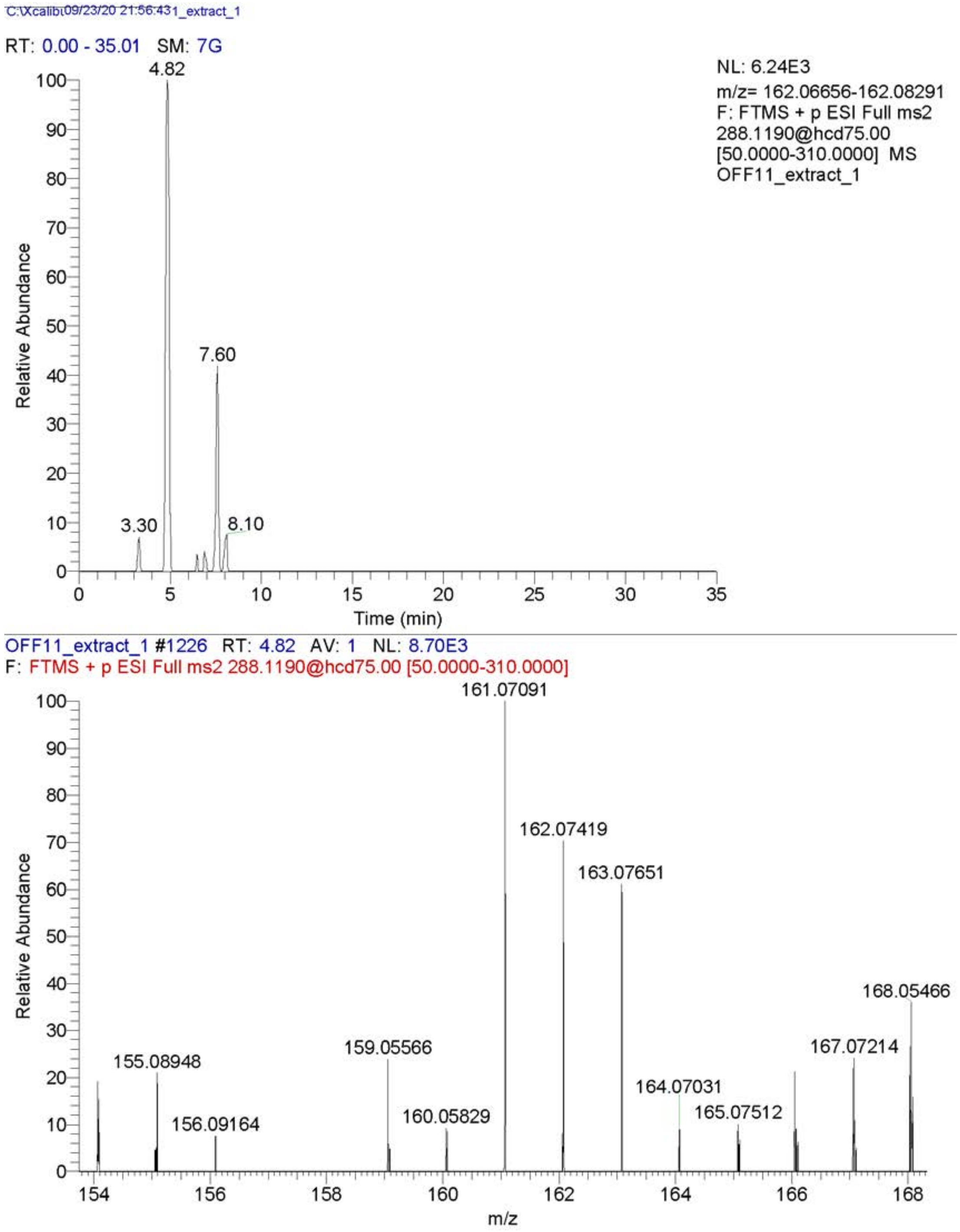
C:\Xcalibι09/23/20 21:56:431_extract_1
RT: 0.00 - 35.01 SM: 7G
NL: 6.24E3
m/z= 162.06656-162.08291
F: FTMS + p ESI Full ms2
288.1190@hcd75.00
[50.0000-310.0000] MS
OFF11_extract_1
4.82
7.60
3.30
8.10
Relative Abundance
Time (min)
OFF11_extract_1 #1226 RT: 4.82 AV: 1 NL: 8.70E3
F: FTMS + p ESI Full ms2 288.1190@hcd75.00 [50.0000-310.0000]
161.07091
162.07419
163.07651
168.05466
159.05566
167.07214
155.08948
164.07031
165.07512
160.05829
156.09164
m/z

S. mcgrathi

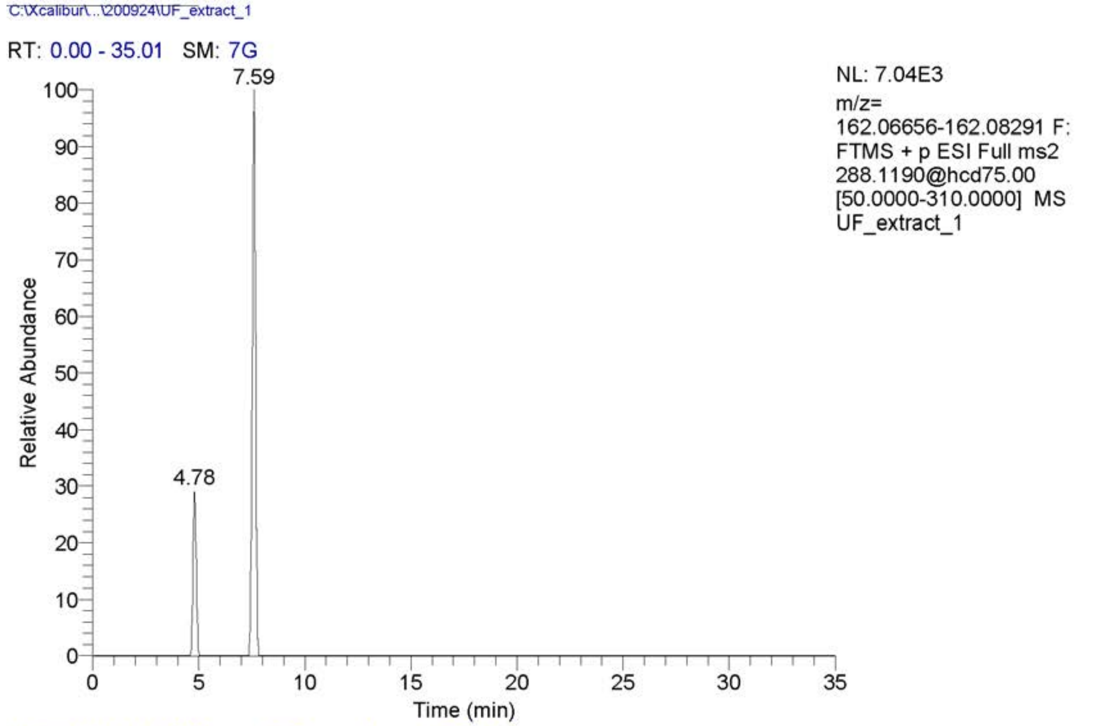

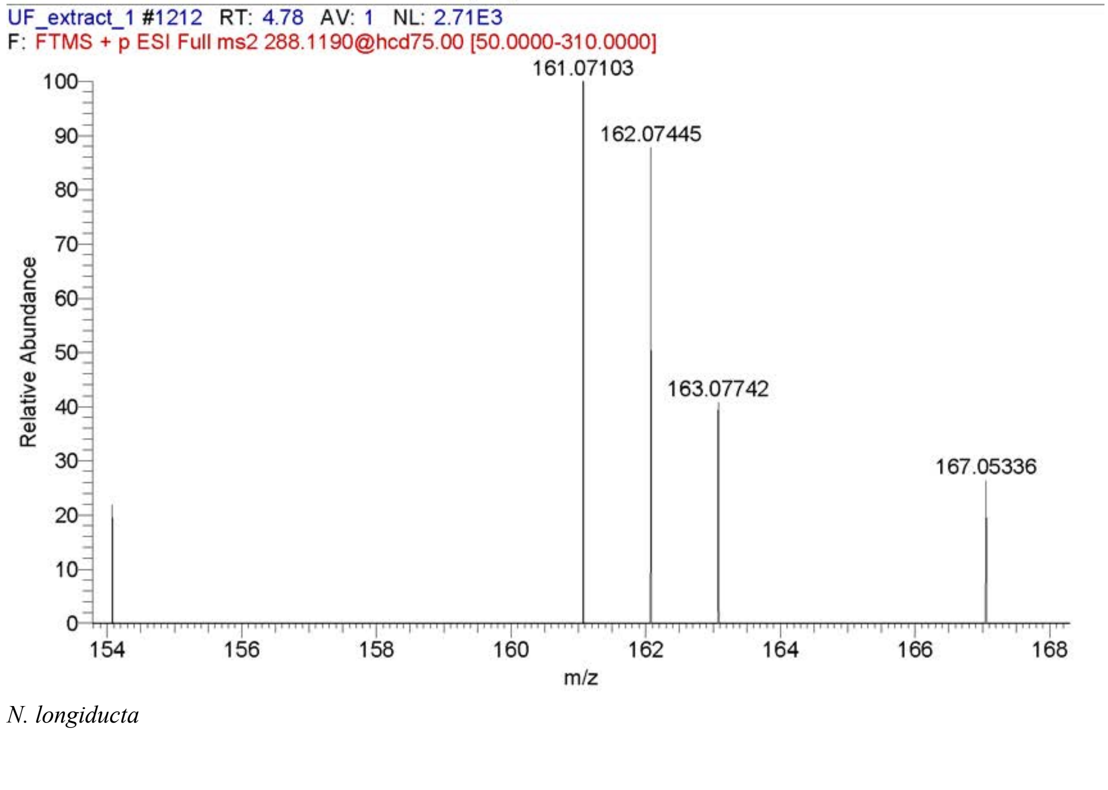

N. longiducta

unknown compound #1 m/z 270.1085

Samples were determined to contain this analyte when the daughter ion 162.07 was observed at a similar time in each sample. These daughter ions were taken from Bane, Lehane, Dikshit, O'Riordan and Furey [1]. The samples that were determined to contain this analyte were *T. brocchii* (RT: 8.58), *T. brocchii* (RT: 8.48), *S. mcgrathi* (RT: 8.44), *S. mcgrathi* (RT: 8.59), *S. mcgrathi* (RT: 8.61), *S. mcgrathi* (RT: 8.56), *S. mcgrathi* (RT: 8.47), *S. mcgrathi* (RT: 8.60), *S. mcgrathi* (RT: 8.58), *S. mcgrathi* (RT: 8.70), *P. velutinus* (RT: 8.60), *Pseudoceros sp. 1* (RT: 8.49), *Pseudoceros sp. 3* (RT: 8.48)

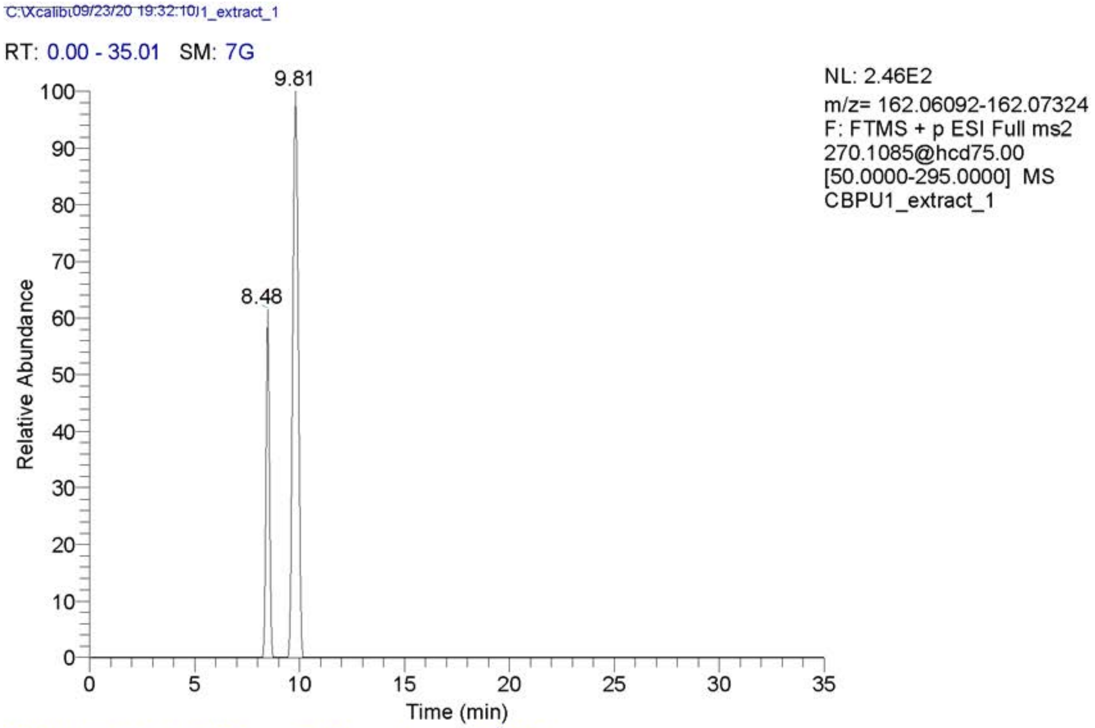

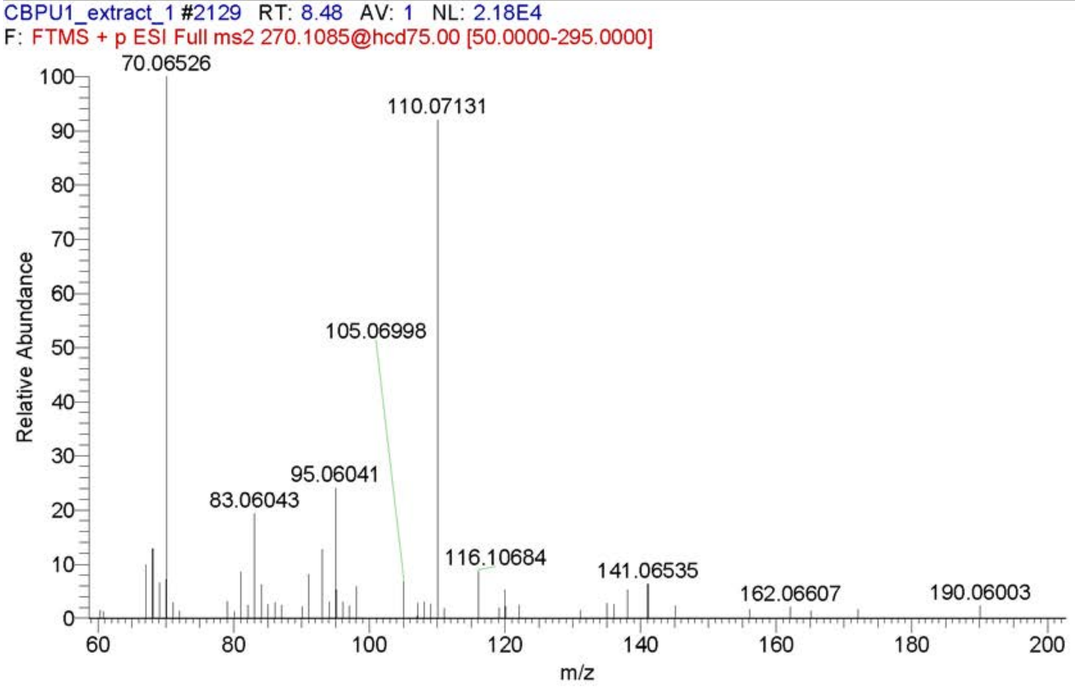

Pseudoceros sp. 3

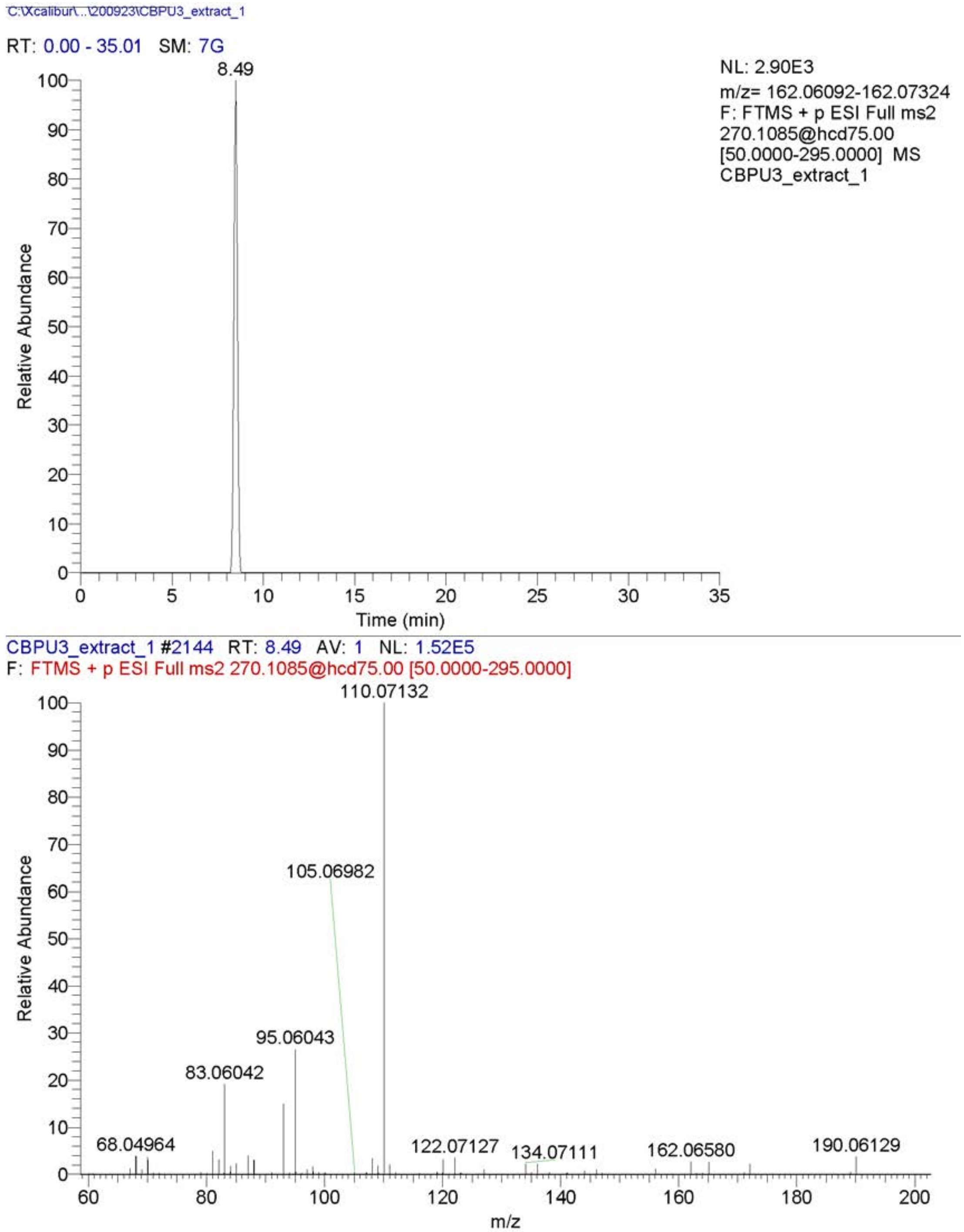

Pseudoceros sp. 1

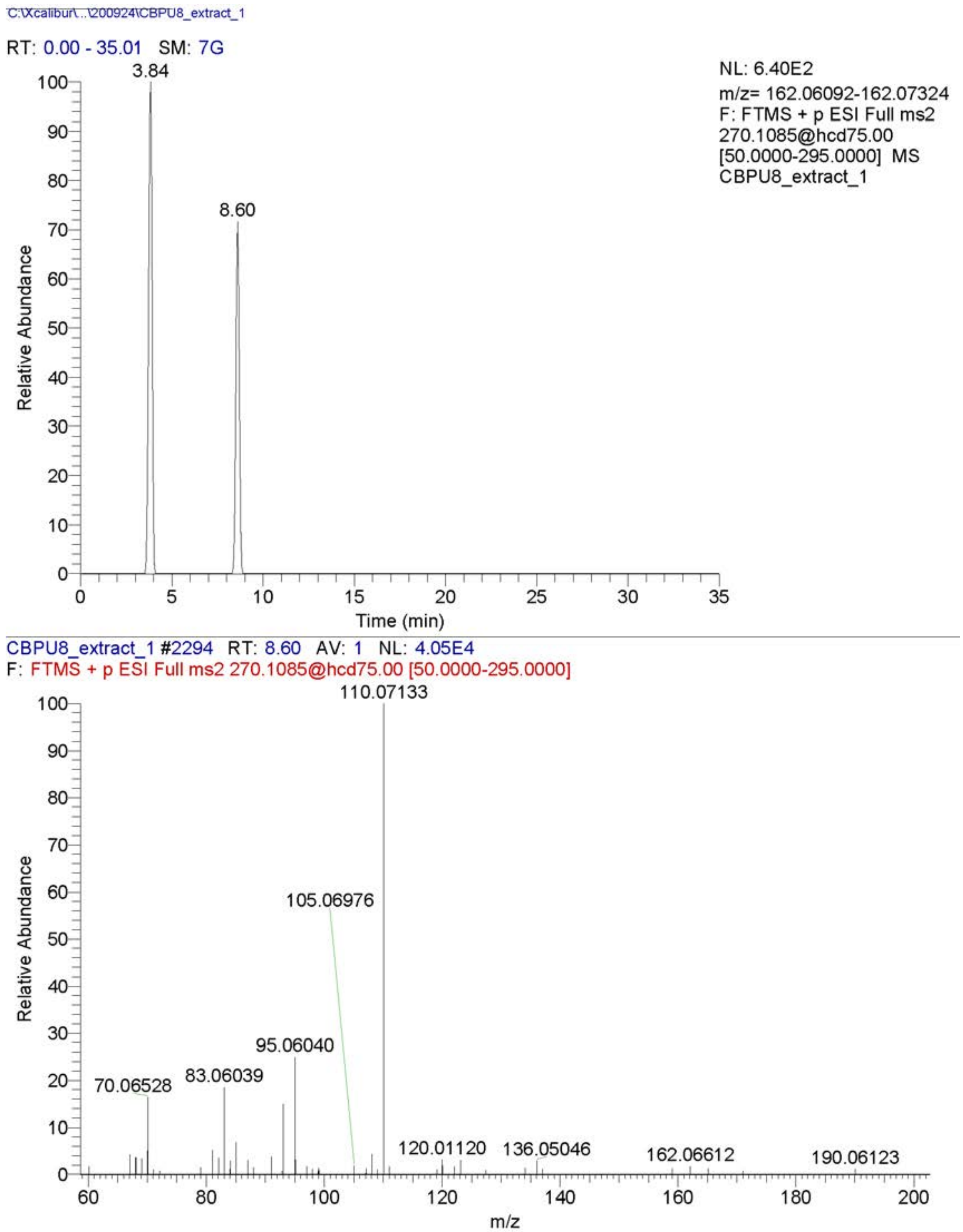
C:\Xcalibur\...\200924\CBPU8_extract_1
RT: 0.00 - 35.01 SM: 7G
3.84
8.60
NL: 6.40E2
m/z= 162.06092-162.07324
F: FTMS + p ESI Full ms2
270.1085@hcd75.00
[50.0000-295.0000] MS
CBPU8_extract_1
Relative Abundance
Time (min)
CBPU8_extract_1 #2294 RT: 8.60 AV: 1 NL: 4.05E4
F: FTMS + p ESI Full ms2 270.1085@hcd75.00 [50.0000-295.0000]
110.07133
105.06976
95.06040
83.06039
70.06528
120.01120
136.05046
162.06612
190.06123
Relative Abundance
m/z

P. velutinus

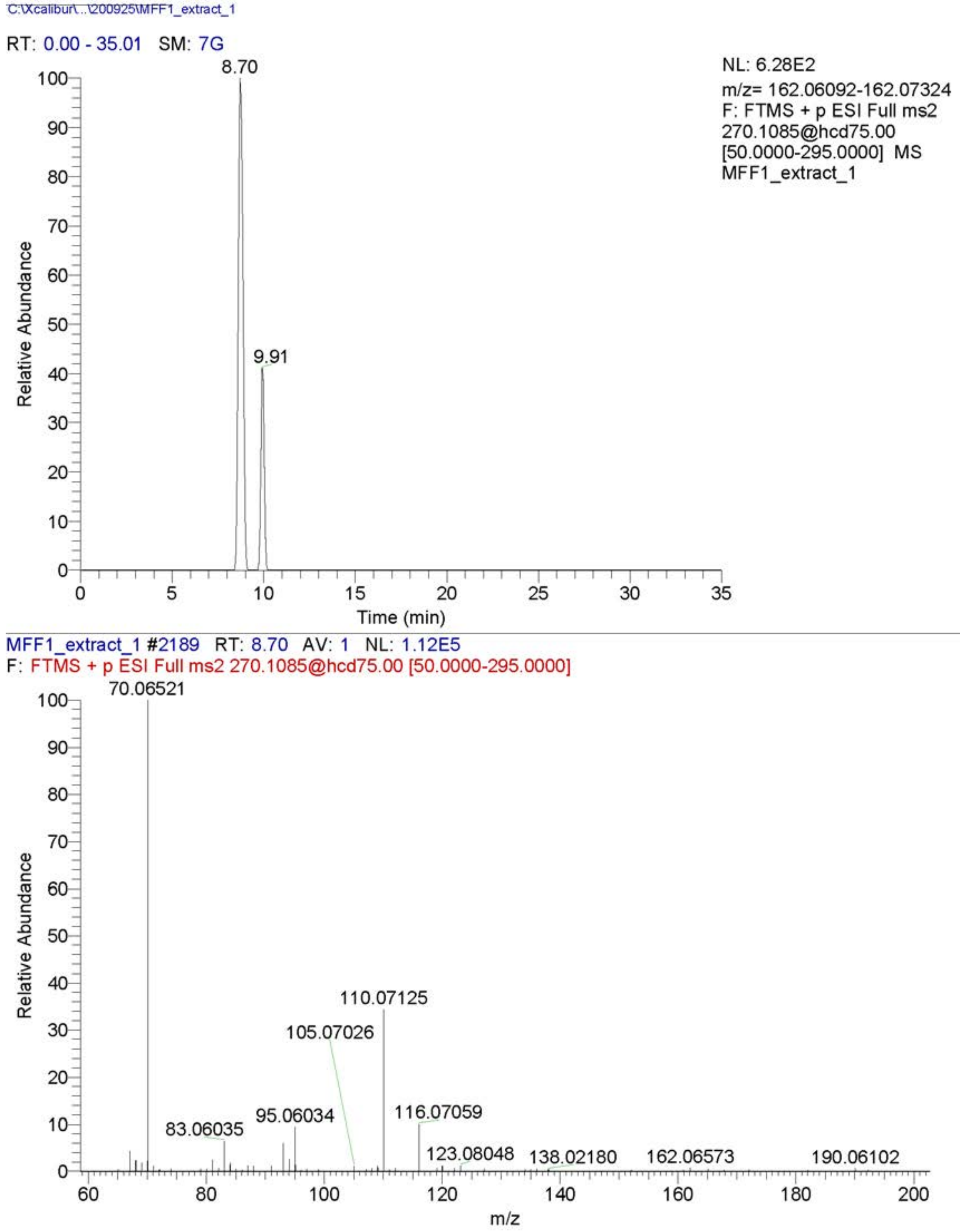
C:\Xcalibur\...\200925\MFF1_extract_1
RT: 0.00 - 35.01 SM: 7G
NL: 6.28E2
m/z= 162.06092-162.07324
F: FTMS + p ESI Full ms2
270.1085@hcd75.00
[50.0000-295.0000] MS
MFF1_extract_1
8.70
9.91
Relative Abundance
Time (min)
MFF1_extract_1 #2189 RT: 8.70 AV: 1 NL: 1.12E5
F: FTMS + p ESI Full ms2 270.1085@hcd75.00 [50.0000-295.0000]
70.06521
110.07125
105.07026
95.06034
83.06035
116.07059
123.08048
138.02180
162.06573
190.06102
m/z

S. mcgrathi

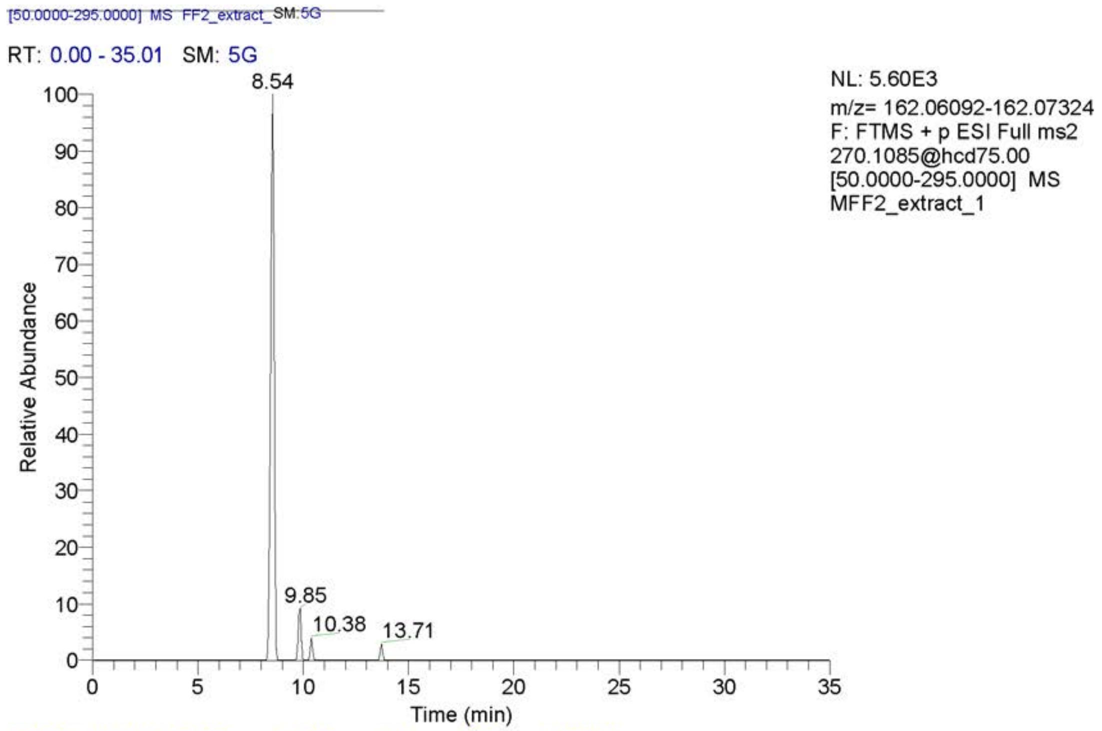
[50.0000-295.0000] MS FF2_extract_SM:5G
RT: 0.00 - 35.01 SM: 5G
NL: 5.60E3
m/z= 162.06092-162.07324
F: FTMS + p ESI Full ms2
270.1085@hcd75.00
[50.0000-295.0000] MS
MFF2_extract_1
8.54
9.85
10.38
13.71
Relative Abundance
Time (min)

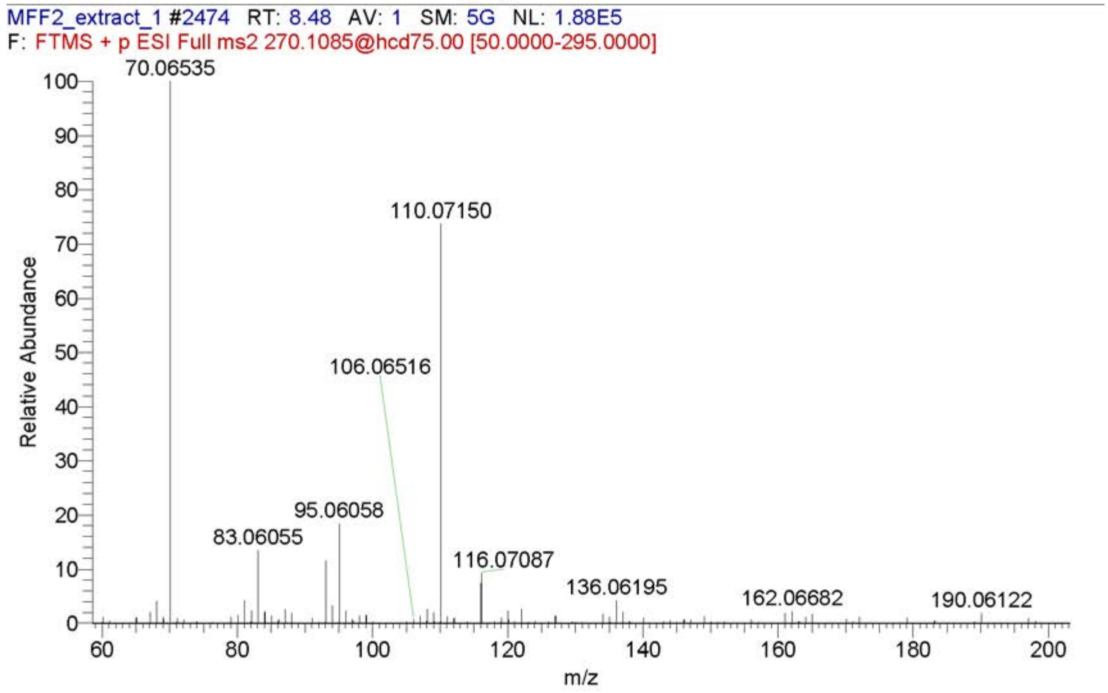
MFF2_extract_1 #2474 RT: 8.48 AV: 1 SM: 5G NL: 1.88E5
F: FTMS + p ESI Full ms2 270.1085@hcd75.00 [50.0000-295.0000]
70.06535
110.07150
106.06516
95.06058
83.06055
116.07087
136.06195
162.06682
190.06122
Relative Abundance
m/z

S. mcgrathi

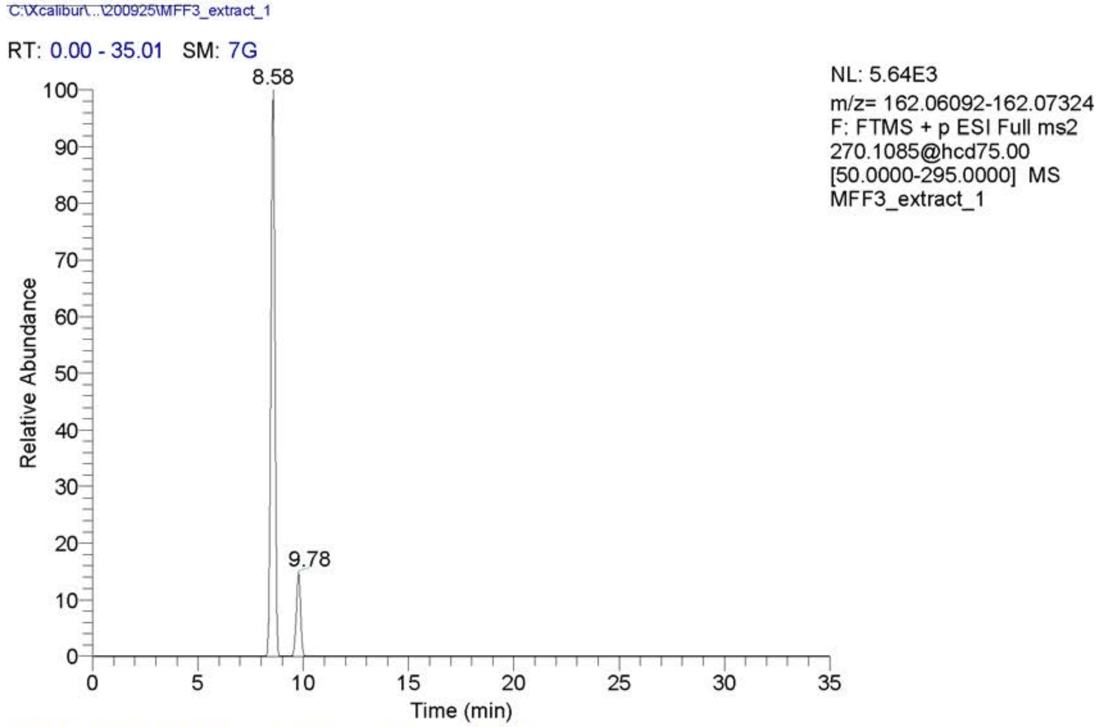
C:\Xcalibur\...\200925\MFF3_extract_1
RT: 0.00 - 35.01 SM: 7G
8.58
9.78
NL: 5.64E3
m/z= 162.06092-162.07324
F: FTMS + p ESI Full ms2
270.1085@hcd75.00
[50.0000-295.0000] MS
MFF3_extract_1
Relative Abundance
Time (min)

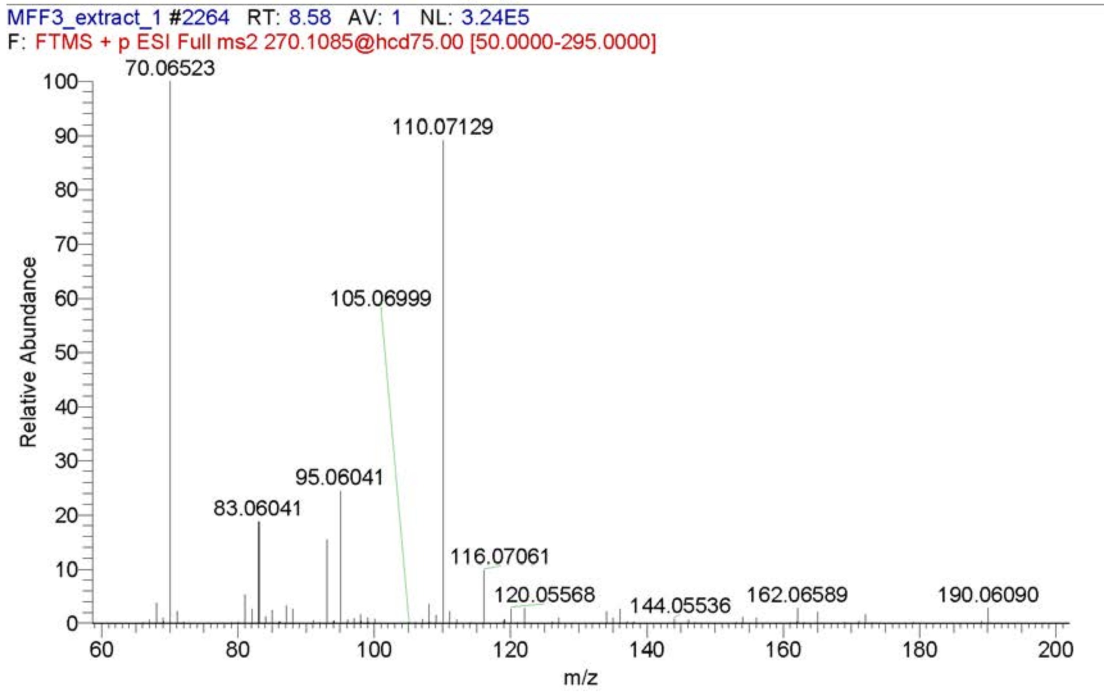
MFF3_extract_1 #2264 RT: 8.58 AV: 1 NL: 3.24E5
F: FTMS + p ESI Full ms2 270.1085@hcd75.00 [50.0000-295.0000]
70.06523
110.07129
105.06999
95.06041
83.06041
116.07061
120.05568
144.05536
162.06589
190.06090
Relative Abundance
m/z

S. mcgrathi

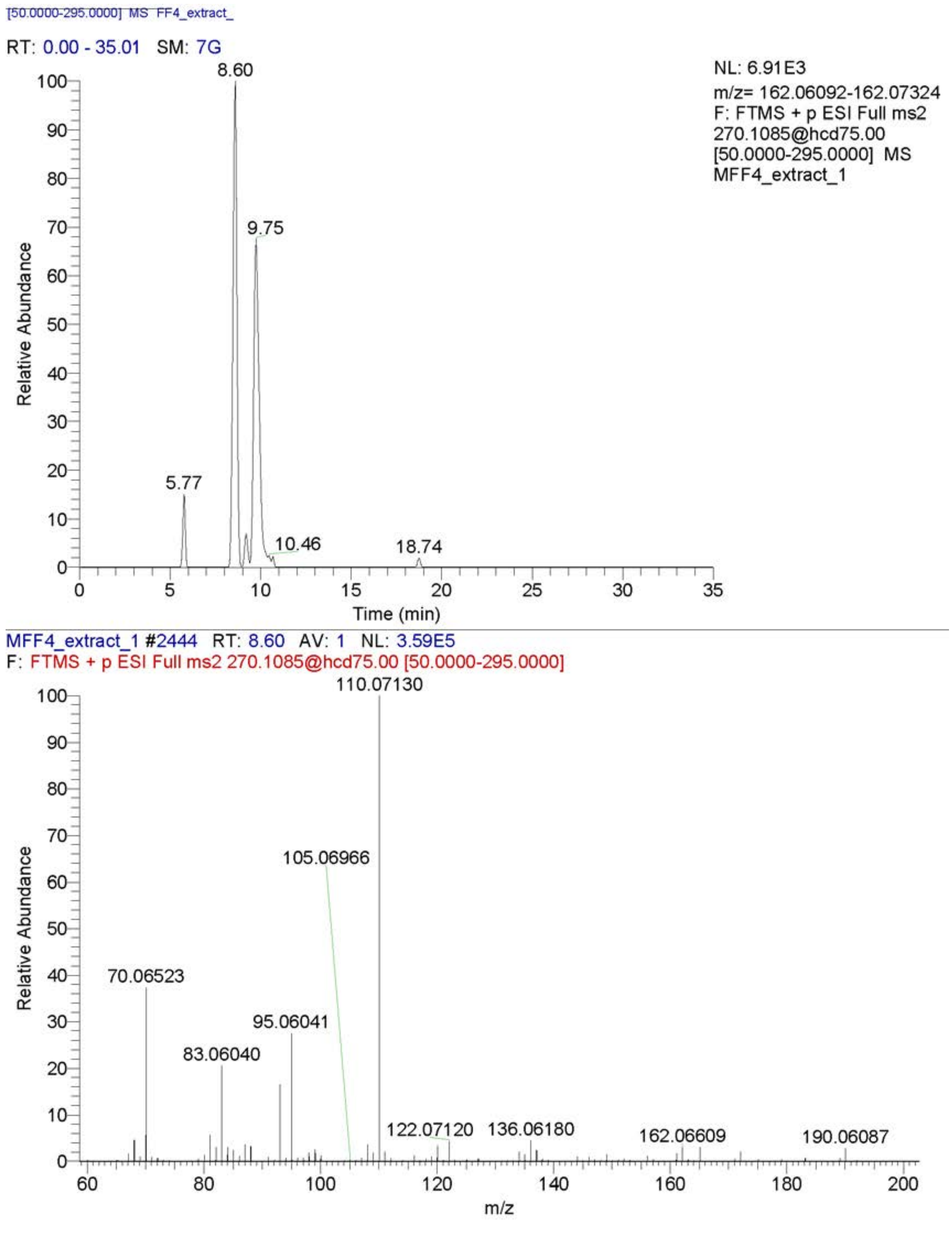
[50.0000-295.0000] MS FF4_extract_
RT: 0.00 - 35.01 SM: 7G
NL: 6.91E3
m/z= 162.06092-162.07324
F: FTMS + p ESI Full ms2 270.1085@hcd75.00 [50.0000-295.0000] MS MFF4_extract_1
8.60
9.75
5.77
10.46
18.74
Relative Abundance
Time (min)
MFF4_extract_1 #2444 RT: 8.60 AV: 1 NL: 3.59E5
F: FTMS + p ESI Full ms2 270.1085@hcd75.00 [50.0000-295.0000]
110.07130
105.06966
70.06523
95.06041
83.06040
122.07120
136.06180
162.06609
190.06087
m/z

S. mcgrathi

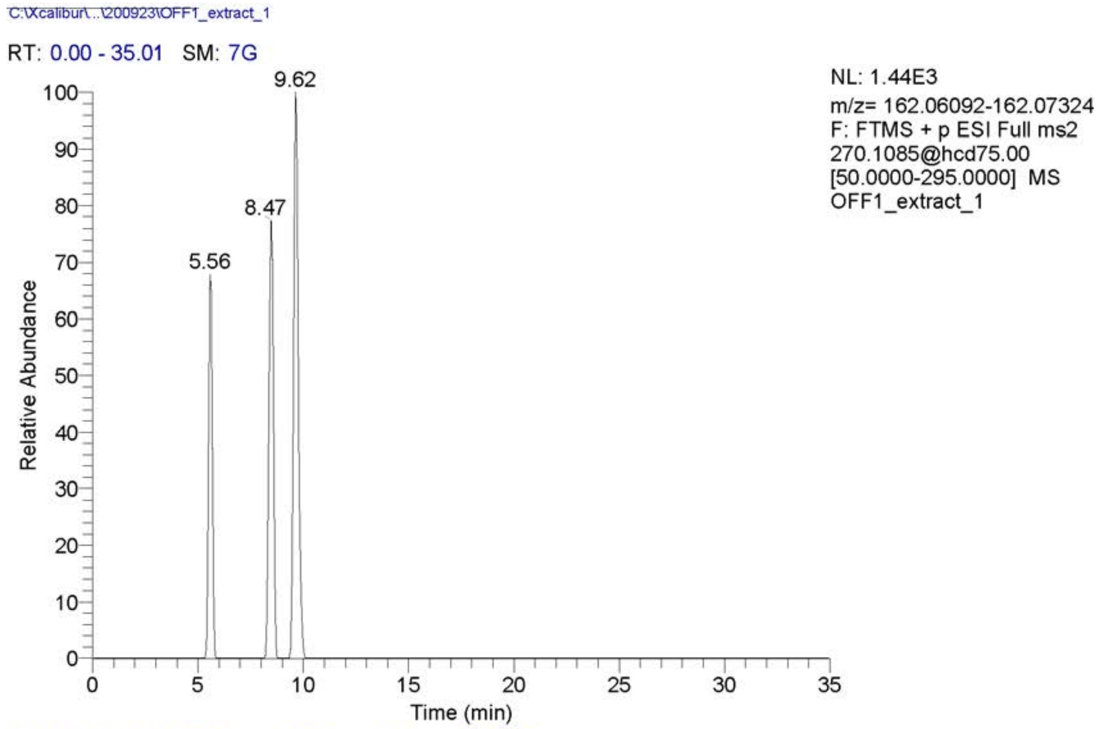
C:\Xcalibur\...\200923\OFF1_extract_1
RT: 0.00 - 35.01 SM: 7G
NL: 1.44E3
m/z= 162.06092-162.07324
F: FTMS + p ESI Full ms2
270.1085@hcd75.00
[50.0000-295.0000] MS
OFF1_extract_1
9.62
8.47
5.56
Relative Abundance
Time (min)

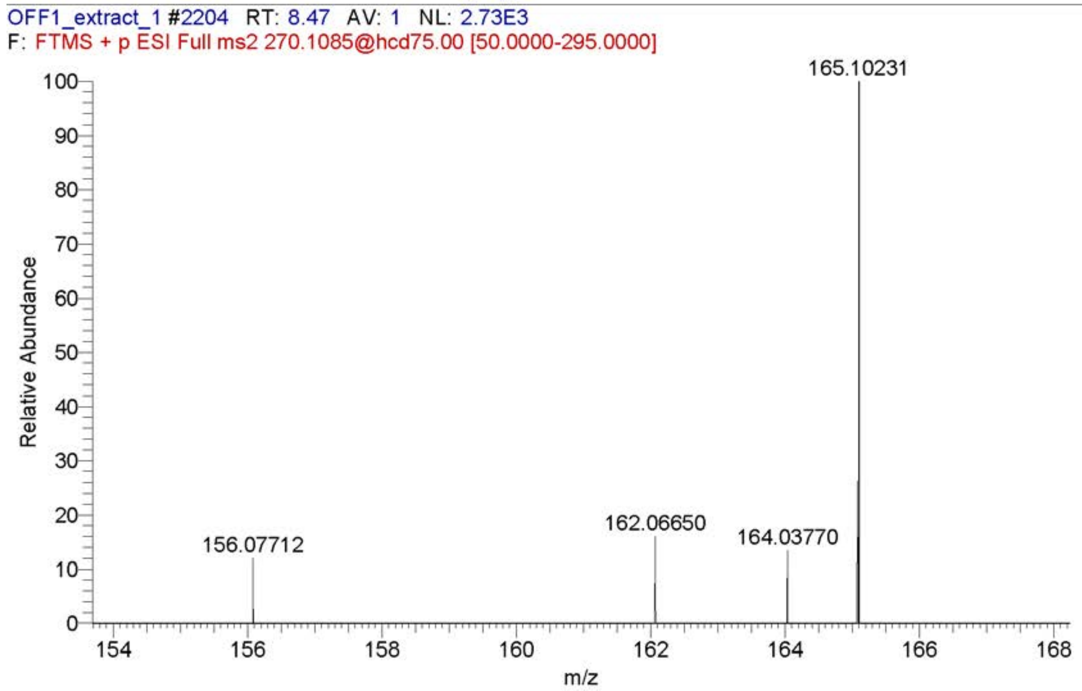
OFF1_extract_1 #2204 RT: 8.47 AV: 1 NL: 2.73E3
F: FTMS + p ESI Full ms2 270.1085@hcd75.00 [50.0000-295.0000]
165.10231
162.06650
164.03770
156.07712
Relative Abundance
m/z

S. mcgrathi

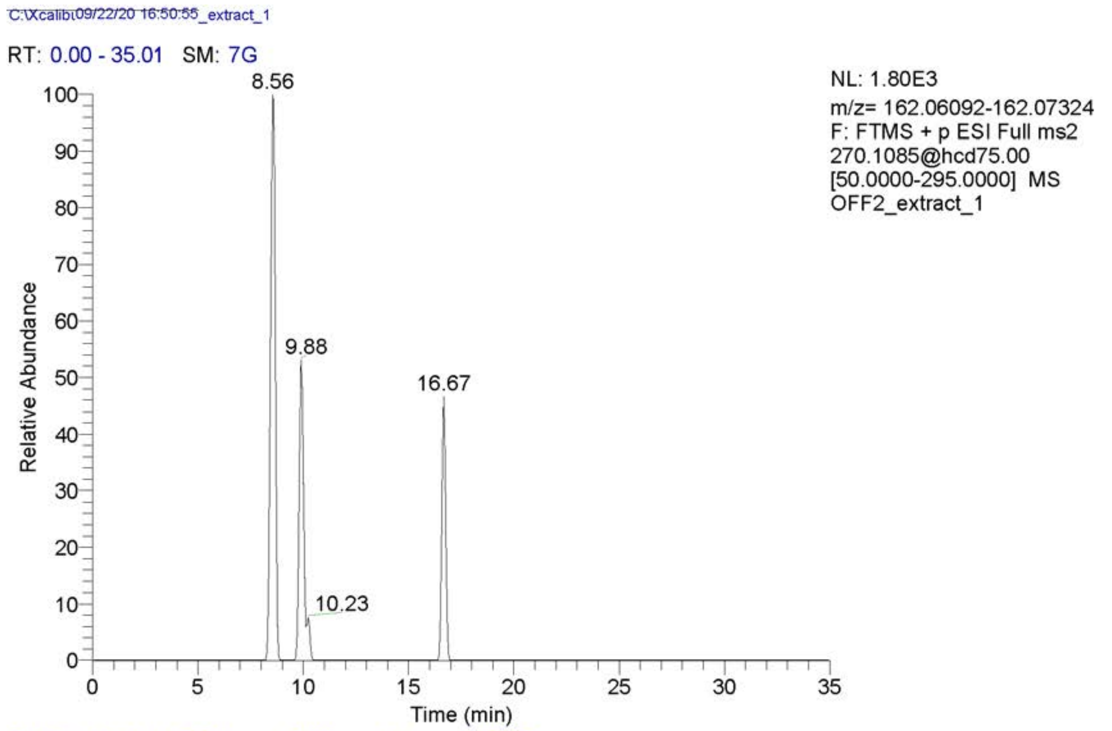
C:\Xcalibu09/22/20 16:50:55_extract_1
RT: 0.00 - 35.01 SM: 7G
NL: 1.80E3
m/z= 162.06092-162.07324
F: FTMS + p ESI Full ms2
270.1085@hcd75.00
[50.0000-295.0000] MS
OFF2_extract_1
8.56
9.88
10.23
16.67
Relative Abundance
Time (min)

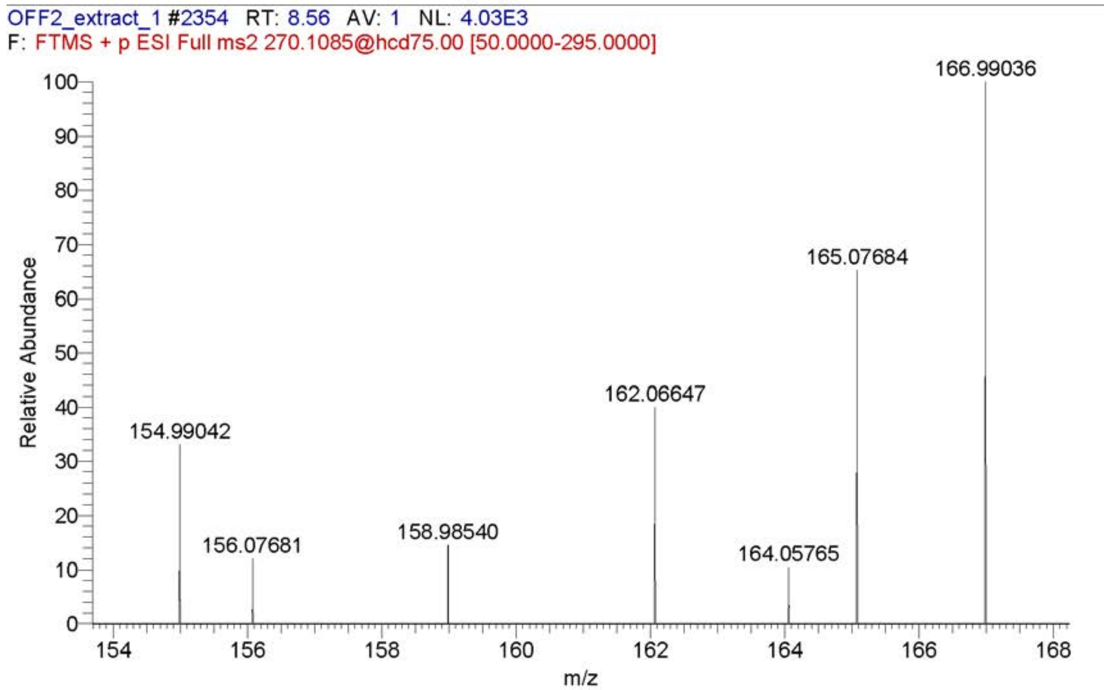
OFF2_extract_1 #2354 RT: 8.56 AV: 1 NL: 4.03E3
F: FTMS + p ESI Full ms2 270.1085@hcd75.00 [50.0000-295.0000]
166.99036
165.07684
162.06647
154.99042
156.07681
158.98540
164.05765
Relative Abundance
m/z

S. mcgrathi

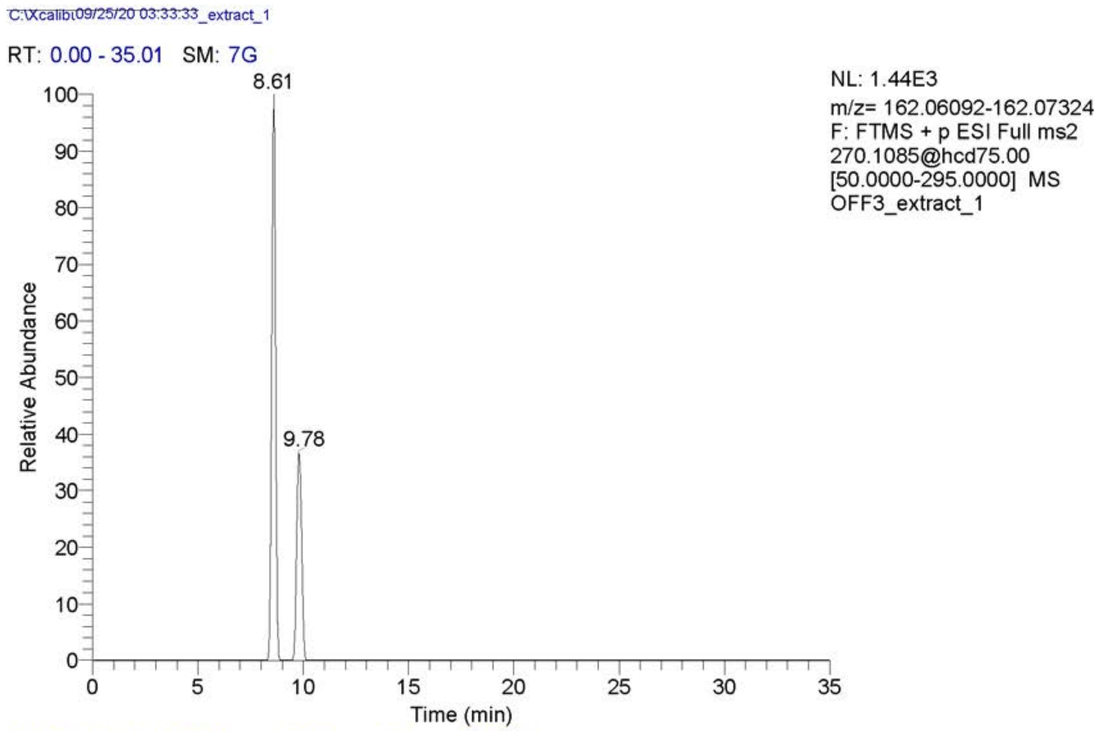
C:\Xcalibι09/25/20 03:33:33_extract_1
RT: 0.00 - 35.01 SM: 7G
NL: 1.44E3
m/z= 162.06092-162.07324
F: FTMS + p ESI Full ms2
270.1085@hcd75.00
[50.0000-295.0000] MS
OFF3_extract_1
8.61
9.78
Relative Abundance
Time (min)

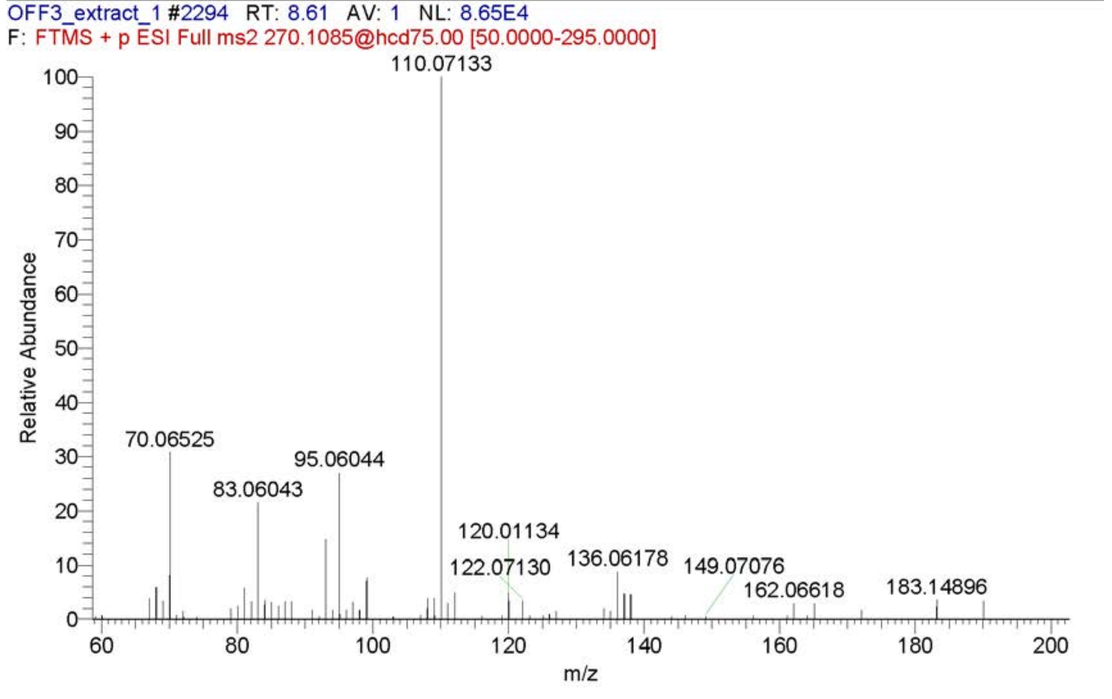
OFF3_extract_1 #2294 RT: 8.61 AV: 1 NL: 8.65E4
F: FTMS + p ESI Full ms2 270.1085@hcd75.00 [50.0000-295.0000]
110.07133
70.06525
83.06043
95.06044
120.01134
122.07130
136.06178
149.07076
162.06618
183.14896
Relative Abundance
m/z

S. mcgrathi

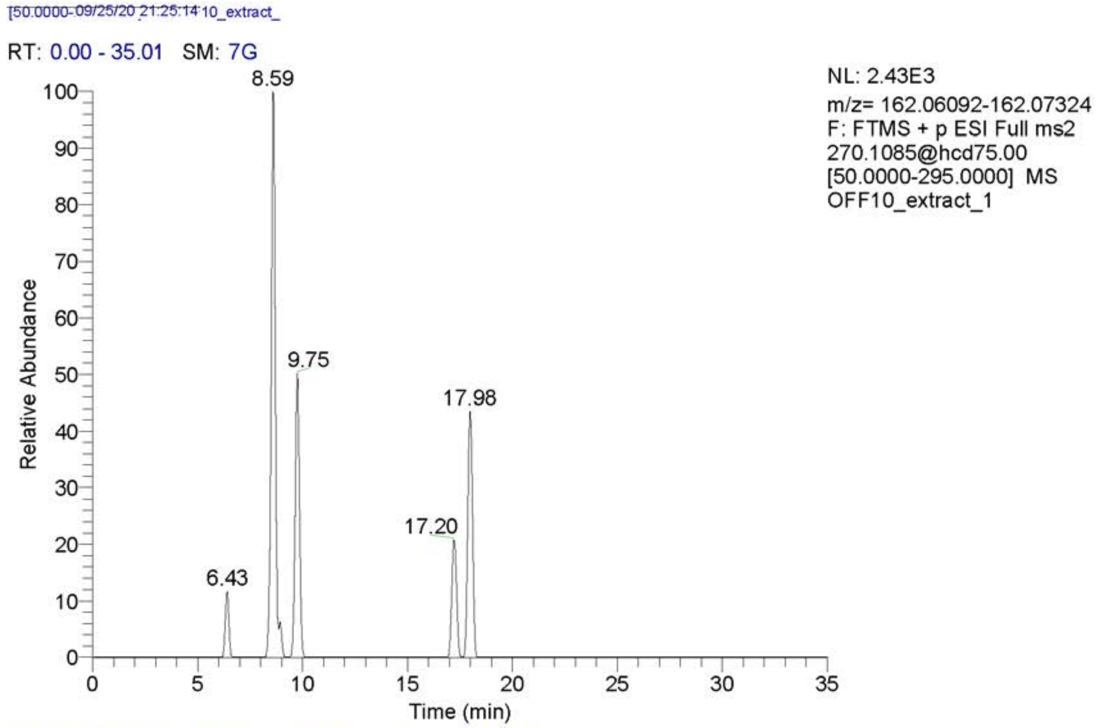

[50.0000-09/25/20 21:25:14 10_extract_
RT: 0.00 - 35.01 SM: 7G
NL: 2.43E3
m/z= 162.06092-162.07324
F: FTMS + p ESI Full ms2 270.1085@hcd75.00 [50.0000-295.0000] MS OFF10_extract_1
8.59
9.75
17.98
17.20
6.43
Relative Abundance
Time (min)

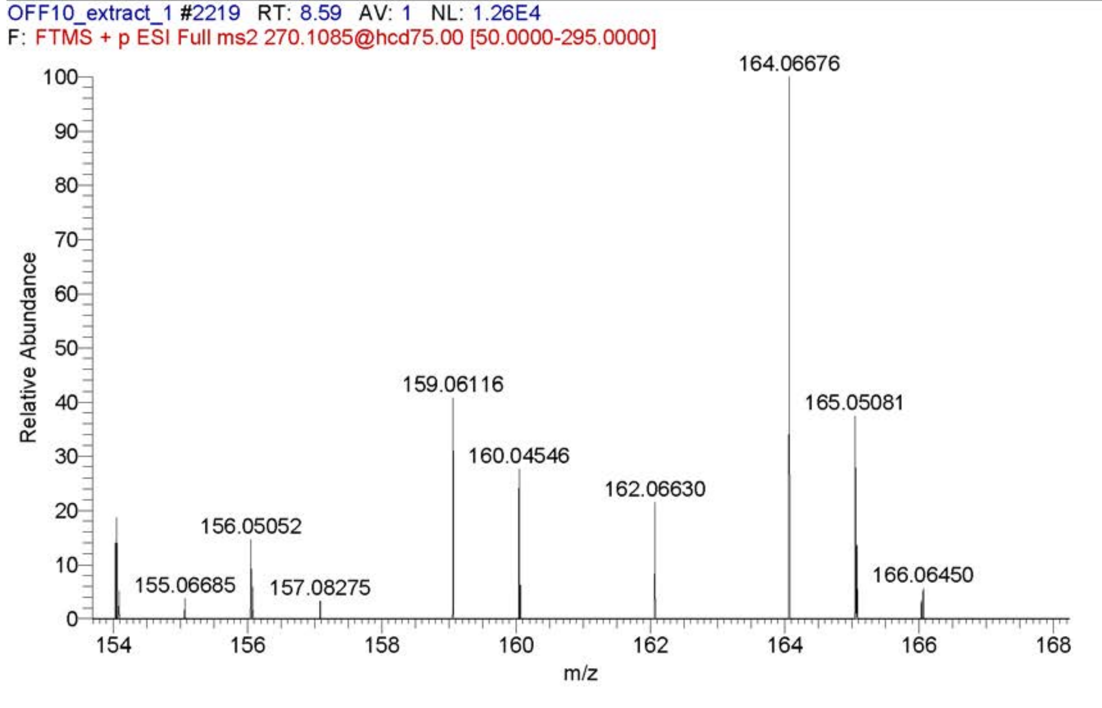

OFF10_extract_1 #2219 RT: 8.59 AV: 1 NL: 1.26E4
F: FTMS + p ESI Full ms2 270.1085@hcd75.00 [50.0000-295.0000]
164.06676
159.06116
165.05081
160.04546
162.06630
156.05052
155.06685
157.08275
166.06450
Relative Abundance
m/z

S. mcgrathi

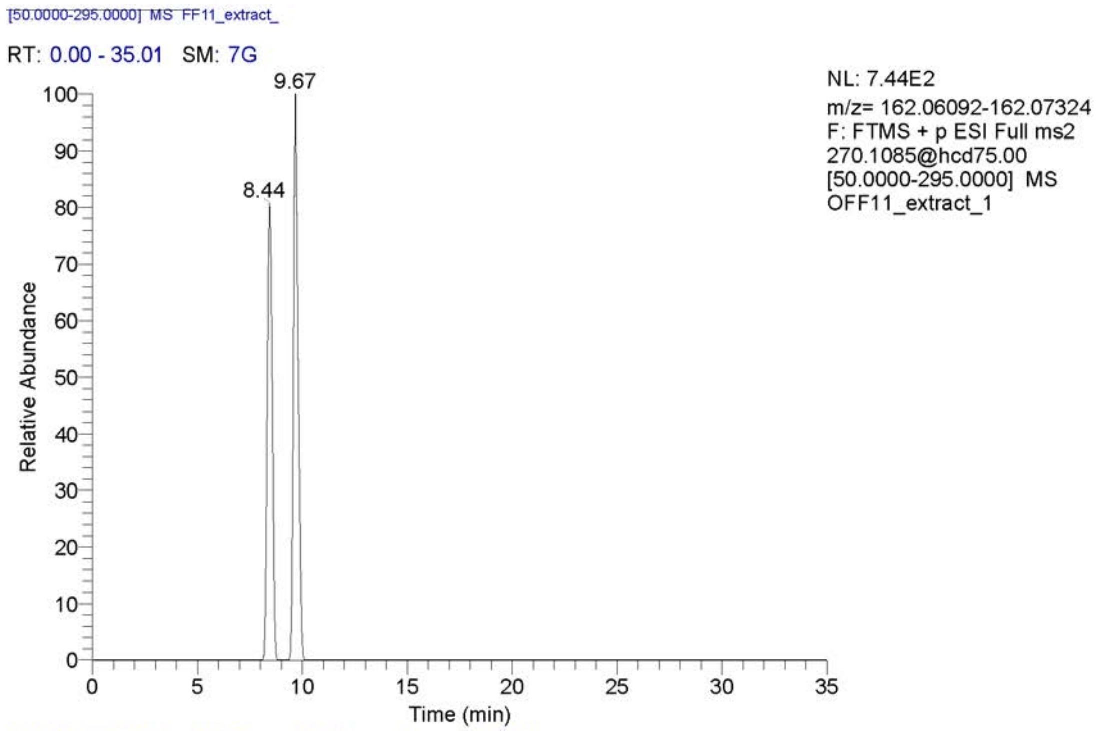
[50.0000-295.0000] MS FF11_extract_
RT: 0.00 - 35.01 SM: 7G
9.67
8.44
NL: 7.44E2
m/z= 162.06092-162.07324
F: FTMS + p ESI Full ms2
270.1085@hcd75.00
[50.0000-295.0000] MS
OFF11_extract_1
Relative Abundance
Time (min)

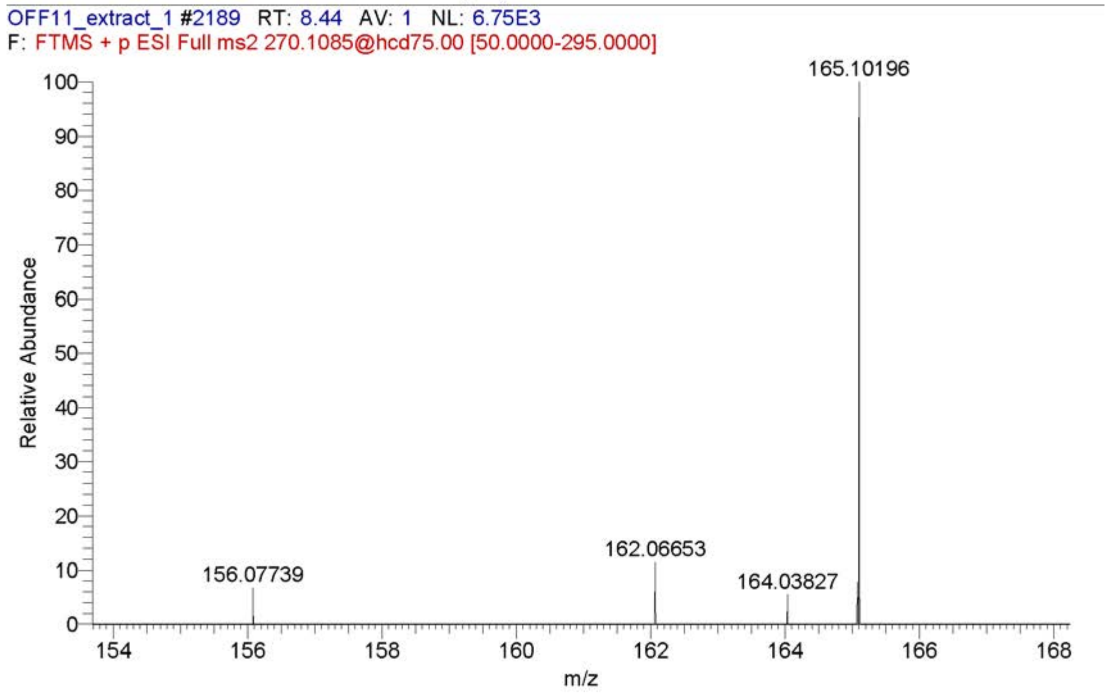
OFF11_extract_1 #2189 RT: 8.44 AV: 1 NL: 6.75E3
F: FTMS + p ESI Full ms2 270.1085@hcd75.00 [50.0000-295.0000]
165.10196
162.06653
156.07739
164.03827
Relative Abundance
m/z

S. mcgrathi

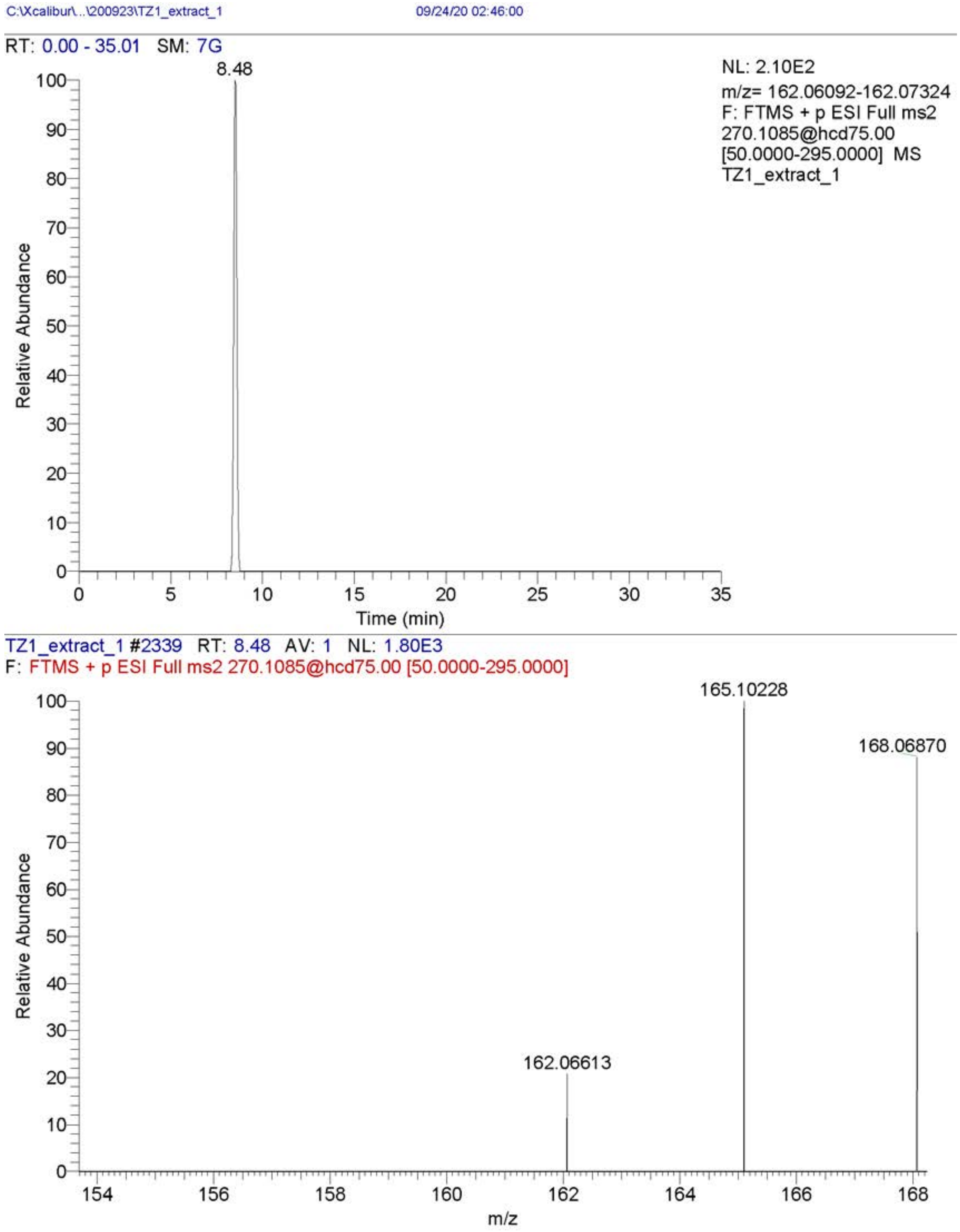
C:\Xcalibur\...\200923\TZ1_extract_1
09/24/20 02:46:00
RT: 0.00 - 35.01 SM: 7G
8.48
NL: 2.10E2
m/z= 162.06092-162.07324
F: FTMS + p ESI Full ms2
270.1085@hcd75.00
[50.0000-295.0000] MS
TZ1_extract_1
Relative Abundance
Time (min)
TZ1_extract_1 #2339 RT: 8.48 AV: 1 NL: 1.80E3
F: FTMS + p ESI Full ms2 270.1085@hcd75.00 [50.0000-295.0000]
165.10228
168.06870
162.06613
Relative Abundance
m/z

T. brocchii

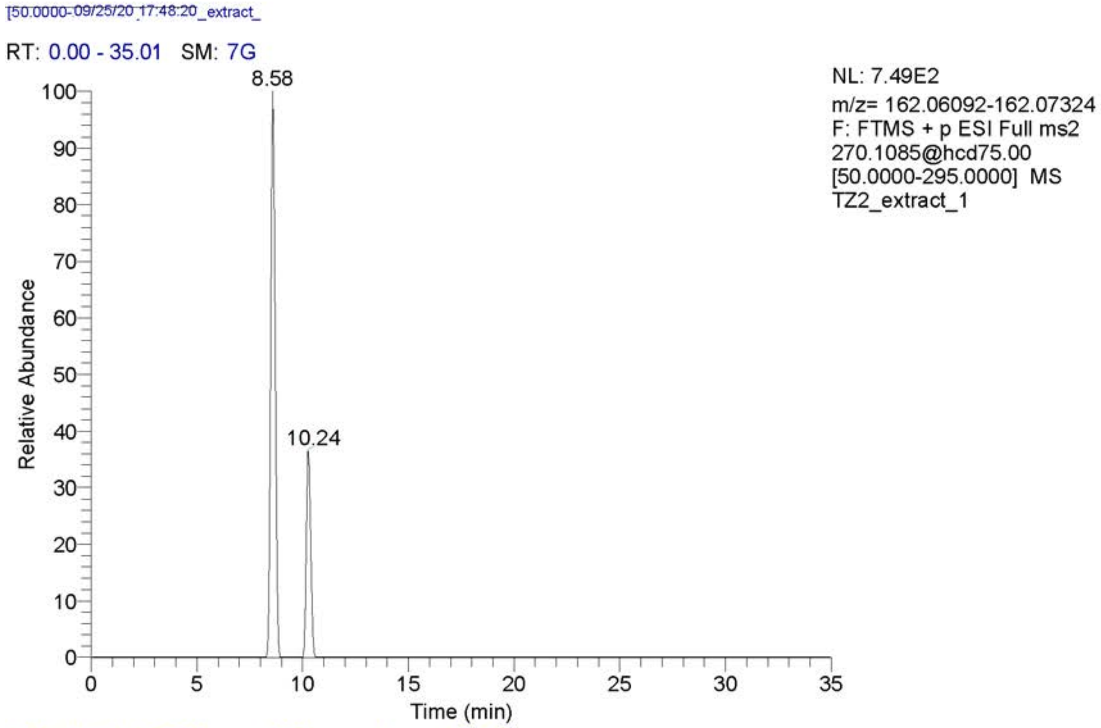
[50.0000-09/25/20 17:48:20_extract_
RT: 0.00 - 35.01 SM: 7G
NL: 7.49E2
m/z= 162.06092-162.07324
F: FTMS + p ESI Full ms2 270.1085@hcd75.00 [50.0000-295.0000] MS
TZ2_extract_1
8.58
10.24
Relative Abundance
Time (min)

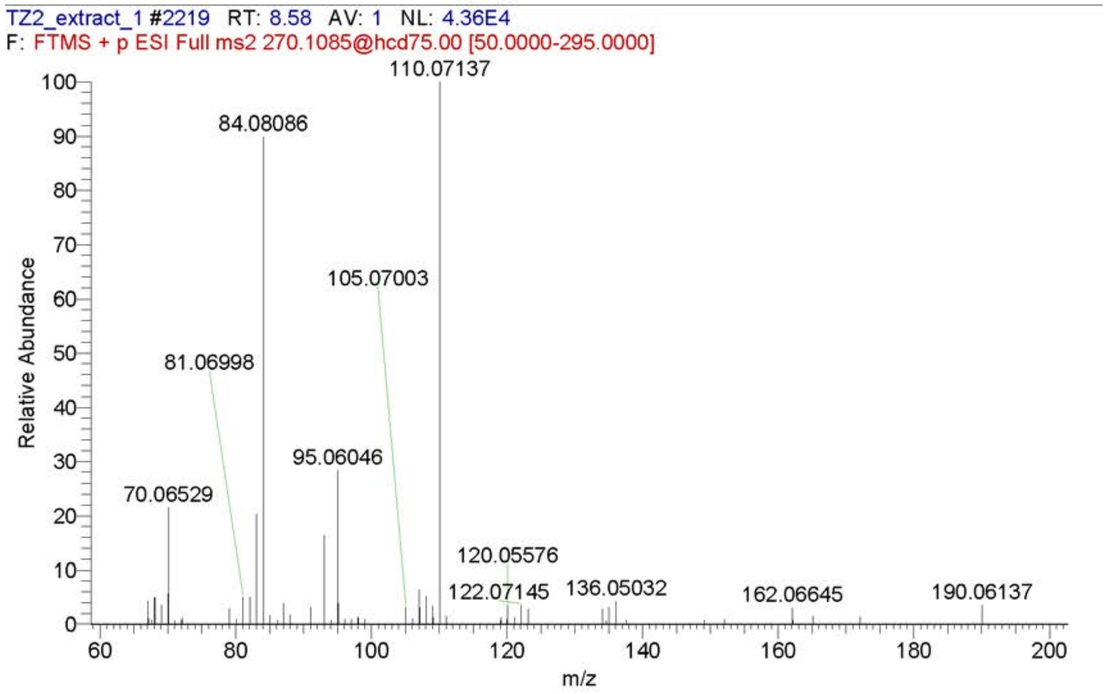
TZ2_extract_1 #2219 RT: 8.58 AV: 1 NL: 4.36E4
F: FTMS + p ESI Full ms2 270.1085@hcd75.00 [50.0000-295.0000]
110.07137
84.08086
105.07003
81.06998
95.06046
70.06529
120.05576
122.07145
136.05032
162.06645
190.06137
Relative Abundance
m/z

T. brocchii

4,4a-anhydro-5,6,11-trideoxyTTX (9) m/z 254.1135

Samples were determined to contain this analyte when the daughter ion 162.07 was observed at a similar time in each sample. These daughter ions were taken from Bane, Lehane, Dikshit, O'Riordan and Furey [1]. The samples that were determined to contain this analyte were, *S. mcgrathi* (RT: 8.01), *Eurylepta sp.* (RT: 8.03).

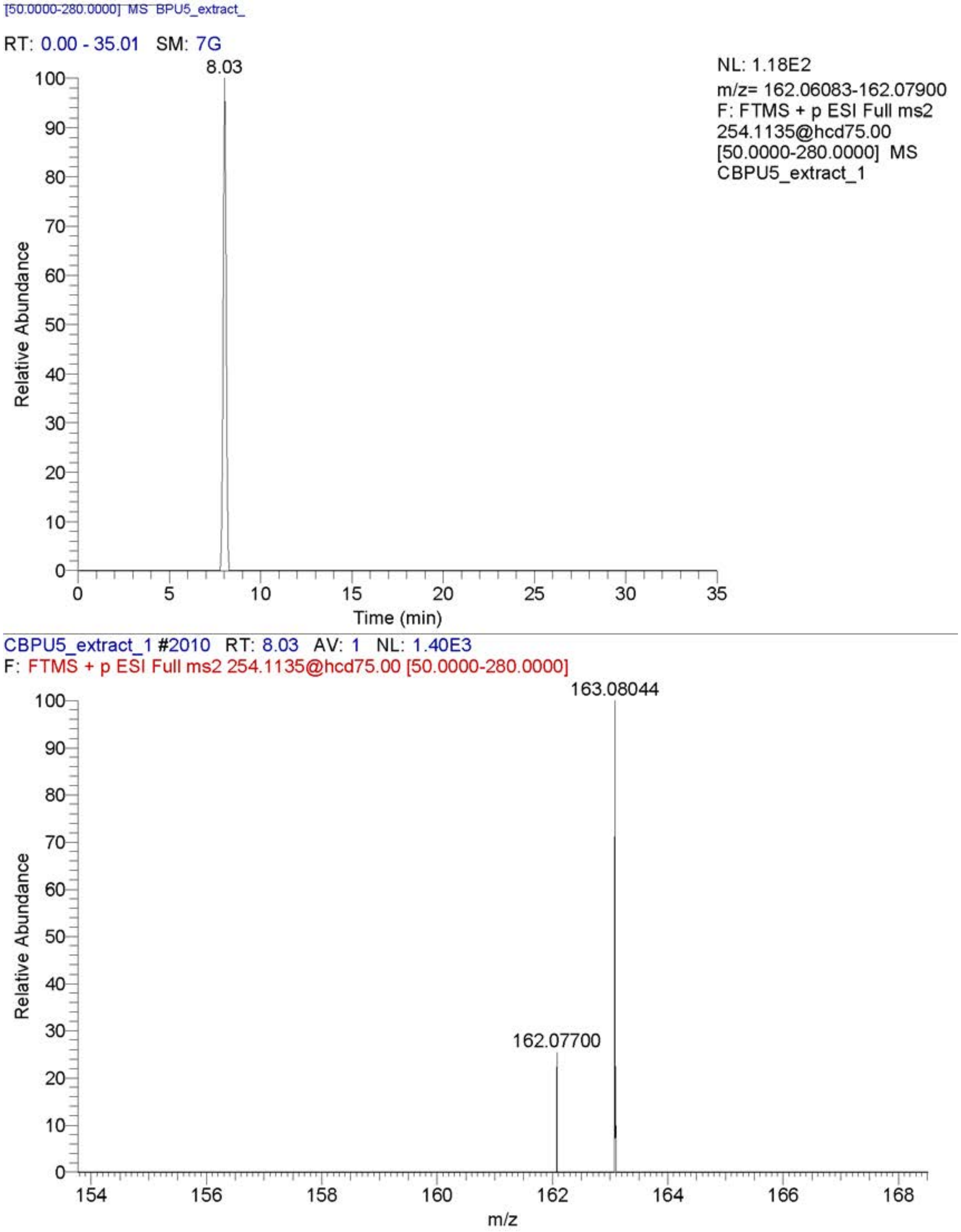

Eurylepta sp.

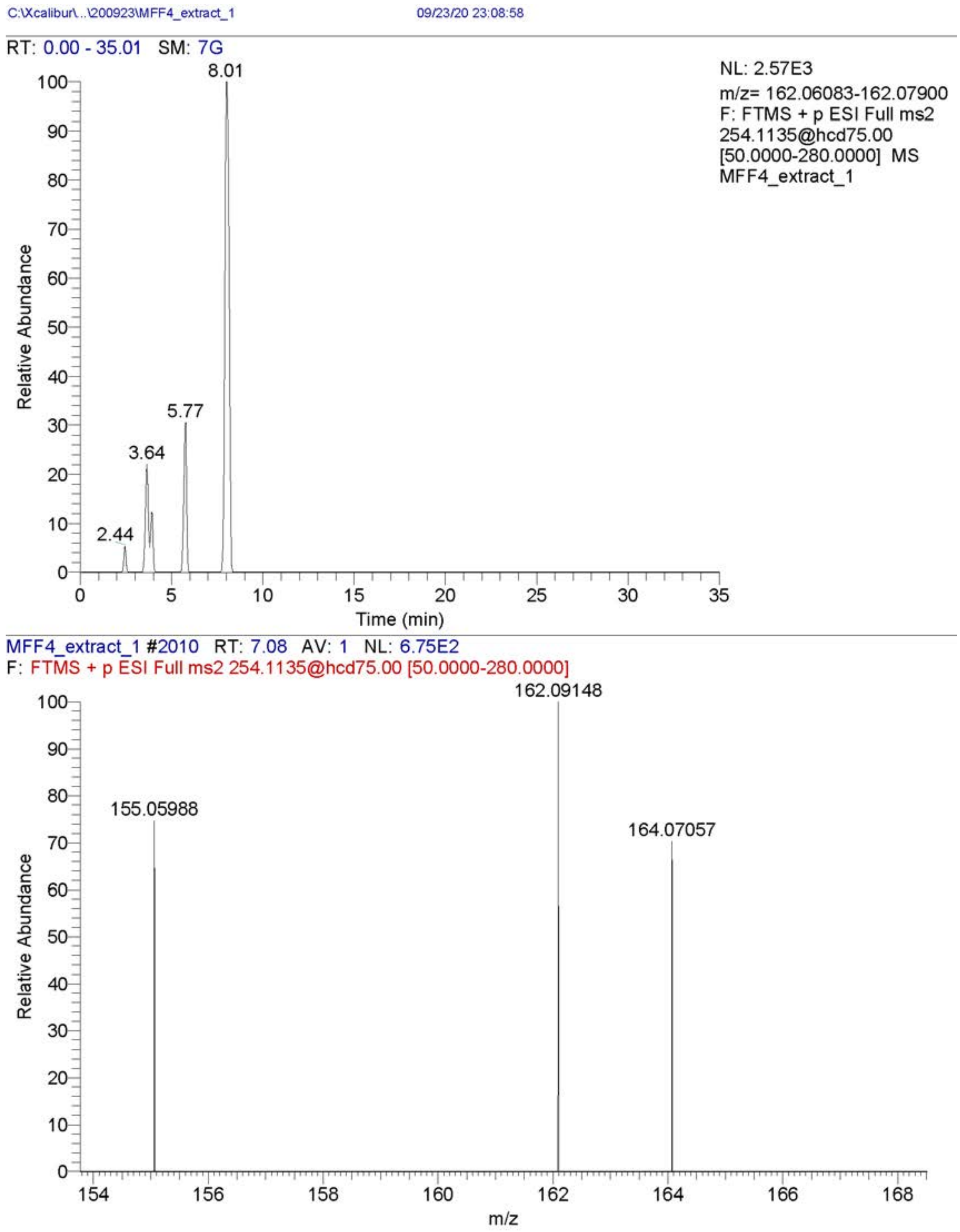
C:\Xcalibur\...\200923\MFF4_extract_1
09/23/20 23:08:58
RT: 0.00 - 35.01 SM: 7G
NL: 2.57E3
m/z= 162.06083-162.07900
F: FTMS + p ESI Full ms2
254.1135@hcd75.00
[50.0000-280.0000] MS
MFF4_extract_1
8.01
5.77
3.64
2.44
Relative Abundance
Time (min)
MFF4_extract_1 #2010 RT: 7.08 AV: 1 NL: 6.75E2
F: FTMS + p ESI Full ms2 254.1135@hcd75.00 [50.0000-280.0000]
162.09148
155.05988
164.07057
Relative Abundance
m/z

S. mcgrathi

Unknown compound #2 (c.f. TTX-11 carboxylic acid (6)) m/z 334.0881

This analogue was first identified during MALDI-MSI as this analogue closely followed the distribution of 11-deoxyTTX (304.1139), upon closer inspection there were several other MS samples that contained this analogue and as such these data are included. There is no PRM data published for this compound however daughter ions 161.07 and 137.01 were regularly found in these samples. The samples that were determined to contain this analyte were, *Stylochus sp. 1* (RT: 7.55), *S. mcgrathi* (RT: 7.57), *S. mcgrathi* (RT: 7.54), *S. mcgrathi* (RT: 7.57), *S. mcgrathi* (RT: 7.59), *E. celerrima* (RT: 7.54), and *E. celerrima* (RT: 7.55).

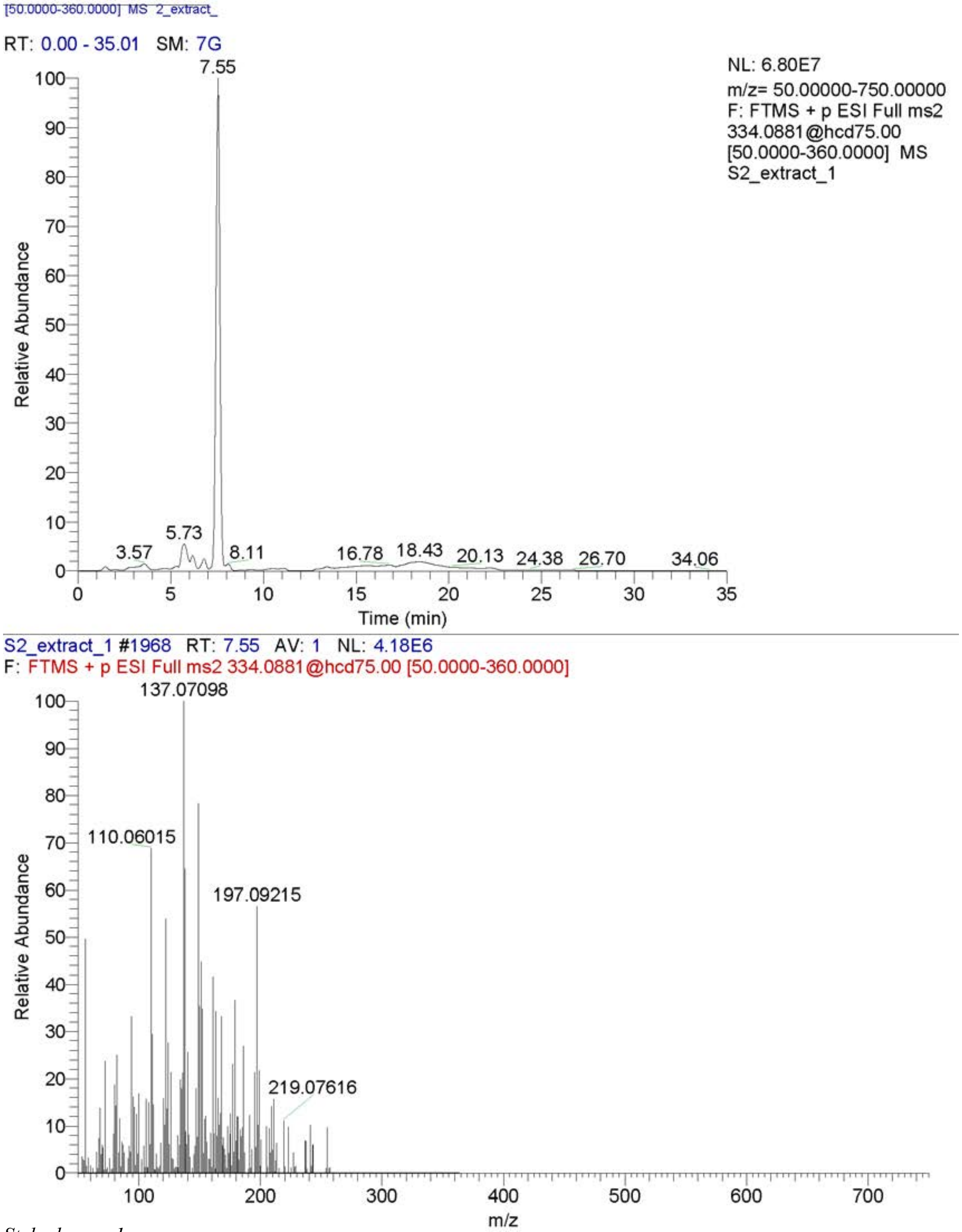

Stylochus sp.1

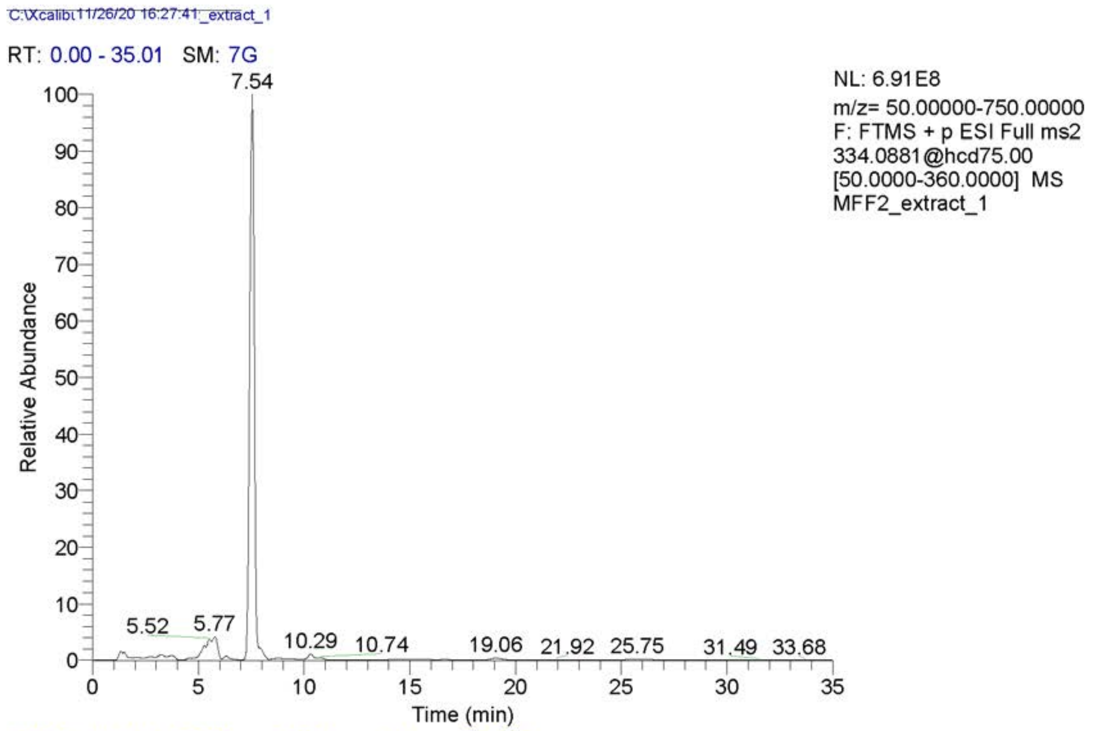
C:\Xcalibu\11/26/20 16:27:41_extract_1
RT: 0.00 - 35.01 SM: 7G
NL: 6.91E8
m/z= 50.00000-750.00000
F: FTMS + p ESI Full ms2
334.0881@hcd75.00
[50.0000-360.0000] MS
MFF2_extract_1
7.54
5.52
5.77
10.29
10.74
19.06
21.92
25.75
31.49
33.68
Relative Abundance
Time (min)

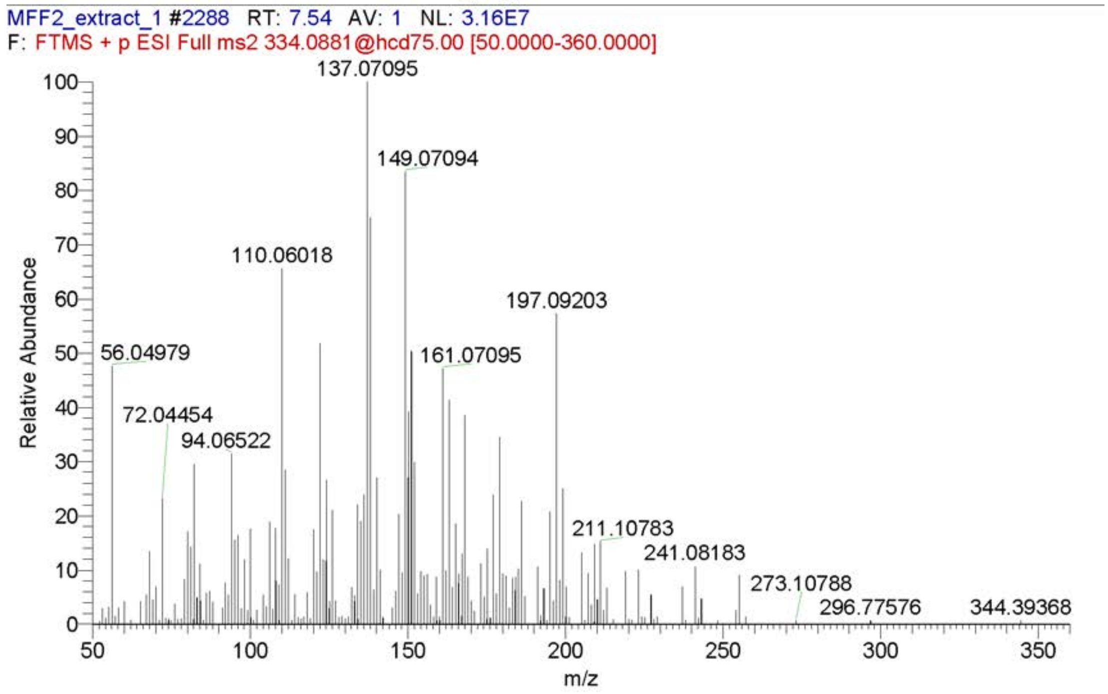
MFF2_extract_1 #2288 RT: 7.54 AV: 1 NL: 3.16E7
F: FTMS + p ESI Full ms2 334.0881@hcd75.00 [50.0000-360.0000]
137.07095
149.07094
110.06018
197.09203
56.04979
161.07095
72.04454
94.06522
211.10783
241.08183
273.10788
296.77576
344.39368
Relative Abundance
m/z

S. mcgrathi

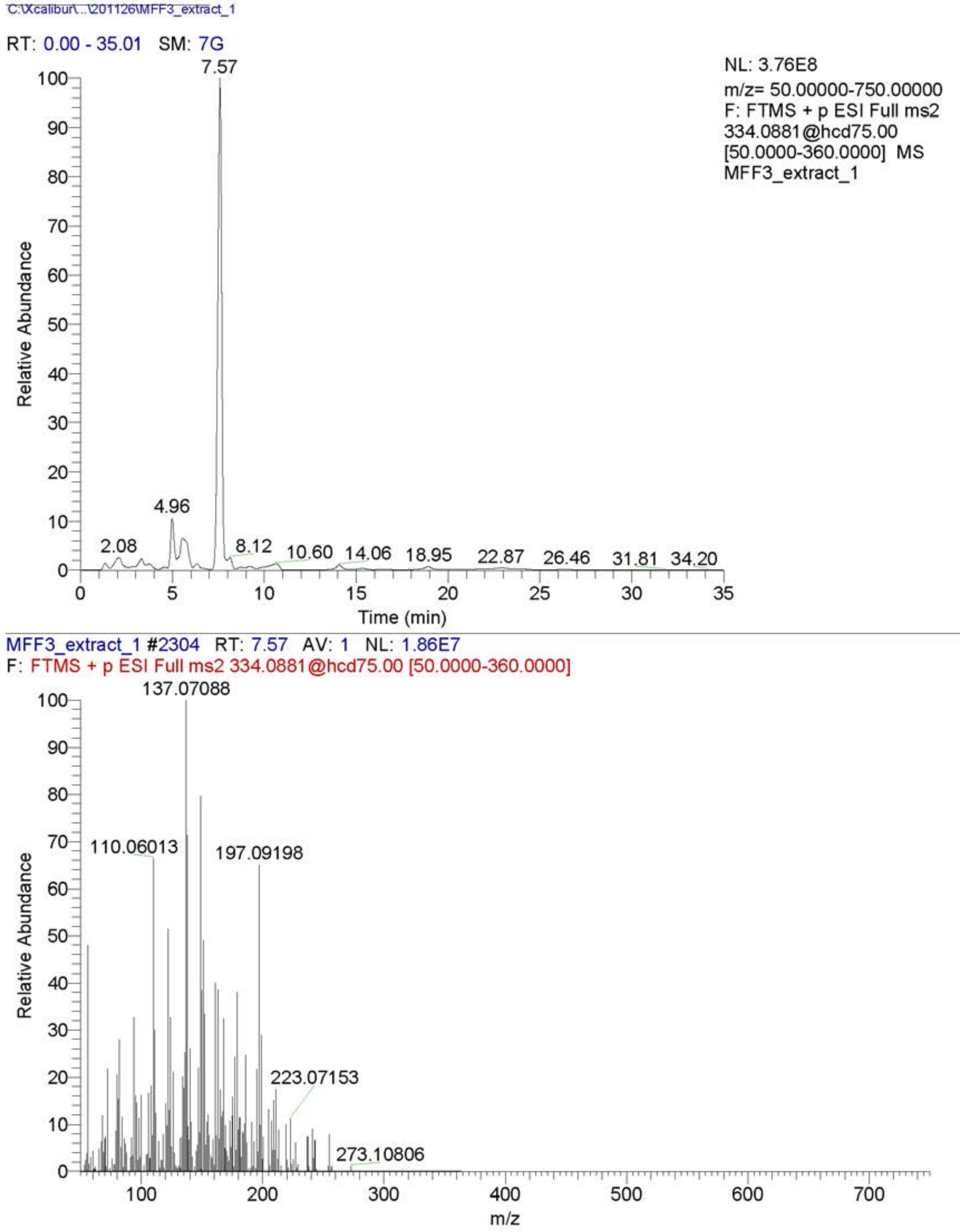

C:\Xcalibur\...\201126\MFF3_extract_1
RT: 0.00 - 35.01 SM: 7G
NL: 3.76E8
m/z= 50.00000-750.00000
F: FTMS + p ESI Full ms2 334.0881@hcd75.00 [50.0000-360.0000] MS
MFF3_extract_1
7.57
4.96
2.08
8.12
10.60
14.06
18.95
22.87
26.46
31.81
34.20
Relative Abundance
Time (min)
MFF3_extract_1 #2304 RT: 7.57 AV: 1 NL: 1.86E7
F: FTMS + p ESI Full ms2 334.0881@hcd75.00 [50.0000-360.0000]
137.07088
110.06013
197.09198
223.07153
273.10806
Relative Abundance
m/z

S. mcgrathi

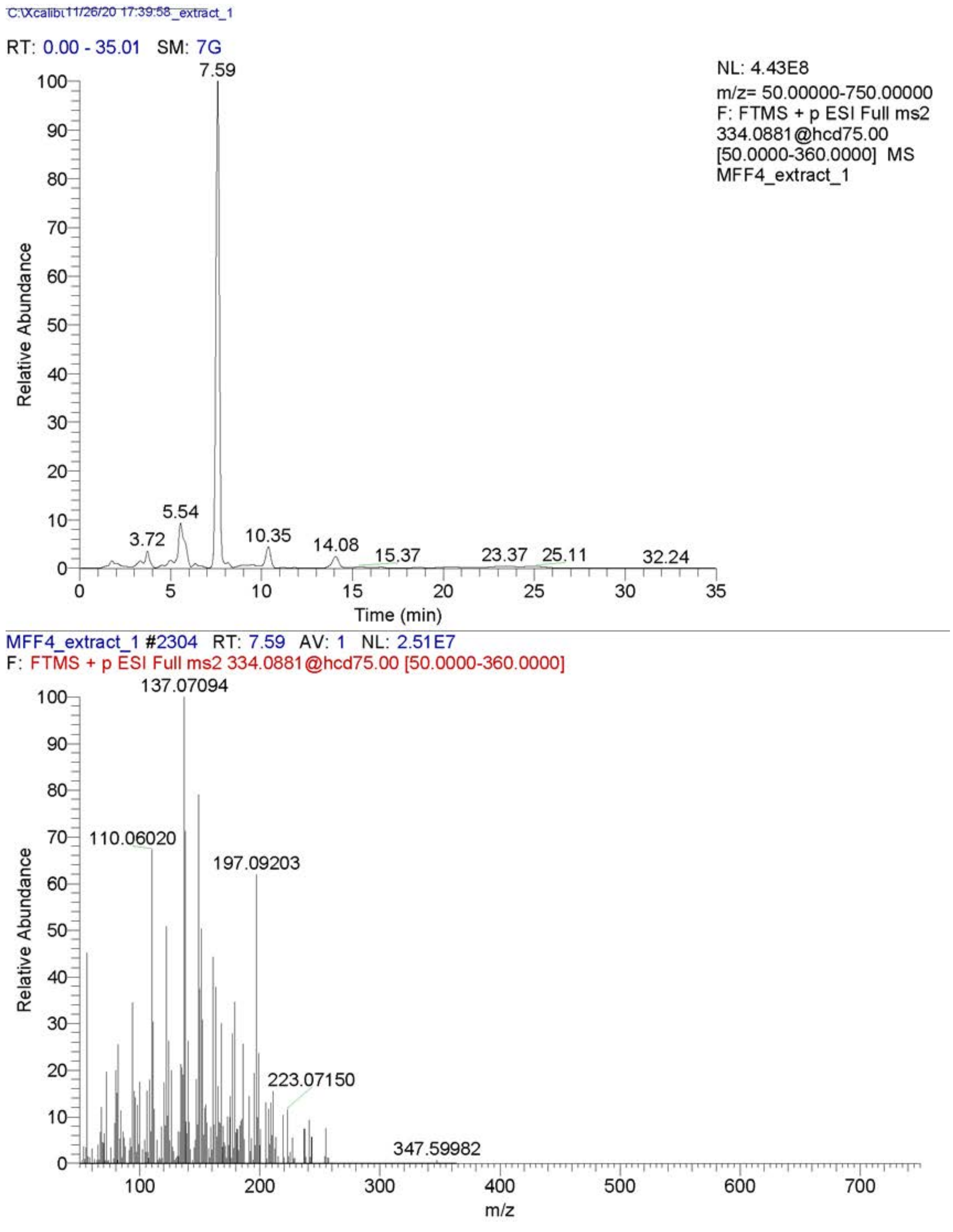
C:\Xcalibu\11/26/20 17:39:58_extract_1
RT: 0.00 - 35.01 SM: 7G
NL: 4.43E8
m/z= 50.00000-750.00000
F: FTMS + p ESI Full ms2 334.0881@hcd75.00 [50.0000-360.0000] MS
MFF4_extract_1
Relative Abundance
Time (min)
7.59
5.54
3.72
10.35
14.08
15.37
23.37
25.11
32.24
MFF4_extract_1 #2304 RT: 7.59 AV: 1 NL: 2.51E7
F: FTMS + p ESI Full ms2 334.0881@hcd75.00 [50.0000-360.0000]
137.07094
110.06020
197.09203
223.07150
347.59982
m/z

S. mcgrathi

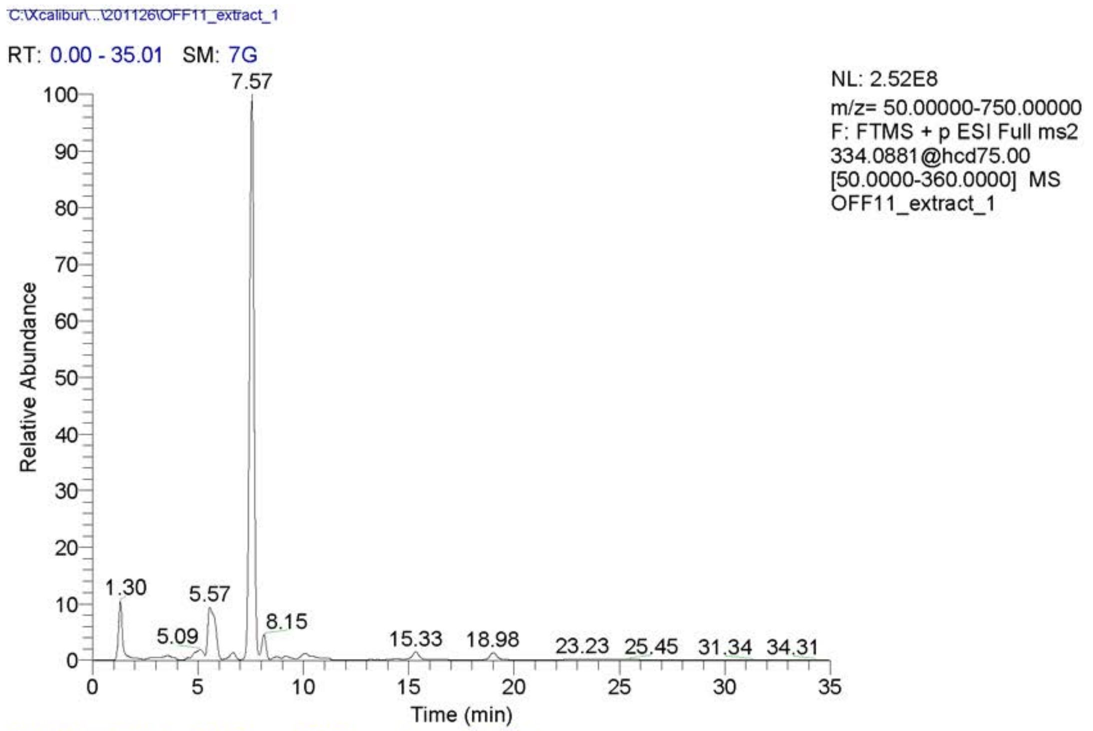
C:\Xcalibur\...\201126\OFF11_extract_1
RT: 0.00 - 35.01 SM: 7G
NL: 2.52E8
m/z= 50.00000-750.00000
F: FTMS + p ESI Full ms2
334.0881@hcd75.00
[50.0000-360.0000] MS
OFF11_extract_1
Relative Abundance
Time (min)
7.57
1.30
5.57
5.09
8.15
15.33
18.98
23.23
25.45
31.34
34.31

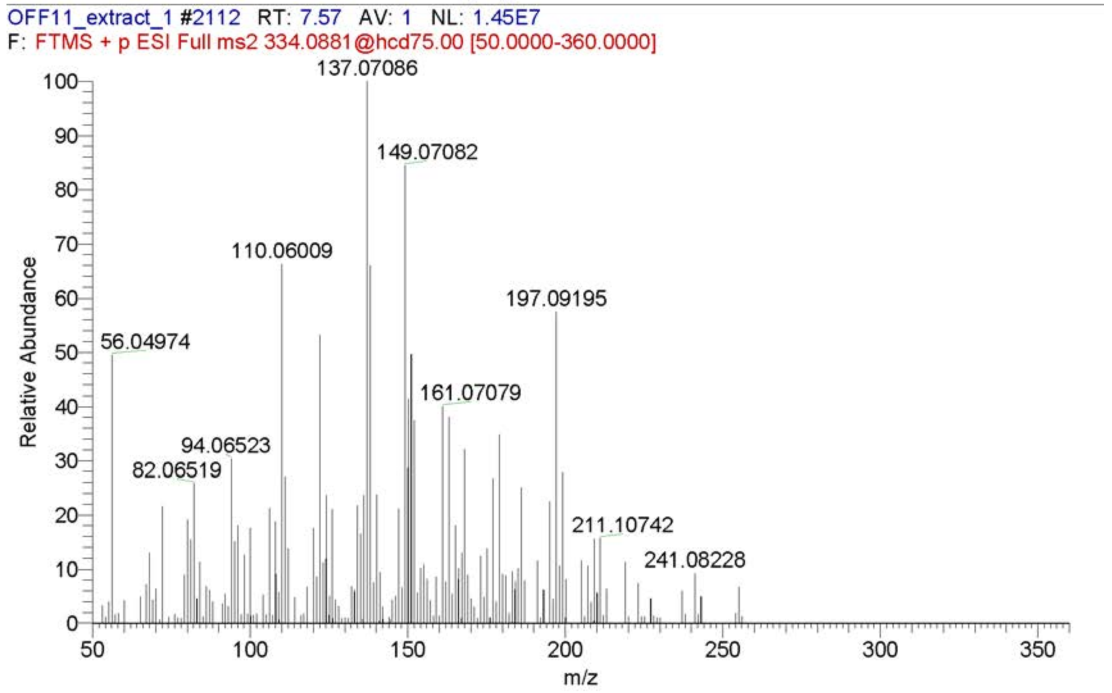
OFF11_extract_1 #2112 RT: 7.57 AV: 1 NL: 1.45E7
F: FTMS + p ESI Full ms2 334.0881@hcd75.00 [50.0000-360.0000]
Relative Abundance
m/z
137.07086
149.07082
110.06009
197.09195
56.04974
161.07079
94.06523
82.06519
211.10742
241.08228

S. mcgrathi

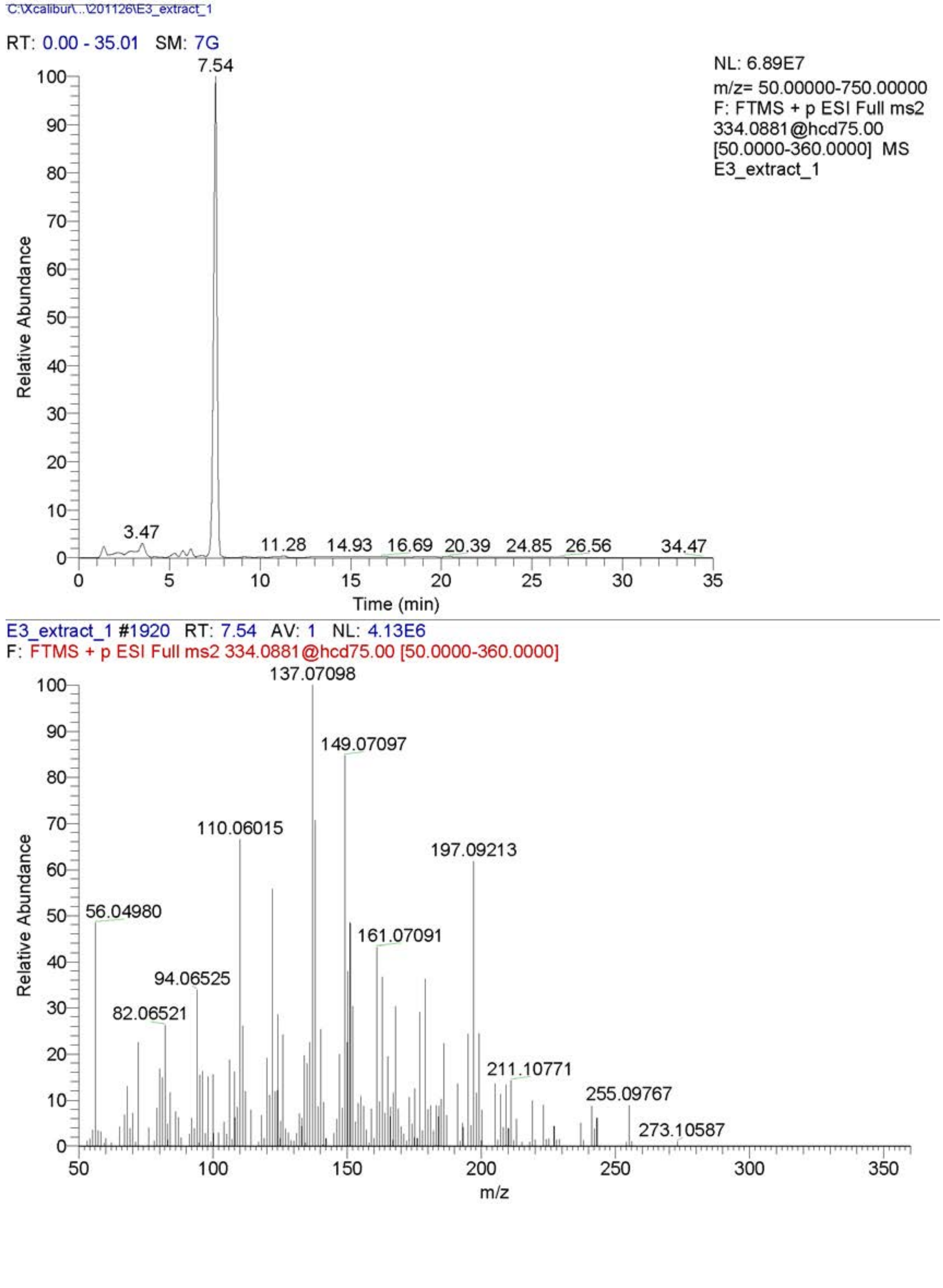

C:\Xcalibur\...\201126\E3_extract_1
RT: 0.00 - 35.01 SM: 7G
NL: 6.89E7
m/z= 50.00000-750.00000
F: FTMS + p ESI Full ms2 334.0881@hcd75.00 [50.0000-360.0000] MS
E3_extract_1
Relative Abundance
Time (min)
7.54
3.47
11.28
14.93
16.69
20.39
24.85
26.56
34.47
E3_extract_1 #1920 RT: 7.54 AV: 1 NL: 4.13E6
F: FTMS + p ESI Full ms2 334.0881@hcd75.00 [50.0000-360.0000]
137.07098
149.07097
110.06015
197.09213
56.04980
161.07091
94.06525
82.06521
211.10771
255.09767
273.10587
m/z

E. celerrima

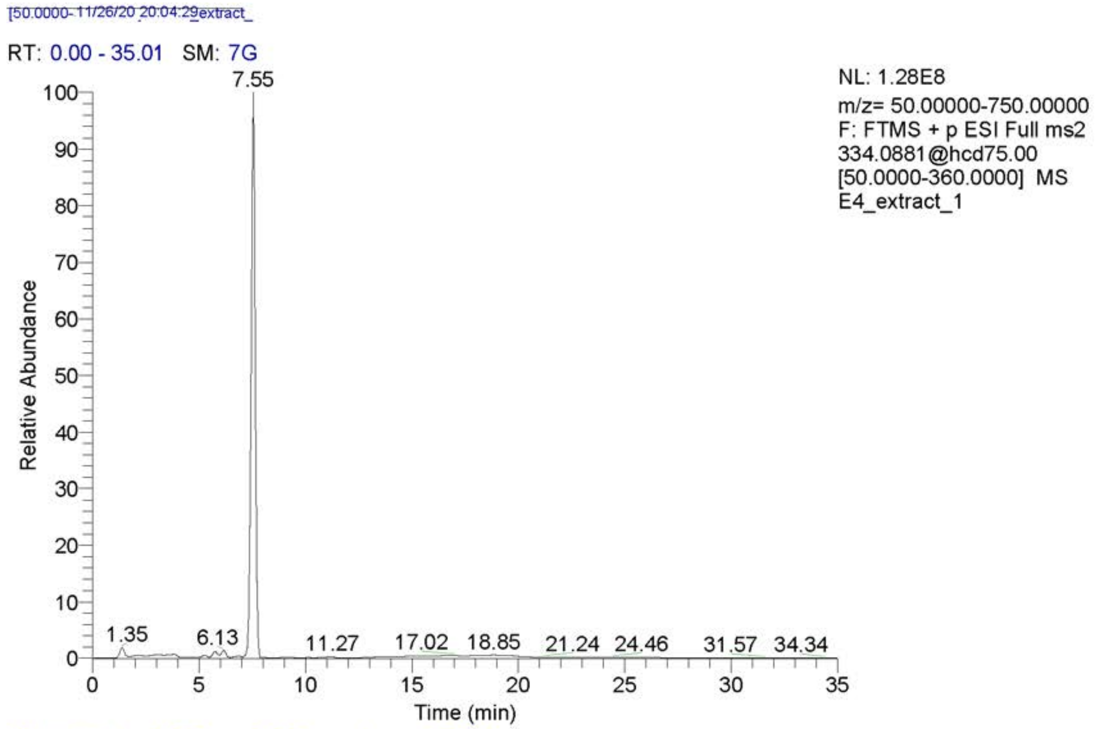
[50.0000- 11/26/20 20:04:29extract_
RT: 0.00 - 35.01 SM: 7G
NL: 1.28E8
m/z= 50.00000-750.00000
F: FTMS + p ESI Full ms2 334.0881@hcd75.00 [50.0000-360.0000] MS
E4_extract_1
Relative Abundance
Time (min)
7.55
1.35
6.13
11.27
17.02
18.85
21.24
24.46
31.57
34.34

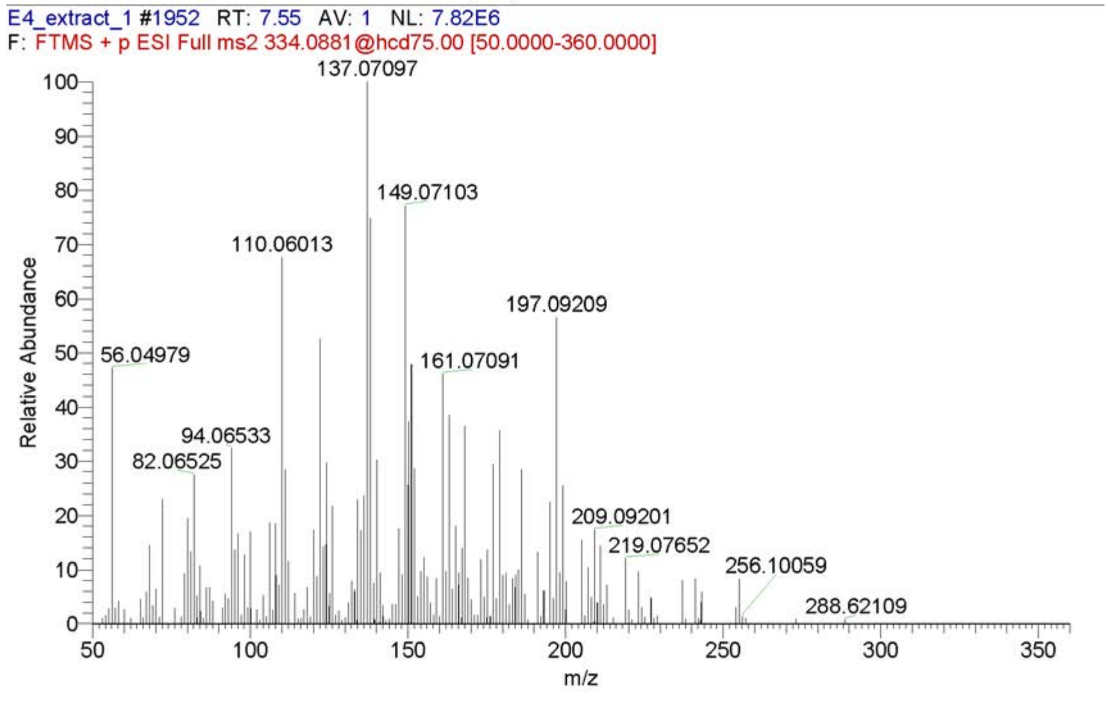
E4_extract_1 #1952 RT: 7.55 AV: 1 NL: 7.82E6
F: FTMS + p ESI Full ms2 334.0881@hcd75.00 [50.0000-360.0000]
Relative Abundance
m/z
137.07097
149.07103
110.06013
197.09209
56.04979
161.07091
94.06533
82.06525
209.09201
219.07652
256.10059
288.62109

E. celerrima

Unknown compound m/z 332.1000-332.3000

This compound was identified during MALDI-MSI because it co-localized with 11-deoxyTTX (**2**). Examination of HILIC-PRM data showed this compound eluted around 16.5 min with a PRM peak at 203.1502 ($C_8H_{19}N_4O_2$). The samples that were determined to contain this analyte were, *S. mcgrathi* (RT: 16.50), *S. mcgrathi* (RT: 16.98), *S. mcgrathi* (RT: 16.61), *S. mcgrathi* (RT: 16.96), *S. mcgrathi* (RT: 16.06), *S. mcgrathi* (RT: 16.39), *S. mcgrathi* (RT: 16.52), *S. mcgrathi* (RT: 16.97).

The exact mass of this compound was 332.19278 suggesting a molecular formula of $C_{13}H_{26}N_5O_5$ (Δmmu = –0.07)

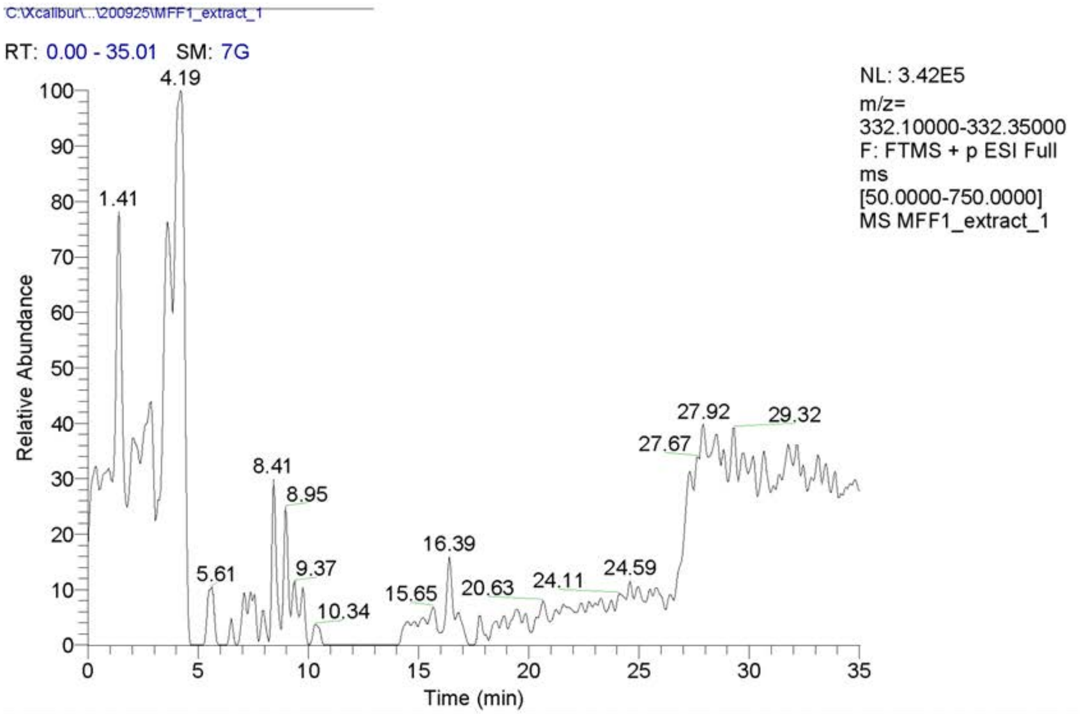

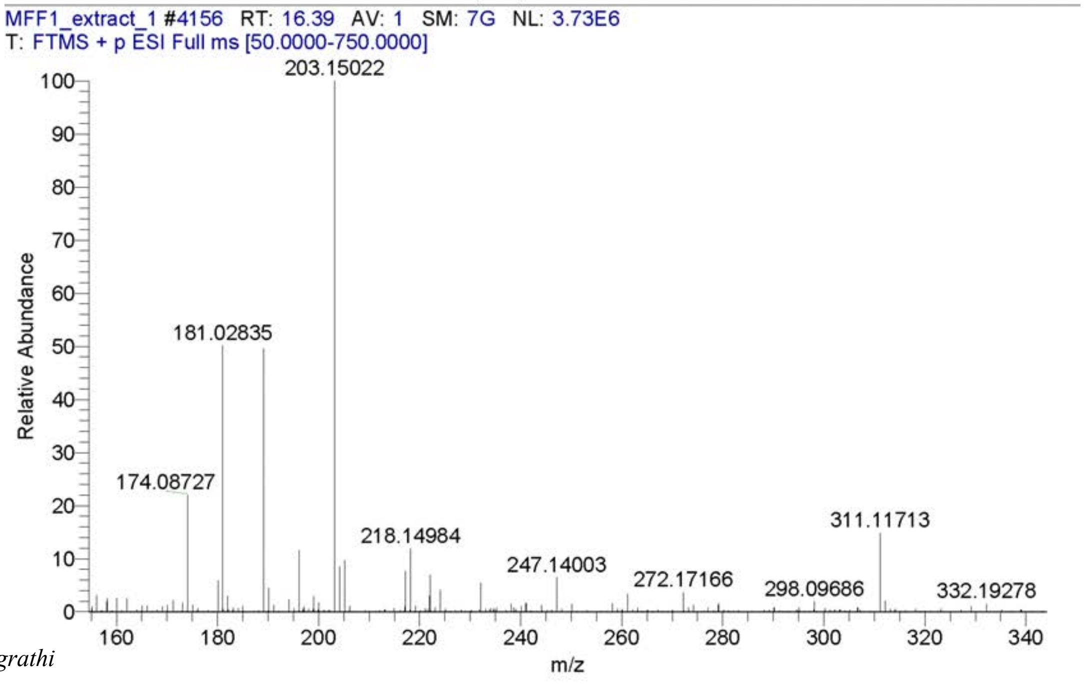

S. mcgrathi

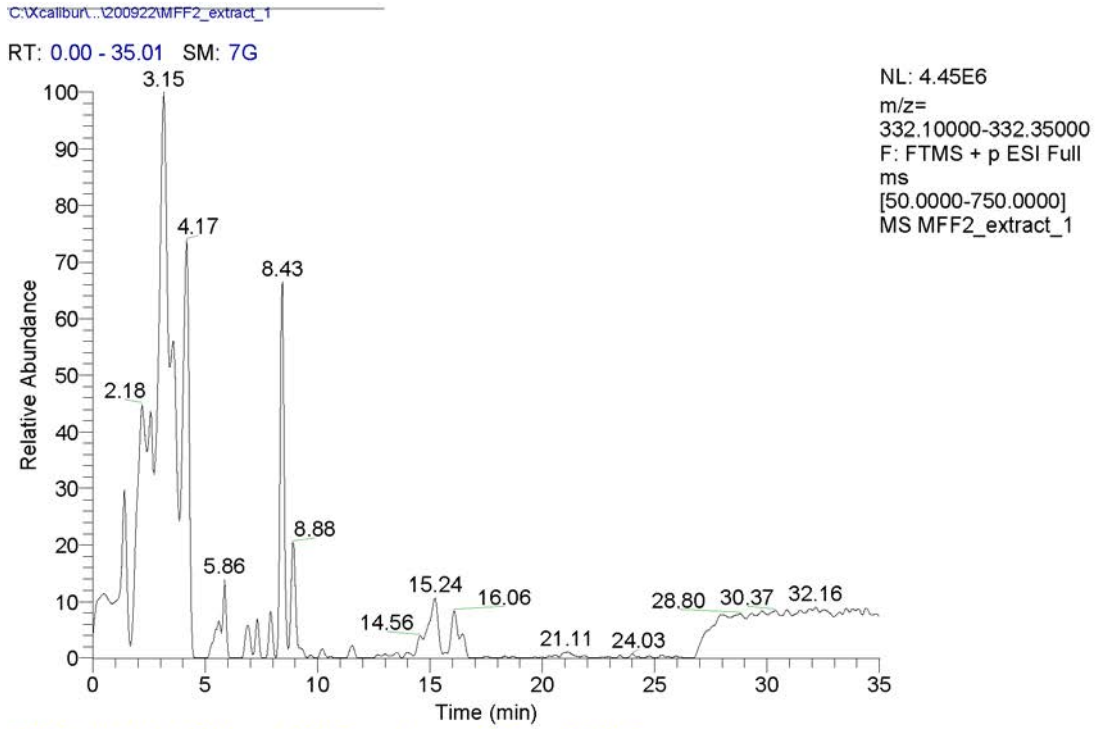

C:\Xcalibur\...\200922\MFF2_extract_1
RT: 0.00 - 35.01 SM: 7G
NL: 4.45E6
m/z=
332.10000-332.35000
F: FTMS + p ESI Full
ms
[50.0000-750.0000]
MS MFF2_extract_1
Relative Abundance
Time (min)
3.15
4.17
8.43
2.18
8.88
5.86
15.24
16.06
14.56
28.80
30.37
32.16
21.11
24.03

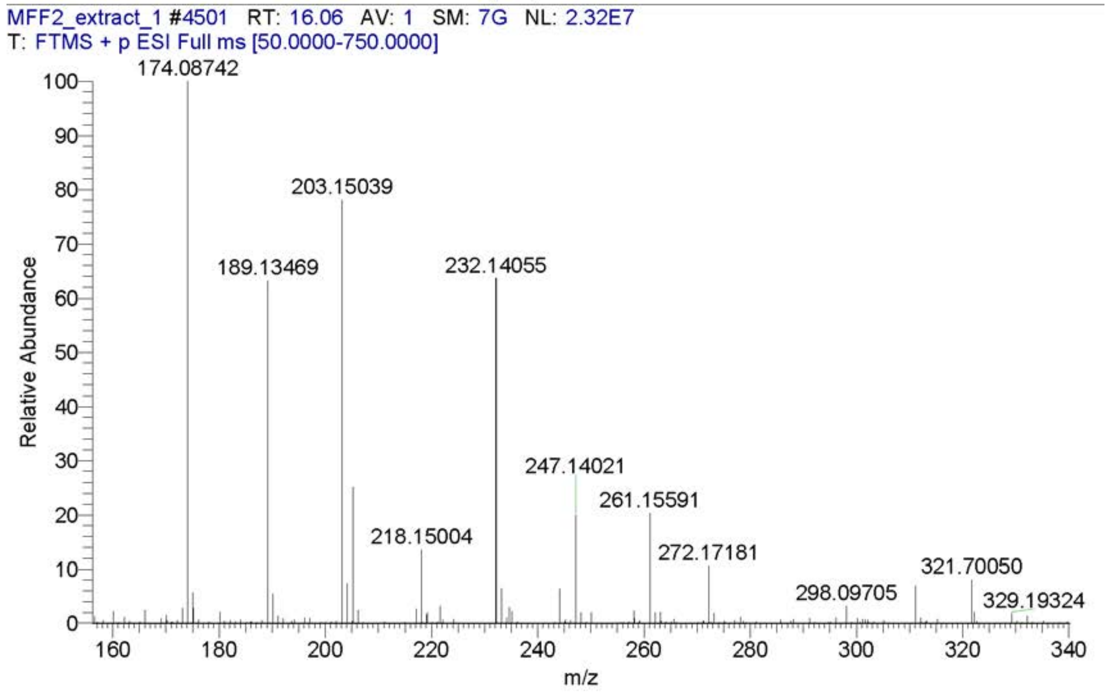

MFF2_extract_1 #4501 RT: 16.06 AV: 1 SM: 7G NL: 2.32E7
T: FTMS + p ESI Full ms [50.0000-750.0000]
Relative Abundance
m/z
174.08742
203.15039
189.13469
232.14055
247.14021
261.15591
218.15004
272.17181
321.70050
298.09705
329.19324

S. mcgrathi

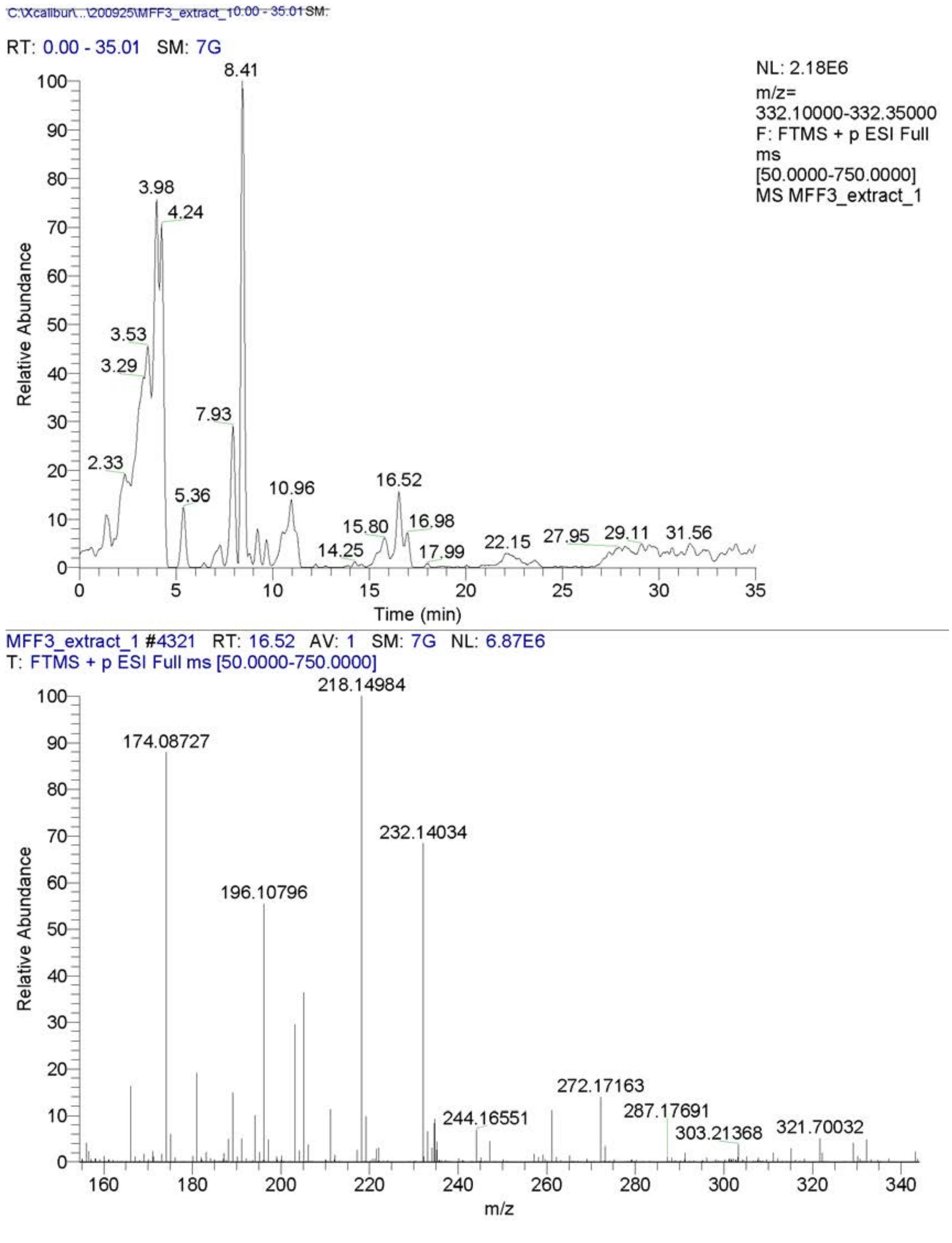
C:\Xcalibur\...\200925\MFF3_extract_1 0.00 - 35.01 SM:
RT: 0.00 - 35.01 SM: 7G
NL: 2.18E6
m/z=
332.10000-332.35000
F: FTMS + p ESI Full
ms
[50.0000-750.0000]
MS MFF3_extract_1
Relative Abundance
Time (min)
MFF3_extract_1 #4321 RT: 16.52 AV: 1 SM: 7G NL: 6.87E6
T: FTMS + p ESI Full ms [50.0000-750.0000]
218.14984
174.08727
232.14034
196.10796
272.17163
287.17691
244.16551
303.21368
321.70032
m/z

S. mcgrathi

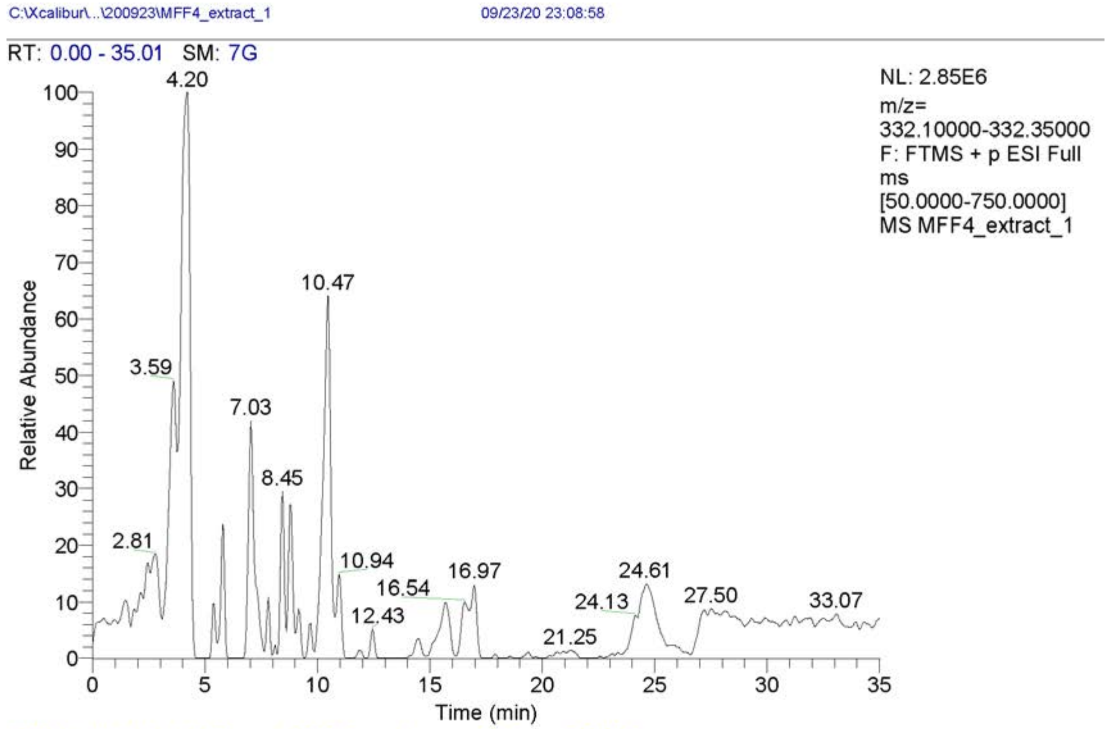
C:\Xcalibur\...\200923\MFF4_extract_1
09/23/20 23:08:58
RT: 0.00 - 35.01 SM: 7G
NL: 2.85E6
m/z= 332.10000-332.35000
F: FTMS + p ESI Full ms [50.0000-750.0000]
MS MFF4_extract_1
Relative Abundance
Time (min)
4.20
10.47
3.59
7.03
8.45
2.81
10.94
16.54
16.97
12.43
24.61
24.13
27.50
33.07
21.25

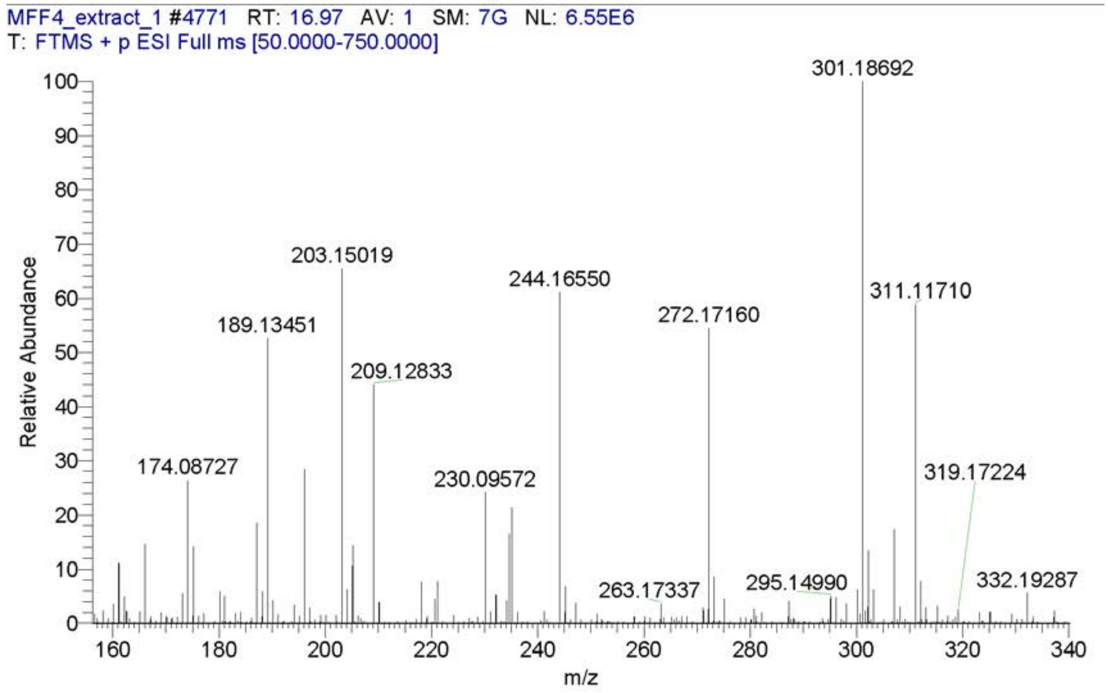
MFF4_extract_1 #4771 RT: 16.97 AV: 1 SM: 7G NL: 6.55E6
T: FTMS + p ESI Full ms [50.0000-750.0000]
Relative Abundance
m/z
301.18692
203.15019
244.16550
311.11710
272.17160
189.13451
209.12833
174.08727
230.09572
319.17224
263.17337
295.14990
332.19287

S. mcgrathi

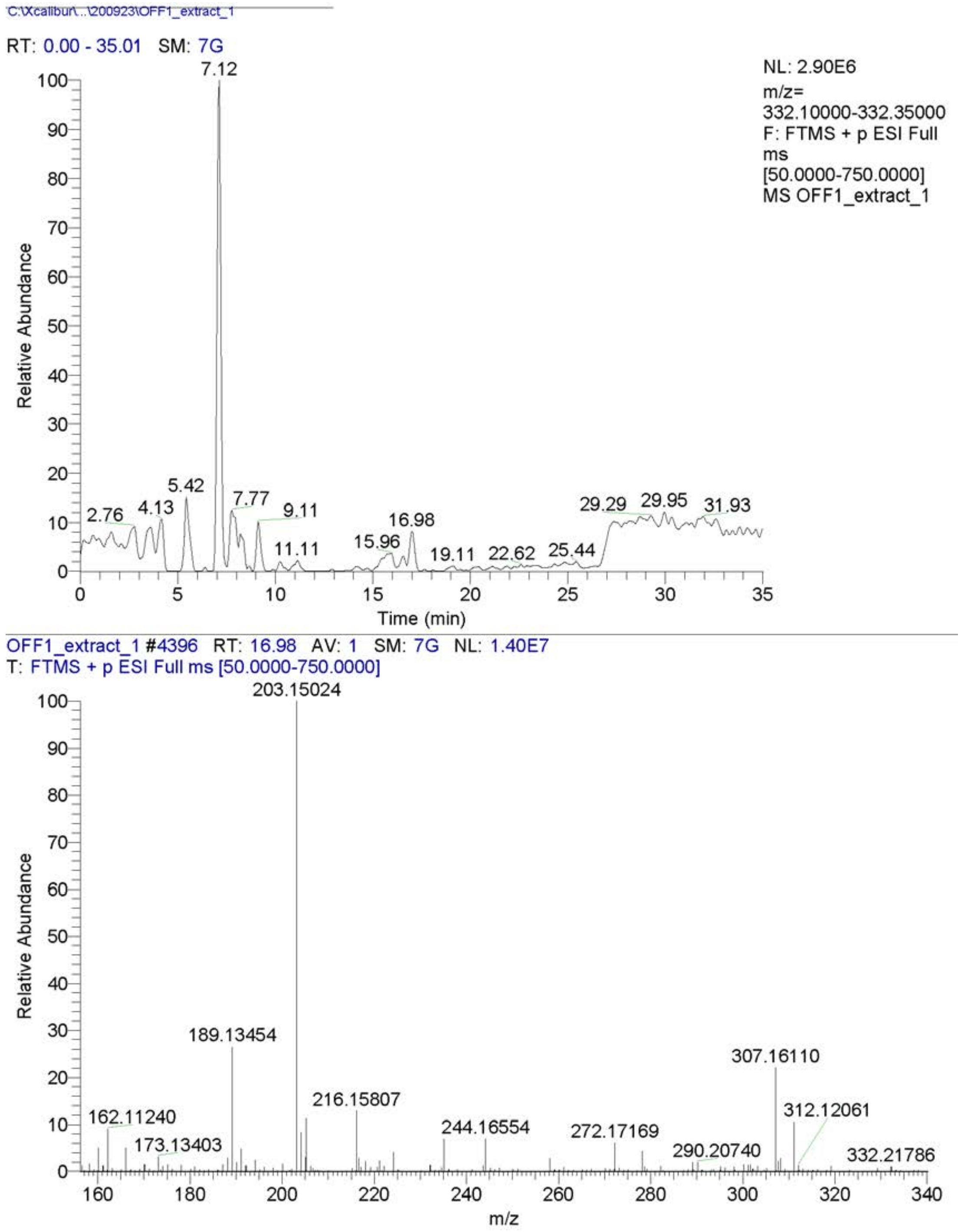
C:\Xcalibur\...\200923\OFF1_extract_1
RT: 0.00 - 35.01 SM: 7G
NL: 2.90E6
m/z= 332.10000-332.35000
F: FTMS + p ESI Full ms [50.0000-750.0000]
MS OFF1_extract_1
7.12
5.42
2.76
4.13
7.77
9.11
11.11
15.96
16.98
19.11
22.62
25.44
29.29
29.95
31.93
Relative Abundance
Time (min)
OFF1_extract_1 #4396 RT: 16.98 AV: 1 SM: 7G NL: 1.40E7
T: FTMS + p ESI Full ms [50.0000-750.0000]
203.15024
189.13454
162.11240
173.13403
216.15807
244.16554
272.17169
290.20740
307.16110
312.12061
332.21786
Relative Abundance
m/z

S. mcgrathi

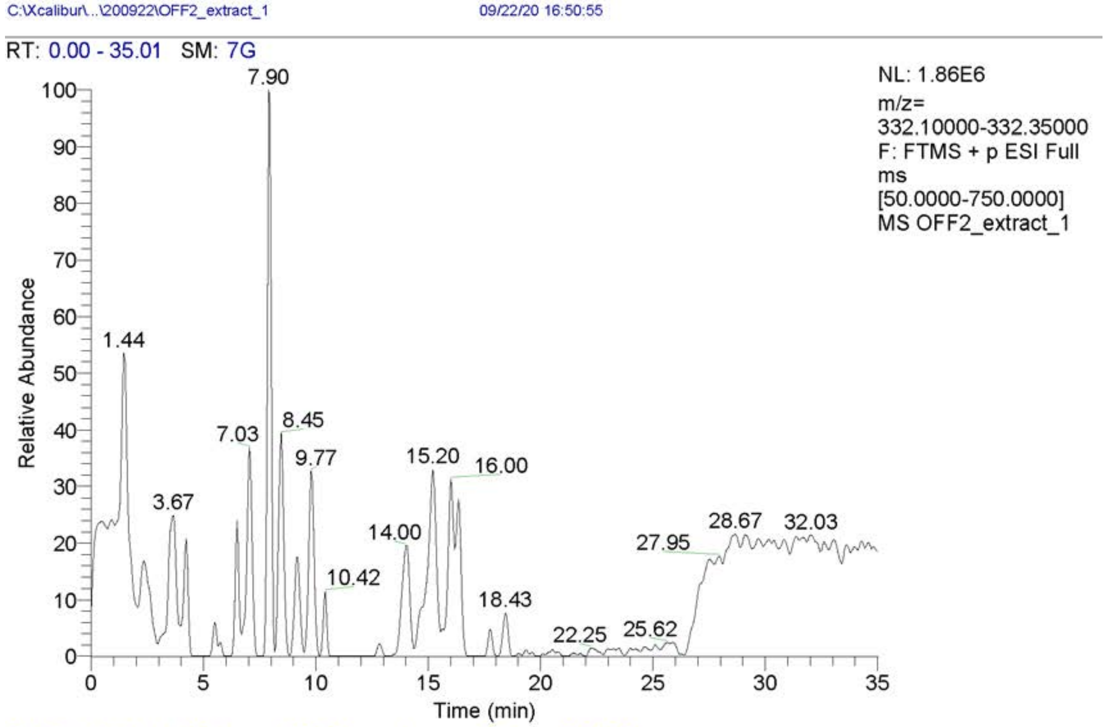

C:\Xcalibur\...\200922\OFF2_extract_1
09/22/20 16:50:55
RT: 0.00 - 35.01 SM: 7G
NL: 1.86E6
m/z=
332.10000-332.35000
F: FTMS + p ESI Full
ms
[50.0000-750.0000]
MS OFF2_extract_1
Relative Abundance
Time (min)
7.90
1.44
3.67
7.03
8.45
9.77
10.42
14.00
15.20
16.00
18.43
22.25
25.62
27.95
28.67
32.03

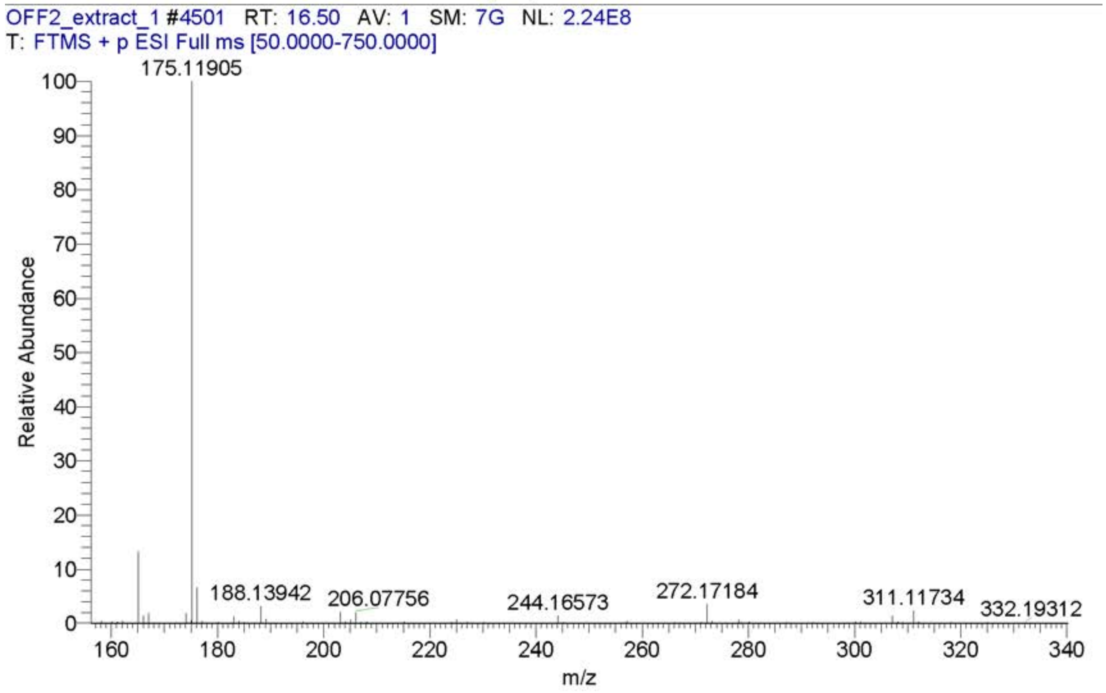

OFF2_extract_1 #4501 RT: 16.50 AV: 1 SM: 7G NL: 2.24E8
T: FTMS + p ESI Full ms [50.0000-750.0000]
Relative Abundance
m/z
175.11905
188.13942
206.07756
244.16573
272.17184
311.11734
332.19312

S. mcgrathi

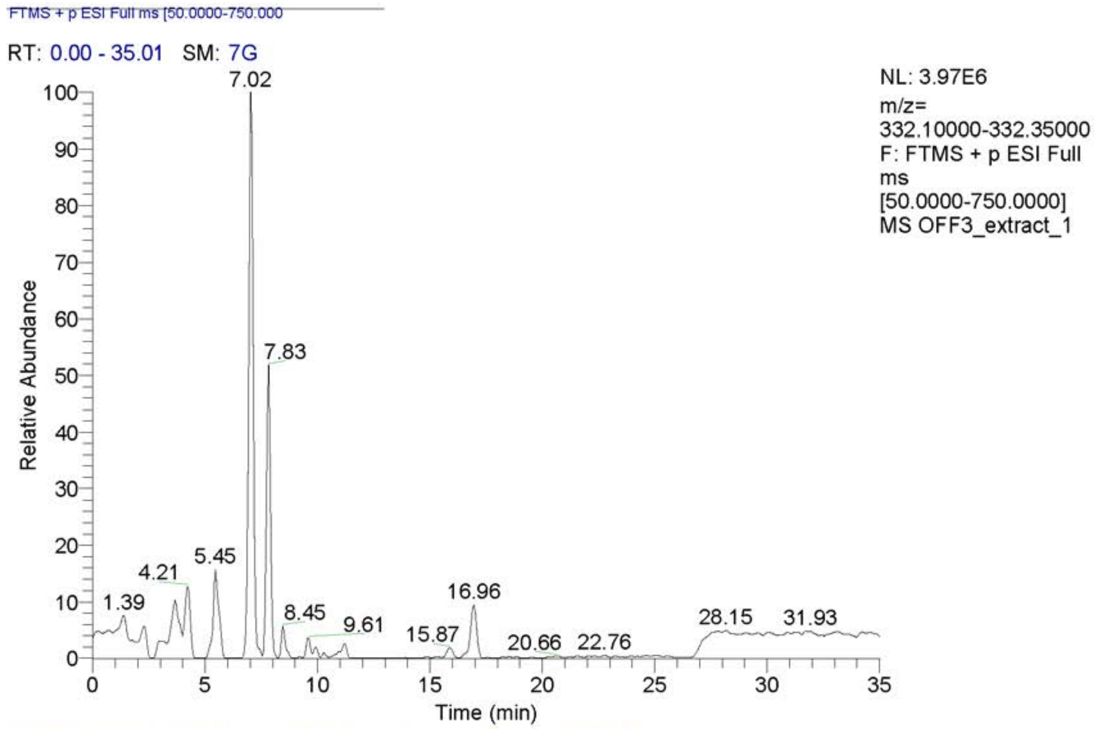
FTMS + p ESI Full ms [50.0000-750.000
RT: 0.00 - 35.01 SM: 7G
NL: 3.97E6
m/z=
332.10000-332.35000
F: FTMS + p ESI Full
ms
[50.0000-750.0000]
MS OFF3_extract_1
Relative Abundance
Time (min)
7.02
7.83
1.39
4.21
5.45
8.45
9.61
15.87
16.96
20.66
22.76
28.15
31.93

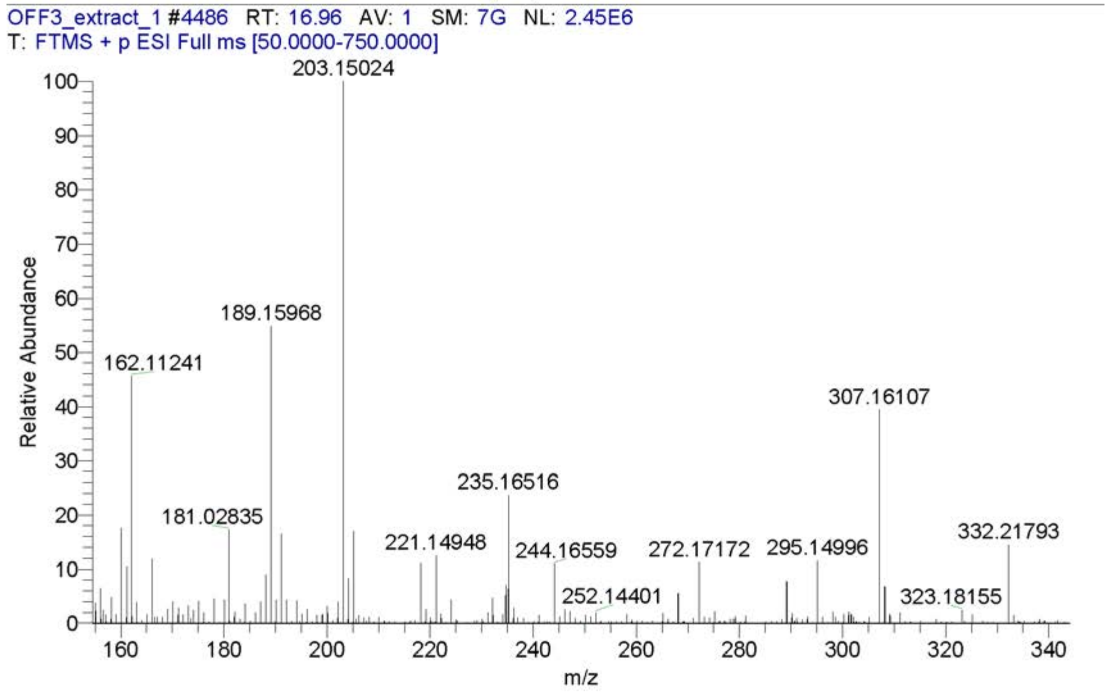
OFF3_extract_1 #4486 RT: 16.96 AV: 1 SM: 7G NL: 2.45E6
T: FTMS + p ESI Full ms [50.0000-750.0000]
Relative Abundance
m/z
203.15024
189.15968
162.11241
307.16107
235.16516
181.02835
332.21793
221.14948
244.16559
272.17172
295.14996
252.14401
323.18155

S. mcgrathi

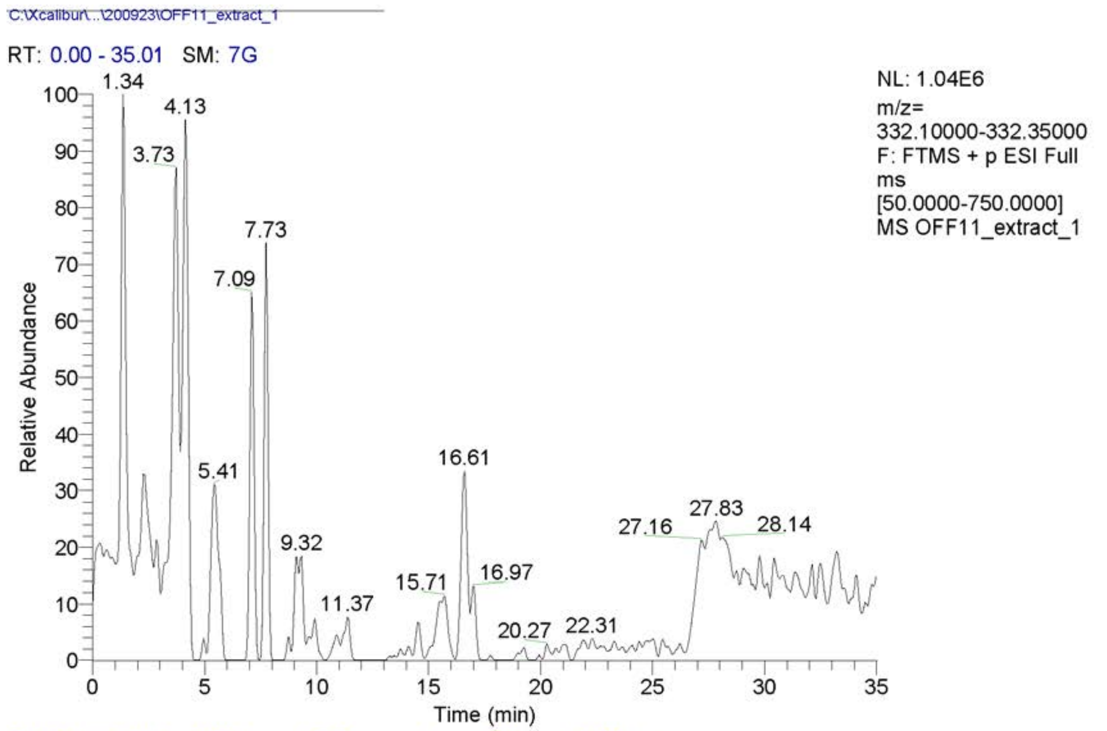
C:\Xcalibur\...\200923\OFF11_extract_1
RT: 0.00 - 35.01 SM: 7G
NL: 1.04E6
m/z= 332.10000-332.35000
F: FTMS + p ESI Full ms [50.0000-750.0000]
MS OFF11_extract_1
Relative Abundance
Time (min)
1.34
4.13
3.73
7.73
7.09
5.41
16.61
27.83
27.16
28.14
9.32
16.97
15.71
11.37
20.27
22.31

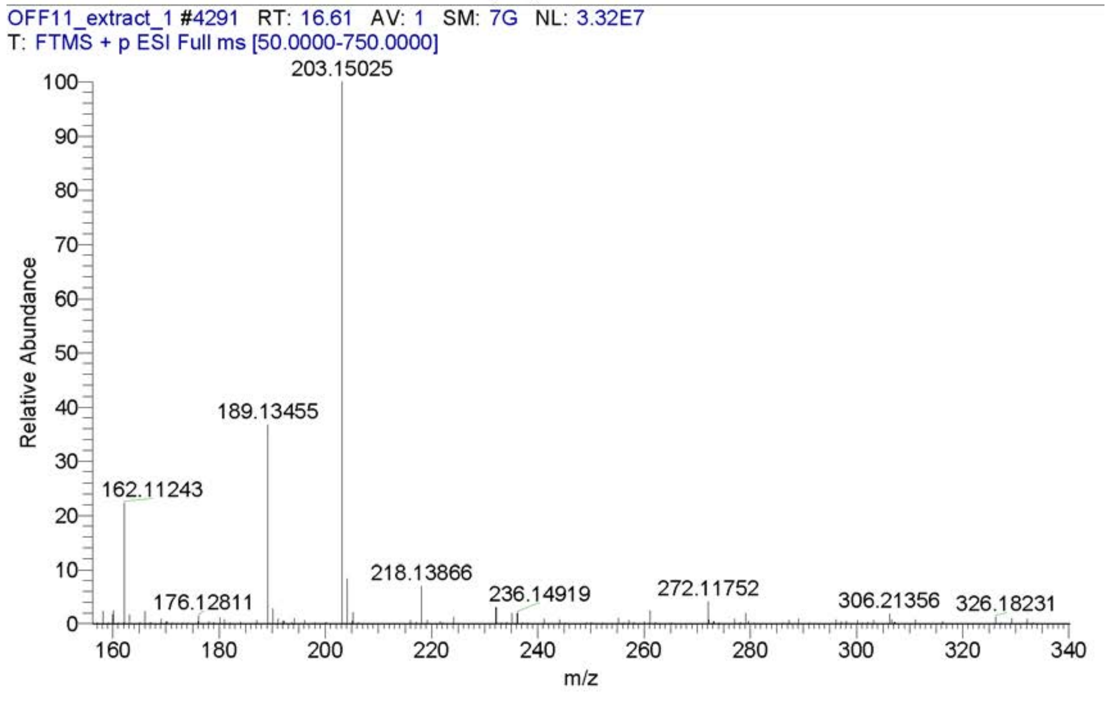
OFF11_extract_1 #4291 RT: 16.61 AV: 1 SM: 7G NL: 3.32E7
T: FTMS + p ESI Full ms [50.0000-750.0000]
Relative Abundance
m/z
203.15025
189.13455
162.11243
218.13866
176.12811
236.14919
272.11752
306.21356
326.18231

S. mcgrathi

References

1. Bane, V.; Lehane, M.; Dikshit, M.; O'Riordan, A.; Furey, A., Tetrodotoxin: Chemistry, toxicity, source, distribution and detection. *Toxins* **2014,** *6*, 693-755.
2. Bane, V.; Hutchinson, S.; Sheehan, A.; Brosnan, B.; Barnes, P.; Lehane, M.; Furey, A., LC-MS/MS method for the determination of tetrodotoxin (TTX) on a triple quadruple mass spectrometer. *Food Additives & Contaminants: Part A* **2016,** *33*, 1728-1740.
3. Bane, V.; Brosnan, B.; Barnes, P.; Lehane, M.; Furey, A., High-resolution mass spectrometry analysis of tetrodotoxin (TTX) and its analogues in puffer fish and shellfish. *Food Additives & Contaminants: Part A* **2016,** *33*, 1468-1489.
